# Supplementary material for: DNA damage repair kinase DNA‐PK and cGAS synergize to induce cancer‐related inflammation in glioblastoma
Source: EMBO J. 2022 Dec 27;42(7):e111961. doi: 10.15252/embj.2022111961 (PMC10068334; doi:10.15252/embj.2022111961)
Supplement: Supplementary file 2 — Table EV1 [file EMBJ-42-e111961-s003.docx]

**Table EV1: Oligonucleotides**

| **qPCR Primers** | **Fwd** | **Rev** |
| --- | --- | --- |
| **Human** |  |  |
| *GAPDH* | CTGGCGTCTTCACCACCATGG | CATCACGCCACAGTTTCCCGG |
| *IFNB* | GAATGGGAGGCTTGAATACTGCCT | TAGCAAAGATGTTCTGGAGCATCTC |
| *ACTB* | GGACTTCGAGCAAGAGATGG | AGCACTGTGTTGGCGTACAG |
| *CXCL10* | GAAAGCAGTTAGCAAGGAAAGGTG | ATGTAGGGAAGTGATGGGAGAGG |
| *CCL2* | AGAATCACCAGCAGCAAGTGTCC | TCCTGAACCCACTTCTGCTTGG |
| *CCL3* | GGCTCTCTGCAACCAGTTCT | TGAAATTCTGTGGAATCTGCC |
| *CCL5* | CCTGCTGCTTTGCCTACATTGC | ACACACTTGGCGGTTCTTTCGG |
| *MXA* | ATGAGCTAATCACCCTGGAG | ATACCCAATGTCAGCAGGC |
| *OAS1* | AGGAAAGGTGCTTCCGAGGTAG | GGACTGAGGAAGACAACCAGGT |
| *IFIT1* | GCCTTGCTGAAGTGTGGAGGAA | ATCCAGGCGATAGGCAGAGATC |
| *IFIT2* | GGAGCAGATTCTGAGGCTTTGC | GGATGAGGCTTCCAGACTCCAA |
| *IL6* | GACCCAACCACAAATGCCAG | GTGCCCATGCTACATTTGCC |
| *IFNL2/3* | CTGCCACATAGCCCAGTTCA | AGCGACTCTTCTAAGGCATCT |
| **Mouse** |  |  |
| *Gapdh* | TTCACCACCATGGAGAAGGC | GGCATCGACTGTGGTCATGA |
| *Ifnb* | CTGCGTTCCTGCTGTGCTTCTCCA | TTCTCCGTCATCTCCATAGGGATC |
| *Cxcl10* | ATGACGGGCCAGTGAGAATG | TCAACACGTGGGCAGGATAG |
| **Zebrafish** |  |  |
| *ef1a* | AGAAGGCTGCCAAGACCAAG | AGAGGTTGGGAAGAACACGC |
| *tnfa* | GGAGAGTTGCCTTTACCGCT | CCTGGGTCTTATGGAGCGTG |
| *cxcl11* | ACTCAACATGGTGAAGCCAGTGCT | CTTCAGCGTGGCTATGACTTCCAT |
| *arg2* | GAAGCCGTTCCTGTCTGCCA | TCGGCCTTTGCTTCCTTGCC |
| *il10* | TCAGAGCAGGAGAGTCGAATGCA | CGATTGGGGTTGTGGAGTGCTT |
| **RNA Guides** | **Fwd** | **Rev** |
| CTRL | CACCGAGCACGTAATGTCCGTGGAT | AAACATCCACGGACATTACGTGCTC |
| IRF3 | CACCGAGCTGACACTCACCTTCCCC | AAACGGGGAAGGTGAGTGTCAGCTC |
| CTRL | CACCGACGGAGGCTAAGCGTCGCAA | AAACTTGCGACGCTTAGCCTCCGTC |
| STING | CACCGCATATTACATCGGATATCTG | AAACCAGATATCCGATGTAATATGC |
| cGAS | CACCGGAACTTTCCCGCCTTAGGCA | AAACTGCCTAAGGCGGGAAAGTTCC |
| CTRL  (for GL261) | GTGTAGTTCGACCATTCGTG | CACGAATGGTCGAACTACAC |
| cGAS  (for GL261) | CCTTACGACTTTCCGCGCCT | AGGCGCGGAAAGTCGTAAGG |
| **siRNA** |  | |
| si*DNA-PKcs1* | GAUCGCACCUUACUCUGUUUU | |
| si*DNA-PKcs2* | CUUUAUGGUGGCCAUGGAGUU | |
| si*DNA-PKcs*3 | Cat # J-005030-06-0010 on-TARGET plus | |
| si*KU701* | ACAAGCAGUGGACCUGACUU | |
| si*KU702* | GUCAGGGUGGGAGUCAUAUUAUU | |
| si*KU80* | GGAUGGAGUUACUCUGAUUUU | |
| **DNA probes** |  | |
| Fwd | ACATCTAGTACATGTCTAGTCAGTATCTAGTGATTATCTAGACATACATGATCTATGACATATATAGTGGATAAGTGTGG | |
| Rev | CCACACTTATCCACTATATATGTCATAGATCATGTATGTCTAGATAATCACTAGATACTGACTAGACATGTACTAGATGT | |
