# Supplementary figures and images for: DNA damage repair kinase DNA‐PK and cGAS synergize to induce cancer‐related inflammation in glioblastoma (part 1 of 2)
Source: EMBO J. 2022 Dec 27;42(7):e111961. doi: 10.15252/embj.2022111961 (PMC10068334; doi:10.15252/embj.2022111961)

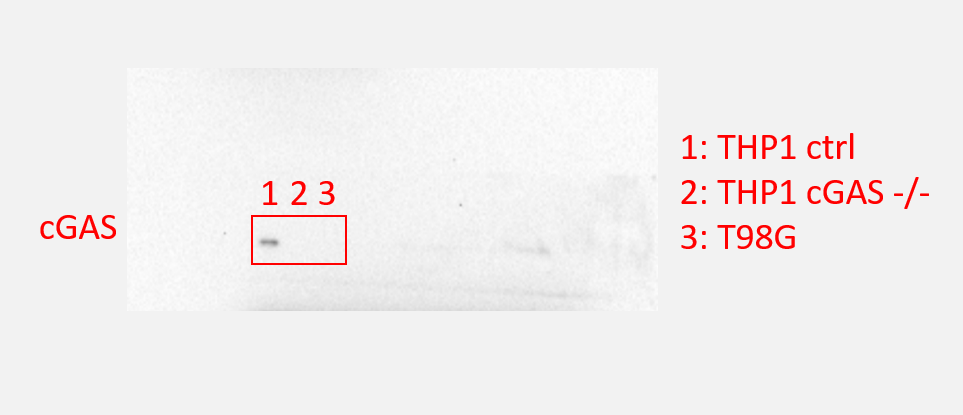

Supplement: Supplementary file 3 — Source Data for Expanded View [file EMBJ-42-e111961-s004.zip › EV Figures/EV Figure 1/EV Figure 1A/Ev Fig 1A_western_cGAS.tif]

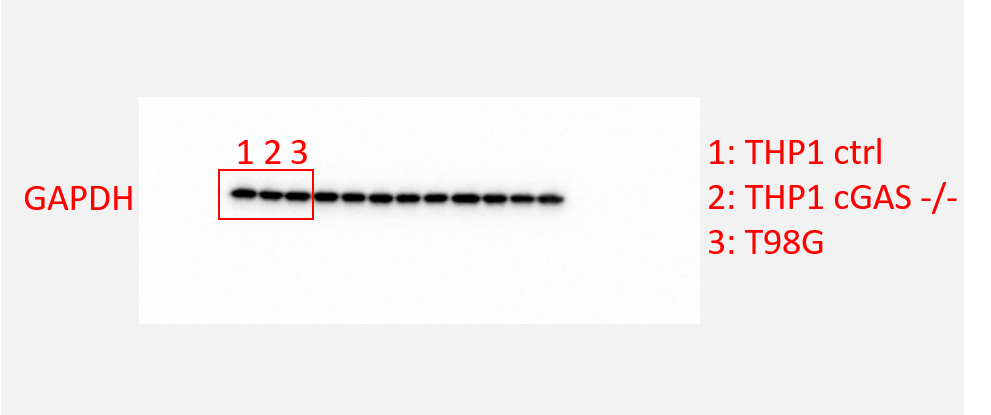

Supplement: Supplementary file 3 — Source Data for Expanded View [file EMBJ-42-e111961-s004.zip › EV Figures/EV Figure 1/EV Figure 1A/Ev Fig 1A_western_GAPDH.tif]

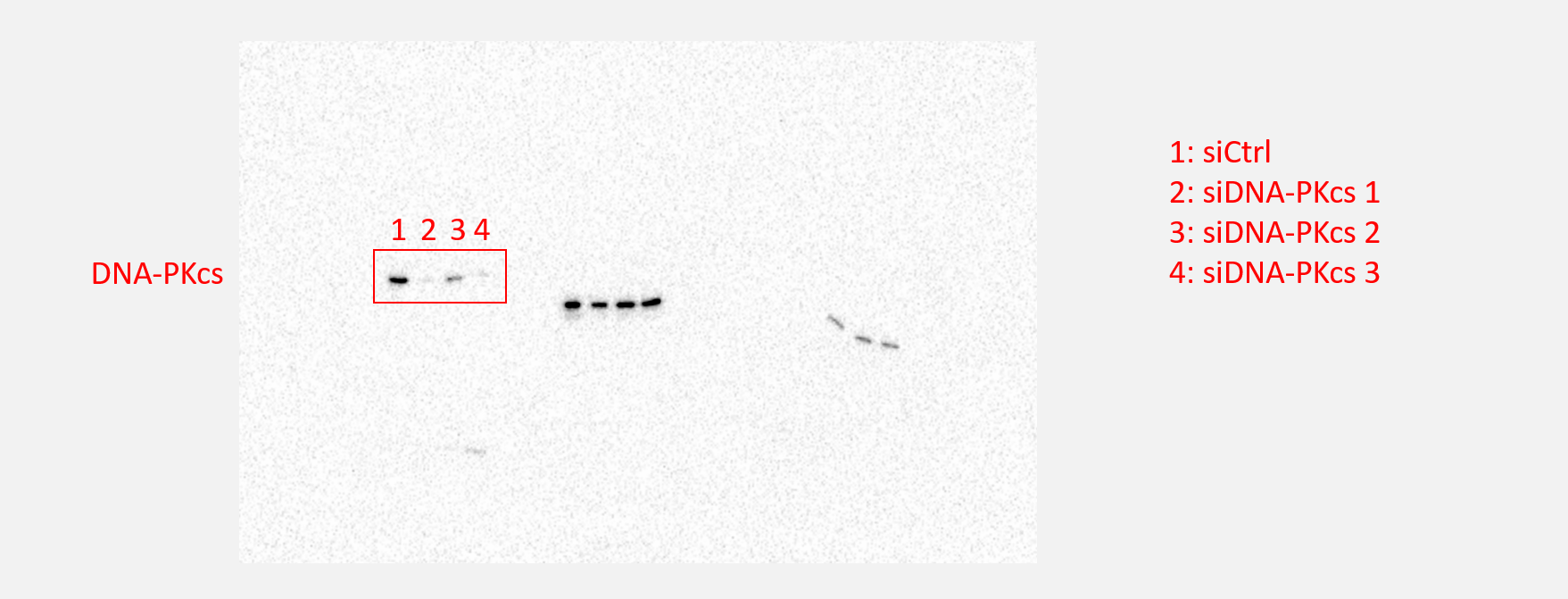

Supplement: Supplementary file 3 — Source Data for Expanded View [file EMBJ-42-e111961-s004.zip › EV Figures/EV Figure 1/EV Figure 1I/EV Figure 1I_western_DNA-PKcs.tif]

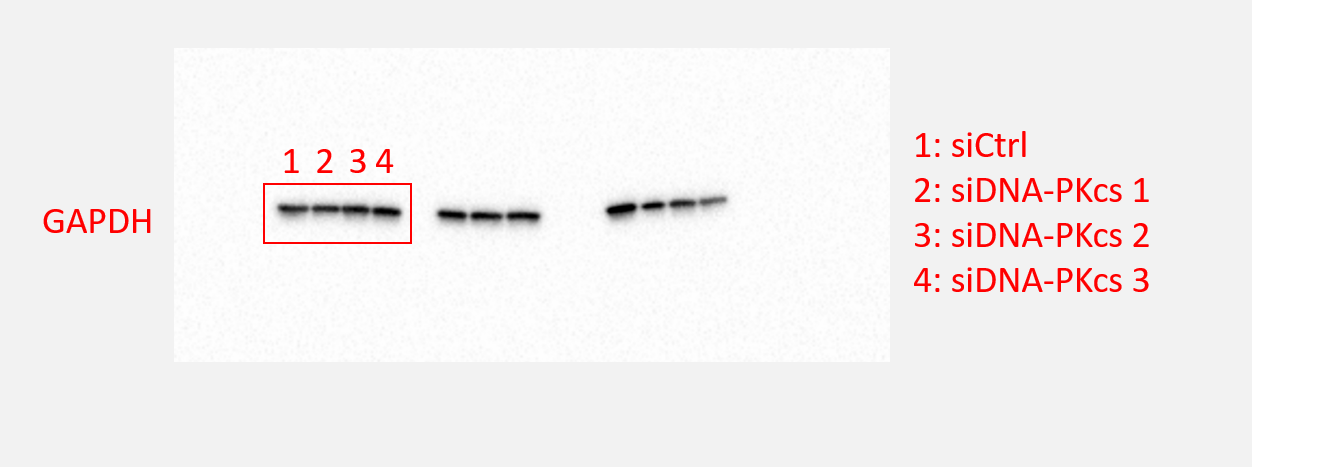

Supplement: Supplementary file 3 — Source Data for Expanded View [file EMBJ-42-e111961-s004.zip › EV Figures/EV Figure 1/EV Figure 1I/EV Figure 1I_western_GAPDH.tif]

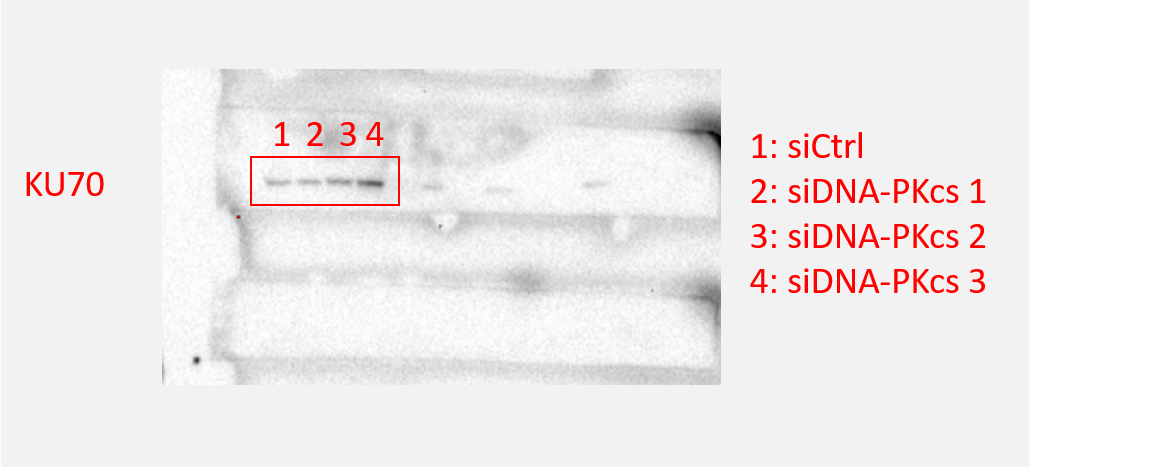

Supplement: Supplementary file 3 — Source Data for Expanded View [file EMBJ-42-e111961-s004.zip › EV Figures/EV Figure 1/EV Figure 1I/EV Figure 1I_western_KU70.tif]

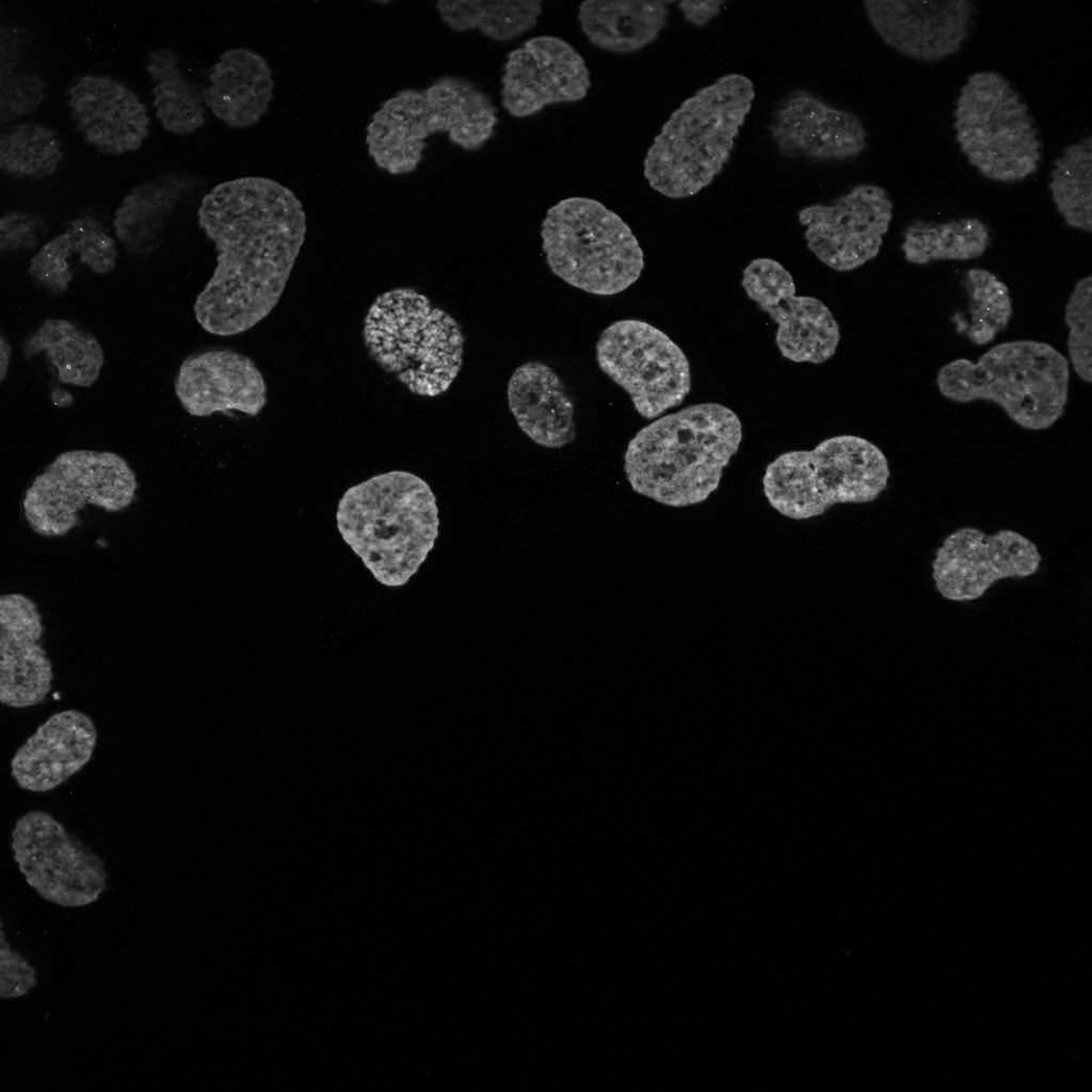

Supplement: Supplementary file 3 — Source Data for Expanded View [file EMBJ-42-e111961-s004.zip › EV Figures/EV Figure 1/EV Figure IK/EV Fig 1K_IF_53BP1_dmso dmso.tif]

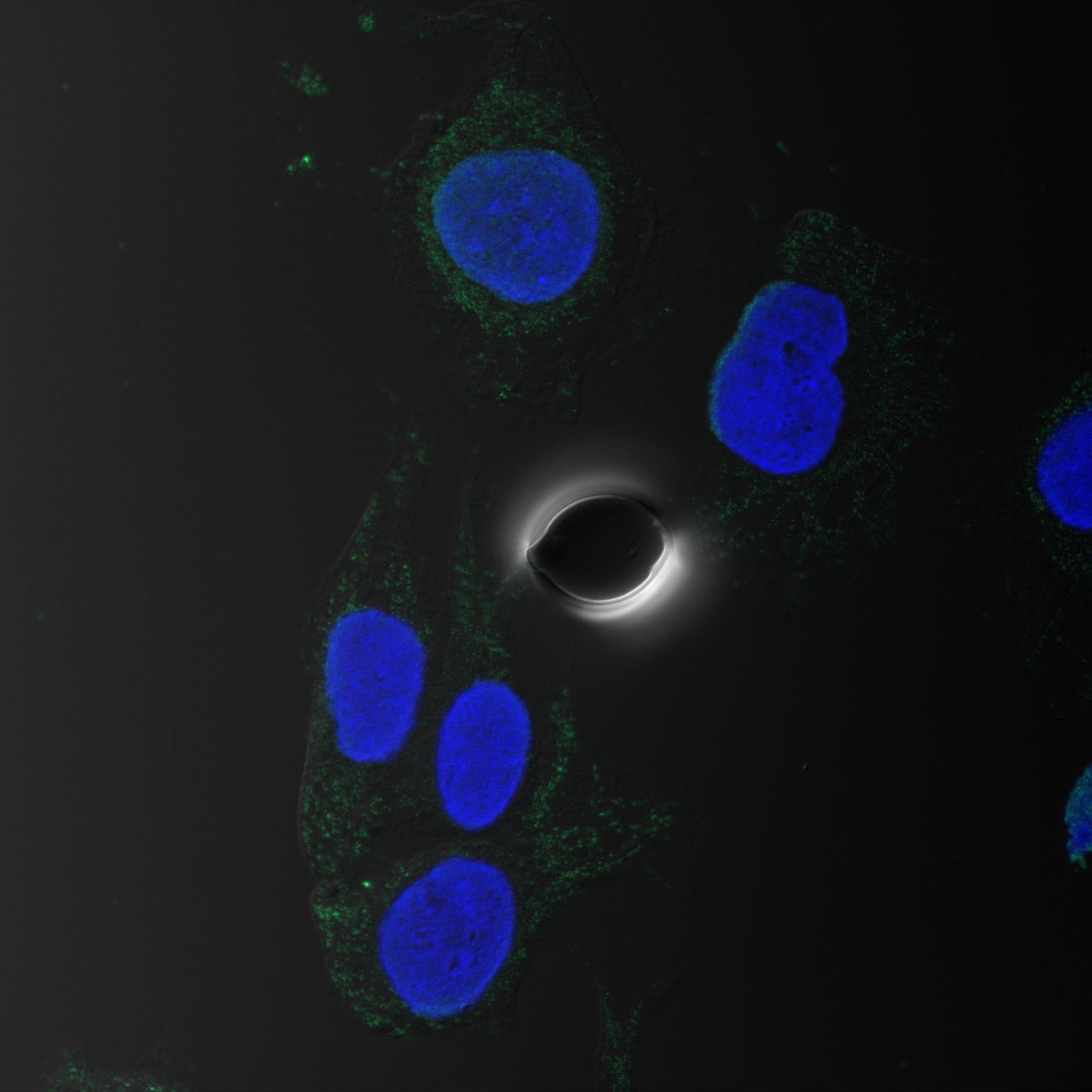

Supplement: Supplementary file 3 — Source Data for Expanded View [file EMBJ-42-e111961-s004.zip › EV Figures/EV Figure 1/EV Figure IK/EV Fig 1K_IF_dsDNA_CPTNU7441.czi.tif]

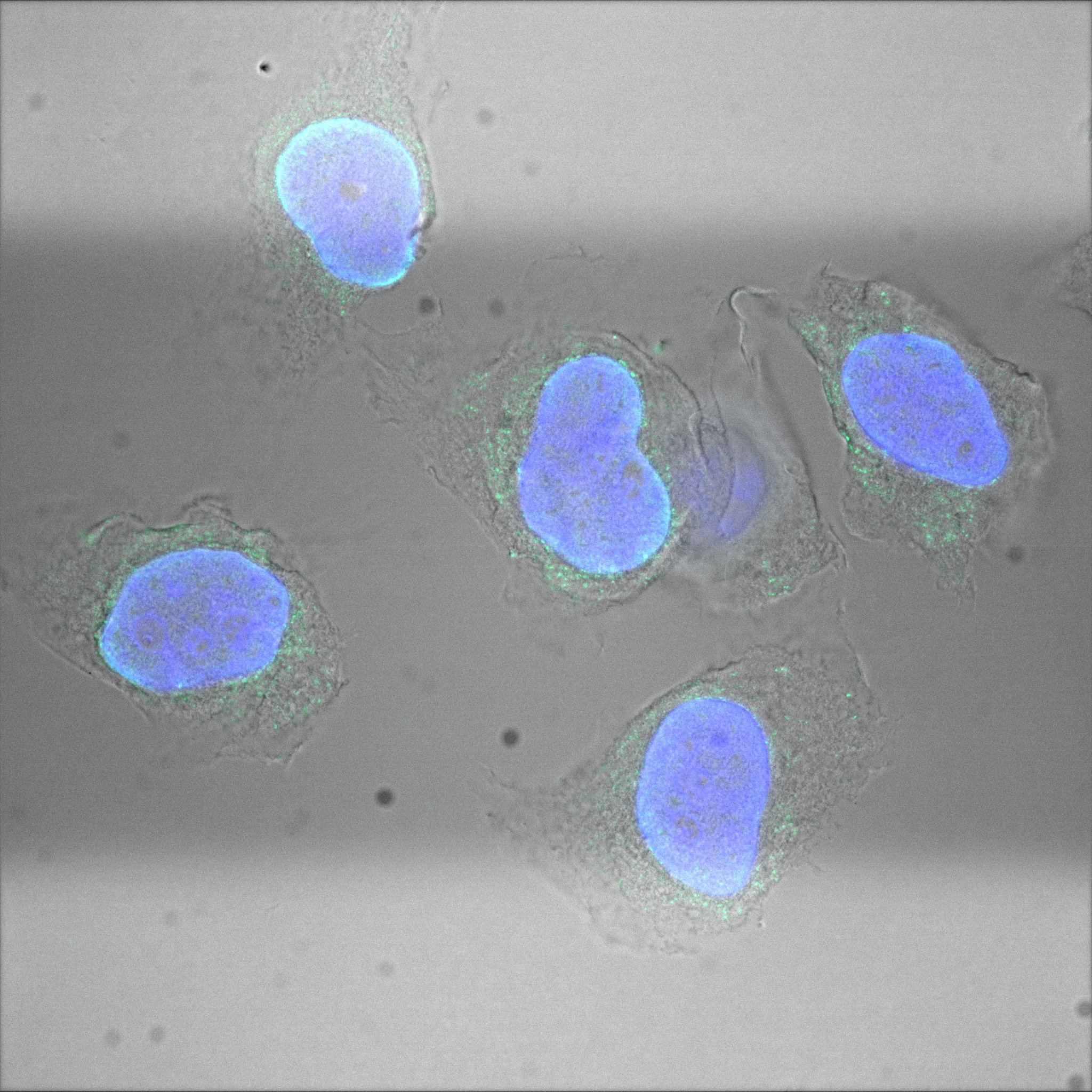

Supplement: Supplementary file 3 — Source Data for Expanded View [file EMBJ-42-e111961-s004.zip › EV Figures/EV Figure 1/EV Figure IK/EV Fig 1K_IF_dsDNA_CPTDmso.czi.tif]

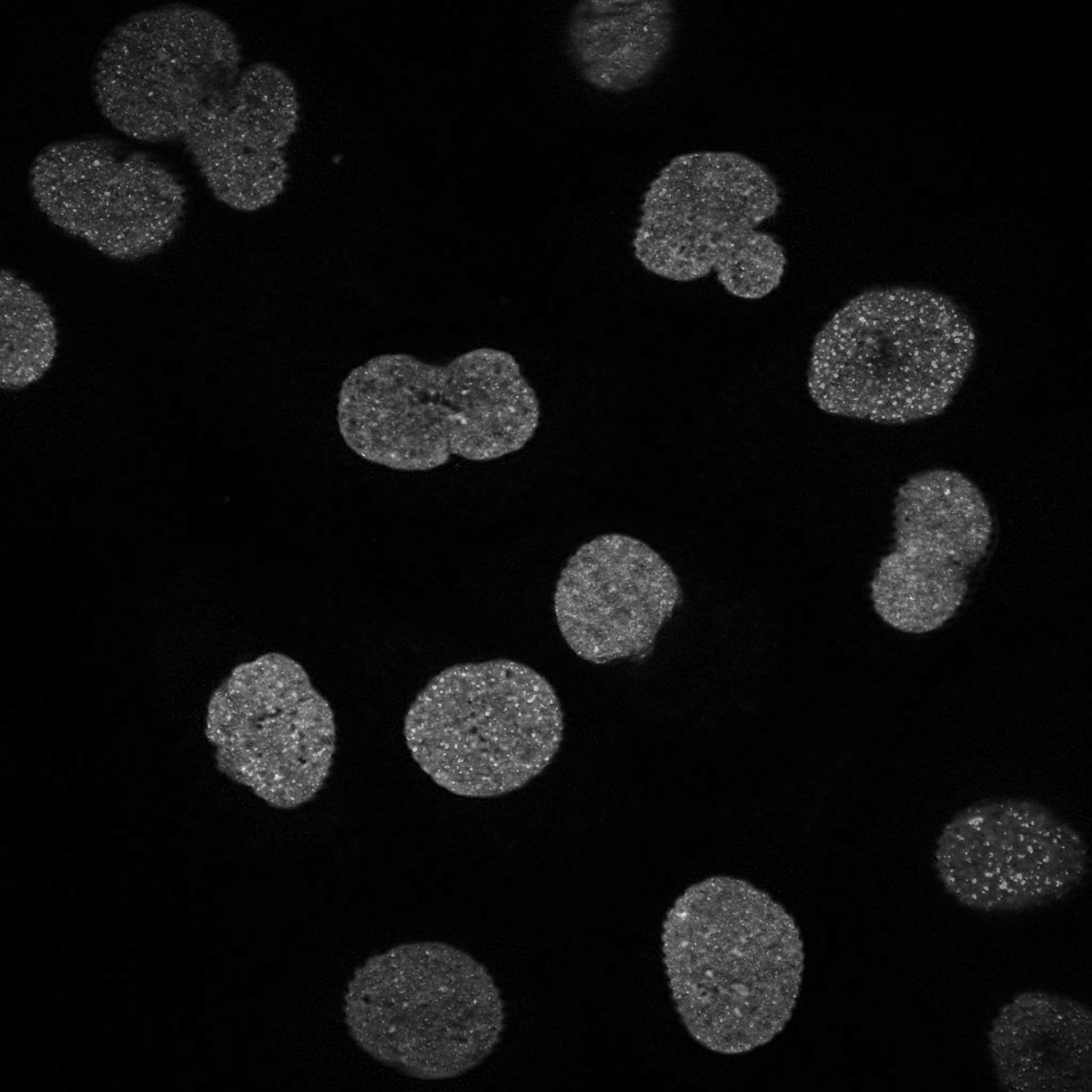

Supplement: Supplementary file 3 — Source Data for Expanded View [file EMBJ-42-e111961-s004.zip › EV Figures/EV Figure 1/EV Figure IK/EV Fig 1K_IF_53BP1_CPT NU7441.tif]

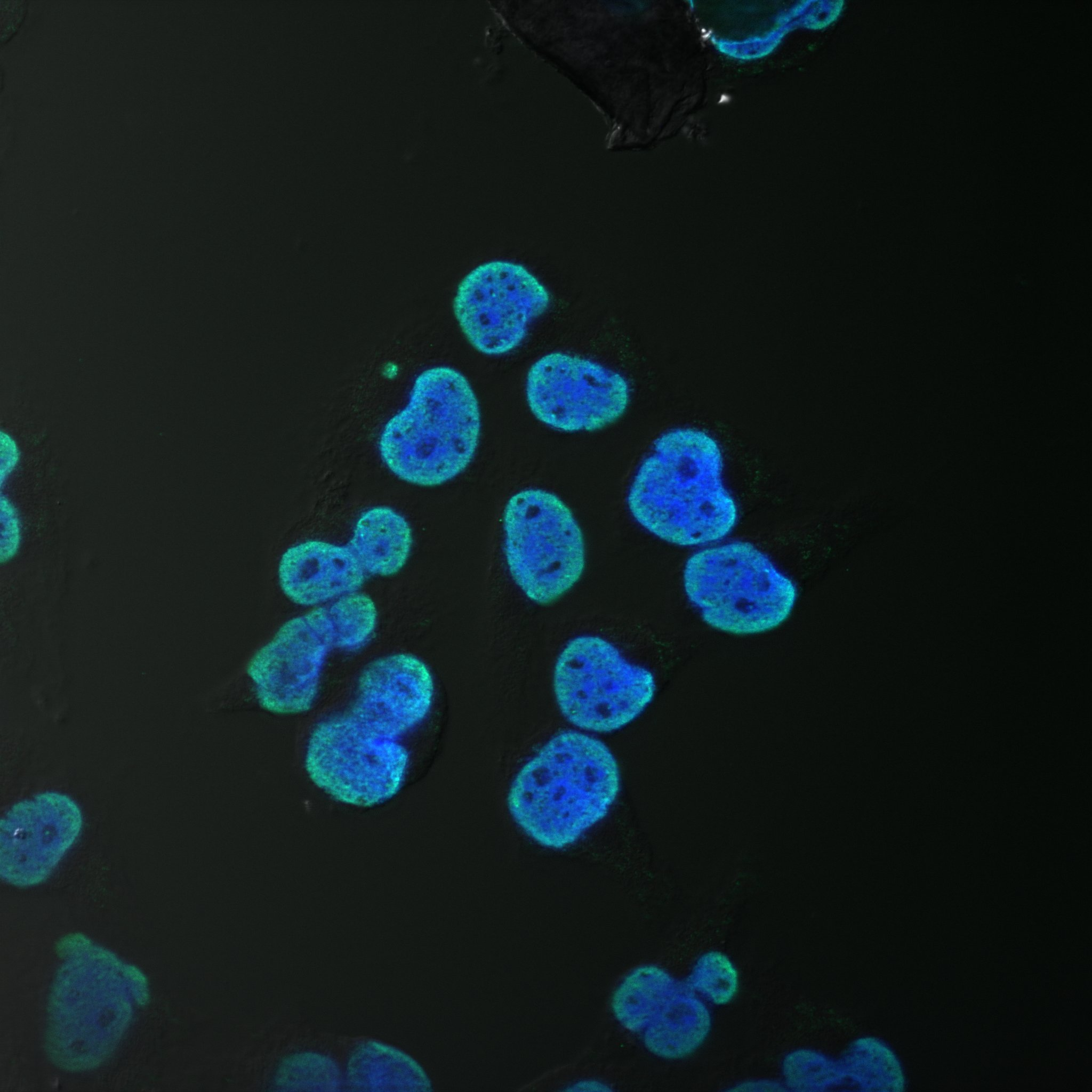

Supplement: Supplementary file 3 — Source Data for Expanded View [file EMBJ-42-e111961-s004.zip › EV Figures/EV Figure 1/EV Figure IK/EV Fig 1K_IF_dsDNA_DmsoDmso.czi.tif]

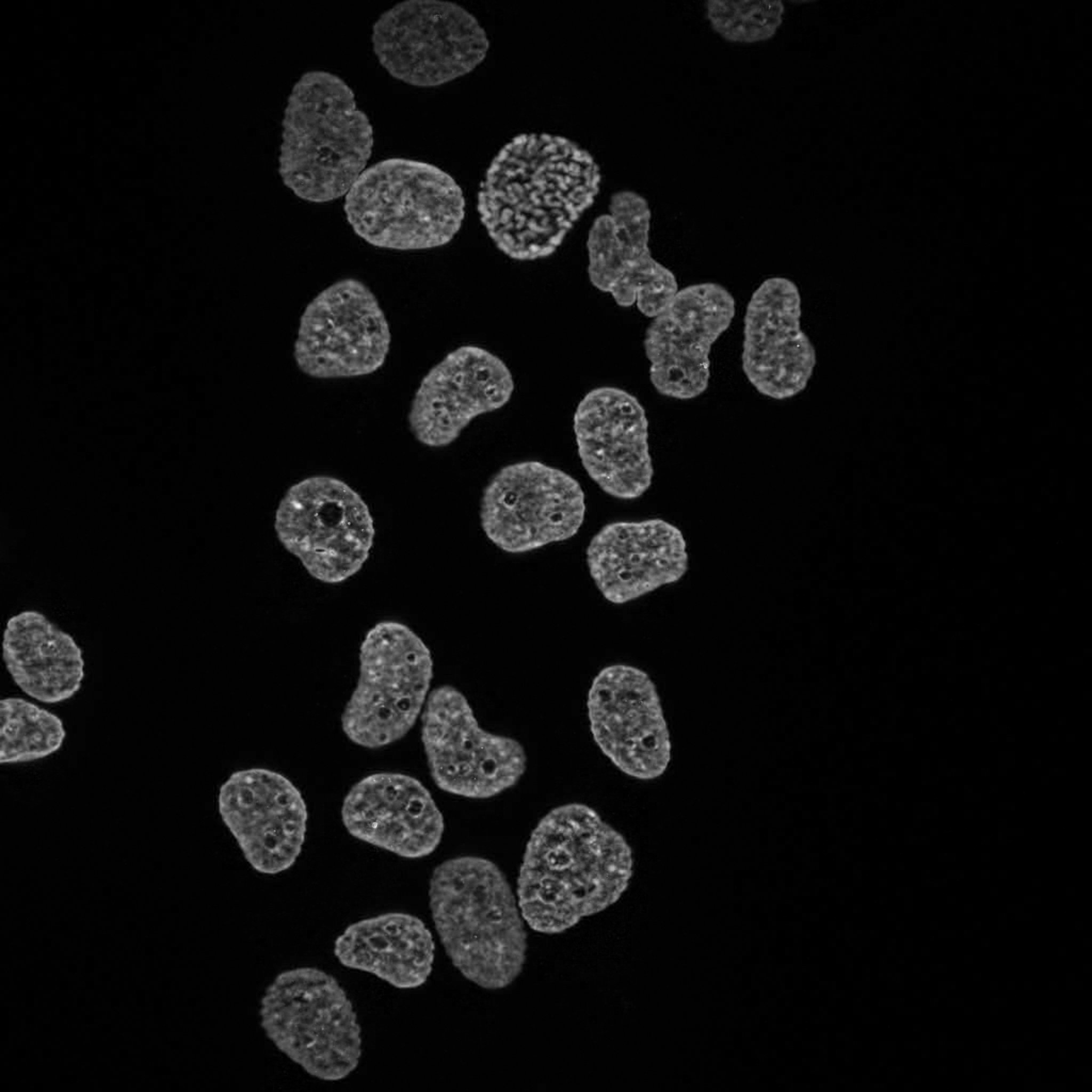

Supplement: Supplementary file 3 — Source Data for Expanded View [file EMBJ-42-e111961-s004.zip › EV Figures/EV Figure 1/EV Figure IK/EV Fig 1K_IF_53BP1_dmso NU7441.tif]

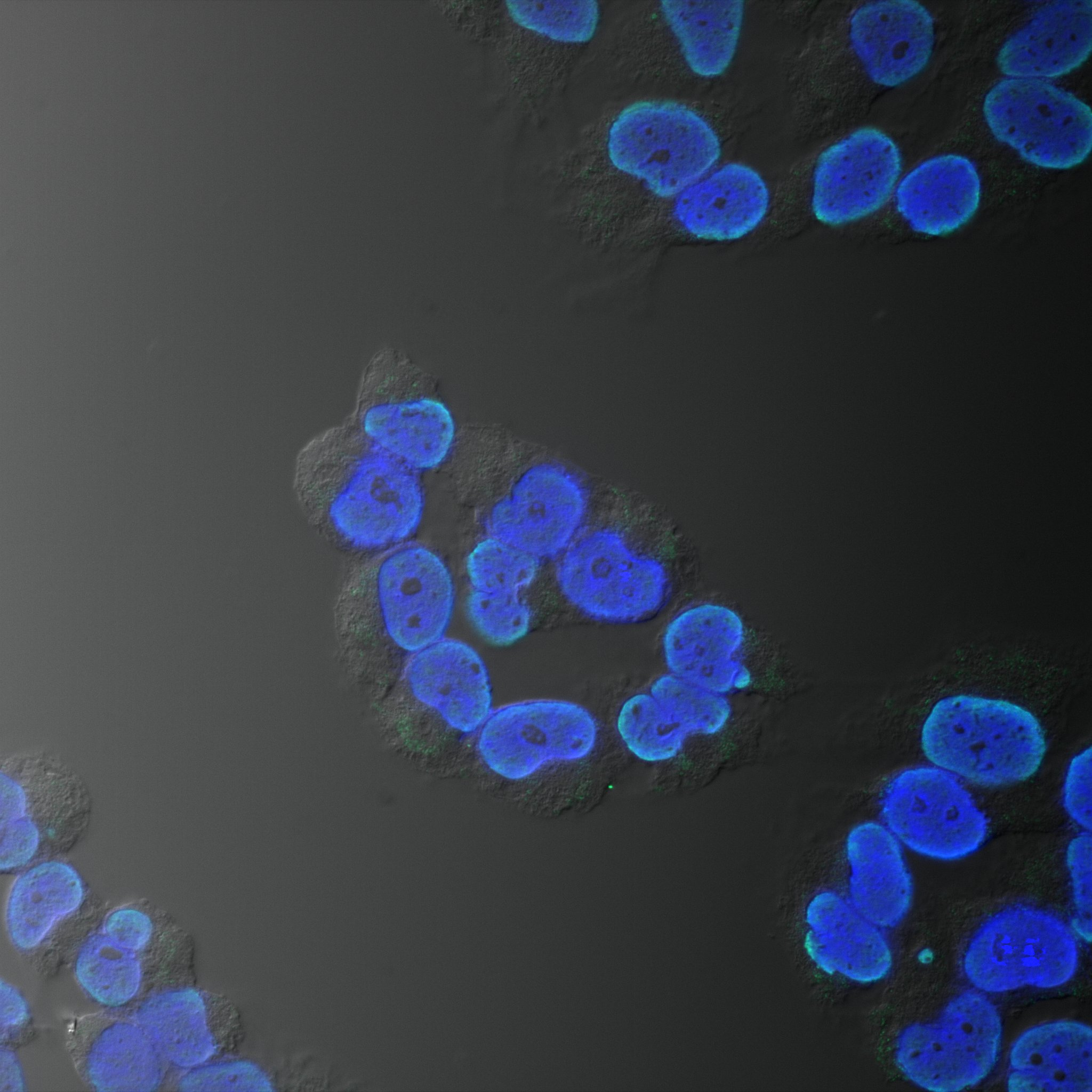

Supplement: Supplementary file 3 — Source Data for Expanded View [file EMBJ-42-e111961-s004.zip › EV Figures/EV Figure 1/EV Figure IK/EV Fig 1K_IF_dsDNA_DmsoNU7441.czi.tif]

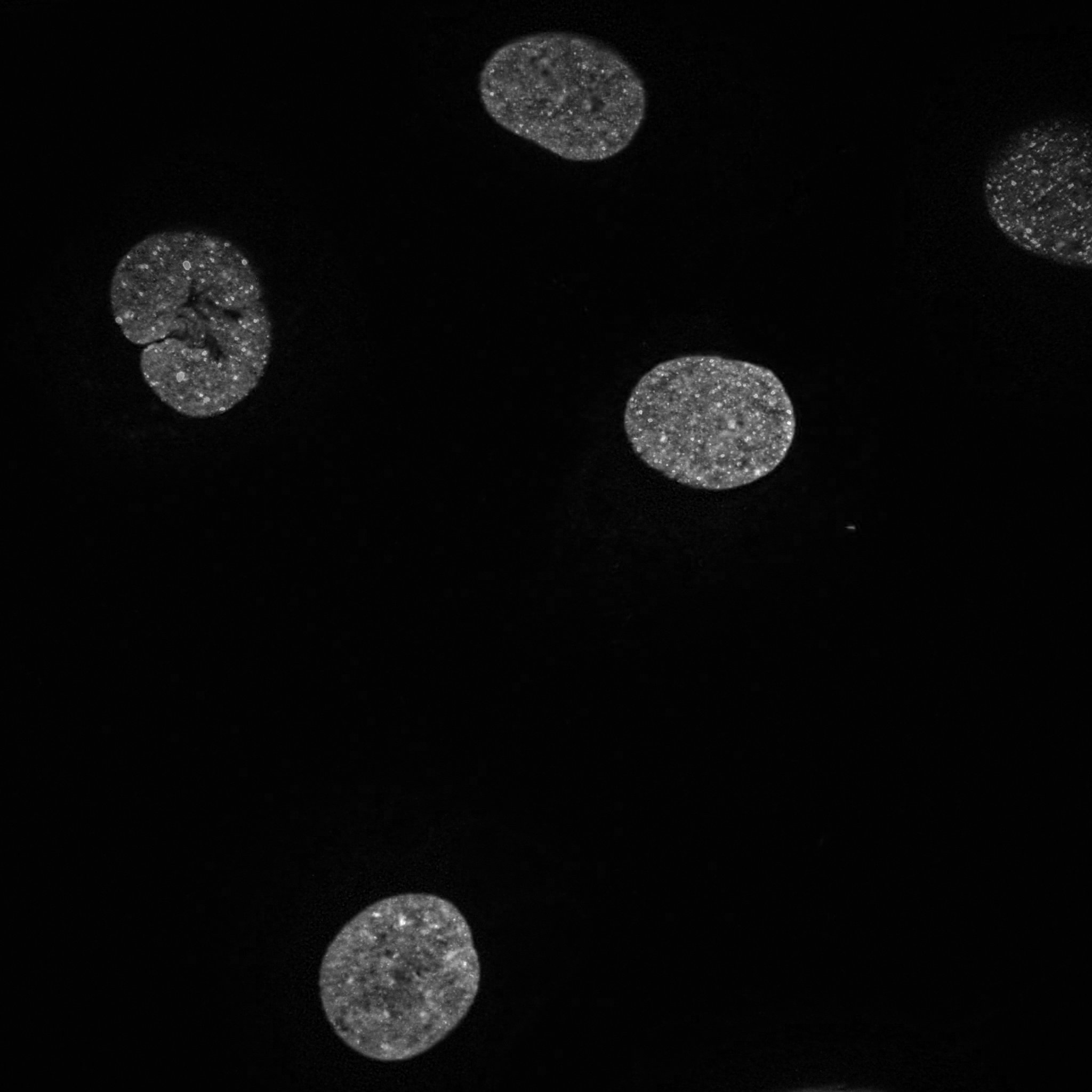

Supplement: Supplementary file 3 — Source Data for Expanded View [file EMBJ-42-e111961-s004.zip › EV Figures/EV Figure 1/EV Figure IK/EV Fig 1K_IF_53BP1_CPT dmso.tif]

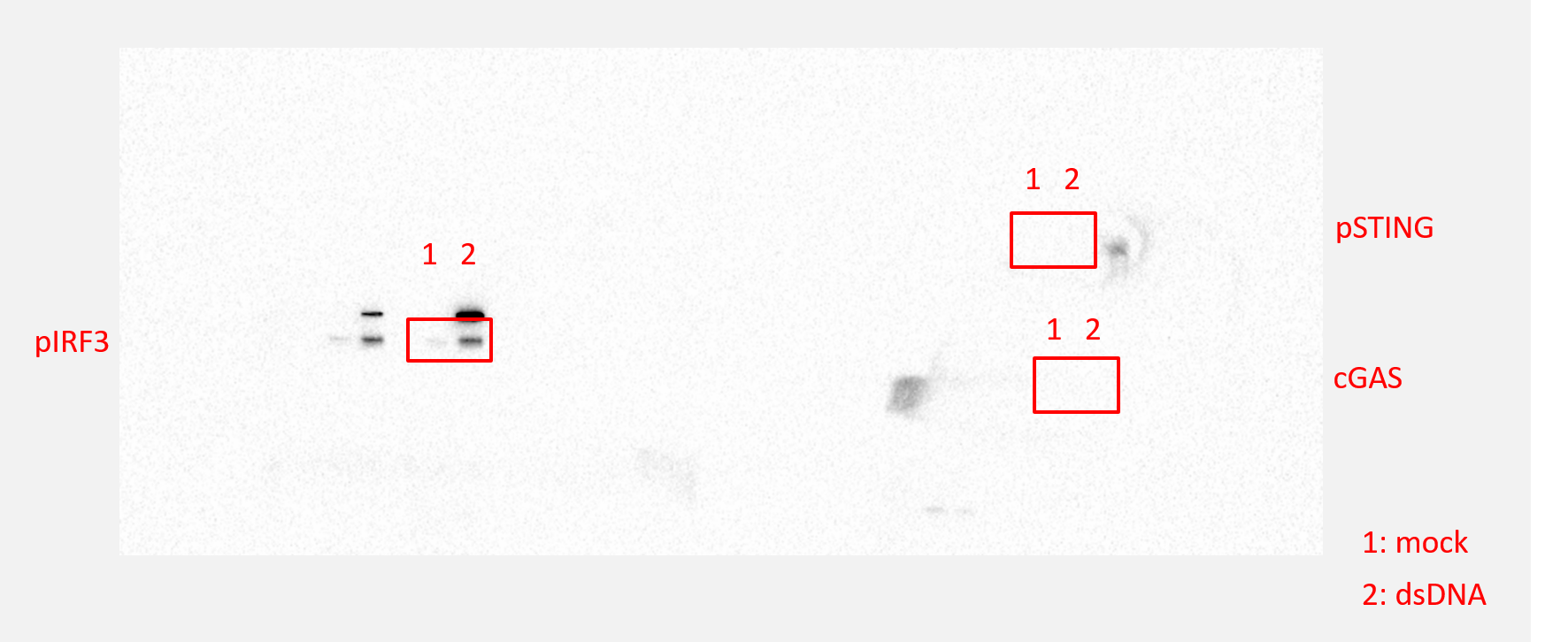

Supplement: Supplementary file 3 — Source Data for Expanded View [file EMBJ-42-e111961-s004.zip › EV Figures/EV Figure 1/EV Figure 1B/Ev Fig 1B_western_pSTING, pIRF3 & cGAS.tif]

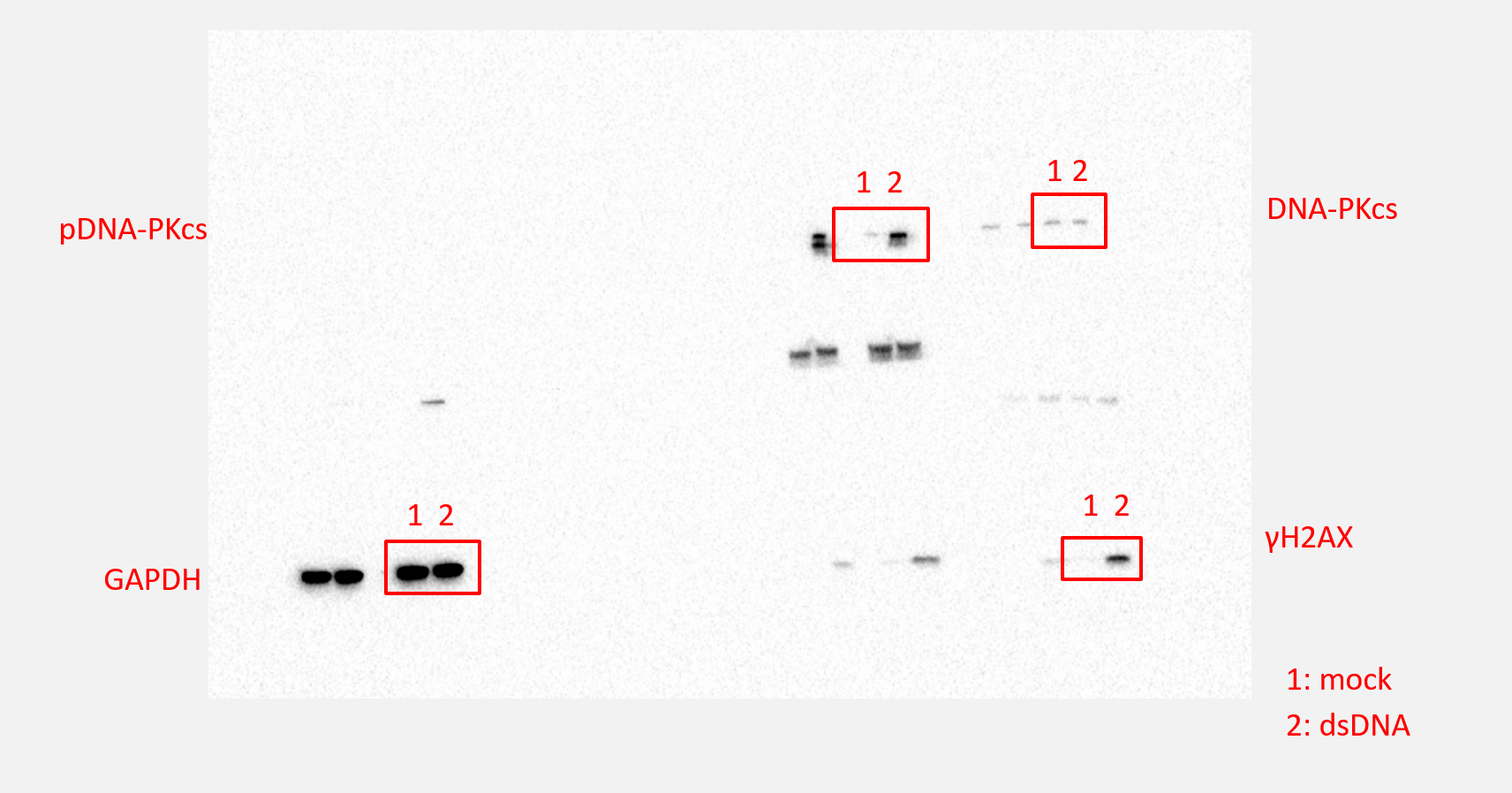

Supplement: Supplementary file 3 — Source Data for Expanded View [file EMBJ-42-e111961-s004.zip › EV Figures/EV Figure 1/EV Figure 1B/Ev Fig 1B_western_GAPDH, pDNA-PKcs, DNA-PKcs &gH2AX.tif]

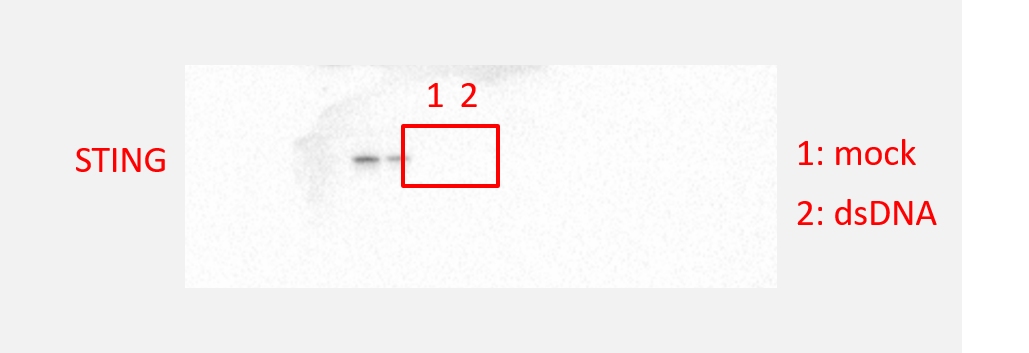

Supplement: Supplementary file 3 — Source Data for Expanded View [file EMBJ-42-e111961-s004.zip › EV Figures/EV Figure 1/EV Figure 1B/Ev Fig 1B_western_STING.tif]

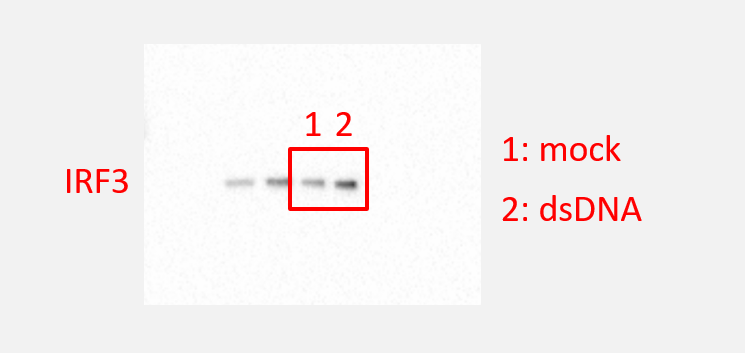

Supplement: Supplementary file 3 — Source Data for Expanded View [file EMBJ-42-e111961-s004.zip › EV Figures/EV Figure 1/EV Figure 1B/Ev Fig 1B_western_IRF3.tif]

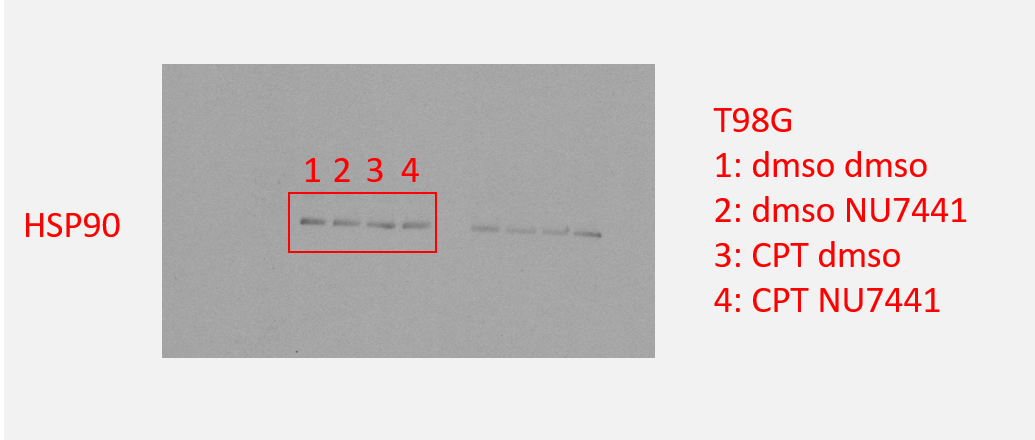

Supplement: Supplementary file 3 — Source Data for Expanded View [file EMBJ-42-e111961-s004.zip › EV Figures/EV Figure 1/EV Figure 1J/Ev Fig 1J_western_HSP90.tif]

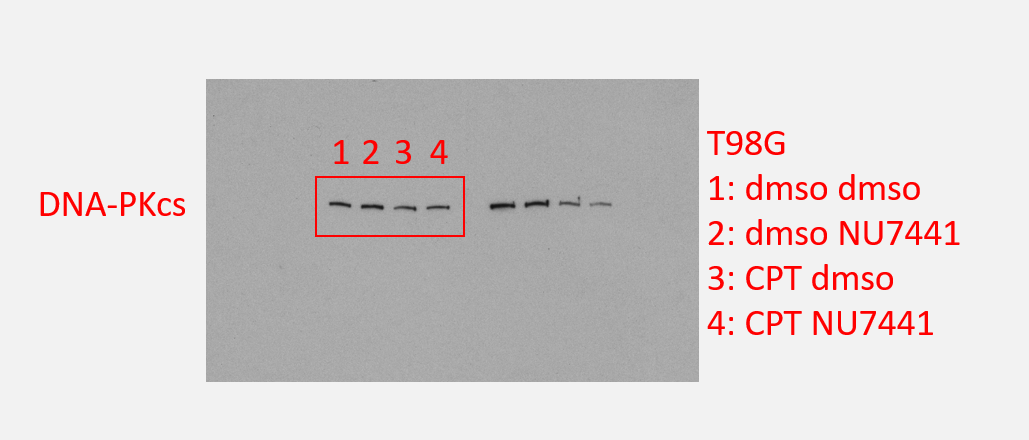

Supplement: Supplementary file 3 — Source Data for Expanded View [file EMBJ-42-e111961-s004.zip › EV Figures/EV Figure 1/EV Figure 1J/Ev Fig 1J_western_DNA-PKcs.tif]

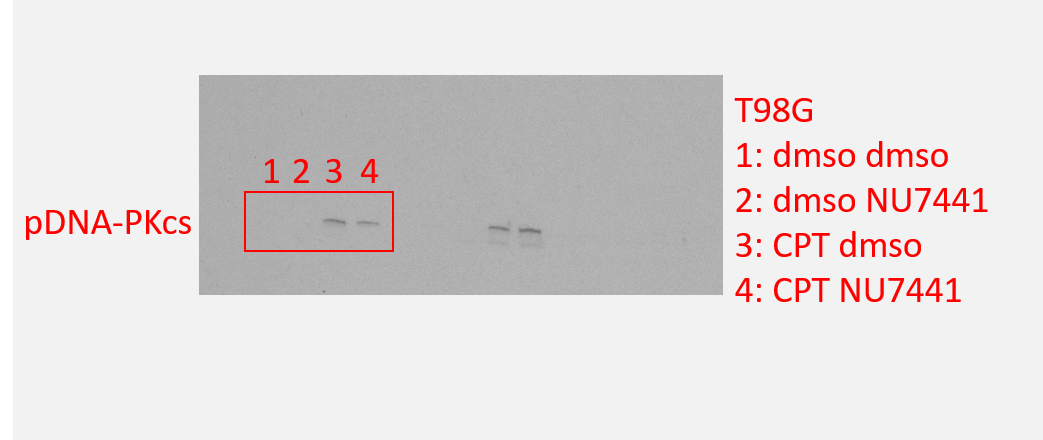

Supplement: Supplementary file 3 — Source Data for Expanded View [file EMBJ-42-e111961-s004.zip › EV Figures/EV Figure 1/EV Figure 1J/Ev Fig 1J_western_pDNA-PKcs.tif]

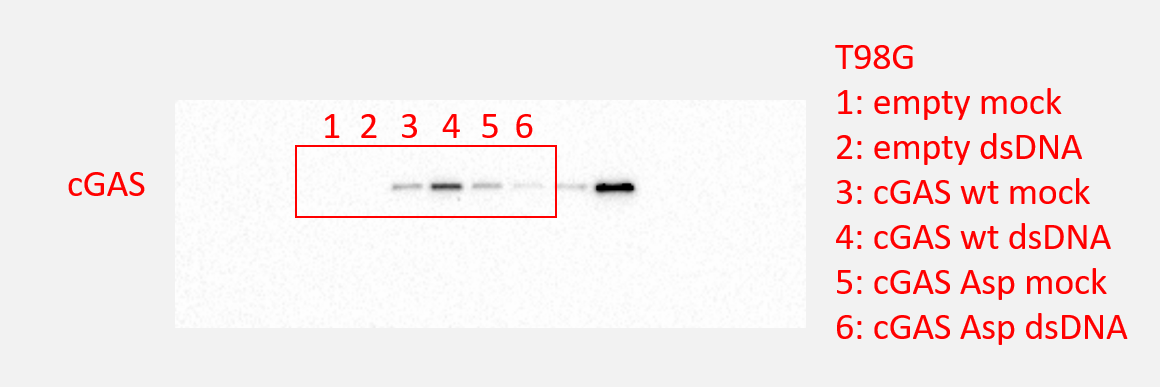

Supplement: Supplementary file 3 — Source Data for Expanded View [file EMBJ-42-e111961-s004.zip › EV Figures/EV Figure 3/EV Figure 3L/EV Fig 3L_western_cGAS.tif]

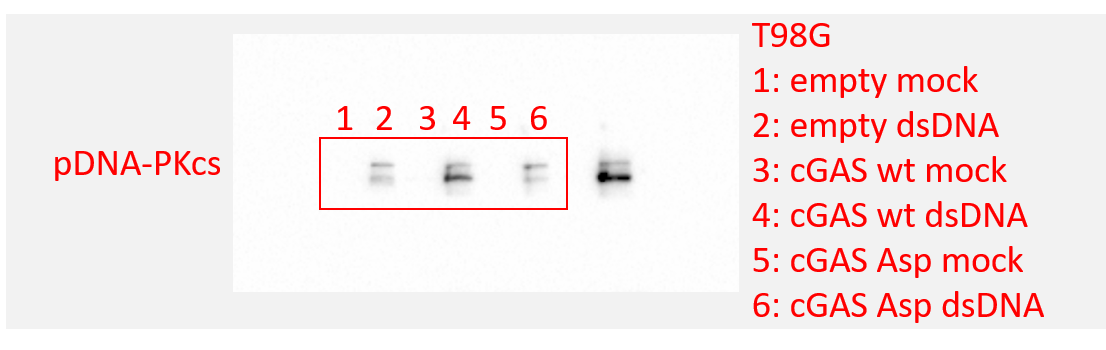

Supplement: Supplementary file 3 — Source Data for Expanded View [file EMBJ-42-e111961-s004.zip › EV Figures/EV Figure 3/EV Figure 3L/EV Fig 3L_western_pDNA-PKcs.tif]

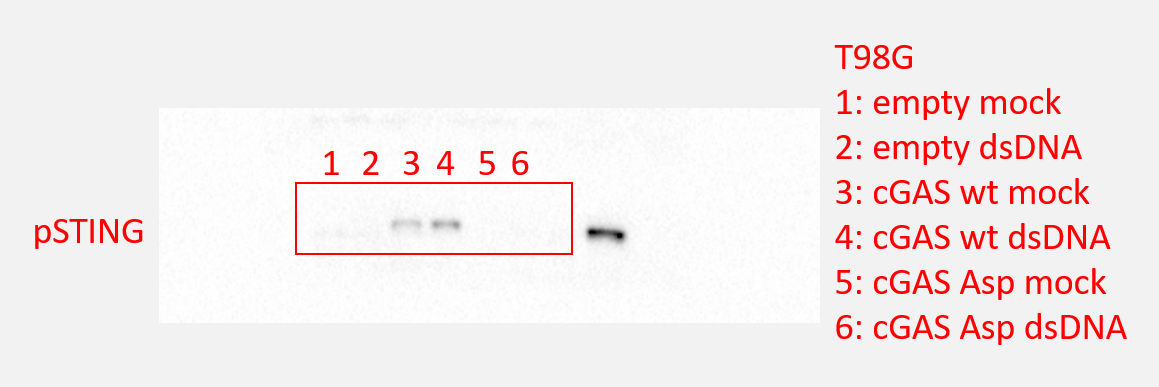

Supplement: Supplementary file 3 — Source Data for Expanded View [file EMBJ-42-e111961-s004.zip › EV Figures/EV Figure 3/EV Figure 3L/EV Fig 3L_western_pSTING.tif]

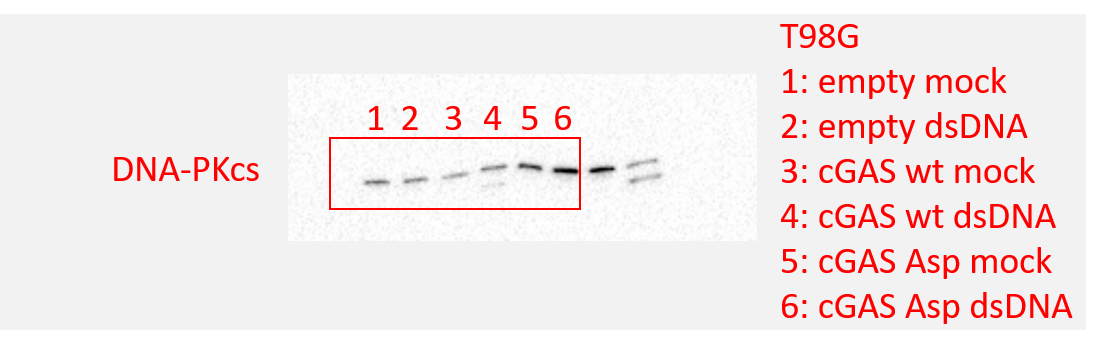

Supplement: Supplementary file 3 — Source Data for Expanded View [file EMBJ-42-e111961-s004.zip › EV Figures/EV Figure 3/EV Figure 3L/EV Fig 3L_western_DNA-PKcs.tif]

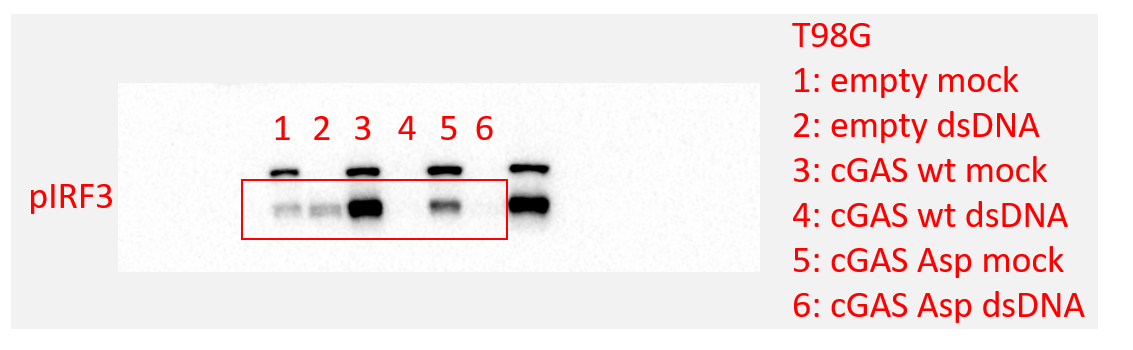

Supplement: Supplementary file 3 — Source Data for Expanded View [file EMBJ-42-e111961-s004.zip › EV Figures/EV Figure 3/EV Figure 3L/EV Fig 3L_western_pIRF3.tif]

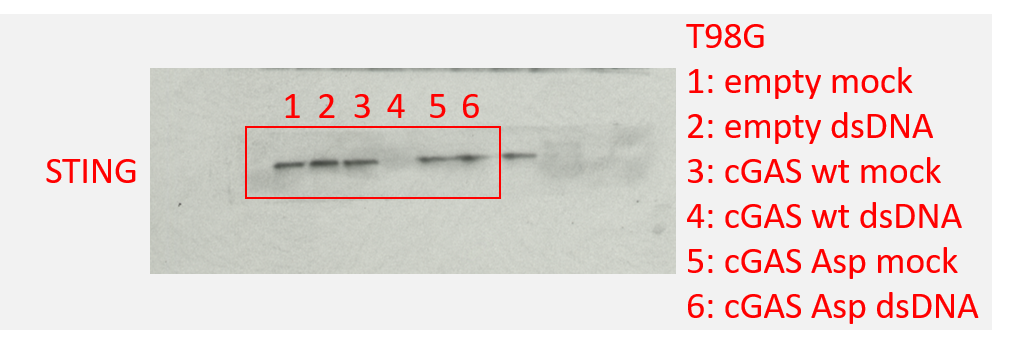

Supplement: Supplementary file 3 — Source Data for Expanded View [file EMBJ-42-e111961-s004.zip › EV Figures/EV Figure 3/EV Figure 3L/EV Fig 3L_western_STING.tif]

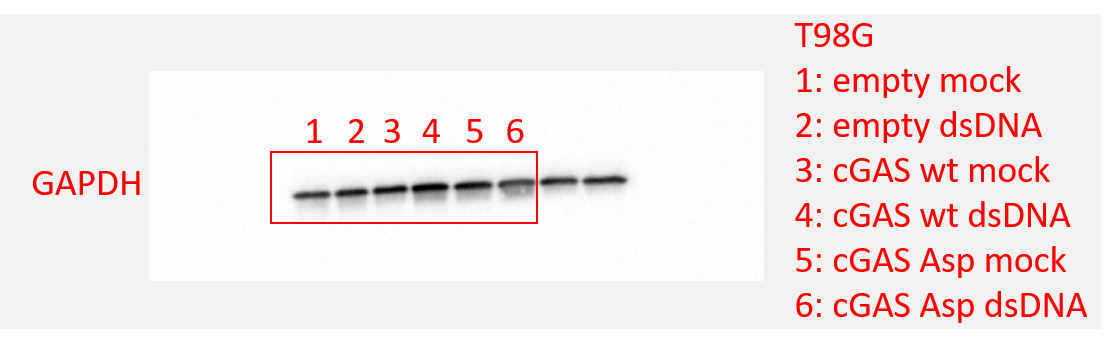

Supplement: Supplementary file 3 — Source Data for Expanded View [file EMBJ-42-e111961-s004.zip › EV Figures/EV Figure 3/EV Figure 3L/EV Fig 3L_western_GAPDH.tif]

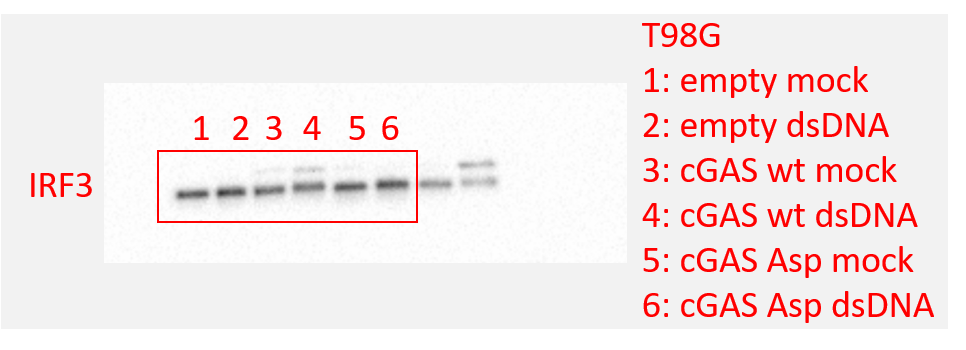

Supplement: Supplementary file 3 — Source Data for Expanded View [file EMBJ-42-e111961-s004.zip › EV Figures/EV Figure 3/EV Figure 3L/EV Fig 3L_western_IRF3.tif]

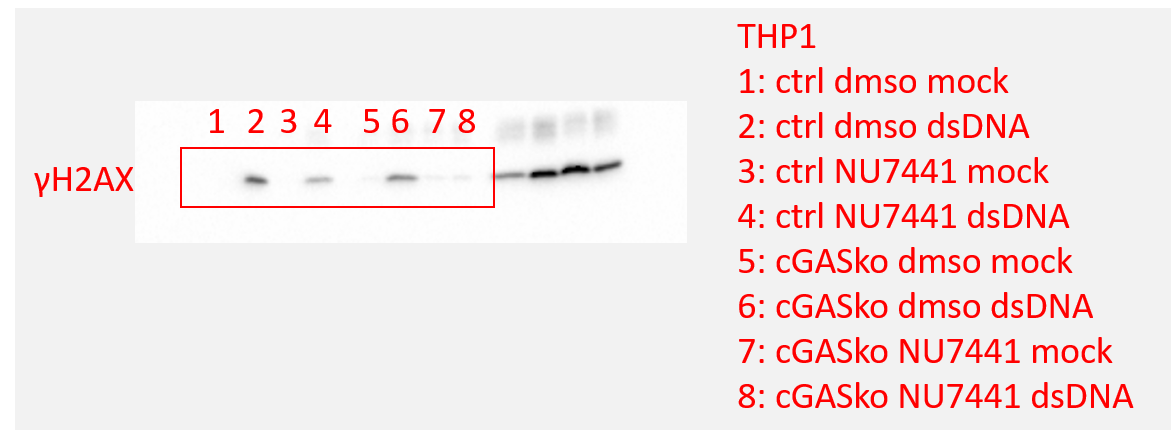

Supplement: Supplementary file 3 — Source Data for Expanded View [file EMBJ-42-e111961-s004.zip › EV Figures/EV Figure 3/EV Figure 3E/EV Fig3E_western_gH2AX.tif]

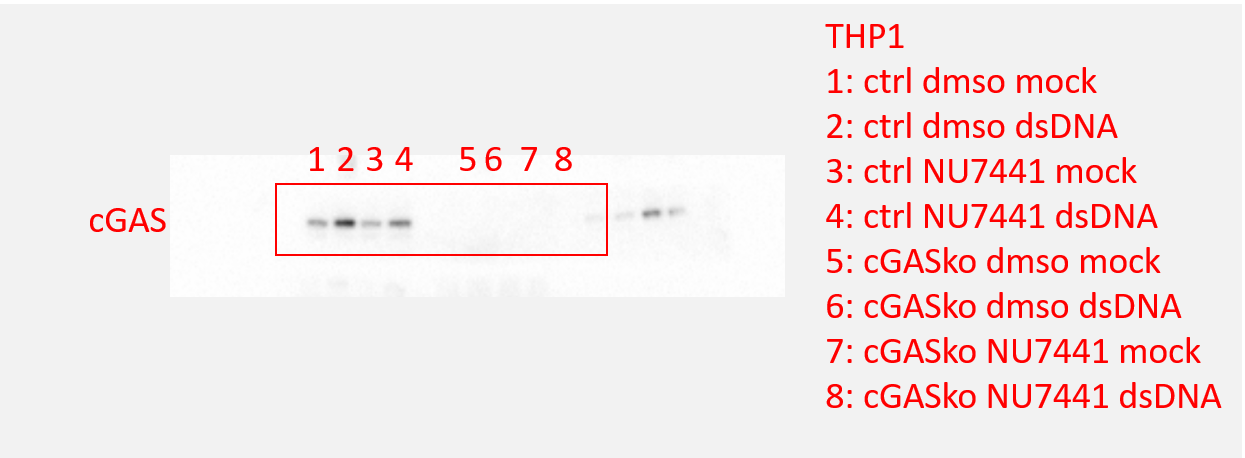

Supplement: Supplementary file 3 — Source Data for Expanded View [file EMBJ-42-e111961-s004.zip › EV Figures/EV Figure 3/EV Figure 3E/EV Fig3E_western_cGAS.tif]

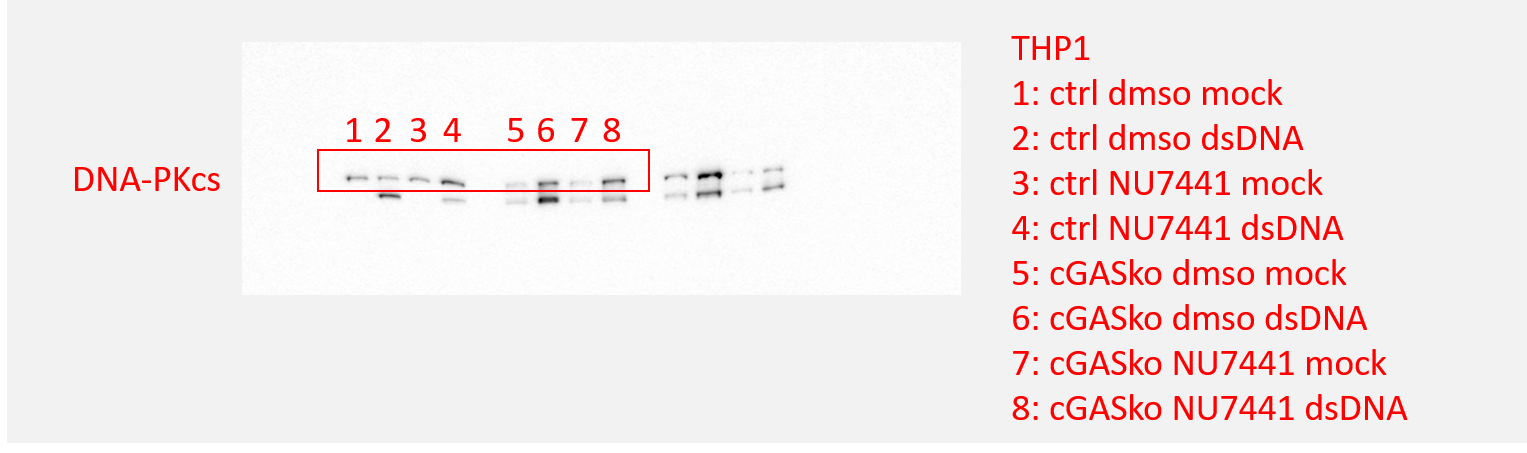

Supplement: Supplementary file 3 — Source Data for Expanded View [file EMBJ-42-e111961-s004.zip › EV Figures/EV Figure 3/EV Figure 3E/EV Fig3E_western_DNA-PKcs.tif]

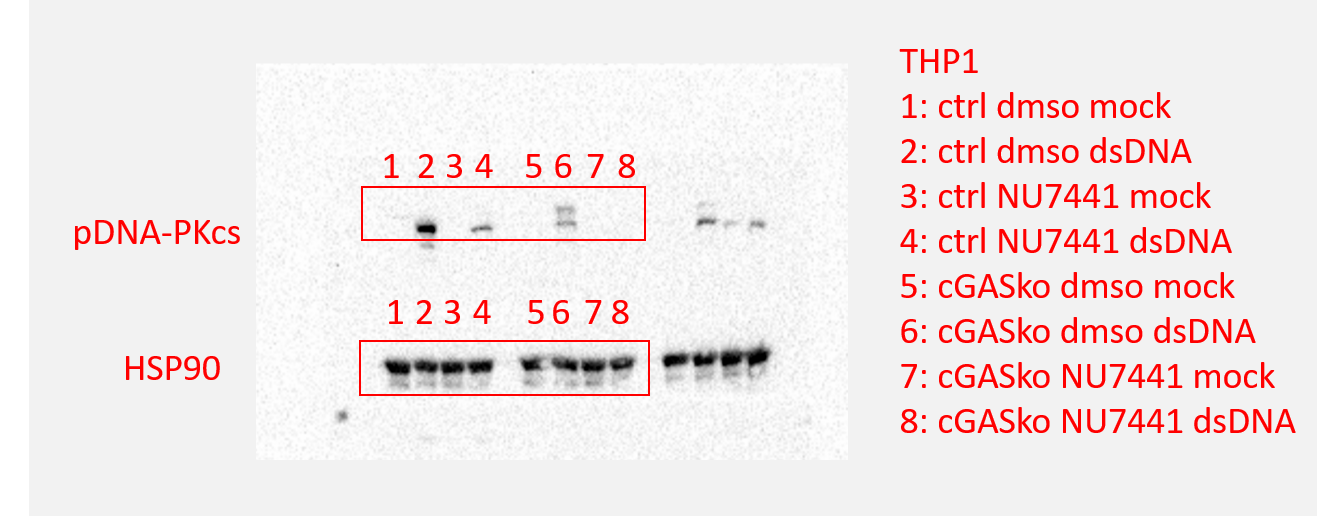

Supplement: Supplementary file 3 — Source Data for Expanded View [file EMBJ-42-e111961-s004.zip › EV Figures/EV Figure 3/EV Figure 3E/EVFig3E_western_pDNA-PKcs & HSP90.tif]

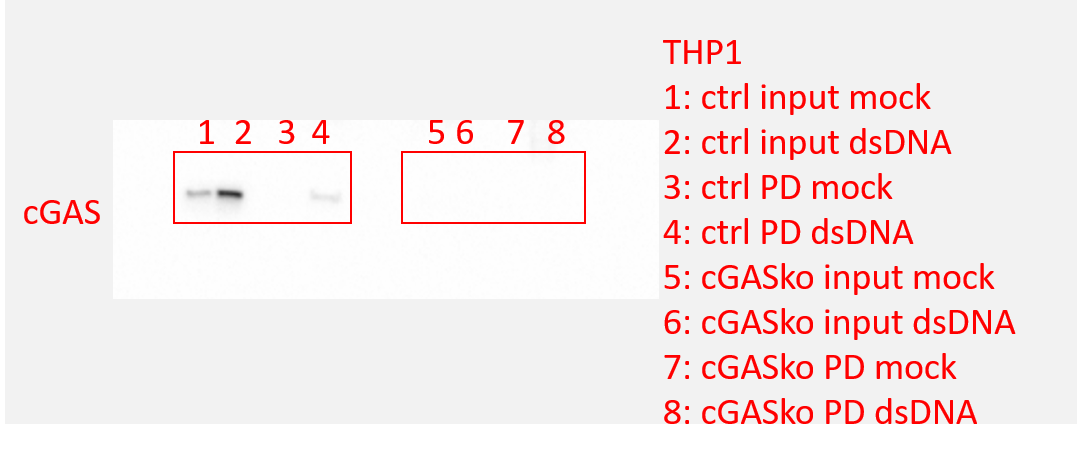

Supplement: Supplementary file 3 — Source Data for Expanded View [file EMBJ-42-e111961-s004.zip › EV Figures/EV Figure 3/EV Figure 3B/Ev Fig 3B_western_cGAS.tif]

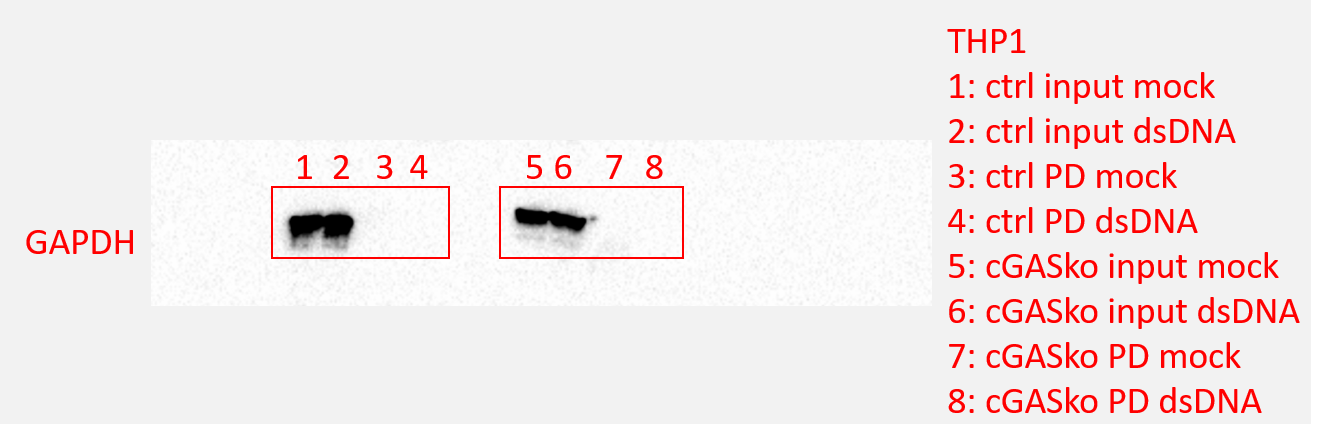

Supplement: Supplementary file 3 — Source Data for Expanded View [file EMBJ-42-e111961-s004.zip › EV Figures/EV Figure 3/EV Figure 3B/Ev FIG 3B_western_GAPDH.tif]

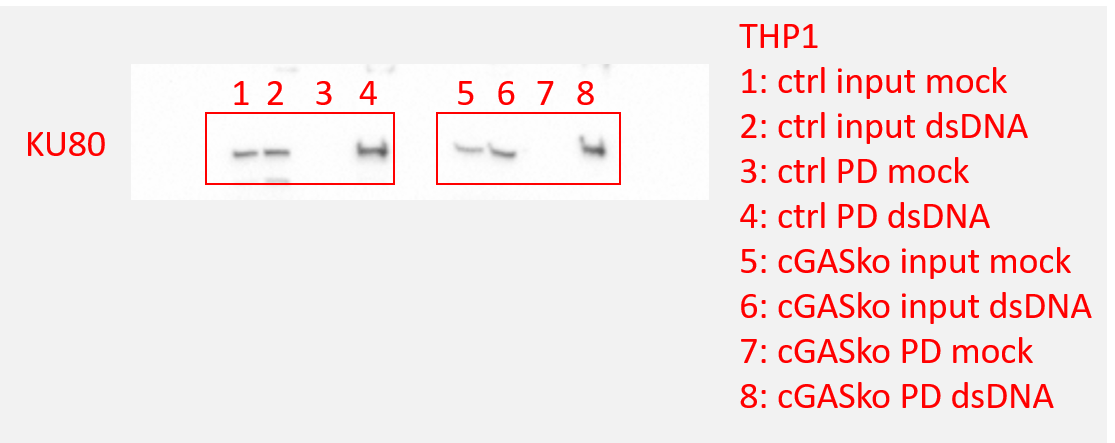

Supplement: Supplementary file 3 — Source Data for Expanded View [file EMBJ-42-e111961-s004.zip › EV Figures/EV Figure 3/EV Figure 3B/Ev FIG 3B _western_KU80.tif]

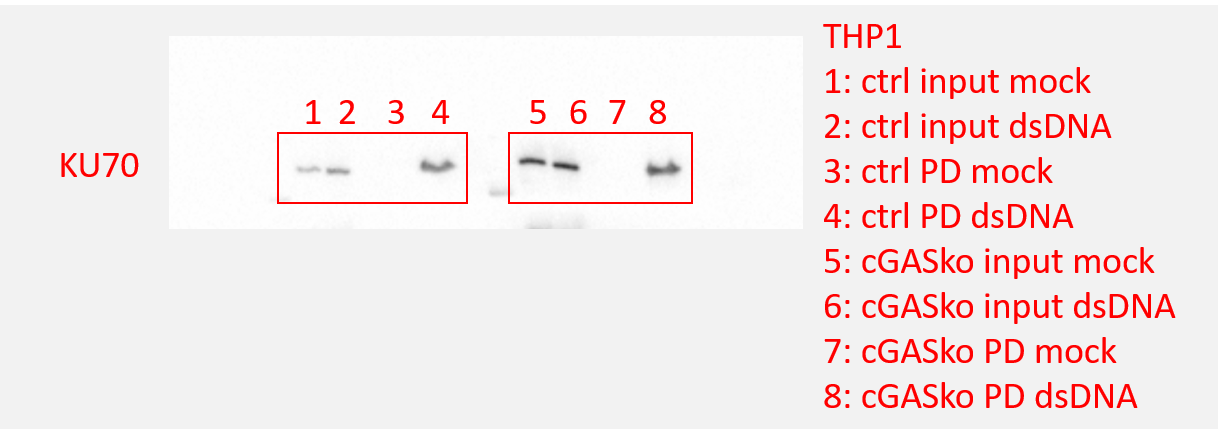

Supplement: Supplementary file 3 — Source Data for Expanded View [file EMBJ-42-e111961-s004.zip › EV Figures/EV Figure 3/EV Figure 3B/Ev FIG 3B_western_KU70.tif]

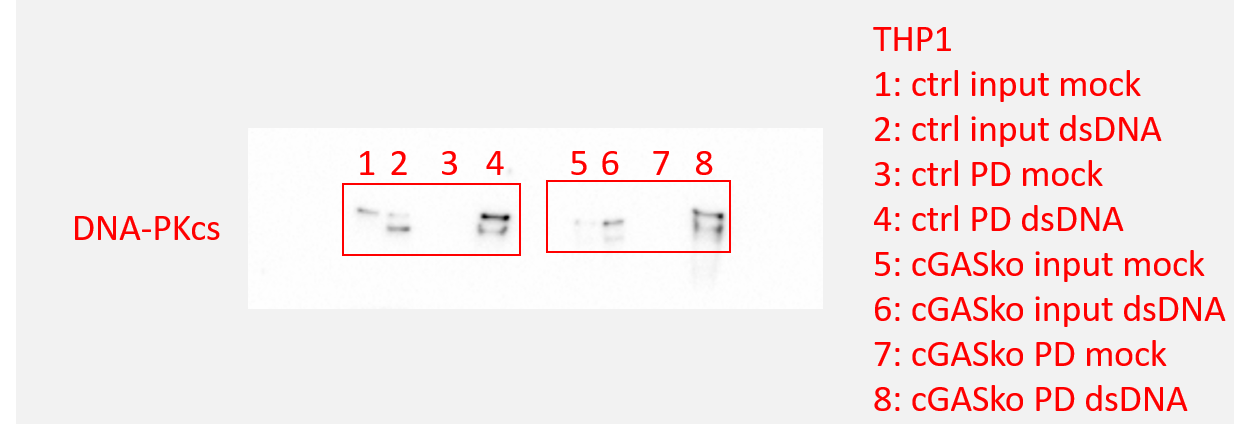

Supplement: Supplementary file 3 — Source Data for Expanded View [file EMBJ-42-e111961-s004.zip › EV Figures/EV Figure 3/EV Figure 3B/Ev Fig 3B_western_DNA-PKcs.tif]

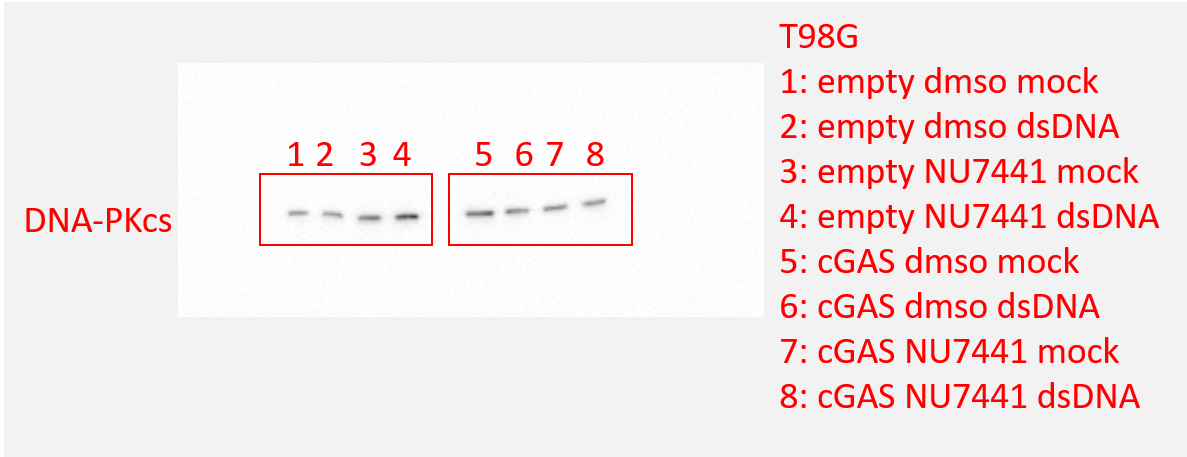

Supplement: Supplementary file 3 — Source Data for Expanded View [file EMBJ-42-e111961-s004.zip › EV Figures/EV Figure 3/EV Figure 3D/EV Fig 3D_western_DNA-PKcs.tif]

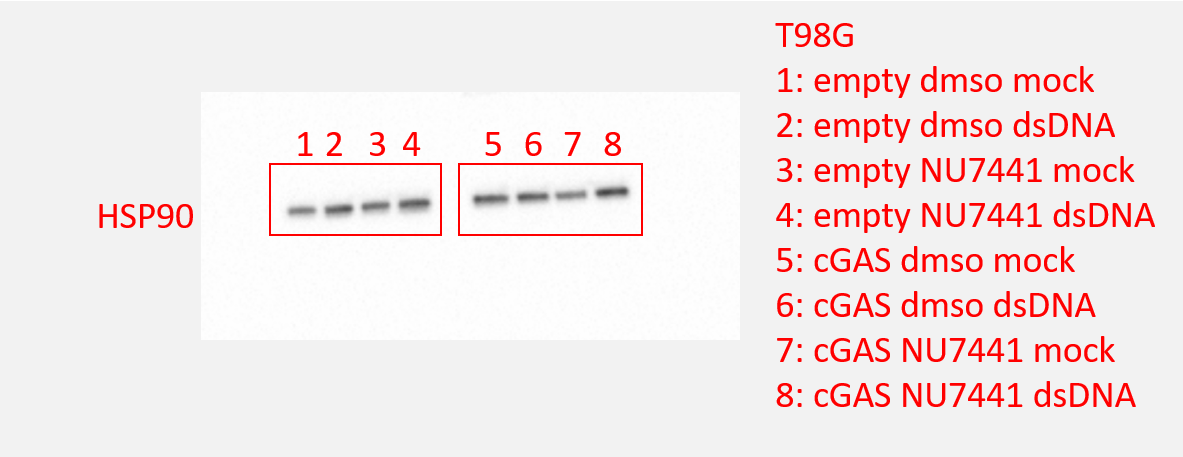

Supplement: Supplementary file 3 — Source Data for Expanded View [file EMBJ-42-e111961-s004.zip › EV Figures/EV Figure 3/EV Figure 3D/EV Fig 3D_western_HSP90.tif]

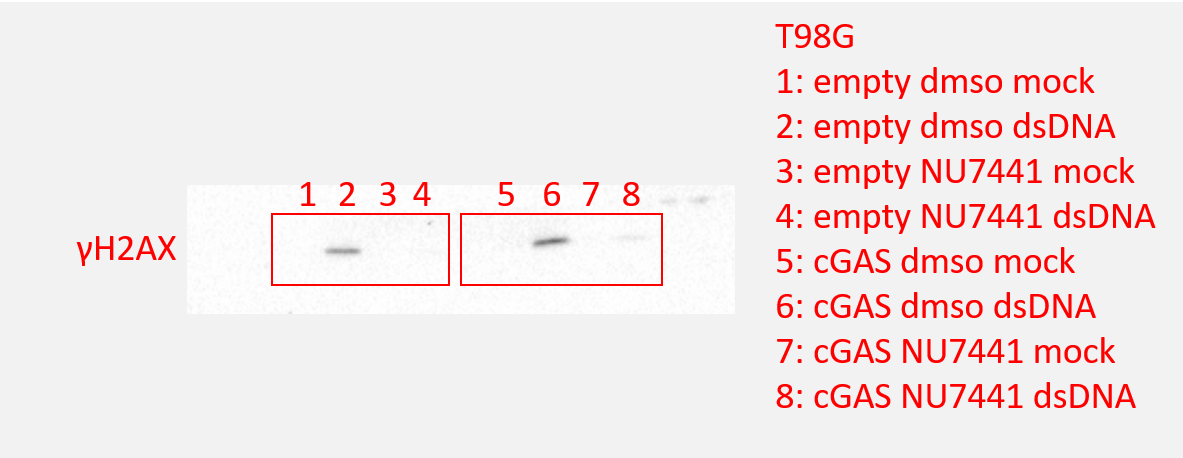

Supplement: Supplementary file 3 — Source Data for Expanded View [file EMBJ-42-e111961-s004.zip › EV Figures/EV Figure 3/EV Figure 3D/EV Fig 3D_western_gH2AX.tif]

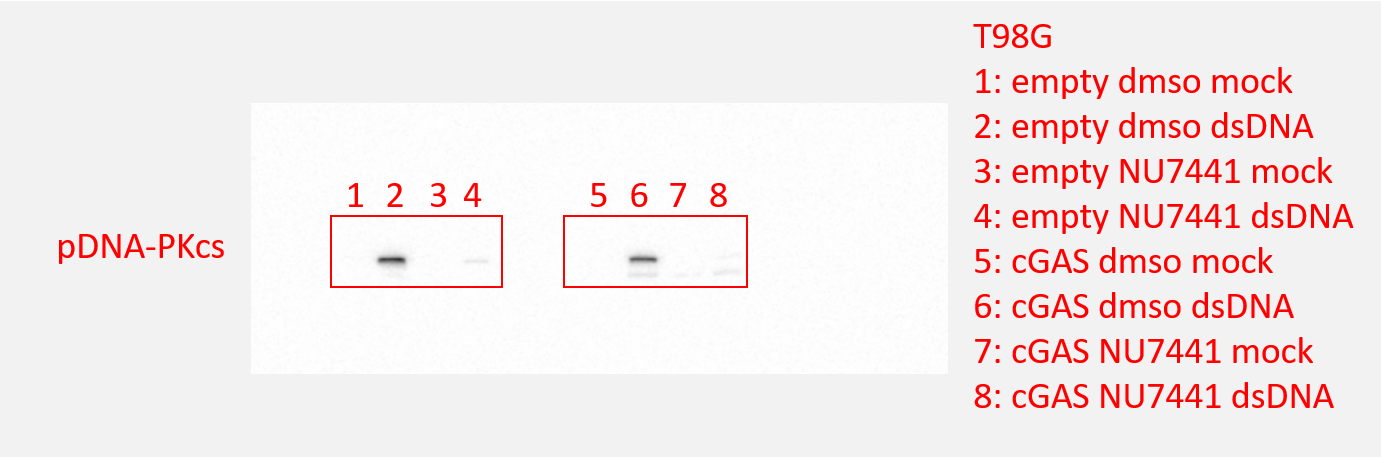

Supplement: Supplementary file 3 — Source Data for Expanded View [file EMBJ-42-e111961-s004.zip › EV Figures/EV Figure 3/EV Figure 3D/EV Fig 3D_western_pDNA-PKcs.tif]

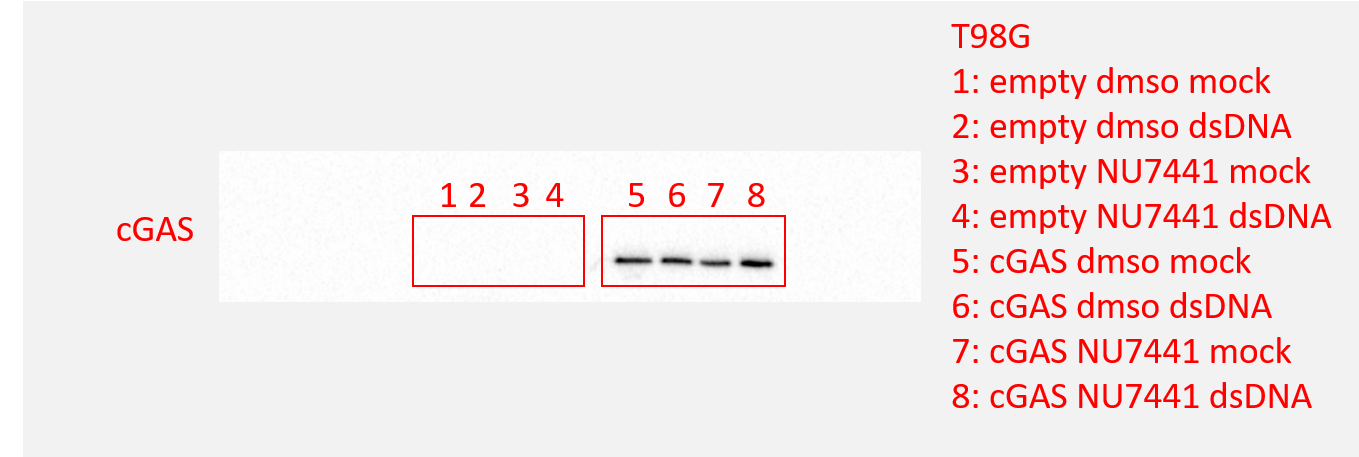

Supplement: Supplementary file 3 — Source Data for Expanded View [file EMBJ-42-e111961-s004.zip › EV Figures/EV Figure 3/EV Figure 3D/Ev Fig 3D_western_cGAS.tif]

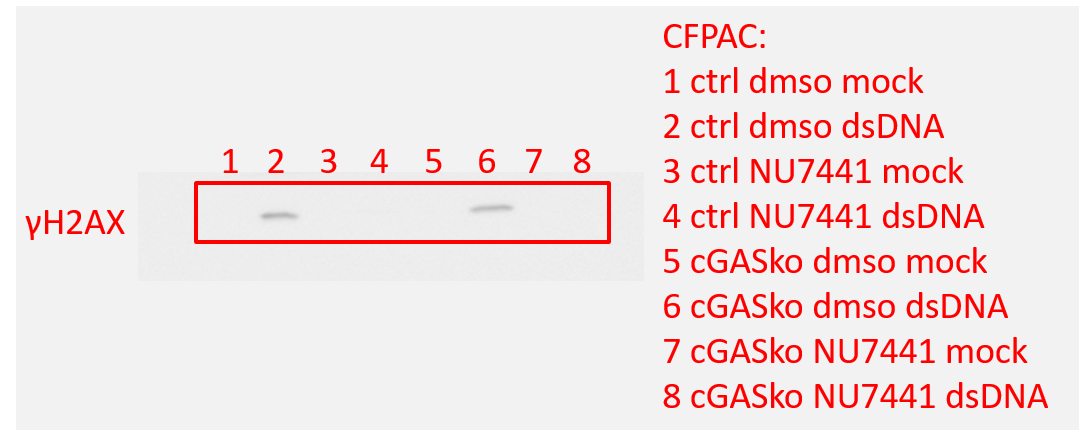

Supplement: Supplementary file 3 — Source Data for Expanded View [file EMBJ-42-e111961-s004.zip › EV Figures/EV Figure 3/EV Figure 3F/EV FIG 3F_western_gH2AX.tif]

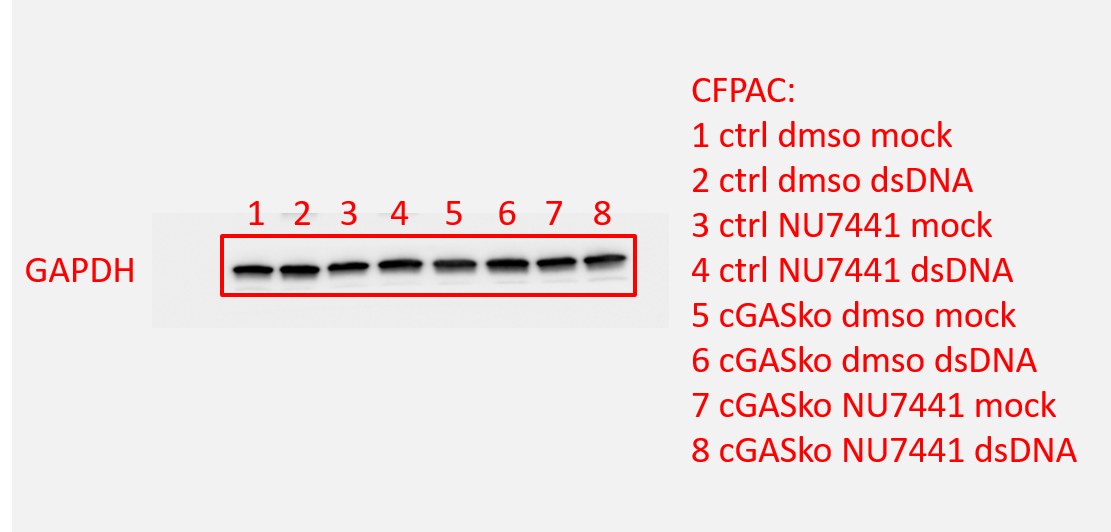

Supplement: Supplementary file 3 — Source Data for Expanded View [file EMBJ-42-e111961-s004.zip › EV Figures/EV Figure 3/EV Figure 3F/EV FIG 3F_western_GAPDH.tif]

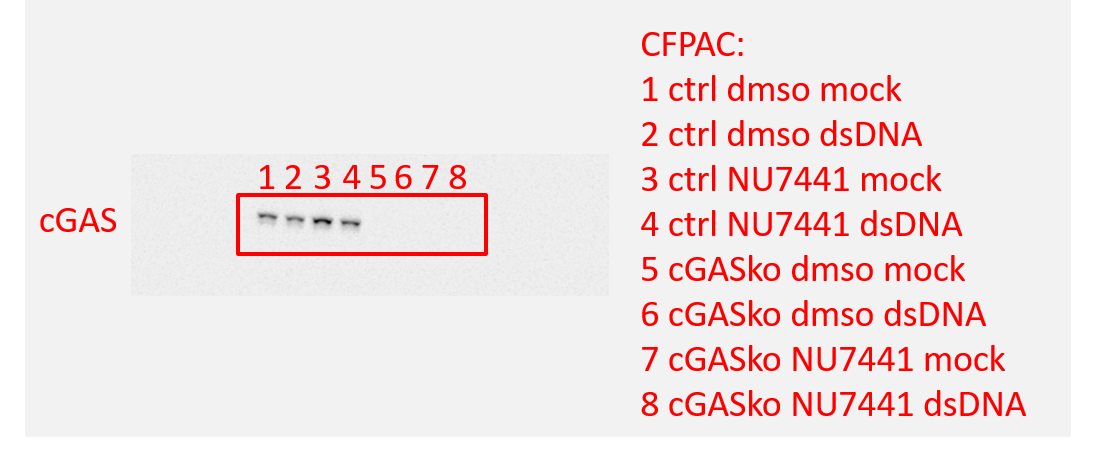

Supplement: Supplementary file 3 — Source Data for Expanded View [file EMBJ-42-e111961-s004.zip › EV Figures/EV Figure 3/EV Figure 3F/EV Fig 3F_western_cGAS.tif]

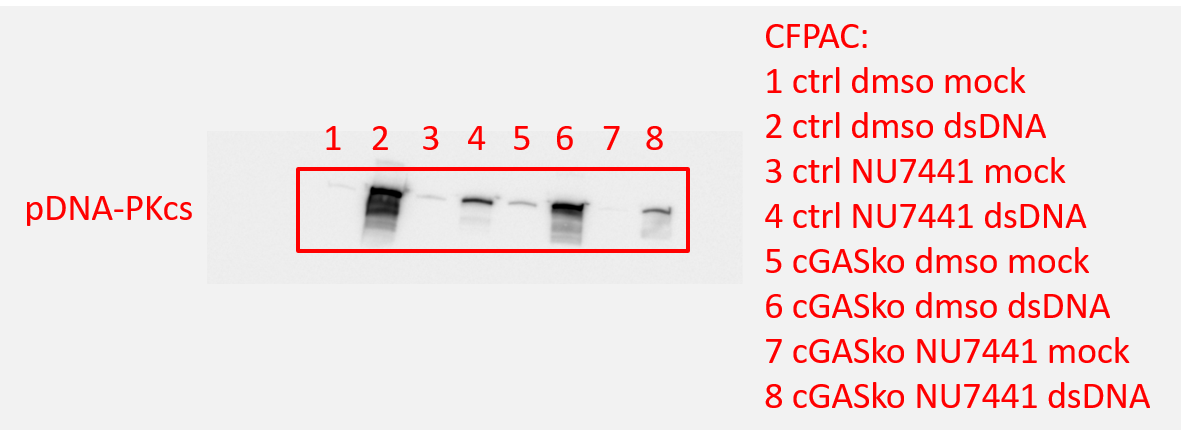

Supplement: Supplementary file 3 — Source Data for Expanded View [file EMBJ-42-e111961-s004.zip › EV Figures/EV Figure 3/EV Figure 3F/EV FIG 3F_western_pDNA-PKcs.tif]

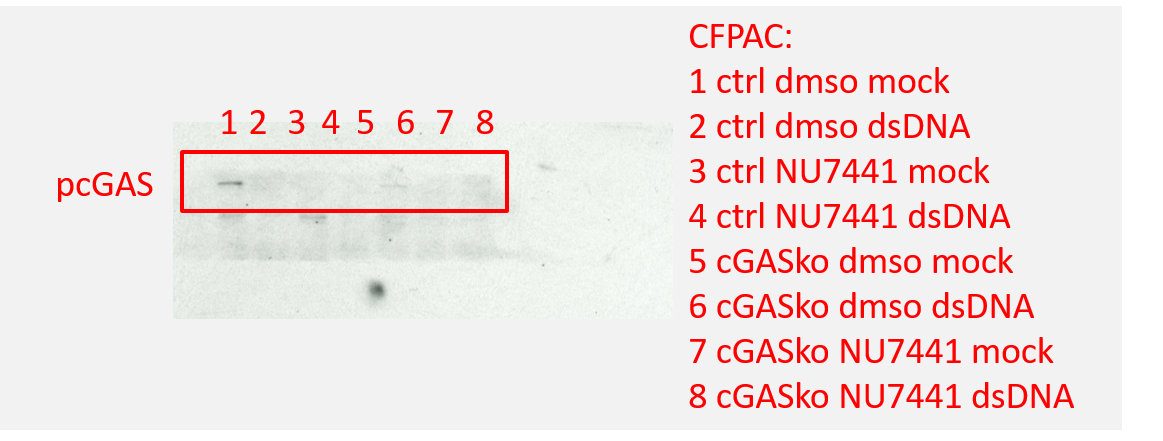

Supplement: Supplementary file 3 — Source Data for Expanded View [file EMBJ-42-e111961-s004.zip › EV Figures/EV Figure 3/EV Figure 3F/EV FIG 3F_western_pcGAS.tif]

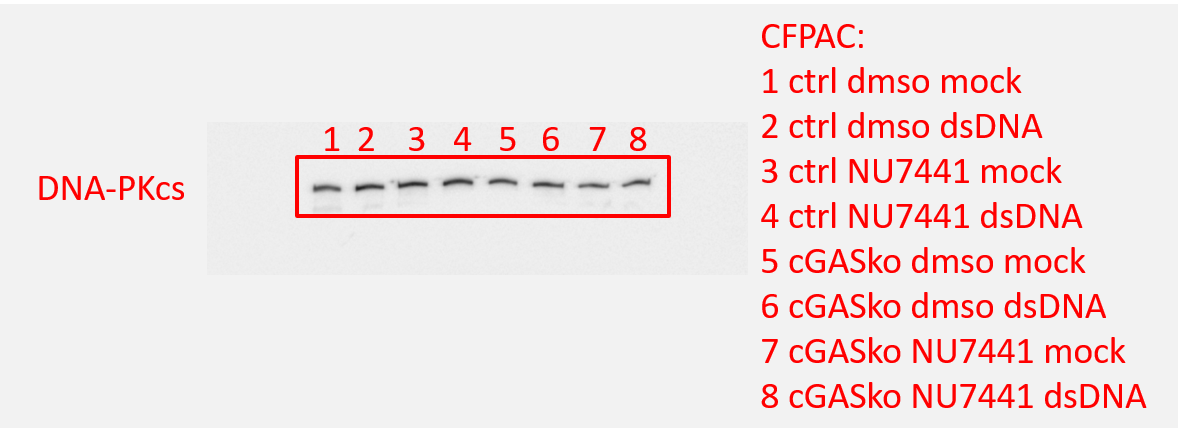

Supplement: Supplementary file 3 — Source Data for Expanded View [file EMBJ-42-e111961-s004.zip › EV Figures/EV Figure 3/EV Figure 3F/EV Fig 3F_western_DNA-PKcs.tif]

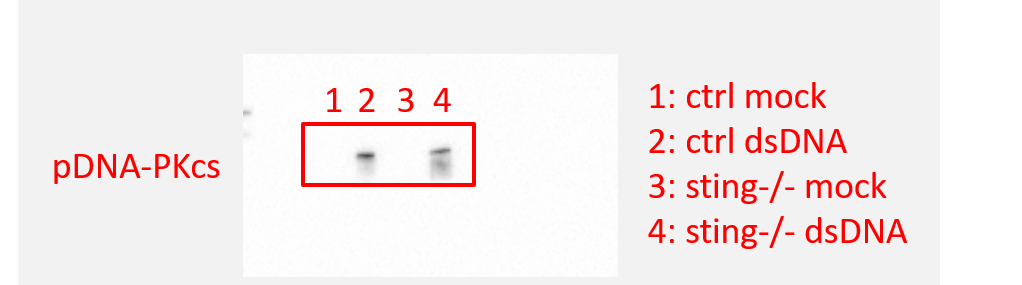

Supplement: Supplementary file 3 — Source Data for Expanded View [file EMBJ-42-e111961-s004.zip › EV Figures/EV Figure 2/EV Figure 2G/Ev Figure 2G_western_pDNA-PKcs.tif]

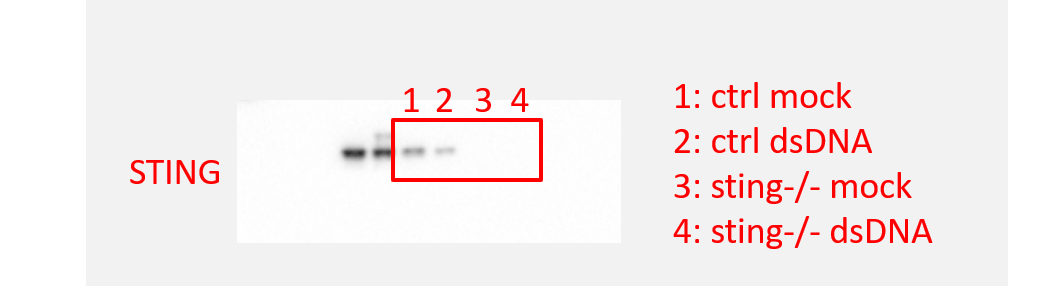

Supplement: Supplementary file 3 — Source Data for Expanded View [file EMBJ-42-e111961-s004.zip › EV Figures/EV Figure 2/EV Figure 2G/Ev Figure 2G_western_STING.tif]

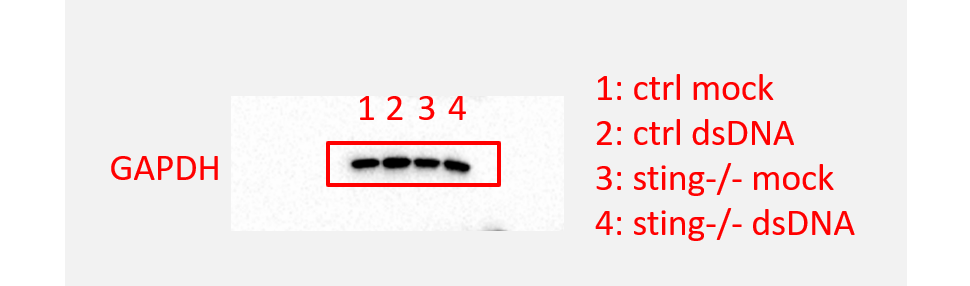

Supplement: Supplementary file 3 — Source Data for Expanded View [file EMBJ-42-e111961-s004.zip › EV Figures/EV Figure 2/EV Figure 2G/Ev Figure 2G_western_GAPDH.tif]

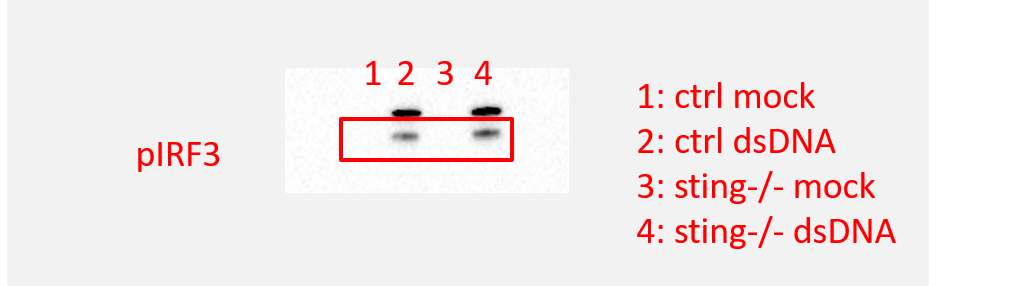

Supplement: Supplementary file 3 — Source Data for Expanded View [file EMBJ-42-e111961-s004.zip › EV Figures/EV Figure 2/EV Figure 2G/Ev Figure 2G_western_pIRF3.tif]

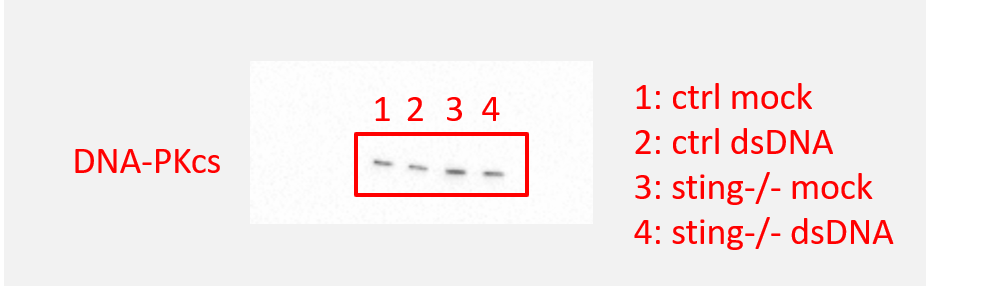

Supplement: Supplementary file 3 — Source Data for Expanded View [file EMBJ-42-e111961-s004.zip › EV Figures/EV Figure 2/EV Figure 2G/Ev Figure 2G_western_DNA-PKcs.tif]

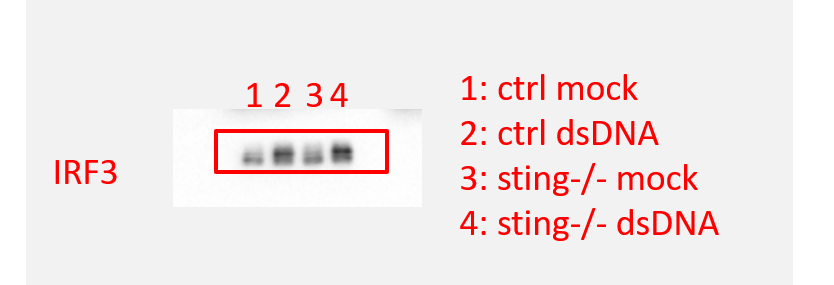

Supplement: Supplementary file 3 — Source Data for Expanded View [file EMBJ-42-e111961-s004.zip › EV Figures/EV Figure 2/EV Figure 2G/Ev Figure 2G_western_IRF3.tif]

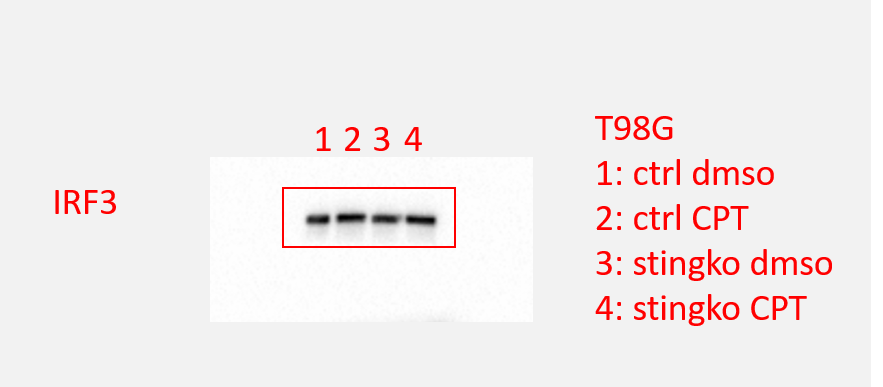

Supplement: Supplementary file 3 — Source Data for Expanded View [file EMBJ-42-e111961-s004.zip › EV Figures/EV Figure 2/EV Figure 2I/Ev Fig2I_western_IRF3.tif]

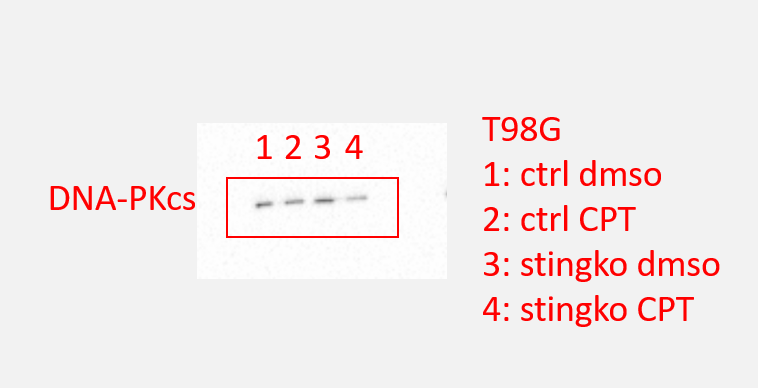

Supplement: Supplementary file 3 — Source Data for Expanded View [file EMBJ-42-e111961-s004.zip › EV Figures/EV Figure 2/EV Figure 2I/EV Fig2I_western_DNA-PKcs.tif]

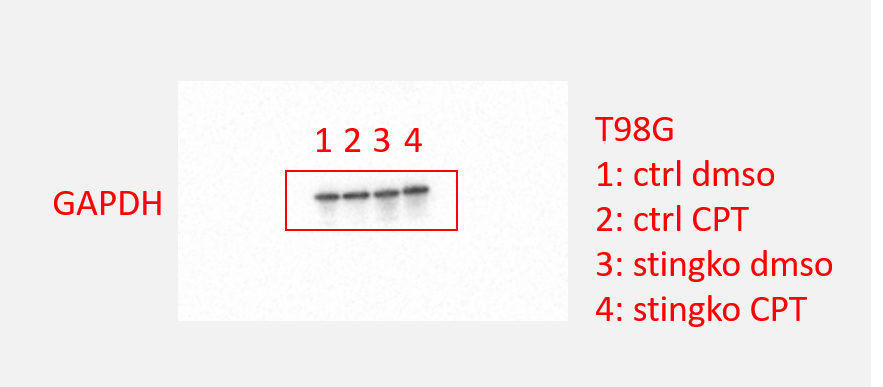

Supplement: Supplementary file 3 — Source Data for Expanded View [file EMBJ-42-e111961-s004.zip › EV Figures/EV Figure 2/EV Figure 2I/Ev Fig2I_western_GAPDH.tif]

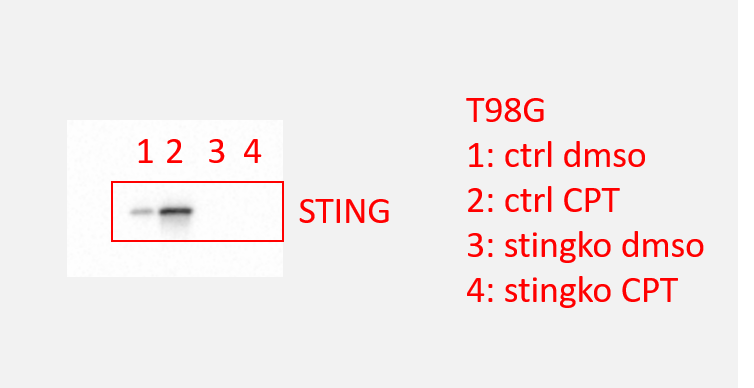

Supplement: Supplementary file 3 — Source Data for Expanded View [file EMBJ-42-e111961-s004.zip › EV Figures/EV Figure 2/EV Figure 2I/Ev Fig2I_western_STING.tif]

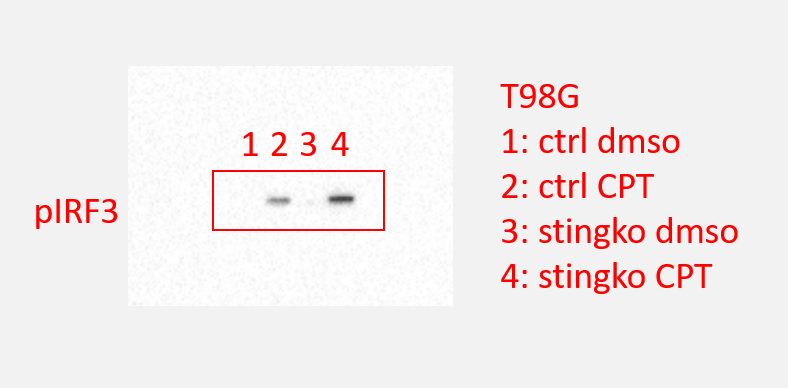

Supplement: Supplementary file 3 — Source Data for Expanded View [file EMBJ-42-e111961-s004.zip › EV Figures/EV Figure 2/EV Figure 2I/Ev Fig2I_western_pIRF3.tif]

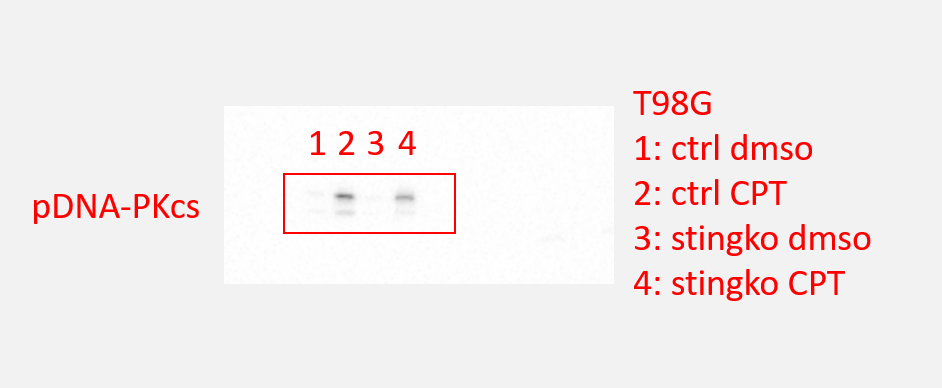

Supplement: Supplementary file 3 — Source Data for Expanded View [file EMBJ-42-e111961-s004.zip › EV Figures/EV Figure 2/EV Figure 2I/Ev Fig2I_western_pDNA-PKcs.tif]

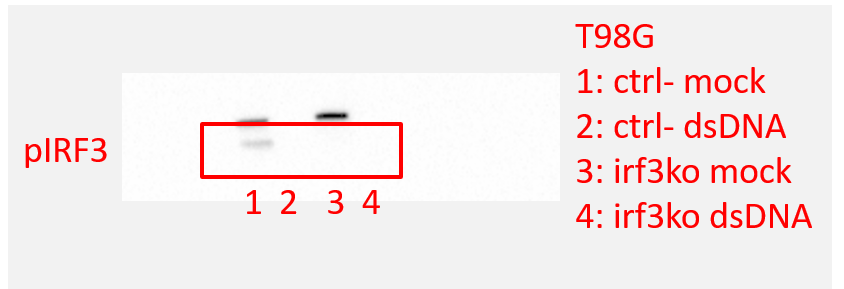

Supplement: Supplementary file 3 — Source Data for Expanded View [file EMBJ-42-e111961-s004.zip › EV Figures/EV Figure 2/EV Figure 2H/Ev Fig2H_western_pIRF3.tif]

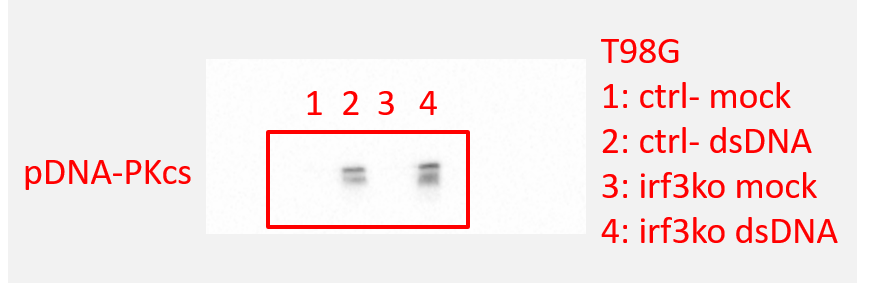

Supplement: Supplementary file 3 — Source Data for Expanded View [file EMBJ-42-e111961-s004.zip › EV Figures/EV Figure 2/EV Figure 2H/Ev Fig2H _western_pDNA-PKcs.tif]

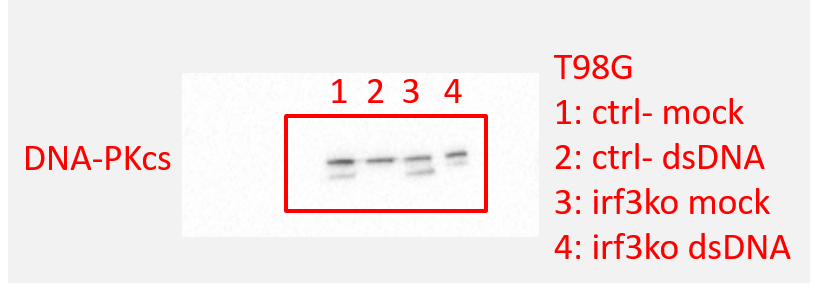

Supplement: Supplementary file 3 — Source Data for Expanded View [file EMBJ-42-e111961-s004.zip › EV Figures/EV Figure 2/EV Figure 2H/Ev Fig2H_western_DNA-PKcs.tif]

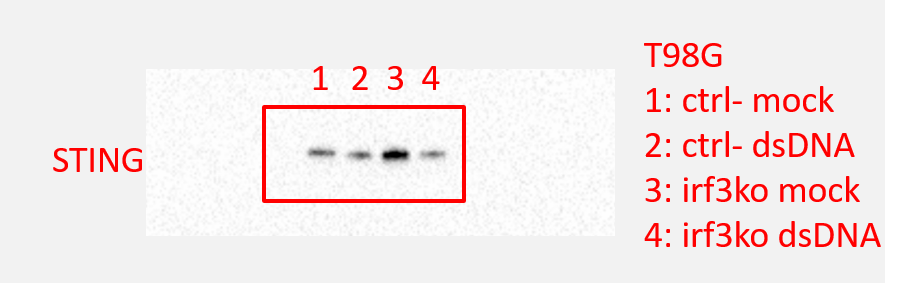

Supplement: Supplementary file 3 — Source Data for Expanded View [file EMBJ-42-e111961-s004.zip › EV Figures/EV Figure 2/EV Figure 2H/Ev Fig2H_western_STING.tif]

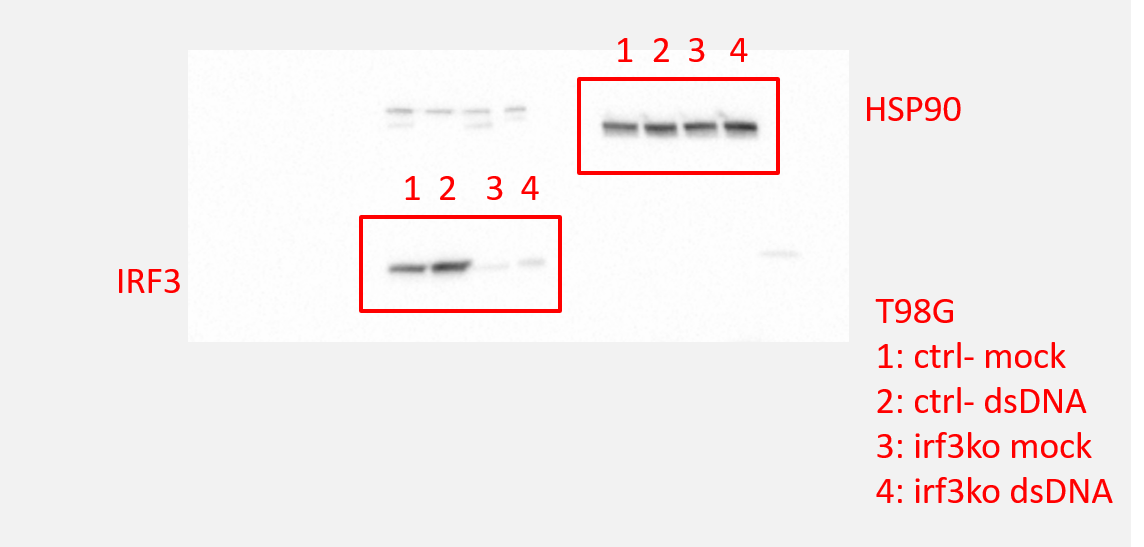

Supplement: Supplementary file 3 — Source Data for Expanded View [file EMBJ-42-e111961-s004.zip › EV Figures/EV Figure 2/EV Figure 2H/Ev Fig2H_western_HSP90 & IRF3.tif]

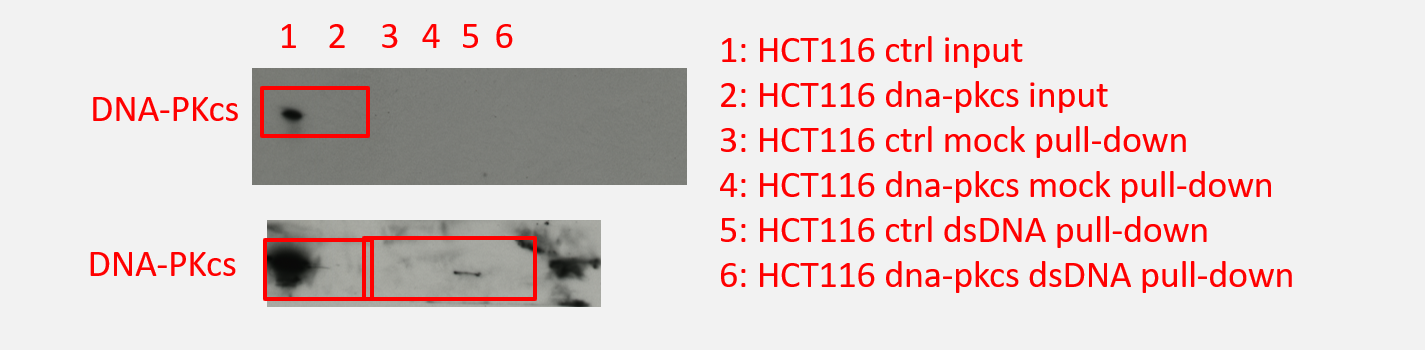

Supplement: Supplementary file 3 — Source Data for Expanded View [file EMBJ-42-e111961-s004.zip › EV Figures/EV Figure 2/EV Figure 2C/Ev Fig 2C_western_DNA-PKcs.tif]

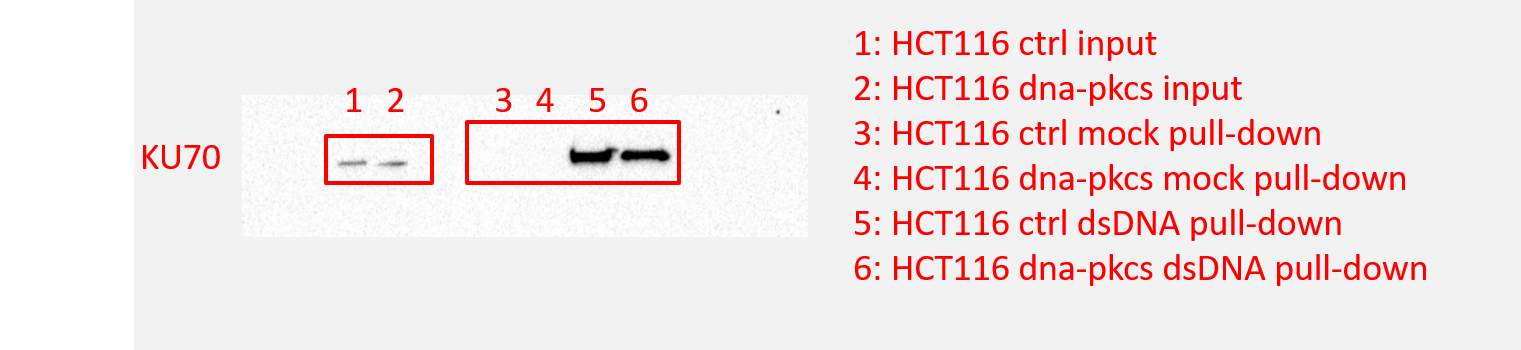

Supplement: Supplementary file 3 — Source Data for Expanded View [file EMBJ-42-e111961-s004.zip › EV Figures/EV Figure 2/EV Figure 2C/Ev Fig 2C_western_KU70.tif]

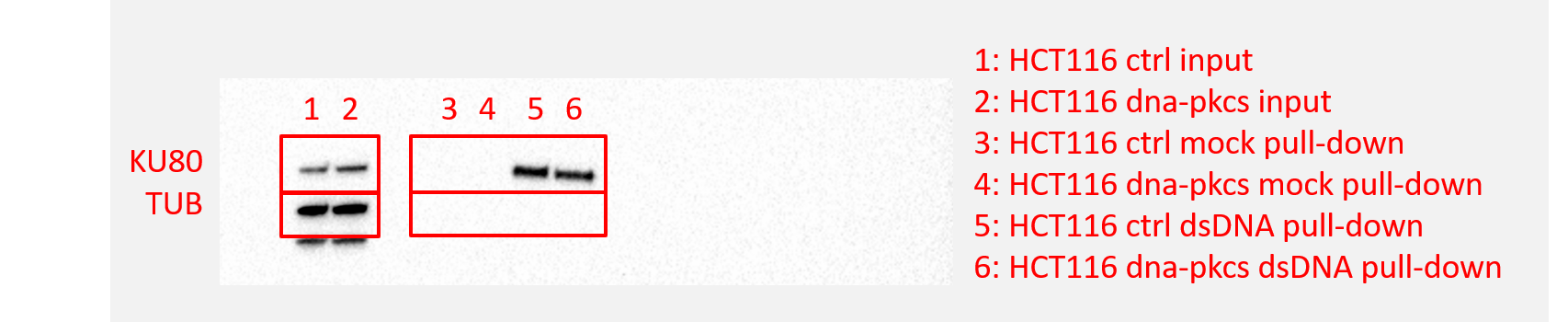

Supplement: Supplementary file 3 — Source Data for Expanded View [file EMBJ-42-e111961-s004.zip › EV Figures/EV Figure 2/EV Figure 2C/Ev Fig 2C_western_KU80 & Tub.tif]

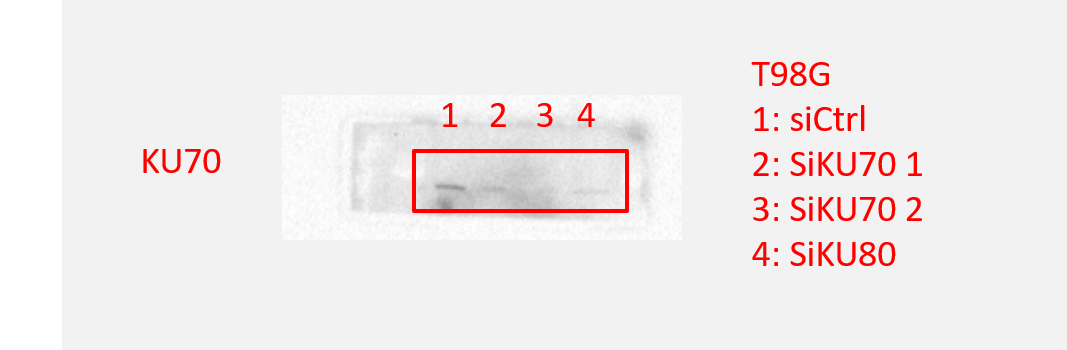

Supplement: Supplementary file 3 — Source Data for Expanded View [file EMBJ-42-e111961-s004.zip › EV Figures/EV Figure 2/EV Figure 2D/Ev Fig 2D_western_KU70.tif]

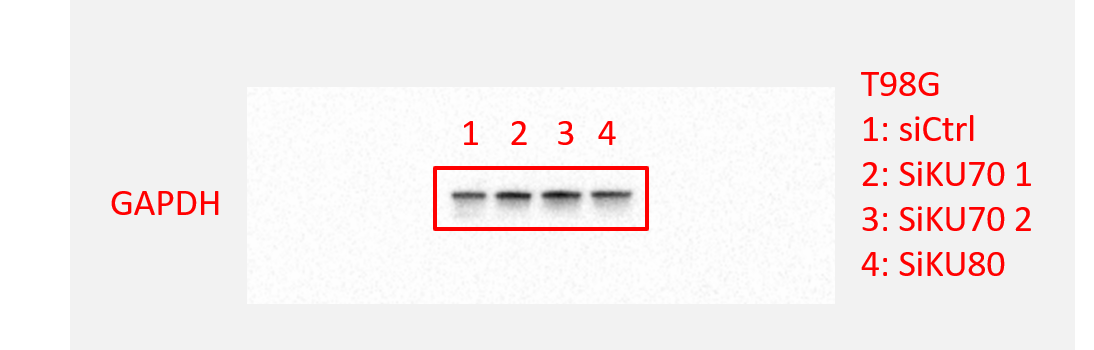

Supplement: Supplementary file 3 — Source Data for Expanded View [file EMBJ-42-e111961-s004.zip › EV Figures/EV Figure 2/EV Figure 2D/Ev Fig 2D_western_gapdh.tif]

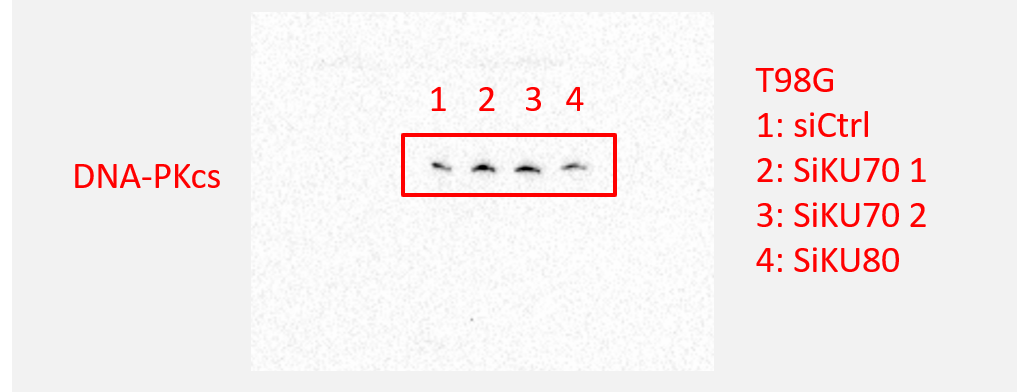

Supplement: Supplementary file 3 — Source Data for Expanded View [file EMBJ-42-e111961-s004.zip › EV Figures/EV Figure 2/EV Figure 2D/Ev Fig 2D_western_DNA-PKcs.tif]

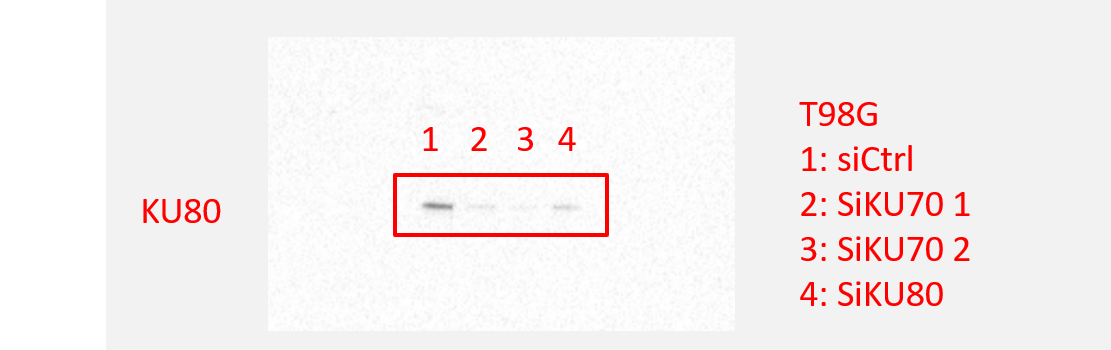

Supplement: Supplementary file 3 — Source Data for Expanded View [file EMBJ-42-e111961-s004.zip › EV Figures/EV Figure 2/EV Figure 2D/Ev Fig 2D_western_KU80.tif]

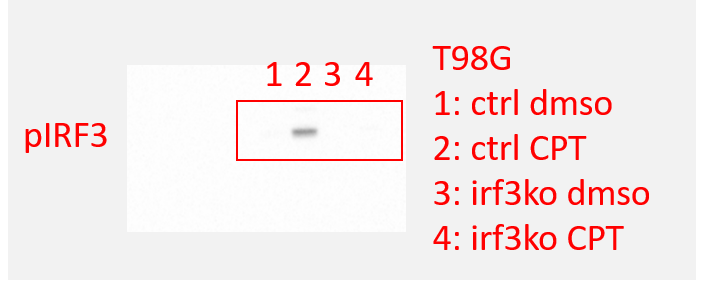

Supplement: Supplementary file 3 — Source Data for Expanded View [file EMBJ-42-e111961-s004.zip › EV Figures/EV Figure 2/EV Figure 2J/Ev Fig2J_western_pIRF3.tif]

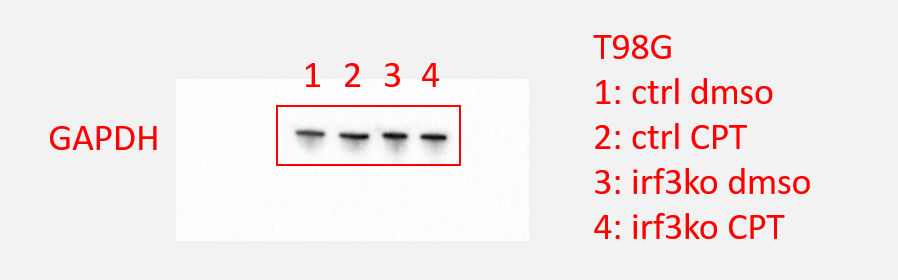

Supplement: Supplementary file 3 — Source Data for Expanded View [file EMBJ-42-e111961-s004.zip › EV Figures/EV Figure 2/EV Figure 2J/Ev Fig2J_western_GAPDH.tif]

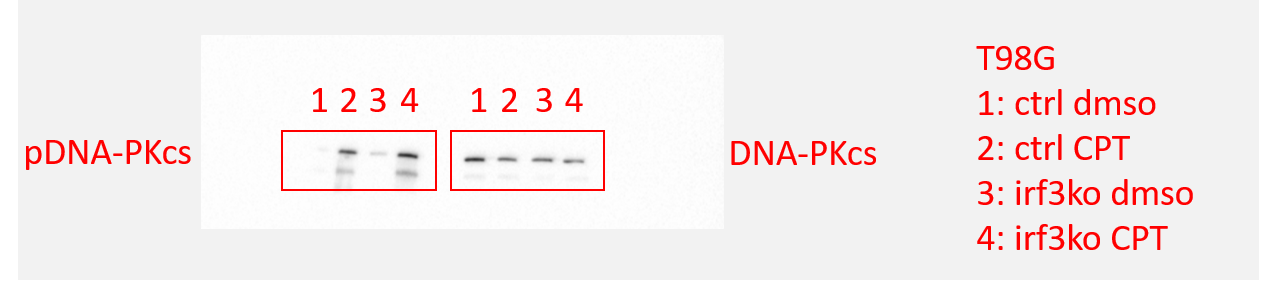

Supplement: Supplementary file 3 — Source Data for Expanded View [file EMBJ-42-e111961-s004.zip › EV Figures/EV Figure 2/EV Figure 2J/Ev Fig2J_western_pDNA-PKcs & DNA-PKcs.tif]

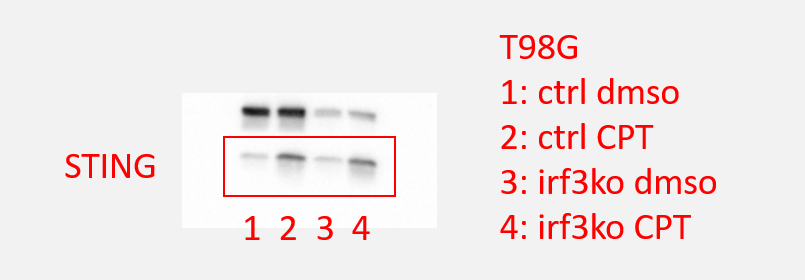

Supplement: Supplementary file 3 — Source Data for Expanded View [file EMBJ-42-e111961-s004.zip › EV Figures/EV Figure 2/EV Figure 2J/Ev Fig2J_western_STING.tif]

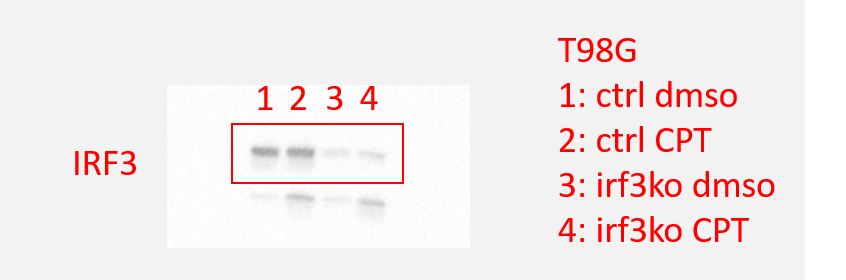

Supplement: Supplementary file 3 — Source Data for Expanded View [file EMBJ-42-e111961-s004.zip › EV Figures/EV Figure 2/EV Figure 2J/Ev Fig2J_western_IRF3.tif]

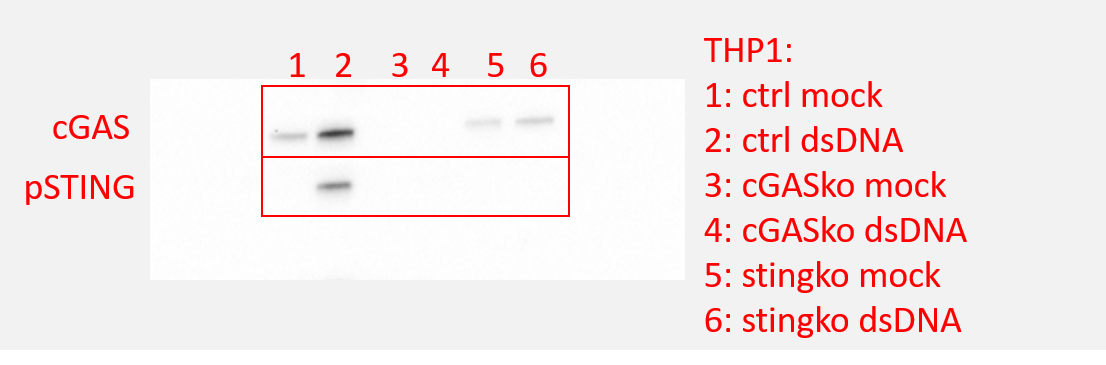

Supplement: Supplementary file 3 — Source Data for Expanded View [file EMBJ-42-e111961-s004.zip › EV Figures/EV Figure 2/EV Figure 2K/Ev Fig 2K_western_cGAS & pSTING.tif]

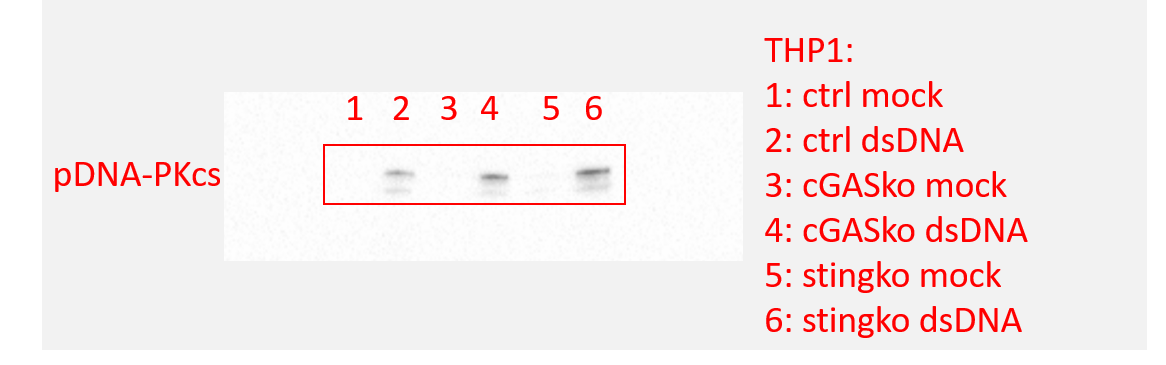

Supplement: Supplementary file 3 — Source Data for Expanded View [file EMBJ-42-e111961-s004.zip › EV Figures/EV Figure 2/EV Figure 2K/Ev Fig 2K_western_pDNA-PKcs.tif]

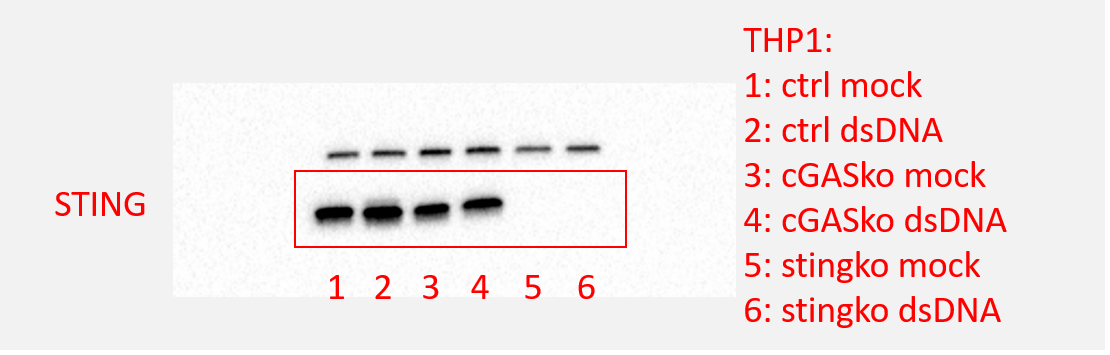

Supplement: Supplementary file 3 — Source Data for Expanded View [file EMBJ-42-e111961-s004.zip › EV Figures/EV Figure 2/EV Figure 2K/Ev Fig 2K_western_STING.tif]

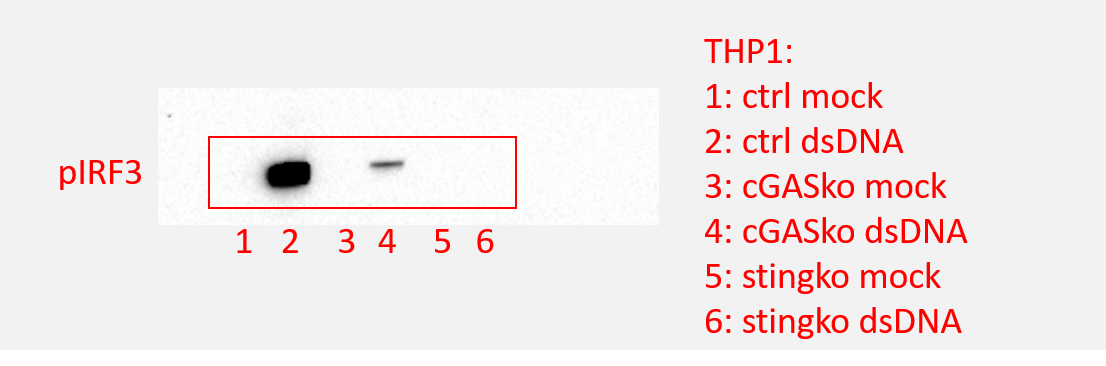

Supplement: Supplementary file 3 — Source Data for Expanded View [file EMBJ-42-e111961-s004.zip › EV Figures/EV Figure 2/EV Figure 2K/Ev Fig 2K_western_pIRF3.tif]

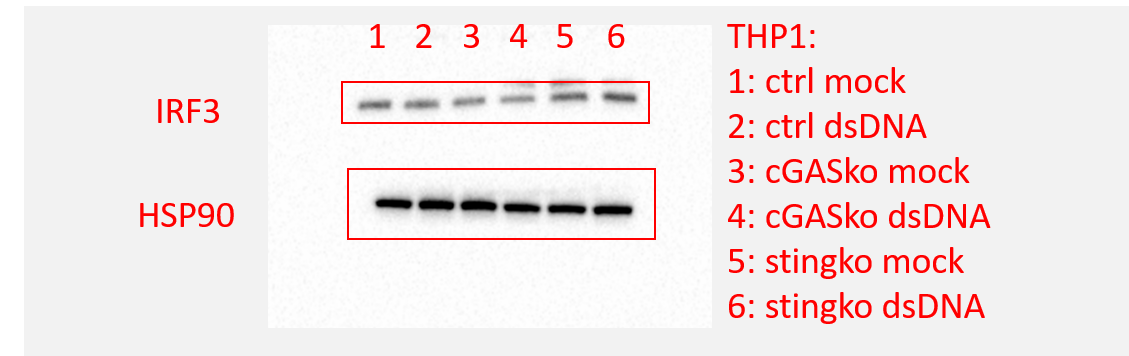

Supplement: Supplementary file 3 — Source Data for Expanded View [file EMBJ-42-e111961-s004.zip › EV Figures/EV Figure 2/EV Figure 2K/Ev Fig 2K_western_HSP90 & IRF3.tif]

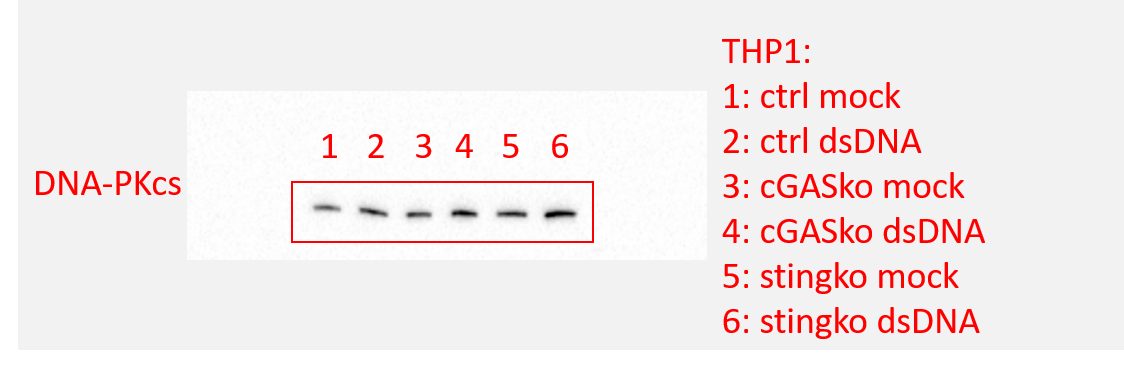

Supplement: Supplementary file 3 — Source Data for Expanded View [file EMBJ-42-e111961-s004.zip › EV Figures/EV Figure 2/EV Figure 2K/Ev Fig 2K_western_DNA-PKcs.tif]

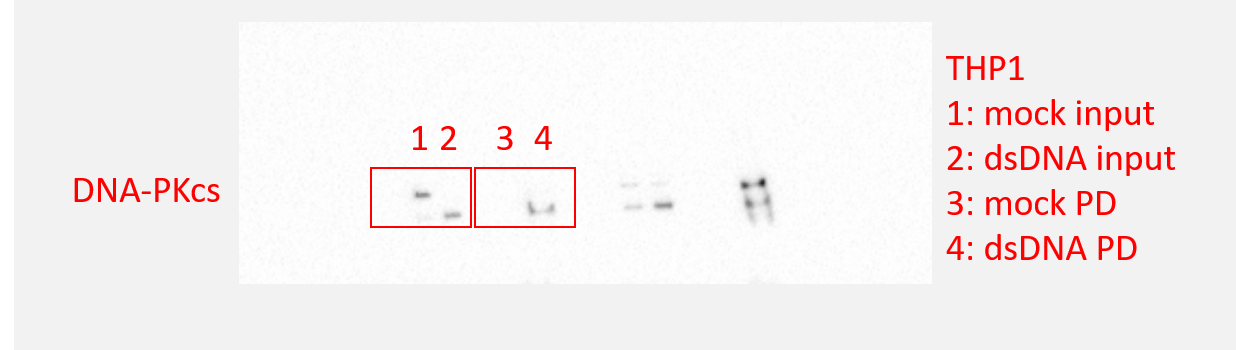

Supplement: Supplementary file 3 — Source Data for Expanded View [file EMBJ-42-e111961-s004.zip › EV Figures/EV Figure 2/EV Figure 2B/Ev Fig 2B_western_DNA-PKcs.tif]

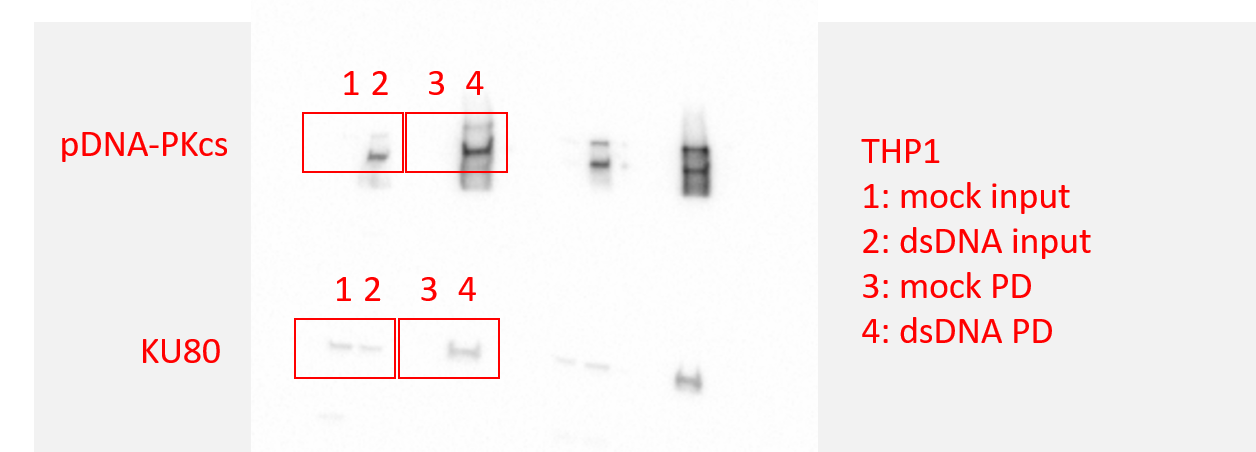

Supplement: Supplementary file 3 — Source Data for Expanded View [file EMBJ-42-e111961-s004.zip › EV Figures/EV Figure 2/EV Figure 2B/Ev Fig 2B_western_pDNA-PKcs & KU80.tif]

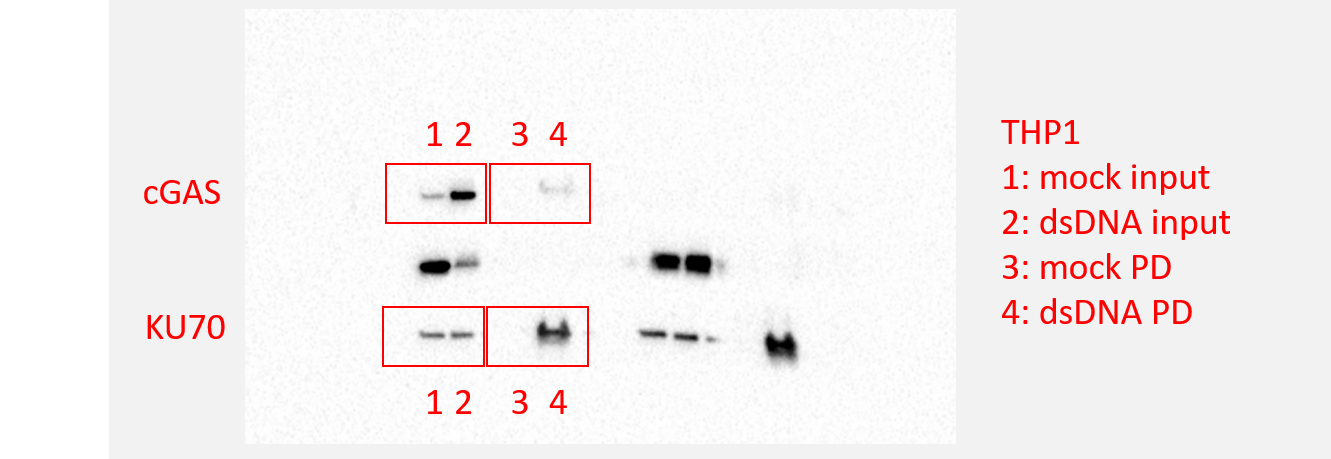

Supplement: Supplementary file 3 — Source Data for Expanded View [file EMBJ-42-e111961-s004.zip › EV Figures/EV Figure 2/EV Figure 2B/Ev Fig 2B_western_cGAS & KU70.tif]

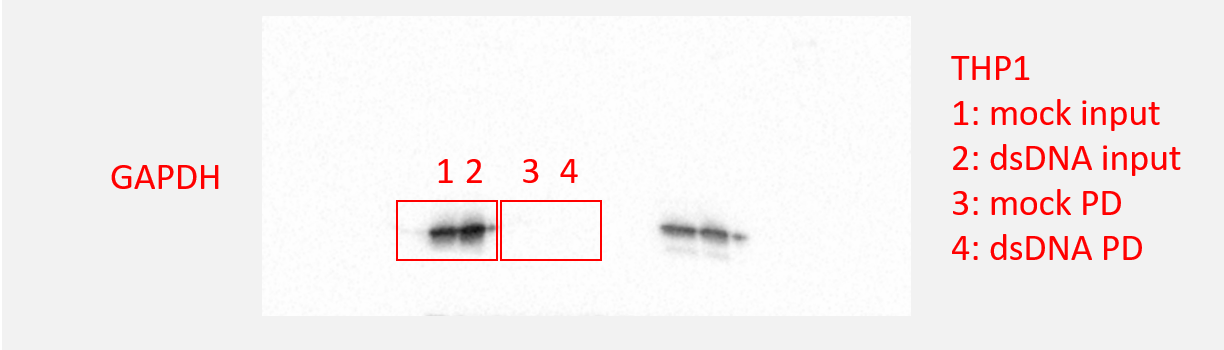

Supplement: Supplementary file 3 — Source Data for Expanded View [file EMBJ-42-e111961-s004.zip › EV Figures/EV Figure 2/EV Figure 2B/Ev Fig 2B_western_gapdh.tif]

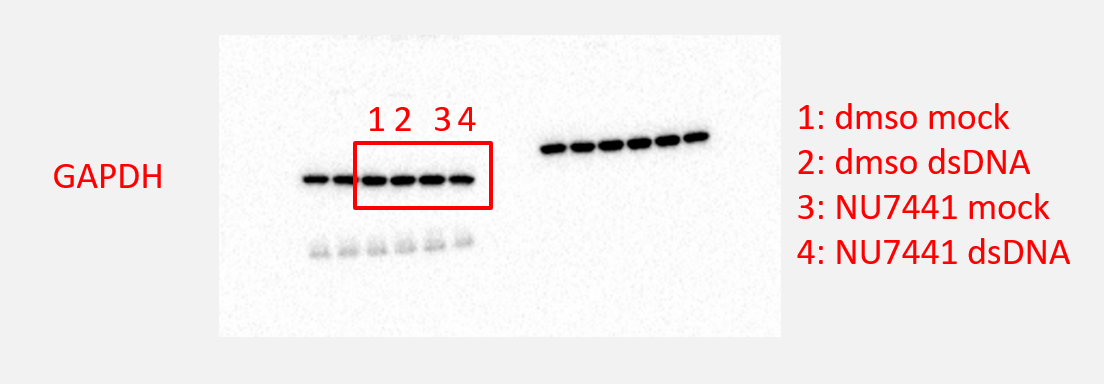

Supplement: Supplementary file 5 — Source Data for Figure 1 [file EMBJ-42-e111961-s009.zip › Figure 1/Figure 1F/Fig 1F_western_GAPDH.tif]

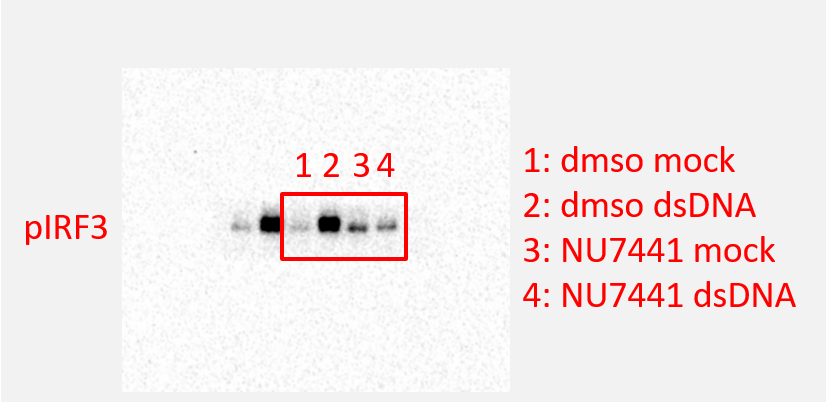

Supplement: Supplementary file 5 — Source Data for Figure 1 [file EMBJ-42-e111961-s009.zip › Figure 1/Figure 1F/Fig 1F_western_pIRF3.tif]

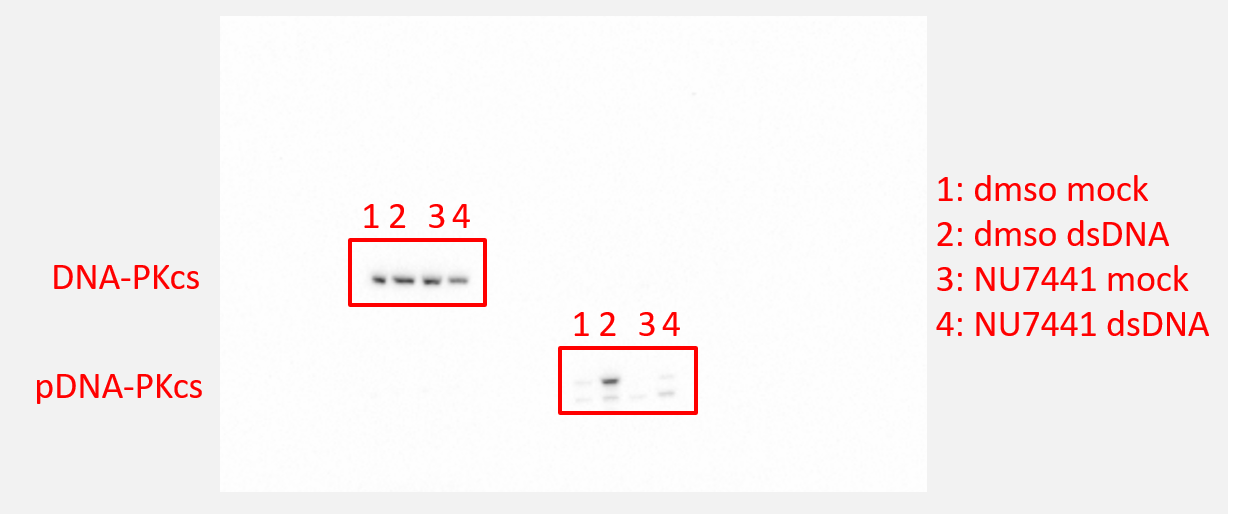

Supplement: Supplementary file 5 — Source Data for Figure 1 [file EMBJ-42-e111961-s009.zip › Figure 1/Figure 1F/Fig 1F_western_DNA-PKcs & pDNA-PKcs.tif]

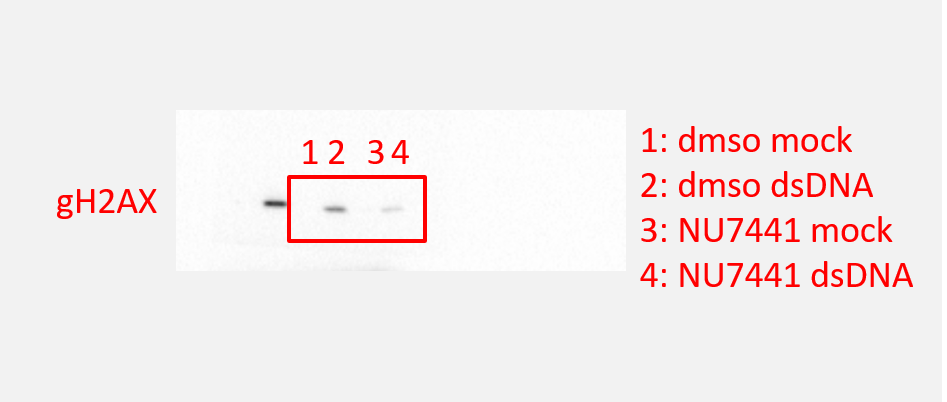

Supplement: Supplementary file 5 — Source Data for Figure 1 [file EMBJ-42-e111961-s009.zip › Figure 1/Figure 1F/Fig 1F_western_gH2AX.tif]

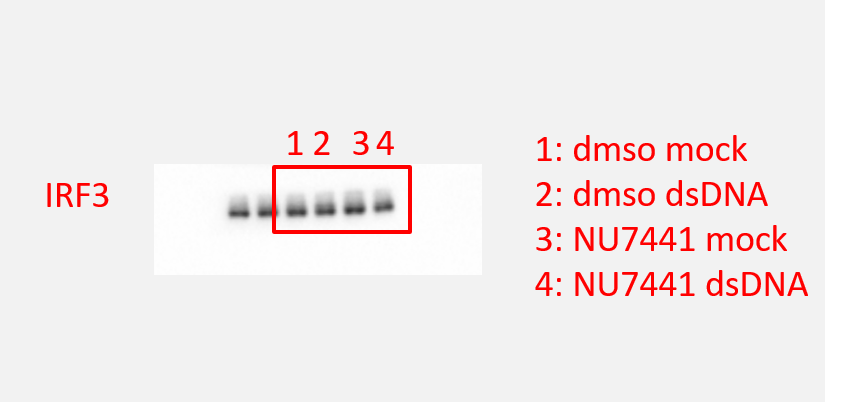

Supplement: Supplementary file 5 — Source Data for Figure 1 [file EMBJ-42-e111961-s009.zip › Figure 1/Figure 1F/Fig 1F_western_IRF3.tif]

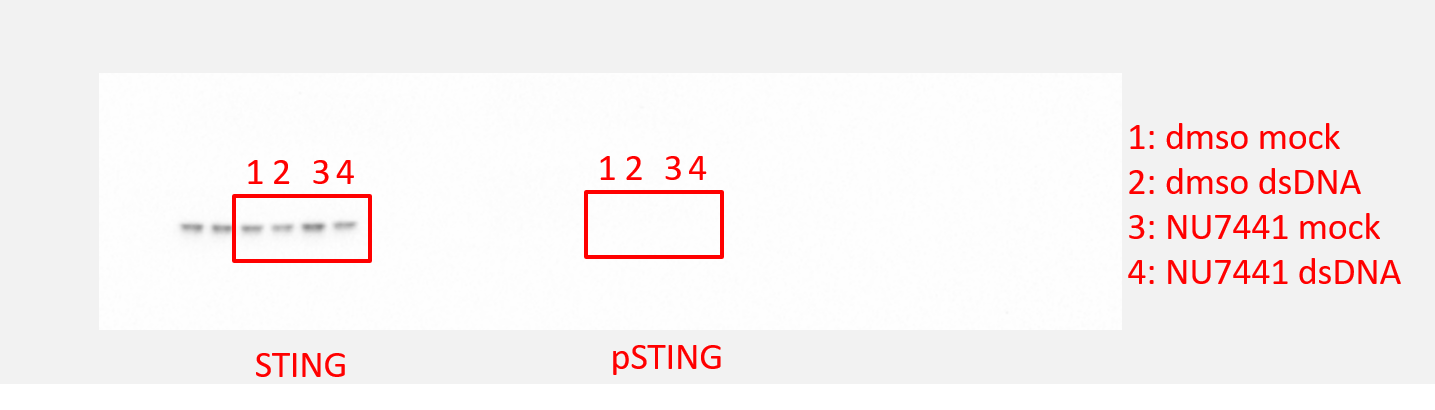

Supplement: Supplementary file 5 — Source Data for Figure 1 [file EMBJ-42-e111961-s009.zip › Figure 1/Figure 1F/Fig 1F_western_pSTING & STING.tif]

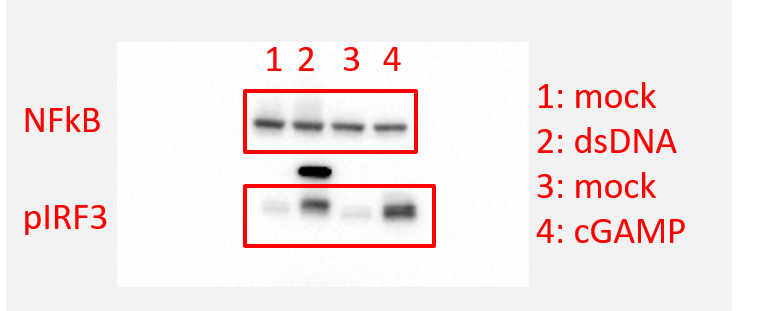

Supplement: Supplementary file 5 — Source Data for Figure 1 [file EMBJ-42-e111961-s009.zip › Figure 1/Figure 1A/Fig1A_western_NFkB & pIRF3.tif]

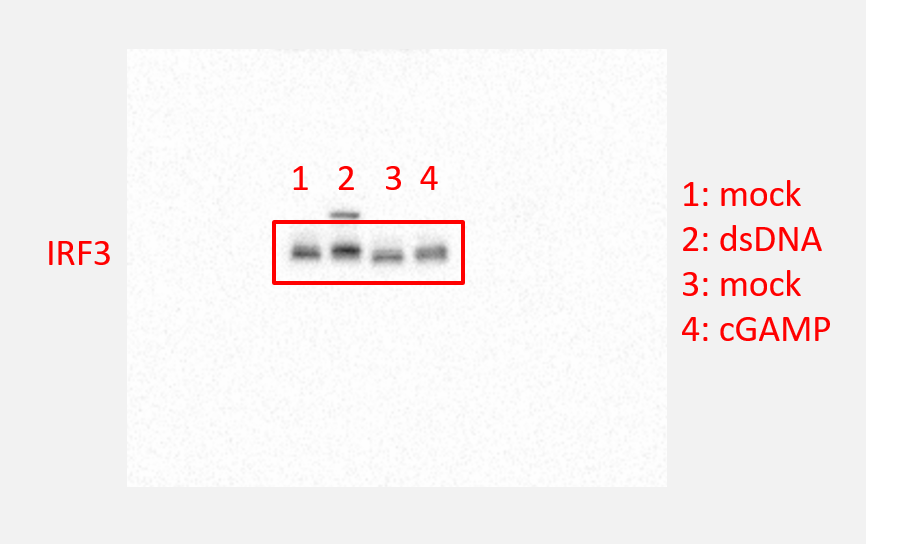

Supplement: Supplementary file 5 — Source Data for Figure 1 [file EMBJ-42-e111961-s009.zip › Figure 1/Figure 1A/Fig1A_western_IRF3.tif]

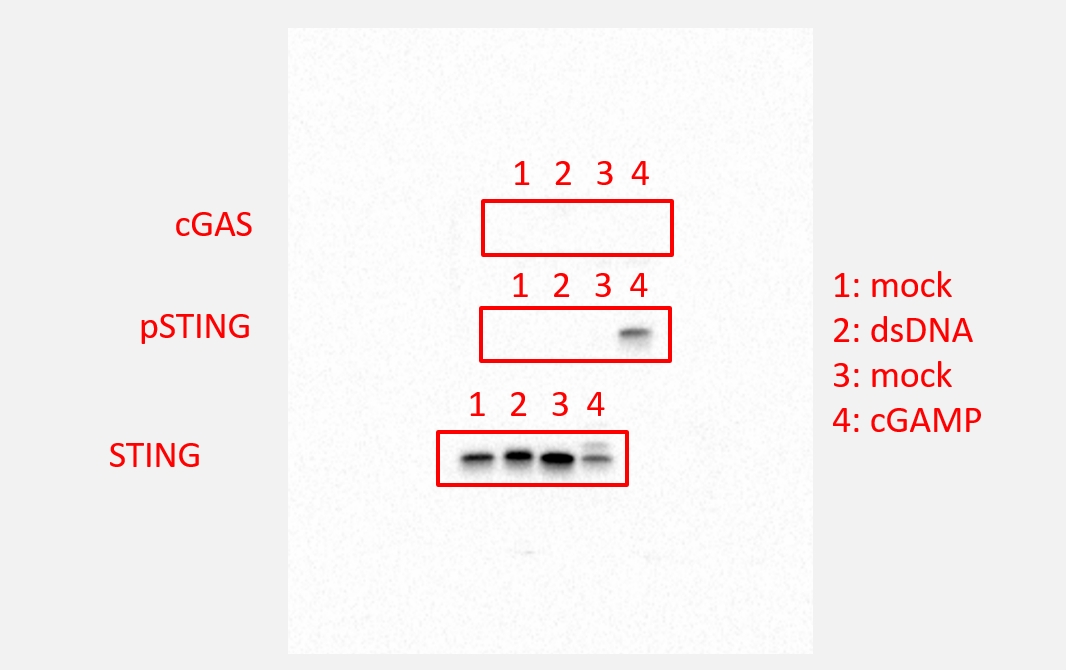

Supplement: Supplementary file 5 — Source Data for Figure 1 [file EMBJ-42-e111961-s009.zip › Figure 1/Figure 1A/Fig1A_western_cGAS, pSTING & STING.tif]

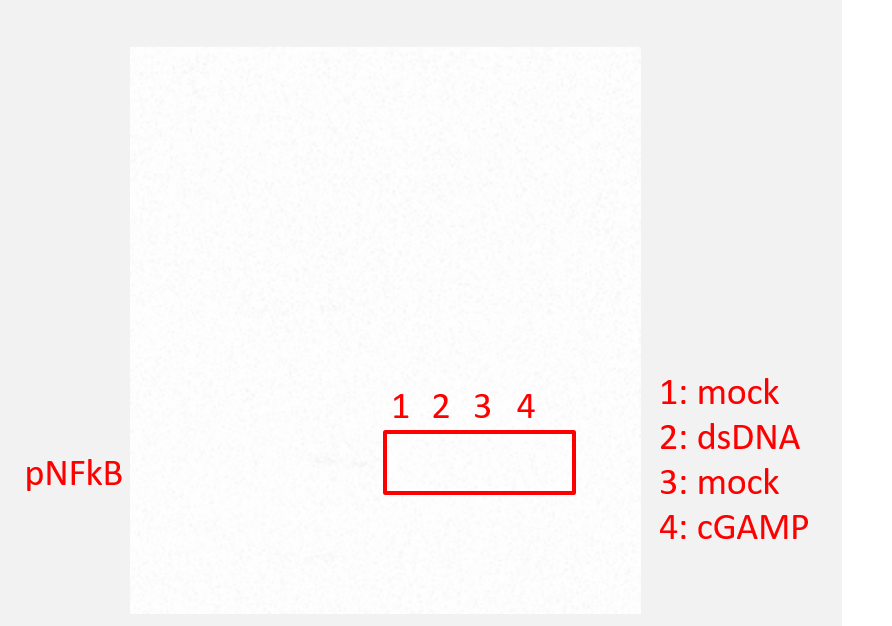

Supplement: Supplementary file 5 — Source Data for Figure 1 [file EMBJ-42-e111961-s009.zip › Figure 1/Figure 1A/Fig1A_western_pNFkB.tif]

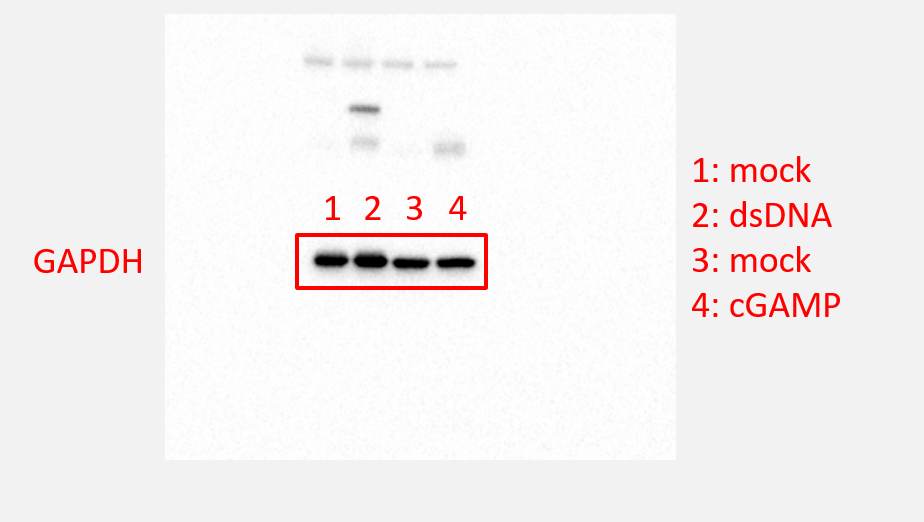

Supplement: Supplementary file 5 — Source Data for Figure 1 [file EMBJ-42-e111961-s009.zip › Figure 1/Figure 1A/Fig1A_western_GAPDH.tif]

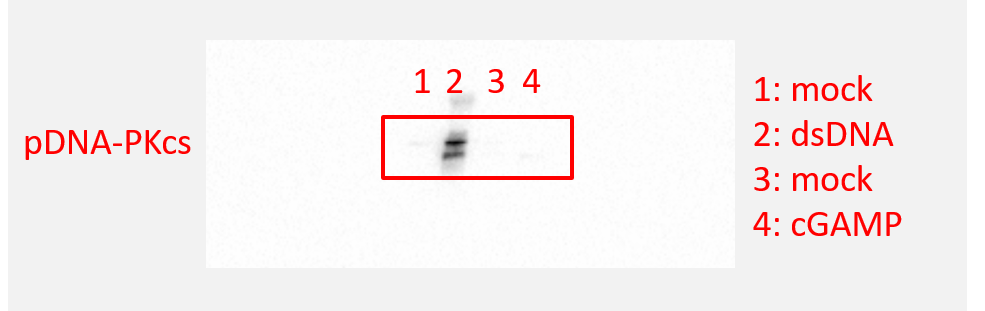

Supplement: Supplementary file 5 — Source Data for Figure 1 [file EMBJ-42-e111961-s009.zip › Figure 1/Figure 1A/Fig1A_western_pDNA-PKcs.tif]

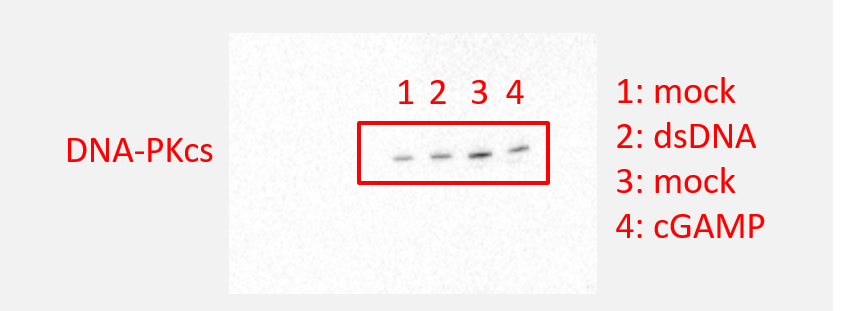

Supplement: Supplementary file 5 — Source Data for Figure 1 [file EMBJ-42-e111961-s009.zip › Figure 1/Figure 1A/Fig1A_western_DNA-PKcs.tif]
