# Supplementary material for: DNA damage repair kinase DNA‐PK and cGAS synergize to induce cancer‐related inflammation in glioblastoma
Source: EMBO J. 2022 Dec 27;42(7):e111961. doi: 10.15252/embj.2022111961 (PMC10068334; doi:10.15252/embj.2022111961)
Supplement: Supplementary file 4 — PDF+ [file EMBJ-42-e111961-s007.pdf]

Article

SOURCE DATA TRANSPARENT PROCESS OPEN ACCESS

THE EMBO JOURNAL

DNA damage repair kinase DNA-PK and cGAS synergize to induce cancer-related inflammation in glioblastoma

Clara Taffoni[1,†], Johanna Marines[1,2,†], Hanane Chamma[1], Soumyabrata Guha[1], Mathilde Saccas[1], Amel Bouzid[1], Ana-Luiza Chaves Valadao[1], Clément Maghe[3,4], Jane Jardine[3,4], Mi Kyung Park[5], Katarzyna Polak[1], Mara De Martino[6], Claire Vanpouille-Box[6], Maguy Del Rio[7], Celine Gongora[7], Julie Gavard[3,4,8], Nicolas Bidère[3,4], Min Sup Song[5], Donovan Pineau[9], Jean-Philippe Hugnot[9], Karima Kissa[10], Laura Fontenille[2], Fabien P Blanchet[11], Isabelle K Vila[1] & Nadine Laguette[1,*]

Abstract

Cytosolic DNA promotes inflammatory responses upon detection by the cyclic GMP-AMP (cGAMP) synthase (cGAS). It has been suggested that cGAS downregulation is an immune escape strategy harnessed by tumor cells. Here, we used glioblastoma cells that show undetectable cGAS levels to address if alternative DNA detection pathways can promote pro-inflammatory signaling. We show that the DNA-PK DNA repair complex (i) drives cGAS-independent IRF3-mediated type I Interferon responses and (ii) that its catalytic activity is required for cGAS-dependent cGAMP production and optimal downstream signaling. We further show that the cooperation between DNA-PK and cGAS favors the expression of chemokines that promote macrophage recruitment in the tumor microenvironment in a glioblastoma model, a process that impairs early tumorigenesis but correlates with poor outcome in glioblastoma patients. Thus, our study supports that cGAS-dependent signaling is acquired during tumorigenesis and that cGAS and DNA-PK activities should be analyzed concertedly to predict the impact of strategies aiming to boost tumor immunogenicity.

Keywords cGAS; DNA-PK; inflammation; tumor immunogenicity
Subject Categories Cancer; Immunology; Signal Transduction
DOI 10.15252/embj.2022111961 | Received 23 June 2022 | Revised 30 November 2022 | Accepted 2 December 2022 | Published online 27 December 2022
The EMBO Journal (2023) 42: e111961

Introduction

Most cells mount type I Interferon (IFN) responses in the presence of cytosolic DNA (Hartmann, 2017). One of the key pathways involved in the detection of immune-stimulatory DNA relies on the cyclic GMP-AMP synthase (cGAS). Upon detection of dsDNA, ssDNA, or RNA:DNA hybrids (Guerra *et al*, 2020, Sun *et al*, 2013), cGAS produces the cyclic GMP-AMP (cGAMP) second messenger. Interaction of cGAMP with the Stimulator of Interferon Genes (STING) promotes the assembly of a signalosome comprised of the Tank Binding Kinase 1 (TBK1) and transcription factors such as Interferon Regulatory Factor 3 (IRF3) (Tanaka & Chen, 2012). TBK1-dependent phosphorylation of IRF3 leads to its nuclear translocation and subsequent activation of transcriptional programs that ultimately lead to the production of inflammatory cytokines, chemokines, and type I IFNs (Ishikawa *et al*, 2009).

The cGAS-STING signaling cascade has been shown to be essential to the orchestration of antitumor responses (Corrales *et al*, 2016, Zhu *et al*, 2019). Indeed, activating the cGAS-STING axis can promote tumor rejection through increasing tumor immunogenicity and priming T cell responses (Sen *et al*, 2019, Sivick *et al*, 2018). However, STING activation can also foster metastatic dissemination (Chen *et al*, 2016) and impair the establishment of durable immunity (Larkin *et al*, 2017). In addition, the cGAS-STING axis has been shown to support the survival of chromosomally unstable cancers (Hong *et al*, 2022), providing an explanation for cGAS-STING

1 IGH, Université de Montpellier, CNRS, Montpellier, France
2 Azelead©, Montpellier, France
3 Team SOAP, CRCI2NA, Nantes Université, Inserm, CNRS, Université d'Angers, Nantes, France
4 Equipe Labellisée Ligue Contre le Cancer, Paris, France
5 Department of Molecular and Cellular Oncology, The University of Texas MD Anderson Cancer Center, Houston, TX, USA
6 Department of Radiation Oncology, Weill Cornell Medicine, New York, NY, USA
7 Institut de Recherche en Cancérologie de Montpellier (IRCM), INSERM, Université de Montpellier, ICM, Montpellier, France
8 Institut de Cancérologie de l'Ouest (ICO), Saint-Herblain, France
9 Institut de Génomique Fonctionnelle (IGF), Université de Montpellier, CNRS, INSERM, Montpellier, France
10 Université de Montpellier, CNRS UMR 5235, Montpellier, France
11 Institut de Recherche en Infectiologie de Montpellier, Université de Montpellier, CNRS, Montpellier, France
*Corresponding author. Tel: +33 434359811; E-mail: nadine.laguette@igh.cnrs.fr
†These authors contributed equally to this work

© 2022 The Authors. Published under the terms of the CC BY 4.0 license.

inactivation in primary tumors (Bakhoum & Cantley, 2018, Bakhoum *et al*, 2018) and activation in late tumorigenesis (Mayca Pozo *et al*, 2021). In addition to these tumor-intrinsic parameters, the diversity of cells composing the tumor microenvironment and their differential expression of cGAS and/or STING are also determinant for tumor fate (Chamma *et al*, 2022a). Prior studies have suggested that downregulation of the cGAS-STING axis is an immune escape strategy exploited by tumor cells (Song *et al*, 2017, Xia *et al*, 2016), despite evidence that high expression of cGAS and/or STING predicts poor outcome for cancer patients (An *et al*, 2019). Reconciling both views likely requires the integration of both tumor-intrinsic and -extrinsic immunogenicity drivers.

Recently, the DNA-dependent protein kinase (DNA-PK) complex, involved in the repair of double-strand DNA lesions by non-homologous end-joining (NHEJ) (Yue *et al*, 2020), has been involved in the detection of DNA virus-derived cytosolic dsDNA, eliciting type I or III IFN responses (Burleigh *et al*, 2020, Ferguson *et al*, 2012, Morchikh *et al*, 2017, Sui *et al*, 2017, Zhang *et al*, 2011). The DNA-PK core complex is a holoenzyme composed of the $KU70^{XRCC6}$ and $KU80^{XRCC5}$ subunits that ensure the recruitment of the DNA-dependent protein kinase catalytic subunit ($DNA\text{-}PKcs^{PRKDC}$) to double-strand breaks (Hammel *et al*, 2010). How cGAS- and DNA-PK-mediated detection of dsDNA are coordinated remains the subject of controversies. Indeed, there are reports indicating that DNA-PKcs inhibits cGAS (Sun *et al*, 2020, Wang *et al*, 2022), while others suggest that DNA-PK may be required for cGAS-STING-dependent inflammatory responses to viral DNA (Morchikh *et al*, 2017, Tao *et al*, 2022). Intriguingly, despite the crucial role of DNA-PK in NHEJ, and the tight link between DNA repair machineries and nucleic acid sensing (Taffoni *et al*, 2021), there is no evidence for a role of DNA-PK in eliciting inflammatory responses following genotoxic stress (Burleigh *et al*, 2020).

Here, we used glioblastoma cells to interrogate how type I IFN responses are initiated in the absence of detectable cGAS. We found that DNA-PK can promote nucleic acid- and chemotherapy-associated inflammatory responses independently of cGAS. Further, we uncover that cGAS and DNA-PK cooperate for optimal STING-dependent signaling, thereby defining tumor immunogenicity. Our work thus suggests that cGAS-dependent signaling is acquired during tumorigenesis.

Results

DNA-PK catalytic activity promotes cytosolic dsDNA-dependent type I Interferon responses in cGAS-deficient cells

Assessment of dsDNA-induced type I IFN responses in the T98G glioblastoma cell line, which does not express detectable cGAS levels (Figs 1A and EV1A), showed increased Interferon β (*IFNB*) and C-X-C motif chemokine ligand 10 (*CXCL10*) Interferon-stimulated gene (ISG) mRNA levels (Fig 1B), attesting to the activation of type I IFN responses. Western blot (WB) analysis further showed that dsDNA stimulation of T98G led to phosphorylation of IRF3 (pIRF3) (Fig 1A). Similar analysis, conducted on $CD133^+$ glioblastoma stem cells (namely, Gli4 and Gli7) (Guichet *et al*, 2013) and patient-derived glioblastoma stem-like cells (GSC4, 6, 9, 13, 15) (Harford-Wright *et al*, 2017), confirmed the absence of cGAS (Figs 1C and EV1B–D) and showed that upon challenge with dsDNA these cells present increased pIRF3 (Figs 1C and EV1B and D), *IFNB* and *CXCL10* (Figs 1D and EV1E and F). In addition, conditioned media from T98G cells treated with dsDNA were sufficient to induce the expression of the Interferon-induced protein with tetratricopeptide repeats (*IFIT*) *1*, *IFIT2*, myxovirus resistance protein 1 (*MXA*) and 2′-5′-Oligoadenylate Synthetase 1 (*OAS1*) ISGs in the THP-1 myeloid cell line (Fig 1E). This attests to the production of bioactive type I IFNs from glioblastoma cells, where cGAS is not detectable, further indicating that glioblastoma cells possess cGAS-independent cytosolic dsDNA detection mechanisms.

Because the DNA-PK complex was previously reported as an alternative cytosolic dsDNA sensor, we interrogated whether DNA-PK could be responsible for the type I IFN response elicited by dsDNA in absence of cGAS. To this aim, we first performed WB analysis using an antibody specific for the auto-phosphorylation of DNA-PKcs on Serine 2056 (pDNA-PKcs), which reflects its activation (Chen *et al*, 2005). Stimulation of T98G, Gli4, Gli7, and GSC9

Figure 1. DNA-PK catalytic activity promotes nucleic acid-dependent type I IFN responses in cGAS-deficient cells.

A T98G cells were challenged or not with dsDNA or with 2′3′cGAMP for 6 h prior to whole cell extraction and Western Blot (WB) analysis using indicated antibodies.
B *IFNB* and *CXCL10* mRNA levels were analyzed by RT–qPCR in samples treated as in A ($n = 3$ independent experiments).
C Gli4 cells were treated as in A prior to WB analysis using indicated antibodies.
D *IFNB* and *CXCL10* mRNA levels were analyzed by RT–qPCR in samples treated as in C ($n = 3$ independent experiments).
E Phorbol 12-myristate 13-acetate (PMA)-treated THP1 cells were incubated for 24 h with conditioned media derived from T98G cells treated or not with dsDNA. *IFIT1*, *IFIT2*, *MXA*, *OAS1* mRNA levels were analyzed by RT–qPCR. Graphs present a representative biological triplicate ($n = 3$ independent experiments).
F T98G cells were challenged or not with dsDNA for 6 h, in the presence or not of the NU7441 DNA-PKcs inhibitor, prior WB analysis using indicated antibodies.
G *IFNB* and *CXCL10* mRNA levels were analyzed by RT–qPCR in samples treated as in F ($n = 4$ independent experiments).
H T98G cells were treated with non-targeting (CTRL) or DNA-PKcs-targeting siRNAs for 72 h prior to 6 h challenge with dsDNA. *IFNB* and *CXCL10* mRNA levels were analyzed by RT–qPCR. Graphs present a representative biological triplicate ($n = 3$ independent experiments).
I T98G cells were treated or not with 0.16 μM camptothecin (CPT) for 48 h prior to immunofluorescence analysis using dsDNA- and 53BP1-specific antibodies, and DAPI nuclear staining ($n = 3$ independent experiments). Scale bar, 20 μm.
J Whole cell extracts from T98G cells treated or not for 72 h with 0.16 μM CPT were analyzed by WB using indicated antibodies.
K *IFNB* and *CXCL10* mRNA levels were analyzed by RT–qPCR in samples treated as in J ($n = 3$ independent experiments).
L T98G cells were treated or not with 0.16 μM CPT for 72 h, in presence or not of NU7441, prior to assessment of *IFNB* and *CXCL10* mRNA levels by RT–qPCR. Graphs present a representative biological triplicate ($n = 3$ independent experiments).

Data information: All immunoblots are representative experiments ($n = 3$ independent experiments). All graphs present means ± standard error from the mean (SEM). *P*-values were determined by Student's *t*-test. ns: not significant. $^*P < 0.05$, $^{**}P < 0.01$, $^{***}P < 0.001$, $^{****}P < 0.0001$.
Source data are available online for this figure.

 © 2022 The Authors

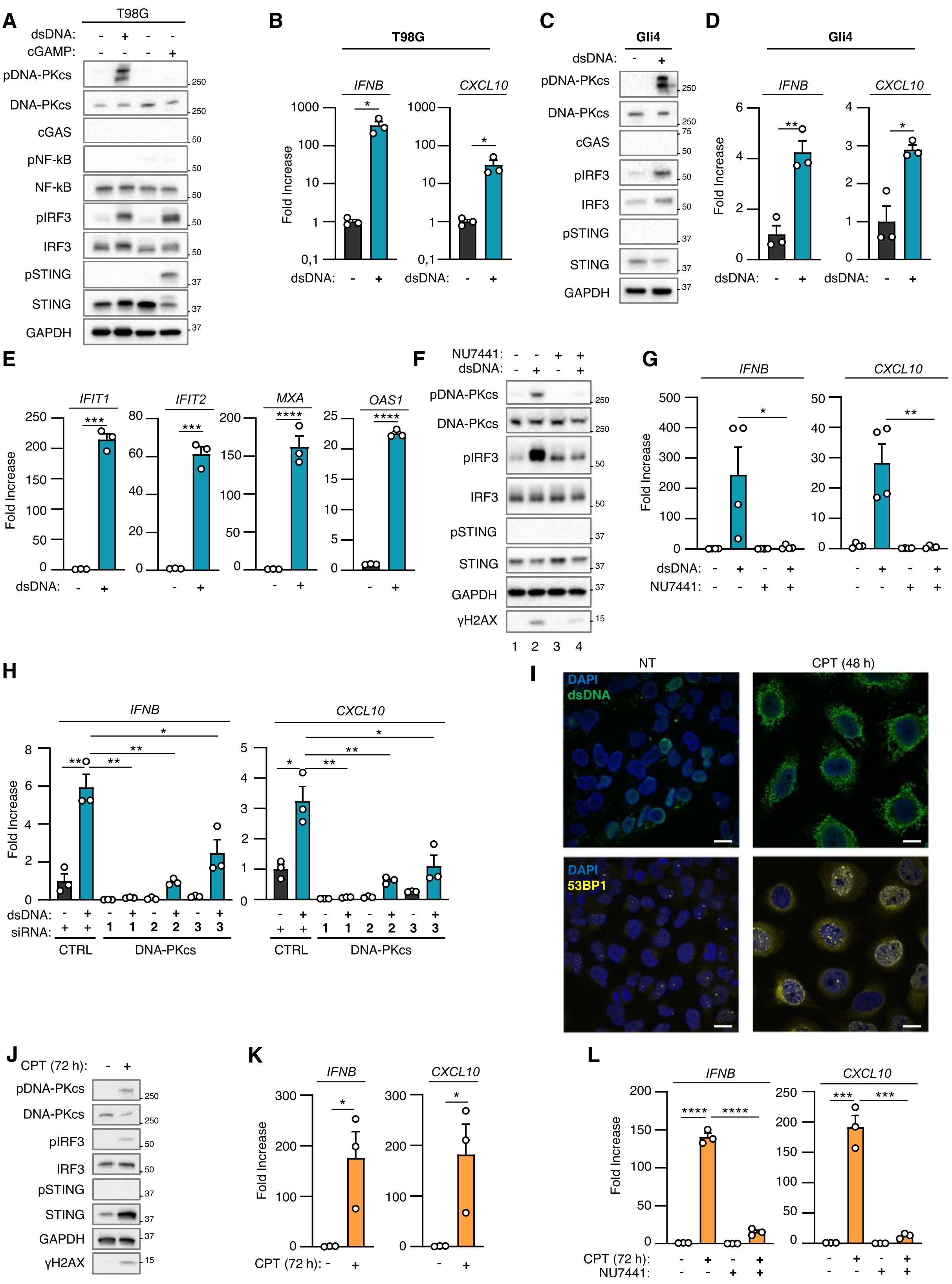
A
T98G
dsDNA:
cGAMP:
pDNA-PKcs
DNA-PKcs
cGAS
pNF-kB
NF-kB
pIRF3
IRF3
pSTING
STING
GAPDH
B
T98G
IFNB
CXCL10
Fold Increase
dsDNA:
C
Gli4
D
Gli4
E
IFIT1
IFIT2
MXA
OAS1
F
NU7441:
dsDNA:
γH2AX
G
IFNB
CXCL10
H
IFNB
CXCL10
siRNA:
CTRL
DNA-PKcs
I
NT
CPT (48 h)
DAPI
dsDNA
DAPI
53BP1
J
CPT (72 h):
K
IFNB
CXCL10
CPT (72 h):
L
IFNB
CXCL10
CPT (72 h):
NU7441:

Figure 1.

with dsDNA led to DNA-PKcs phosphorylation (Figs 1A and C, and EV1B and D). Second, we tested whether DNA-PK is responsible for type I IFN responses in the absence of cGAS in glioblastoma cells by treating T98G, Gli4, and GSC9 with the NU7441 DNA-PKcs inhibitor. WB analysis showed that treatment with NU7441 inhibited dsDNA-induced DNA-PKcs auto-phosphorylation and decreased phosphorylation of the H2AX DNA-PK substrate (Figs 1F and EV1G compare lanes 2 and 4), attesting to efficient DNA-PKcs inhibition. NU7441 treatment also led to a decrease of pIRF3 (Figs 1F and EV1G), *IFNB* and *CXCL10* levels (Figs 1G and EV1F and H). Finally, T98G cells were treated with scrambled or DNA-PKcs-targeting siRNAs prior to analysis of dsDNA-dependent type I IFN responses. Knock-down of DNA-PKcs in T98G cells (Fig EV1I) abrogated dsDNA-induced type I IFN responses (Fig 1H). Thus, DNA-PKcs drives dsDNA-induced type I IFN responses in glioblastoma cells.

DNA-PK controls genotoxic stress-induced type I Interferon responses in absence of cGAS

Previous work has shown that in myeloid cells, DNA-PKcs does not induce genotoxic stress-associated type I IFN responses (Burleigh *et al*, 2020). Here, we questioned whether DNA-PK may be involved in genotoxic stress-associated type I IFN responses in cancer cells that do not express detectable cGAS levels. To this aim, T98G cells were treated with the camptothecin genotoxic agent to induce dsDNA breaks that are primarily repaired by NHEJ (Adachi *et al*, 2004). Staining with a dsDNA-specific antibody showed that such treatment is sufficient to induce cytosolic accumulation of dsDNA (Fig 1J, upper panels) together with accumulation of 53BP1 foci in the nucleus and in the cytosol, reflecting defective repair and accumulation of DNA lesions (Fig 1I, lower panels) (Gonzalez-Suarez *et al*, 2011). WB analysis showed that camptothecin treatment led to increased pDNA-PKcs, and pIRF3, but not pSTING (Fig 1J), together with increased levels of *IFNB* and *CXCL10* (Fig 1K). This suggests that DNA-PK may be responsible for cGAS-independent type I IFN responses following genotoxic stress in T98G cells.

We next treated T98G cells with camptothecin in the presence of NU7441. Since NU7441 inhibits the catalytic activity of DNA-PKcs, as reflected by decreased pDNA-PKcs (Fig EV1J), such treatment presumably impacts both DNA-PK-mediated DNA repair and signaling. Consequently, treatment with NU7441 alone was sufficient to promote the accumulation of DNA damage, as confirmed by the presence of 53BP1 foci and of cytosolic dsDNA (Fig EV1K), without triggering type I IFN responses, as attested by the absence of *IFNB* and *CXCL10* upregulation (Fig 1L). However, treatment with NU7441 abrogated camptothecin-associated *IFNB* and *CXCL10* induction (Fig 1L). Similarly, we found that NU7441 inhibited type I IFN responses elicited by treatment with the etoposide genotoxic agent (Fig EV1L). Thus, altogether, these data support that DNA-PKcs is responsible for genotoxic stress-dependent activation of type I IFN responses in T98G cells.

DNA-PK-dependent detection of cytosolic dsDNA drives STING-independent IRF3-dependent type I IFN responses in cancer cells lacking cGAS

We next questioned the molecular mechanisms involved in DNA-PK-dependent type I IFN responses following challenge with exogenous dsDNA or genotoxic stress. First, immunofluorescence analysis showed that, following dsDNA challenge in T98G and Gli4 cells, activated DNA-PKcs is found in the cytosol (Figs 2A and EV2A), suggesting DNA-PKcs translocation into the cytosol, as previously reported upon UV treatment (Tu *et al*, 2013). Second, we assessed the ability of DNA-PK to interact with cytosolic dsDNA. To this aim, we used 80 nt-long dsDNA or ssDNA, bearing a 5′ biotin on the sense strand to perform streptavidin-affinity pull-down experiments using whole cell extracts from T98G cells. We thereby observed that DNA-PKcs, KU80, and KU70 are recruited to dsDNA (Fig 2B). Similar experiments were performed following 6 h of biotinylated dsDNA transfection in the THP-1 human myeloid cell line (Fig EV2B), a time point at which transfected dsDNA are found in the cytosol (Guerra *et al*, 2020). In these conditions, together with DNA-PKcs, KU70, and KU80, recruitment of pDNA-PKcs to dsDNA was also observed (Fig EV2B). Combined, these experiments support that DNA-PK is recruited to cytosolic dsDNA.

We next tested whether the interaction of KU70:KU80 with dsDNA was required for DNA-PKcs-dependent type I IFN responses. To this aim, we performed KU70 knock-down (Fig 2C), prior to

Figure 2. DNA-PK-dependent detection of cytosolic dsDNA drives STING-independent IRF3-dependent type I IFN responses in cancer cells lacking cGAS.

A T98G cells were treated or not with dsDNA for 6 h prior to immunofluorescence analysis using DNA-PKcs- and pDNA-PKcs-specific antibodies, and DAPI nuclear staining ($n = 3$ independent experiments). Scale bar, 20 µm.
B Whole cell extracts from T98G cells were incubated with 80 nt-long biotinylated ssDNA or dsDNA prior to pull-down using streptavidin-affinity beads. Input and eluates were analyzed by WB using indicated antibodies.
C T98G cells were treated with non-targeting (CTRL) or KU70-targeting siRNAs prior to whole cell extract preparation, and pull-down as in (B). Inputs and eluates were analyzed by WB using indicated antibodies.
D T98G cells were treated with CTRL or KU70-targeting siRNAs prior to challenge with dsDNA for 6 h. *IFNB* and *CXCL10* mRNA levels were analyzed by RT–qPCR. Graphs present a representative biological triplicate ($n = 3$ independent experiments).
E CTRL or *STING*$^{-/-}$ T98G cells were transfected or not with dsDNA for 8 h prior to analysis of *IFNB* and *CXCL10* mRNA levels ($n = 3$ independent experiments).
F As in (E), except that CTRL or *IRF3*$^{-/-}$ T98G cells were transfected or not with dsDNA for 6 h prior to analysis ($n = 3$ independent experiments).
G CTRL or *STING*$^{-/-}$ T98G cells were treated or not with CPT for 72 h prior to analyses of *IFNB* and *CXCL10* mRNA levels ($n = 3$ independent experiments).
H As in (G), except that CTRL and *IRF3*$^{-/-}$ T98G cells were used. Graphs present a representative biological triplicate ($n = 3$ independent experiments).
I Schematic representation of cytosolic dsDNA-dependent type I IFN induction in T98G cells.

Data information: All immunoblots are representative experiments ($n = 3$ independent experiments). All graphs present means ± SEM. *P*-values were determined by Student's *t*-test. ns: not significant. $^{**}P < 0.01$, $^{***}P < 0.001$, $^{****}P < 0.0001$.
Source data are available online for this figure.

© 2022 The Authors

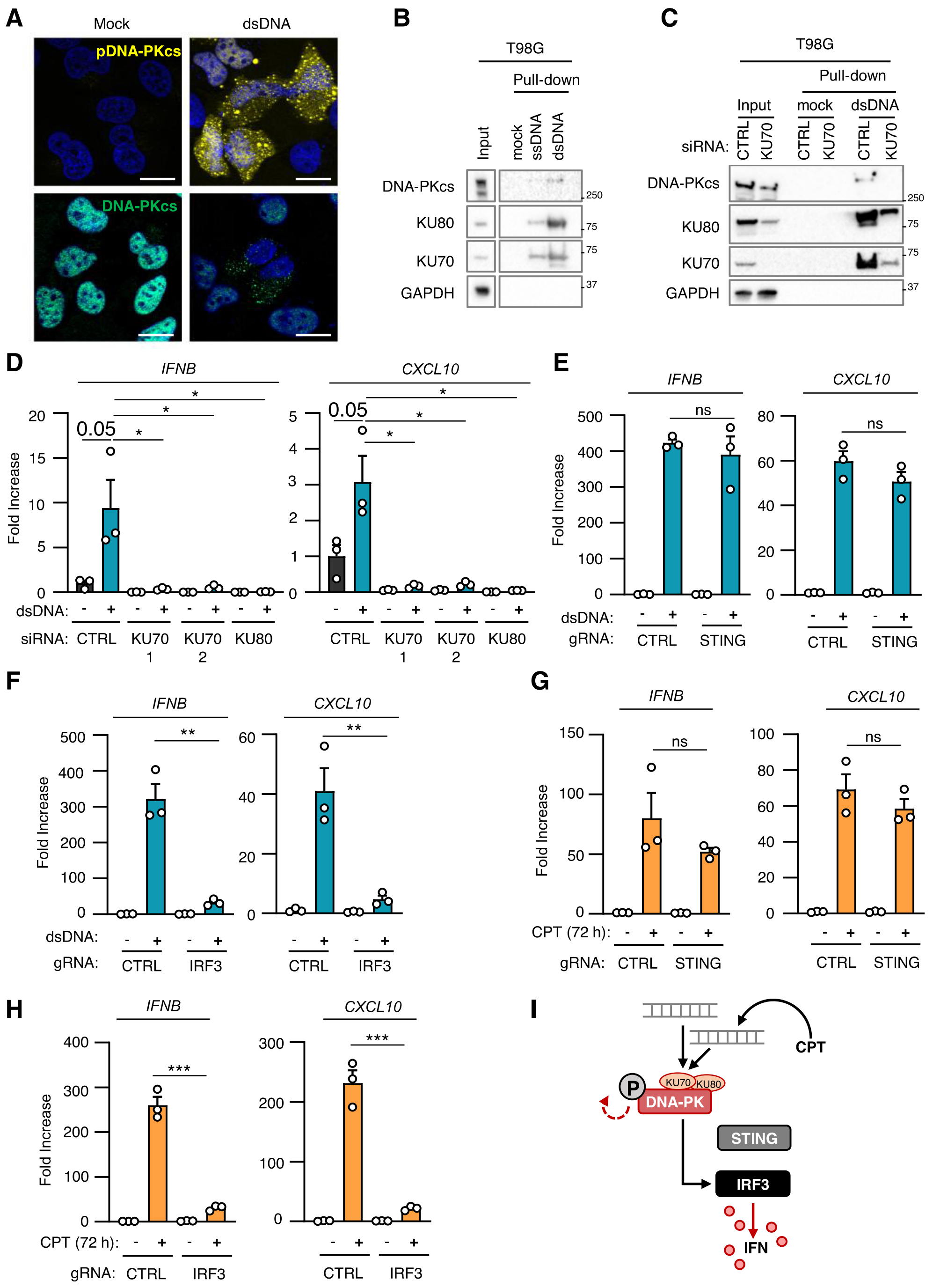

Figure 2.

assessment of the recruitment of DNA-PK to dsDNA. Knock-down of KU70 decreased the recruitment of KU80 while abolishing the recruitment of DNA-PKcs to dsDNA (Fig 2C). Of note, the total protein levels of KU80 and DNA-PKcs were also decreased following KU70 knock-down, likely reflecting destabilization of DNA-PK complexes following KU70 depletion as previously reported (Gu *et al*, 1997, Nussenzweig *et al*, 1996). Conversely, performing dsDNA pull-down using whole cell extracts from control ($HCT116^{CTRL}$) and DNA-PKcs-deficient HCT116 ($HCT116^{PRKDC-/-}$) cells showed that DNA-PKcs is not required for the recruitment of KU70 and KU80 to dsDNA (Fig EV2C). Importantly, knock-down of KU70 and KU80 (Fig EV2D) also abolished dsDNA-induced *IFNB* and *CXCL10* expression in T98G cells (Fig 2D). Thus, these data demonstrate that the recruitment of DNA-PKcs to cytosolic dsDNA through the KU70:KU80 heterodimer is required to trigger type I IFN responses.

WB analyses of STING protein levels and phosphorylation status in glioblastoma cells (Figs 1 and EV1) suggest that the DNA-PK-dependent type I IFN responses elicited by dsDNA transfection are STING-independent. To formally test the requirement for STING, we

generated STING-deficient T98G cells (T98G$^{STING-/-}$) (Fig EV2E and F) where we assessed dsDNA-induced type I IFN responses (Figs 2E and EV2G). Challenge with dsDNA of T98G$^{STING-/-}$ and of their control counterparts (T98G^{CTRL}) promoted IRF3 phosphorylation (Fig EV2G), together with *IFNB* and *CXCL10* upregulation (Fig 2E), regardless of STING expression. The presence of phosphorylated IRF3 following dsDNA challenge in absence of detectable cGAS (Figs 1A and C, and EV1B and D) also suggested that DNA-PK-associated IFN responses require IRF3. To confirm this requirement, IRF3-deficient T98G cells (T98G$^{IRF3-/-}$) (Fig EV2H) were challenged with dsDNA prior to assessment of type I IFN responses. Absence of IRF3 disrupted type I IFN responses in T98G cells (Figs 2F and EV2H), supporting that DNA-PK-dependent, STING-independent, type I IFN responses require IRF3 in cancer cells presenting undetectable cGAS levels.

We next analyzed whether genotoxic stress-induced DNA-PK-dependent type I IFN responses in absence of detectable cGAS levels are governed by similar molecular mechanisms. To this aim, T98G^{CTRL}, T98G$^{STING-/-}$, and T98G$^{IRF3-/-}$ were treated with camptothecin prior to analysis of DNA-PK activation and IFN responses. Similar to what was observed upon challenge with dsDNA, genotoxic stress induced DNA-PKcs phosphorylation, regardless of the expression of STING and IRF3 (Fig EV2I and J). In addition, while induction of type I IFN responses did not require the expression of STING (Fig 2G), the presence of IRF3 was required (Fig 2H). Thus, DNA-PKcs controls genotoxic stress-induced type I IFN responses through IRF3 activation.

Finally, we questioned whether cGAS-independent DNA-PK-associated type I IFN responses can be observed in other cell lines. To this aim, we used cGAS-knockout THP-1 cells (THP-1$^{cGAS-/-}$) and observed that, challenged with dsDNA, induced type I IFN responses (Fig EV2K and L), concomitantly to DNA-PKcs phosphorylation (Fig EV2K). However, STING ablation abolished type I IFN responses in THP-1 cells (Fig EV2K and L). Thus, in contrast to T98G cells, STING is indispensable for type I IFN responses in myeloid cells, suggesting that the requirement for STING in DNA-PK activation is cell type specific. Thus DNA-PKcs drives cytosolic dsDNA-IFN responses through IRF3 (Fig 2I).

cGAS and DNA-PKcs cooperate for optimal dsDNA-induced type I Interferon responses

Considering that DNA-PK can elicit type I IFN responses in absence of cGAS, we next interrogated the impact of co-expressing DNA-PKcs and cGAS in glioblastoma cells. To this aim, we generated T98G cells stably expressing cGAS (T98G^{cGAS}) or not (T98G^{Empty}) (Fig 3A). Expression of cGAS was sufficient to induce constitutive degradation of STING (Fig 3A, compare lanes 3 to 1) and to promote increased basal *IFNB* and *CXCL10* levels (Fig 3B). Additionally, challenge with dsDNA led to enhanced pIRF3 levels together with increased induction of type I IFN responses (Fig 3A and B), indicating that the cGAS-STING axis is efficiently restored in T98G^{cGAS} cells. Similar to what was observed in T98G cells, dsDNA transfection induced cytosolic accumulation of pDNA-PKcs (Fig EV3A). Knowing the strong affinity of cGAS for cytosolic dsDNA (Zhou *et al*, 2018), we addressed whether DNA-PK and cGAS can compete for dsDNA detection, by transfecting biotinylated dsDNA in T98G^{Empty} and T98G^{cGAS} or in THP-1^{CTRL} and THP-1$^{cGAS-/-}$ prior to streptavidin-affinity pull-down. WB analysis showed that the recruitment of subunits of DNA-PK to dsDNA is not altered in the presence of cGAS, except an increase of KU70 associated with dsDNA in T98G^{Empty} as compared to T98G^{cGAS} (Figs 3C and EV3B). Next, we transfected limiting amounts of dsDNA in T98G^{Empty} and T98G^{cGAS} prior to pull-down and assessment of the binding of DNA-PK subunits. Although increasing amounts of dsDNA led to increased cGAS recruitment to dsDNA, recruitment of DNA-PKcs and KU70 appeared unaltered. To the contrary, KU80 binding to pulled-down dsDNA appeared to increase with lower levels of dsDNA (Fig EV3C). Yet, to activate DNA-PKcs-dependent phosphorylation, subunits of DNA-PK work as an heterotrimer in which

Figure 3. cGAS and DNA-PKcs cooperate for optimal dsDNA-induced type I Interferon responses.

A T98G cells stably expressing cGAS (T98G^{cGAS}) or not (T98G^{Empty}) were transfected or not with dsDNA for 6 h prior to whole cell extraction and WB analysis using indicated antibodies.
B *IFNB* and *CXCL10* mRNA levels were analyzed by RT–qPCR in samples treated as in (A). Graphs present a representative biological triplicate ($n = 5$ independent experiments).
C T98G^{Empty} and T98G^{cGAS} were transfected or not with biotinylated dsDNA prior to whole cell extraction and pull-down using streptavidin-affinity beads. Inputs and eluates were analyzed by WB using indicated antibodies.
D T98G^{Empty} and T98G^{cGAS} were transfected or not with dsDNA for 6 h in the presence or not of NU7441 prior to *IFNB* and *CXCL10* expression analysis. Graphs present a representative biological triplicate ($n = 5$ independent experiments).
E T98G^{Empty} and T98G^{cGAS} were treated or not with CPT for 72 h in combination or not with NU7441 (48 h) prior to *IFNB* and *CXCL10* expression analysis. Graphs represent a biological triplicate ($n = 4$ independent experiments).
F THP-1^{CTRL} and THP-1$^{cGAS-/-}$ were transfected or not with dsDNA for 6 h in presence or not of NU7441 prior to *IFNB* and *CXCL10* expression analysis ($n = 3$ independent experiments).
G Intracellular cGAMP levels were analyzed in samples treated as in (E) by ELISA ($n = 3$ independent experiments).
H Intracellular cGAMP levels were analyzed in samples treated as in (F) by ELISA. Graphs present a representative biological triplicate ($n = 2$ independent experiments).
I T98G expressing a catalytic dead cGAS allele (T98G$^{cGAS\text{-}CD}$) and T98G were treated as in (A) prior to *IFNB* and *CXCL10* levels analysis. Graphs present a representative biological triplicate ($n = 3$ independent experiments).
J T98G^{cGAS} were transfected or not with dsDNA in presence or not of NU7441, prior to WB analysis using indicated antibodies.
K THP-1 were transfected or not with dsDNA in presence or not of NU7441, prior to WB analysis using indicated antibodies.
L Schematic representation of the molecular mechanisms involved in the cooperation between DNA-PKcs and cGAS for type I IFN induction.

Data information: All immunoblots are representative experiments ($n = 3$ independent experiments). All graphs present means ± SEM. *P*-values were determined by Student's *t*-test. ns: not significant. $^{*}P < 0.05$, $^{**}P < 0.01$, $^{***}P < 0.001$, $^{****}P < 0.0001$.
Source data are available online for this figure.

© 2022 The Authors

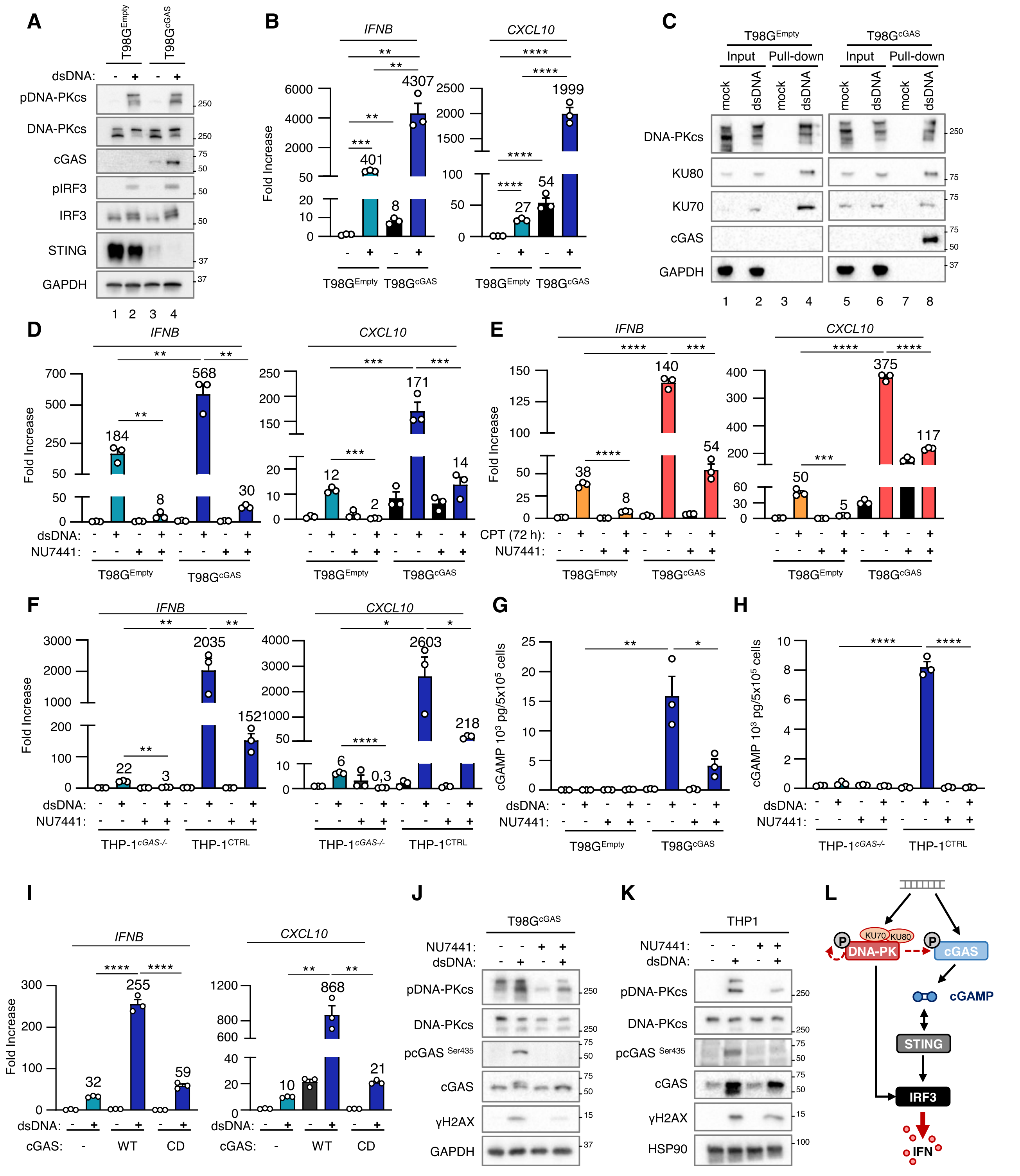

Figure 3.

DNA-PKcs is the limiting factor (Hammarsten & Chu, 1998, West *et al*, 1998). Thus, expressing cGAS in T98G cells restores the cGAS-STING signaling axis without modifying the interaction of DNA-PK with dsDNA ligands.

Given that both cGAS and DNA-PK can detect cytosolic DNA when co-expressed, we next questioned their respective contribution to dsDNA-dependent induction of type I IFN responses. To this aim, T98G^{Empty} and T98G^{cGAS} were either transfected with dsDNA

or treated with camptothecin, in the presence or not of NU7441, prior to evaluation of type I IFN responses. Intriguingly, treatment with NU7441 led to a dramatic decrease of *IFNB* and *CXCL10* expression, both in the presence and absence of cGAS, following dsDNA transfection (Figs 3D and EV3D). Similarly, treatment with camptothecin led to higher type I IFN responses in the presence of cGAS, which were abolished by treatment with NU7441 (Fig 3E). This suggested that DNA-PK and cGAS cooperate for the induction of type I IFN responses, when co-expressed. We interrogated whether a similar cooperation could be witnessed in other cell lines, using THP-1, but also the CFPAC pancreatic cancer cell line, and their $cGAS^{-/-}$ counterparts. Treatment with NU7441 also led to a dramatic decrease of type I IFN responses to dsDNA in both cell types, regardless of the expression of cGAS (Figs 3F and EV3E–G). We next assessed whether the cooperation between cGAS and DNA-PK is operational in murine cell lines. To this aim, mouse embryonic fibroblast (MEF) and their *cGas*-deficient counterpart, as well as the GL261 murine glioblastoma cell line knockout or not for *cGas* were transfected or not with dsDNA in the presence or not of NU7441. We thereby observed that in these murine cell lines, type I IFN responses are potentialized when cGAS and DNA-PK are both functional (Fig EV3H–J). Thus, our data suggest that the mechanism through which DNA-PK and cGAS synergize is conserved in murine models.

To identify the molecular mechanism through which cGAS and DNA-PK cooperate, we tested whether DNA-PK can control cGAS activity. To this aim, we quantified intracellular cGAMP levels in $T98G^{Empty}$ and $T98G^{cGAS}$, but also in $THP\text{-}1^{CTRL}$ and $THP\text{-}1^{cGAS-/-}$ cells, upon challenge with dsDNA in the presence or not of NU7441. Treatment with NU7441 led to a decrease of cGAMP levels in both $T98G^{cGAS}$ and THP-1 cells (Fig 3G and H), although NU7441 did not alter cGAS activity *in vitro* (Fig EV3K). Finally, when T98G stably expressing a catalytic-dead cGAS allele ($T98G^{cGAS\text{-}CD}$) were challenged with dsDNA, type I IFN responses were drastically reduced as compared to those witnessed in $T98G^{cGAS}$ (Figs 3I and EV3L). These data show that the cooperation between cGAS and DNA-PK operates at the level of cGAS activity, and strongly suggest that DNA-PKcs boosts the production of cGAMP by cGAS.

Phosphorylation of the Serine 435 (Ser435) of cGAS has been previously shown to be required to enable cGAS-dependent cGAMP production (Li & Shu, 2020). Because DNA-PKcs bears a Serine/Threonine kinase activity, we asked whether DNA-PKcs could be responsible for this phosphorylation. Challenge with dsDNA of cGAS-proficient cells (namely $T98G^{cGAS}$, THP-1, and CFPAC) showed that the phosphorylation of cGAS on Ser435 was lost upon DNA-PKcs inhibition by NU7441 (Figs 3J and K and EV3F). This supports that DNA-PKcs catalytic activity is required for cGAS phosphorylation at Ser435. Thus, altogether these data show that DNA-PKcs is required for efficient cGAS-dependent cGAMP production (Fig 3L).

cGAS re-expression in glioblastoma cancer cells promotes macrophage recruitment and impairs tumorigenesis

We next questioned whether the synergy between cGAS and DNA-PK could have an impact on glioblastoma tumor immunogenicity. First, because cGAS has been previously shown to alter proliferation (Wang *et al*, 2015, Yang *et al*, 2017), we assessed the growth of $T98G^{Empty}$ and $T98G^{cGAS}$ in 2D and 3D cultures. Follow-up over time showed that cGAS re-expression did not alter the proliferation of T98G cells (Fig 4A and B), nor affected their ability to form spheroids (Fig 4C), ruling out cell-intrinsic defects. However, upon subcutaneous engraftment in nude mice, $T98G^{cGAS}$ failed to form tumors (Fig 4D and E). To visualize early steps of tumorigenesis, we next used zebrafish embryos in which we performed orthotopic transplantation of $T98G^{Empty}$ and $T98G^{cGAS}$ stably expressing a green fluorescence protein (GFP) reporter ($T98G\text{-}GFP^{Empty}$ and $T98G\text{-}GFP^{cGAS}$). Monitoring of the intracranial GFP signal over time showed a faster decrease of the $T98G\text{-}GFP^{cGAS}$ tumor mass, as compared to $T98G\text{-}GFP^{Empty}$ tumors (Fig 4F). Moreover, morphological assessment of tumors showed more elongated pseudopodia, which are hallmarks of invasiveness (Lah *et al*, 2020), in $T98G\text{-}GFP^{Empty}$ tumors as compared to $T98G\text{-}GFP^{cGAS}$ (Fig EV4A and B). Thus, cGAS expression in T98G cells is sufficient to impair early tumorigenesis.

Since nude mice and zebrafish embryos, at the stage at which they were engrafted, do not possess an adaptive immune system, we next hypothesized that differential cytokine and chemokine production may modulate myeloid cell activity in the glioblastoma microenvironment. Migration assays showed that conditioned media from $T98G^{cGAS}$ cells increased the migration of THP-1 cells as compared to that from $T98G^{Empty}$ cells (Fig 5A). However, conditioned media from $T98G^{cGAS}$ cells was not sufficient to promote THP-1 and primary myeloid cell polarization (Fig EV5A and B). Thus, soluble factors secreted by $T98G^{cGAS}$ are sufficient to promote the recruitment of myeloid cells to the tumor mass, but not their polarization. To identify those soluble factors, we profiled cytokines and chemokines levels, using the proteome profiler qualitative array, in the supernatant of $T98G^{Empty}$ and $T98G^{cGAS}$, thereby identifying C-C Motif Chemokine Ligand 2 (CCL2) and 5 (CCL5), in addition to CXCL10, as most upregulated in the supernatant of $T98G^{cGAS}$ as compared to $T98G^{Empty}$ cells (Fig 5B). Such upregulation was also observed at the gene expression level (Fig 5C) and was lost when comparing $T98G^{cGAS}$ to $T98G^{cGAS\text{-}CD}$ (Fig EV5C). To assess whether DNA-PK and cGAS synergize to induce the expression of these genes, we next assessed the expression of *CCL2* and *CCL5* upon dsDNA stimulation in the presence or not of NU7441. Such analyses revealed that while NU7441 treatment abolished the expression of *CCL2* in the presence or absence of cGAS, it did not abolish cGAS-induced *CCL5* expression (Fig EV5D). Interestingly, we also observed that DNA-PK and cGAS appear to synergize to induce the expression of type III IFNs (*IFNL2/3*), *CCL3* and interleukin 6 (*IL-6*) (Fig EV5E). This suggests that while cGAS and DNA-PK synergize to induce the expression of certain genes, there are likely additional parameters controlling gene activation downstream of these two receptors. Furthermore, these data support that the synergy between DNA-PK and cGAS promotes the production of chemokines that can induce macrophage recruitment.

We thus took advantage of the optical traceability of macrophages in the *tg(mfap4:RFP)* zebrafish line, owing to the expression of a red fluorescent protein (RFP) reporter under the control of the *mfap4* promoter. Imaging over time showed enhanced recruitment of myeloid cells around and inside tumors of zebrafish embryos injected with $T98G\text{-}GFP^{cGAS}$, as compared to those injected with

 © 2022 The Authors

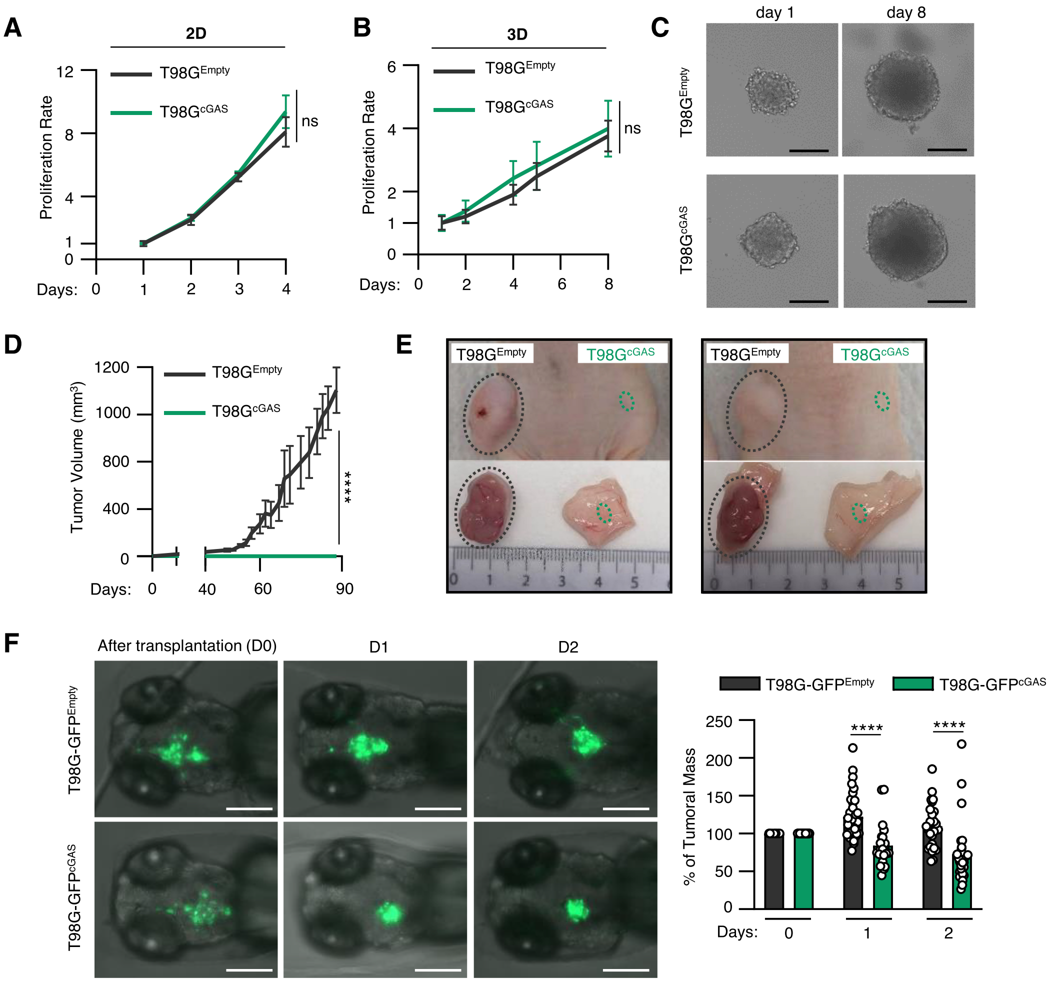

Figure 4. cGAS re-expression in glioblastoma cancer cells impairs tumorigenesis.

A The proliferation of T98G^{Empty} and T98G^{cGAS} was monitored in 2D cultures over 4 days ($n = 3$ independent experiments).
B The volume of spheroids formed by T98G^{Empty} and T98G^{cGAS} was monitored over 8 days ($n = 3$ independent experiments).
C Representative images of T98G^{Empty} and T98G^{cGAS} spheroids measured in (B), at day 1 and day 8. Scale bar, 250 μm.
D The volume of subcutaneous T98G^{Empty} and T98G^{cGAS} tumors in nude mice was measured every 3–4 days by caliper ($n = 6$ mice per group).
E Representative pictures of T98G^{Empty} and T98G^{cGAS} tumors from D, at day 90 post subcutaneous engraftment.
F T98G^{Empty} and T98G^{cGAS} stably expressing a GFP reporter (T98G-GFP^{Empty} and T98G-GFP^{cGAS}, respectively) were xenotransplanted into the head of *tg(mfap4:RFP)* zebrafish line at 3 days post fertilization (dpf). Zebrafish embryos were imaged daily over 3 days. The graph represents the mean (± SEM) percentage of tumor growth normalized by the area on the day of transplantation ($n = 21$ T98G-GFP^{Empty} and $n = 29$ T98G-GFP^{cGAS} embryos). Scale bar, 200 μm.

Data information: All graphs present means ± SEM. *P*-values were determined by Student's *t*-test. ns: not significant. *$P < 0.05$, **$P < 0.01$, ****$P < 0.0001$. One-way Anova with Tukey's multiple comparisons test was used for mice analyses. Mann–Withney test was performed to analyze tumor growth in zebrafish.
Source data are available online for this figure.

T98G-GFP^{Empty} (Fig 5D). Quantification of contacts between tumors and myeloid cells further supported increased recruitment and interaction between tumor masses formed of T98G-GFP^{cGAS} and myeloid cells, as compared to T98G-GFP^{Empty} tumor masses (Fig 5D and E). In addition, assessment of macrophage polarization markers showed increased expression of M1 markers in zebrafish heads where T98G-GFP^{cGAS} were injected, as compared to T98G-GFP^{Empty} tumors (Fig 5F). Thus, these data suggest that cGAS re-expression in T98G cells may be sufficient to promote macrophage recruitment and M1 polarization at the tumor site, impairing tumor engraftment and promoting tumor clearance.

cGAS and DNA-PKcs levels increase with tumor grade

To confirm the physiological relevance of our findings that indicate that cGAS expression impairs early tumorigenesis, we first performed a meta-analysis of glioblastoma tumors, using the GlioVis database (Bowman *et al*, 2017). We examined the co-expression of *PRKDC* and *MB21D1* (encoding cGAS) in glioblastoma tissue samples from the CGGA transcriptomic database. Of the 224 retrieved cases, three statistically well-represented populations were determined based on *PRKDC* and *MB21D1* expression levels: *PRKDC*^{low}/*MB21D1*^{low}, *PRKDC*^{high}/*MB21D1*^{low}, and *PRKDC*^{high}/*MB21D1*^{high}

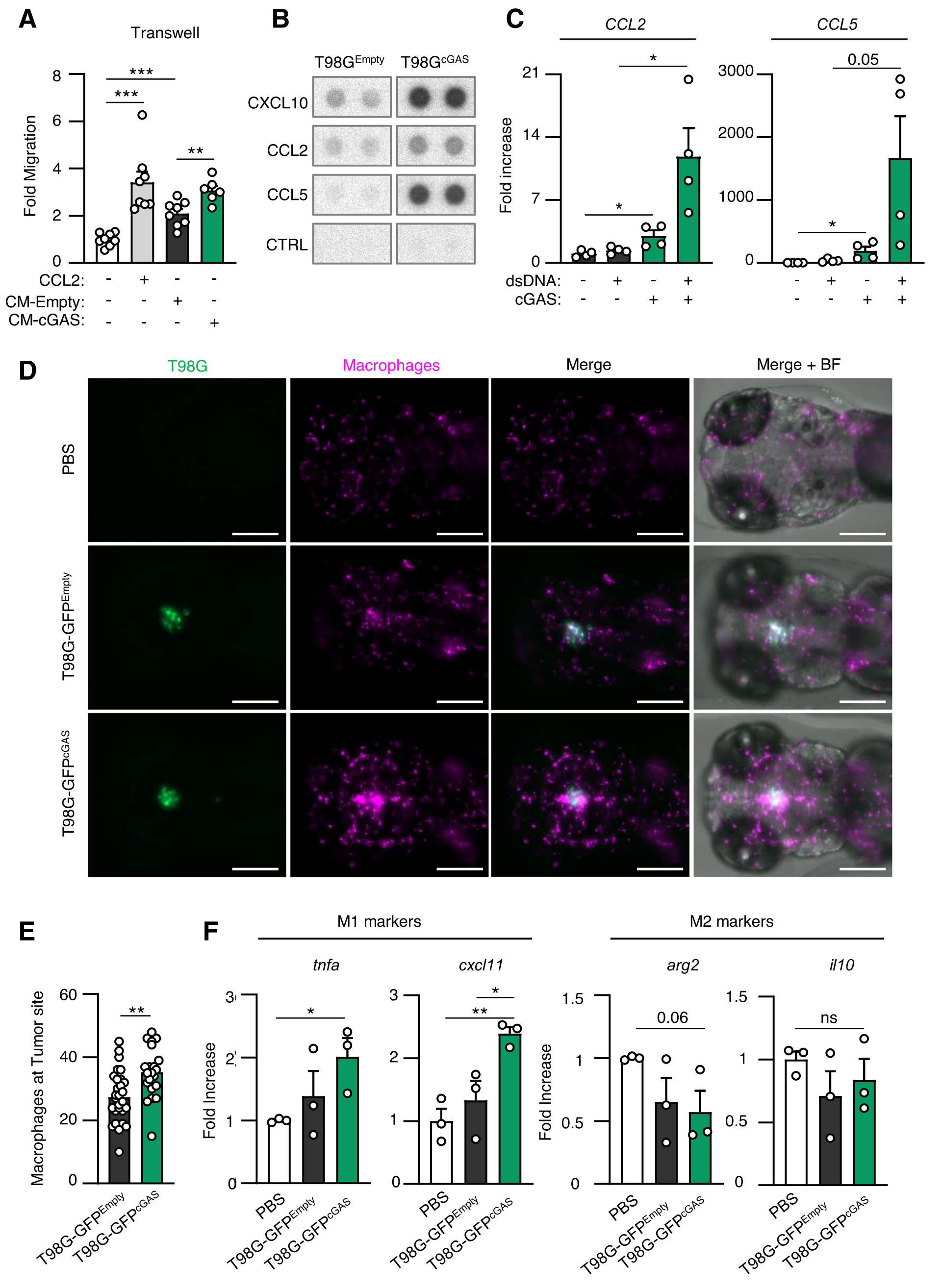

Figure 5. cGAS re-expression in glioblastoma cancer cells promotes macrophage recruitment.

A Graph represents the mean (± SEM) fold migration of THP-1 cells through a 3 μm transwell insert when conditioned media from T98G^{Empty} or T98G^{cGAS} was applied to lower chamber for 6 h. CCL2 was used as positive control (*n* = 8 biological replicates).

B CXCL10, CCL2 and CCL5 protein levels in conditioned media from T98G^{Empty} and T98G^{cGAS} cells were assessed using proteome profiler. Proteins that were found to be the most upregulated in T98G^{cGAS} are shown. Representative immunoblots (*n* = 3 independent experiments).

C T98G^{Empty} and T98G^{cGAS} were transfected or not with dsDNA prior to analyses of *CCL2* and *CCL5* by RT–qPCR (*n* = 4 independent experiments).

D Zebrafish embryos injected with T98G-GFP^{Empty}, T98G-GFP^{cGAS}, or PBS at 3 dpf (Fig 4E) were imaged at 24 h post transplantation. Representative images of macrophage recruitment (purple) in the head (*n* = 21 T98G-GFP^{Empty} and *n* = 29 T98G-GFP^{cGAS} embryos). Scale bar, 200 μm.

E Graph presents the quantification of macrophages recruited at tumor site 24 h post xenotransplantation in (D) (*n* = 21 T98G-GFP^{Empty} and *n* = 29 T98G-GFP^{cGAS} embryos).

F Heads of zebrafish treated as in (D) were isolated prior to RNA extraction and analysis of M1 (*tnfa* and *cxcl11*) or M2 (*arg2* and *il10*) polarization markers. Each value in the graph is the mean of 25 embryos.

Data information: All graphs present means ± SEM. *P*-values were determined by Student's *t*-test. ns: not significant. *$P < 0.05$, **$P < 0.01$, ****$P < 0.0001$. Mann–Whitney test was performed to analyze macrophage recruitment in zebrafish.
Source data are available online for this figure.

© 2022 The Authors

(Fig 6A). Owing to the low number of patients presenting a *PRKDC*^{low}/*MB21D1*^{high} profile, those were not included in the analysis. Analysis of mRNA levels of total macrophages, chemokines/cytokines, and macrophage polarization markers was performed between these groups. We found that expression of *MB21D1* correlated with high expression of *CXCL10*, *CCL2* and *CCL5* chemokines (Fig 6B) and with the increased presence of macrophages (Fig 6C–E). Calculating M1/M2 ratios did not reveal any significant enrichment of a specific subpopulation (Fig 6F). Consistent with Pan-cancer analysis (An *et al*, 2019), analysis of patient survival showed that higher *MB21D1* expression led to worst patient survival as compared to patients with low *MB21D1* expression, supporting that expression of cGAS is a poor outcome marker in glioblastoma (Fig 6G). In addition, analysis of *PRKDC* and *MB21D1* expression in glioblastoma of grades II, III, and IV, indicates that *PRKDC* and *MB21D1* expression increased significantly with the aggressiveness of the tumors (Fig 6H). Next, we performed immunohistochemistry analyses of surgical specimens of human brain tumors (Fig 6I). This showed a positive correlation between cGAS and DNA-PKcs protein levels (Table 1) and increased levels of both proteins with tumor grade (Table 2). Thus, altogether, our data support that DNA-PK and cGAS cooperate to foster a pro-inflammatory environment, by enhancing the production of cytokines and chemokines that attract macrophages to the tumor vicinity, a process that inhibits early tumorigenesis, but fuels cancer-associated inflammation at later stages (Fig 6J).

Discussion

We demonstrate that DNA-PK and cGAS synergize for the production of type I IFNs and chemokines, thus dictating the composition of the tumor microenvironment. This cooperation is therefore an attractive target to modulate tumor immunogenicity. Our data further reveal that the molecular determinants of the activation of DNA-PK-dependent signaling are governed by cell-type specific rules, that, if adequately harnessed, may allow targeting inflammatory responses in specific cells of the tumor microenvironment.

Intriguingly, previous reports have shown that DNA-PKcs can inhibit cGAS activation by direct modulation of its phosphorylation of T68 and S213 (Sun *et al*, 2020) or by inducing cytosolic translocation of PARP1 (Wang *et al*, 2022). While seemingly contradictory with our findings and the recently published description of a requirement for KU subunits for optimal cGAS activation (Tao *et al*, 2022), these studies mostly focused on the role played by DNA-PK-associated inflammatory responses in the context of HSV-1 infection. In that context, it is possible that HSV-1-associated factors and additional cellular perturbations promote activation of pathways that eventually bypass the initial requirement of DNA-PK-dependent activation of cGAS. In addition, our study mostly focused on the study of acute nucleic acid challenge, while HSV-1 infection is monitored at 16 h post infection. In this context, one may hypothesize that at early time points upon challenge with immune-stimulatory nucleic acid species, DNA-PK activation is required for priming cGAS activation, while at later time points, DNA-PK activity may be required to prevent overactivation of inflammatory responses. This ultimately indicates that assessment of the crosstalk between nucleic acid detection pathways necessitates investigation in a time-dependent manner.

Tumor-associated macrophages are the most abundant immune cell population in the glioblastoma tumor microenvironment (Buonfiglioli & Hambardzumyan, 2021) and their presence is generally an indicator of poor outcomes for glioblastoma patients (Wei *et al*, 2020). In agreement, blocking macrophage recruitment through *Ccl2* genetic ablation ameliorates mice survival (Chen *et al*, 2017). Moreover, patients with low tumoral *CCL2* expression survived significantly longer than those with high *CCL2* (Chen *et al*, 2017). Since pDNA-PKcs expression positively correlates with tumor progression (Lan *et al*, 2016), and in the light of our meta-analysis, it is tempting to hypothesize that in tumors where the cGAS-STING pathway fuels tumorigenesis, the use of DNA-PKcs inhibitor may facilitate tumor clearance.

Indeed, DNA-PK inhibitors have been used in preclinical studies in glioblastoma, bringing promising results (Lan *et al*, 2016, Timme *et al*, 2018), and several clinical trials are ongoing (NCT02977780 and NCT04555577). To fully exploit the benefit of DNA-PKcs inhibition, our data support that the expression of cGAS is an important parameter to consider. Conversely, DNA-PK agonists could allow the re-establishment of inflammatory responses in tumors in which the cGAS pathway is not functional, or boost anti-tumoral

Figure 6. cGAS and DNA-PKcs levels increase with tumor grade.

- A Correlation plot between *PRKDC* and *MD21B1* expression in glioblastoma patients. Three distinct populations can be visualized: *PRKDC*^{low}/*MD21B1*^{low}; *PRKDC*^{high}/*MD21B1*^{low} and *PRKDC*^{high}/*MD21B1*^{high} (total patients $n = 224$).
- B Violin plots present chemokine gene expression (*CXCL10*, *CCL2* and *CCL5*) in glioblastoma samples from (A).
- C Violin plots present macrophage gene expression (*CD68* and *ITGAM*) in glioblastoma samples from (A).
- D Violin plots present pro-inflammatory M1 macrophage gene expression (*CD86* and *CD80*) in glioblastoma samples from (A).
- E Violin plots present anti-inflammatory M2 macrophages gene expression (*CD200R1* and *CD209*) in glioblastoma samples from (A).
- F Violin plots present M1/M2 gene expression ratio, calculated using the mean expression of the genes indicated in (D) and (E).
- G Graph presents the survival rate of glioblastoma patients from A that present *PRKDC*^{low}/*MB21D1*^{low}, *PRKDC*^{high}/*MB21D1*^{low} and *PRKDC*^{high}/*MB21D1*^{high} expression.
- H Violin plots present the expression of *PRKDC* and *MB21D1* in datasets analyzed in A, based on tumor grade (II to IV).
- I Representative images of immunohistochemical analysis of DNA-PKcs and cGAS proteins in human brain tumor samples. Scale bar, 75 μm.
- J Schematic representation of the molecular mechanisms involved in the cooperation between DNA-PKcs and cGAS for type I IFN responses and chemokine secretion. In cells with undetectable cGAS levels, interaction with exogenous (1) or endogenous (2) cytosolic dsDNA leads to DNA-PK activation (3) and promotes IRF3-dependent type I IFN responses (4). In cells where both DNA-PK and cGAS are expressed, cytosolic DNA is detected by both DNA-PK and cGAS (5). DNA-PK induces cytokine and chemokine secretion through IRF3 activation and enables cGAS phosphorylation, thus promoting the activation of the cGAS-STING pathway. This cooperation fuels cancer-associated inflammation.

Data information: All graphs present means ± SEM. *P*-values were determined by Student's *t*-test. ns: not significant. $*P < 0.05$, $**P < 0.01$, $***P < 0.001$, $****P < 0.0001$. One-way Anova with Tukey's multiple comparisons test was used to compare gene expression among populations in glioblastoma dataset.
Source data are available online for this figure.

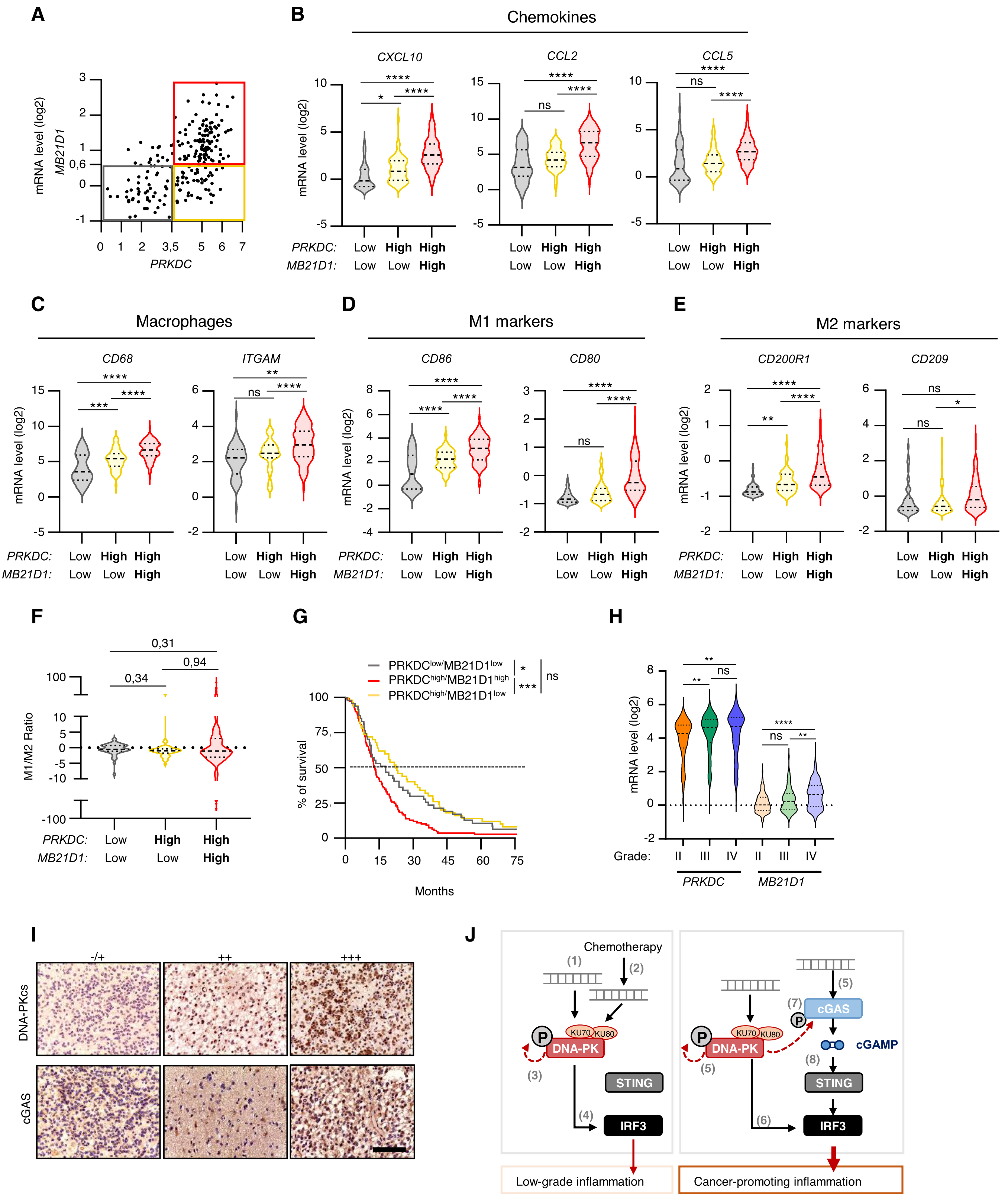

Figure 6.

 © 2022 The Authors

Table 1. Correlation between the levels of DNA-PKcs and cGAS in surgical specimens of human brain tumors.

	DNA-PKcs expression					
		−/+	++	+++	Total	*R*/*P* value
cGAS	−/+	9 (69.2%)	6 (26.1%)	2 (22.2%)	17 (37.8%)	
	++	2 (15.4%)	13 (56.5%)	1 (11.1%)	16 (35.6%)	
	+++	2 (15.4%)	4 (17.4%)	6 (66.7%)	12 (26.6%)	$R = 0.406$
	Total	13 (100%)	23 (100%)	9 (100%)	45 (100%)	$P = 0.006$

Summary of the expression patterns of DNA-PKcs and cGAS in human glioma samples. The correlation between DNA-PKcs and cGAS protein levels was analyzed using SPSS Pearson Chi-Square test ($R = 0.406$, $P = 0.00568$). A *P*-value of < 0.05 was set as the criterion for statistical significance.

Table 2. Correlation between the levels of DNA-PKcs or cGAS and tumor grades in surgical specimens of human brain tumors.

	Tumor grades					
		I/I–II	II/II–III	III/III–IV	IV	*R*/*P* value
cGAS	−/+	6 (66.7%)	3 (18.8%)	5 (41.7%)	0 (0%)	
	++	3 (33.3%)	7 (43.8%)	5 (41.7%)	1 (25%)	
	+++	0 (0%)	6 (37.4%)	2 (16.6%)	3 (75%)	$R = 0.3211$
	Total	9 (100%)	16 (100%)	12 (100%)	4 (100%)	$P = 0.0406$
DNA-PKcs	−/+	5 (55.6%)	3 (18.8%)	6 (50%)	0 (0%)	
	++	4 (44.4%)	10 (62.5%)	1 (8.3%)	0 (0%)	
	+++	0 (0%)	3 (18.8%)	5 (41.7%)	4 (100%)	$R = 0.4335$
	Total	9 (100%)	16 (100%)	12 (100%)	4 (100%)	$P = 0.0046$

Summary of the expression patterns of DNA-PKcs and cGAS in human glioma samples. The correlation between tumor grades and DNA-PKcs or cGAS protein levels was analyzed using Pearson correlation test. A *P*-value of < 0.05 was set as the criterion for statistical significance.

inflammatory responses in those expressing cGAS. This approach may represent a promising therapeutic avenue in glioblastoma patients where STING agonists have shown benefits (preprint: Berger *et al*, 2022). Yet, the use of STING agonists has shown cell-type specific drawbacks that should not be overlooked (Gulen *et al*, 2017, Liu & Guan, 2018, Vila *et al*, 2022). Along the same line, our data support that exploring the functionality of DNA-PK and the impact of STING activation in the different cell types composing the tumor microenvironment is critical for patient stratification.

Using a transgenic zebrafish line, we revealed that the presence of cGAS in tumor cells, at early stages, is sufficient to elicit myeloid cell recruitment and polarization into M1 macrophages that are key players in the initiation of antitumor responses. However, this experimental set-up does not allow evaluation of the role played by cells of the adaptive immune system, which could contribute to shape the overall antitumor response. Yet, high levels of cGAS and STING predict poor prognosis (An *et al*, 2019), and recent reports underscore that the presence of a functional cGAS-STING axis supports tumorigenesis of chromosomally unstable cancers (Hong *et al*, 2022), which is in agreement with our glioblastoma patient data analysis. This observation, together with the fact that terminally differentiated healthy tissues do not express a functional cGAS-STING signaling axis (Dickson, 2016) and that primary tumors repress cGAS-STING activity (Bakhoum *et al*, 2018; Bakhoum & Cantley, 2018), supports that cGAS expression is acquired during tumorigenesis, to the contrary of prior assumptions that cGAS downregulation may be an immune escape mechanism.

Our study raises the possibility that in inflammatory pathologies presenting with pathological chronic STING activation, inhibition of DNA-PKcs in combination with classical Janus kinase inhibitors, which are already used in standard patient care (Sanchez *et al*, 2018), may allow better suppression of chronic type I IFN responses. Conversely, *PRKDC* mutations are associated with autoinflammatory pathologies (Mathieu *et al*, 2015; Esenboga *et al*, 2018) in which cGAS-STING activation should be explored for the design of novel therapeutic strategies.

Materials and Methods

Reagents and Tools table

Reagent/resource	Reference or source	Identifier or catalog number
**Experimental models**		
Athymic Nude Mice (*M. musculus*)	Envigo	Foxn1-nu
Zebrafish	In house	tg(mfap4:RFP)

© 2022 The Authors

Reagents and Tools table (continued)

Reagent/resource	Reference or source	Identifier or catalog number
THP1 (*H. sapiens*)	Gift SR Paludan	RRID:CVCL_0006
HCT116 (*H. sapiens*)	Gift P. Pourquier	RRID:CVCL_B7PT
T98G (*H. sapiens*)	Gift C. Goujon	RRID:CVCL_0556
Gli4 (*H. sapiens*)	Gift J.-P. Hugnot	N/A
Gli7 (*H. sapiens*)	Gift J.-P. Hugnot	N/A
CFPAC (*H. sapiens*)	Gift N. Bonnefoy	RRID:CVCL_1119
GL261 (M.Musculus)	Gift C. Vanpouille-Box	RRID:CVCL_Y003
GSC 4, 6, 9 13, 15 (*H. sapiens*)	J.Gavard & N. Bidère	N/A
293T (*H. sapiens*)		RRID:CVCL_0063
MEF (*M. musculus*)		N/A
**Recombinant DNA**		
pOZ-F/HA cGAS	In house	N/A
pOZ-F/HA	In house	N/A
pOZ-F/HA cGAS-CD	In house	N/A
sfGFP-N1	Addgene	Cat #54737; RRID:Addgene_54737
LentiCRISPRv2GFP plasmid	Addgene	Cat # 82416; RRID:Addgene_82416
LentiCRISPR v2 plasmid	Addgene	Cat # 52961; RRID:Addgene_52961
pLV[CRISPR]-hCas9:T2A:Neo-U6 plasmid	VectorBuilder	
**Antibodies**		
pDNA-PKcs Ser2056	Abcam	Cat# ab124918, RRID:AB_11001004
pDNA-PKcs Ser2056	Abcam	Cat# ab18192, RRID:AB_869495
DNA-PKcs	Bethyl	A300-517AT
DNA-PKcs	Abcam	Cat# ab32566, RRID:AB_731981
KU70	Cell Signaling Technology	Cat# 4104, RRID:AB_1904185
KU80	Cell Signaling Technology	Cat# 2753, RRID:AB_2257526
cGAS	Cell Signaling Technology	Cat# 31659, RRID:AB_2799008
pcGAS Ser420	Abclonal	AP1228
pNF-kB p65 Ser536	Cell Signaling Technology	Cat# 3033, RRID:AB_331284
NF-kB p65	Cell Signaling Technology	Cat# 8242, RRID:AB_10859369
pSTING Ser366	Cell Signaling Technology	Cat# 19781, RRID:AB_2737062
pSTING Ser366	Cell Signaling Technology	Cat# 50907, RRID:AB_2827656
STING	Cell Signaling Technology	Cat# 13647, RRID:AB_2732796
pIRF3 Ser386	Abcam	Cat# ab76493, RRID:AB_1523836
IRF3	Cell Signaling Technology	Cat# 11904, RRID:AB_2722521
IRF3	Proteintech Europe	Cat# 11312-1-AP, RRID:AB_2127004
γH2AX	Cell Signaling Technology	Cat# 9718, RRID:AB_2118009
HSP90	Cell Signaling Technology	Cat# 4877, RRID:AB_2233307
GAPDH	Proteintech Europe	Cat# 60004-1-Ig, RRID:AB_2107436
GAPDH	Santa Cruz	Cat# sc-32233, RRID:AB_627679
αTUBULIN	Proteintech Europe	Cat# 66031-1-Ig, RRID:AB_11042766
αTUBULIN	Santa Cruz	Cat# sc-8035, RRID:AB_628408
Mouse Anti-rabbit IgG	Cell Signaling Technology	#7074; RRID:AB_2099233
Horse anti-mouse IgG	Cell Signaling Technology	#7076; RRID:AB_330924
FITC-anti-human CD38	Biolegend	Cat# 303503, RRID:AB_314355
PE-anti-human CD206	Biolegend	Cat# 321105, RRID:AB_571910

© 2022 The Authors

Reagents and Tools table (continued)

Reagent/resource	Reference or source	Identifier or catalog number
PE-anti-human CD209	Biolegend	Cat# 330106, RRID:AB_1134052
APC-anti-human CD86	Biolegend	Cat# 305411, RRID:AB_493232
FITC-anti-human CD14	Miltenyi Biotec	Cat# 130-110-576, RRID:AB_2655048
**Oligonucleotides and other sequence-based reagents**		
qPCR primers	This study	Table EV1
Guide RNA	This study	Table EV1
dsDNA probes	This study	Table EV1
**Chemicals, enzymes and other reagents**		
NU7441	Biotechne/Tocris	#3712
DMSO	Sigma	D2650
Puromycin	Sigma	P8833
Ethylenediamine tetraacetic acid (EDTA)	Sigma	139-33-3
Bovine Serum Albumin (BSA)	Sigma	A2153-100G
Matrigel	Corning	356234
L-glutamine	GIBCO	A2916801
FBS	Eurobio	CVFSVF00-01
Penicillin/streptomycin	GIBCO	10378016
0.25% trypsin EDTA	GIBCO	25200-056
DMEM	Lonza	BE12-614F
RPMI 1640	Lonza	LZBE12-167F
OPTIMEM	GIBCO	51985
B27	Invitrogen	0050129
N2	Invitrogen	17502048
DMEM/F12	GIBCO	11320033
EGF	Peprotech	AF-100-15
Trizol	Thermofisher	15596018
Super script IV	Thermofisher	18090050
TB Green Premix Ex Taq	TAKARA	RR420W
Rnase out	Thermofisher	10777019
dNTP mix 10mM	Thermofisher	18427013
Kit TURBO DNA-free	Thermofisher	AM1907
Protein assay dye reagent	Biorad	5000006
Jet Prime tranfection kit	Ozyme	POL114-75
GeneJuice transfection reagent	Sigma	70967-5
INTERFERin	Polyplus	POL406-50
PhosphoSTOP	Roche	4906845001
4–15% Mini-PROTEAN® TGX™ Precast Protein Gels	Bio-Rad	#4561086
NuPAGE 10 or 12% Bis-Tris Mini Protein gels	Bio-Rad	NP0302BOX ; NP0342BOX
Trans-Blot Turbo Transfer Pack 0.2 μm Nitrocellulose Midi	Biorad	1704159
SuperSignal West Pico PLUS Chemiluminescent Substrate	Thermofisher	34577
SuperSignal West Femto Maximum Sensitivity Substrate	Thermofisher	34094
Dynabeads M280	Thermofisher	11205D
2′3′-cGAMP ELISA Kit	Cayman	CAY501700
Proteome Profiler Human Chemokine Array Kit	R&D Systems	ARY017
Mammalian Protein Extraction Reagent (M-PER) buffer	Thermo Fisher	78501

© 2022 The Authors

Reagents and Tools table (continued)

Reagent/resource	Reference or source	Identifier or catalog number
**Software**		
Prism Software	GraphPad	Version 9.1
ImageJ	N/A	N/A
Image Lab	Bio-Rad Laboratories	N/A
**Other**		
Thermocycler	N/A	N/A
ChemiDoc Imaging System	Bio-Rad	N/A
LightCycler®	Roche Life Science	N/A
Trans-Blot® Turbo™ Transfer System	Bio-Rad	N/A

Methods and Protocols

Animals

Experiments in mice were conducted using Athymic Nude Foxn1-nu males in which subcutaneous injections were performed, prior to follow-up of tumor size over time using a caliper. These experiments were performed in agreement with European rules and regulations for animal handling (25066-2020040315236430).

In vivo experiments in zebrafish were performed using the *tg (mfap4:RFP)* zebrafish line. Each experiment was conducted using at least 21 individual fish per condition. All experimental procedures on zebrafish were performed in accordance with the European guidelines and regulations for Animal Protection and authorization no. F341725 from the French Ministry of Health.

Cells and cell cultures

THP-1^{CTRL}, THP-1$^{cGAS-/-}$ and THP-1$^{STING-/-}$ were a gift of S. R. Paludan, HCT116^{CTRL}, and HCT116$^{PRKDC-/-}$ were obtained from P. Pourquier, while parental T98G and CFPAC were provided by C. Goujon and N. Bonnefoy, respectively. Gli4 and Gli7 were a gift from J.-P. Hugnot. GL261^{CTRL} and GL261$^{cGAS-/-}$ were a gift of C. Vanpouille-Box.

293T, T98G, CFPAC and MEF and their genetically engineered derivatives were maintained in Dulbecco's modified Eagle's medium (DMEM) supplemented with 10% fetal bovine serum (FBS, Eurobio), 1% penicillin/streptomycin (Lonza), and 1% L-glutamine (Lonza).

GL261 and their genetically engineered derivatives were maintained in DMEM supplemented with 10% FBS, 1% penicillin/streptomycin, 1% L-glutamine, 50 mg/ml gentamicin, 5 mM HEPES, and 50 μM 2-mercaptoethanol. HCT116, THP-1 cells and their derivatives were cultured in RPMI media (Lonza) supplemented with 10% FBS, 1% penicillin/streptomycin, and 1% L-glutamine.

Human glioblastoma cancer stem cells Gli4 and Gli7 were cultured in T75 tissue culture flasks precoated with 40 μg/cm^2 of poly-2-hydroxyethyl methacrylate (poly-HEME, Sigma) to avoid cell adhesion. They were cultivated in DMEM/F12 1:1 (Invitrogen), N2 and B27 supplements (Invitrogen), 2 mM glutamine (Invitrogen), 0.6% glucose (Sigma), 20 μg/ml bovine insulin (Sigma) supplemented by 2 μg/ml Heparin (Sigma), 20 ng/ml EGF (Peprotech) and 10 ng/ml FGF2 (Peprotech). Patient-derived glioblastoma stem-like cells (GSC 4, 6, 9, 13, and 15) were maintained as nonadherent spheroids in serum-free medium (DMEM/F12, with N2, G5 and B27 supplements, GlutaMAX and antibiotics, Life Technologies), as described in (Harford-Wright *et al*, 2017).

Compounds

NU7441: PubChem SID 249565690.

Camptothecin: PubChem CID 24360.

Viral particle production and transduction

To generate knockout and control cell lines, lentiviral particles were produced by co-transfection of 2 × 10^6 293T cells with 5 μg of LentiCRISPRv2GFP or LentiCRISPR v2 plasmids expressing the gRNA targeting the gene of interest or non-targeting control (CTRL) gRNA, 5 μg of psPAX2 and 1 μg of pMD2.G, using the standard calcium phosphate transfection protocol. To generate the T98G cell lines stably expressing WT-cGAS, cGAS-CD, or the corresponding control cell line, retroviral particles containing the transgene encoding Flag- and HA-tagged cGAS (F/HA-cGAS) alleles were produced by co-transfecting 1 × 10^6 293T cells with 5 μg of pOZ-F/HAcGAS or pOZ-F/HAcGAS-CD or pOZ-F/HA, 2.5 μg of MLV GagPol, and 2.5 μg of A-MLV envelope.

Viral particles were harvested 48 h after transfection, filtered with 0.45 μM filters prior to transduction of 6 × 10^5 T98G cells. Medium was replaced 8 h post transduction. Selection was performed 72 h post transduction using 2 μg/ml puromycin for at least 3 days. Selected cells were amplified and the levels of the protein of interest analyzed by Western blot (WB).

Generation of knock-out cell lines

T98G$^{IRF3-/-}$ and T98G^{CTRL} cell lines were generated using the LentiCRISPRv2GFP plasmid (Addgene # 82416). T98G cells were transduced with lentiviral particles and 72 h post-transduction GFP-positive cells were sorted and pooled in a 6-well plate using a BD FACS melody. Cells were next amplified and levels of IRF3 controlled by WB.

Generation of T98G$^{STING-/-}$ and T98G^{CTRL} cell lines was conducted as above, except that the LentiCRISPR v2 plasmid (Addgene #5296) was used, and cells were selected 72 h post transduction using 2 μg/ml puromycin for 7 days. Cells were subsequently amplified and expression of STING controlled by WB. CFPAC$^{cGAS-/-}$ and CFPAC^{CTRL} cell lines were generated using a similar protocol except that puromycin-selected cells were further subjected to clonal selection using limiting dilutions. Clones were subsequently selected based on cGAS protein levels as evaluated by WB.

© 2022 The Authors

GL261$^{cGas-/-}$ and GL261^{CTRL} were generated using the lentiviral vector pLV[CRISPR]-hCas9:T2A:Neo-U6 plasmid containing specific guide RNA (VectorBuilder). Seventy-two hours after lentiviral transduction, cells underwent G418 selection (1 mg/ml) and were subjected to clonal selection using limiting dilutions. Resulting cGAS-deficient clones were screened by WB.

Guide RNAs for the generation of knock-out cell lines are available in Table EV1.

Generation of cell lines stably expressing cGAS

T98G overexpressing F/HA-cGAS (T98G^{cGAS}) or a catalytic dead cGAS allele (T98G$^{cGAS\text{-}CD}$), and their control cell line (T98G^{Empty}) were generated by transducing parental T98G with retroviral particles produced by using the pOZ-F/HAcGAS; pOZ-F/HAcGAS-CD construct or empty vector, respectively, and selected with 2 μg/ml puromycin for 7 days.

Generation of fluorescent glioblastoma cell line for zebrafish experiments

To obtain T98G-GFP^{Empty} and T98G-GFP^{cGAS}, T98G^{Empty}, and T98G^{cGAS} cell lines were stably transfected with sfGFP-N1 (Addgene #54737) using phosphate calcium. After transfection, cells were selected using Geneticin (800 μg/ml) for 4–6 weeks prior to zebrafish experiments.

Site directed mutagenesis

To generate the catalytic dead mutant of cGAS, site-directed mutagenesis was performed using the Quickchange Lightning kit (Agilent) following the manufacturer's instruction and primers (Fwd: 5′-ggcggttttcacgtgatagtcgctgaacttgtccaagtgt-3′; rev: 5′-acacttggacaa gttcagcgactatcacgtgaaaaccgcc-3), purchased from Eurofins Genomics.

Gene silencing

Silencing of KU70, KU80, and DNA-PKcs was achieved in T98G cells using siRNAs and INTERFERin (Polyplus) following the manufacturer's instructions.

siRNAs (Dharmacon™—Horizon Discovery) were used and sequences are available in Table EV1.

Synthetic dsDNA probes

To generate non-biotinylated or biotinylated dsDNA probes, annealing was performed as described in (Guerra *et al*, 2020), using single strand probes obtained by IDT (Stetson & Medzhitov, 2006). Probe sequences are available in Table EV1.

Whole-cell extract preparation and immunoblot

Cells were lysed in 5 packed cell volume of TENTG-150 (20 mM Tris–HCl [pH 7.4], 0.5 mM EDTA, 150 mM NaCl, 10 mM KCl, 0.5% Triton X-100, 1.5 mM $MgCl_2$, and 10% glycerol, supplemented with 10 mM β-mercaptoethanol and 0.5 mM PMSF) for 30 min at 4°C. Lysates were centrifuged 30 min at 14,000 *g*, and supernatants were collected for WB. For phosphorylated protein analysis, buffer was supplemented with PhosphoSTOP (Sigma) before whole-cell extraction. Protein quantification was performed using Bradford assay (Bio-Rad). Samples were run on either 4–15% Mini-PROTEAN® TGX™ Precast Protein Gels (Bio-Rad) (when analysis of DNA-PKcs and γH2AX was required) or on NuPAGE 10 or 12%, Bis-Tris Mini Protein gels (Invitrogen). Proteins were transferred onto nitrocellulose membranes. Membranes were incubated with primary antibodies (1:1,000 dilution except when indicated) for 2 h at RT or overnight at 4°C. Primary antibodies used include: anti-pDNA-PKcs Ser2056 (ab124918, Abcam), anti-pDNA-PKcs Ser2056 (ab18192, Abcam) for mouse cell lines, anti-DNA-PKcs (A300-517AT, Bethyl, 1:500), anti-DNA-PKcs (ab32566, Abcam) for mouse cell lines, anti-KU70 (4104S, Cell Signaling Technology), anti-KU80 (2753S, Cell Signaling Technology), anti-pcGAS Ser420 (AP1228, Abclonal), anti-cGAS (15102, Cell Signaling Technology), anti-cGAS (31659, Cell Signaling Technology) for mouse cell lines, anti-pNF-kB p65 Ser536 (3033P, Cell Signaling Technology), NF-kB p65 (8242, Cell Signaling Technology), anti-pSTING Ser366 (19781, Cell Signaling Technology), anti-pSTING Ser366 (50907, Cell Signaling Technology) for GSC cells, anti-STING (13647, Cell Signaling Technology), anti-pIRF3 Ser386 (ab76493, Abcam), anti-IRF3 (11904, Cell Signaling Technology), anti-IRF3 (11312-1-AP, Proteintech Europe) for GSC cells, anti-γH2AX (9718, Cell Signaling Technology), anti-HSP90 (4877, Cell Signaling Technology), anti-GAPDH (60004-1-Ig, Proteintech Europe, 1:5,000), anti-GAPDH (sc-32233, Santa Cruz, 1:50,000) for GSC cells, anti-αTUBULIN (66031-1-Ig, Proteintech Europe, 1:10,000), anti-αTUBULIN (sc-8035, Santa Cruz) for GSC cells. Membranes were incubated with secondary antibodies (Cell Signaling Technology) at 1:2,000 dilution, for 1 h at RT. Signal was visualized with SuperSignal West Pico Chemiluminescent Substrate (Thermo Fisher Scientific) or SuperSignal West Femto Maximum Sensitivity Substrate (Thermo Fisher Scientific), and images were acquired on a ChemiDoc (Bio-Rad) or using Amersham Hyperfilm™ ECL (GE Healthcare) films.

Biotinylated nucleic acid pull-down using cell extracts following dsDNA transfection

Interaction of endogenous proteins and transfected biotinylated nucleic acids was assessed by transfecting T98G and THP-1 cells with nucleic acids (1 μg/ml) using JetPrime, according to the manufacturer's protocol. Six hours after transfection, cells were harvested and lysed in TENTG-150 on ice for 30 min. Lysates were centrifuged at 14,000 *g* for 30 min at 4°C. Equal amounts of whole-cell lysates were incubated for 3 h at 4°C on a wheel with 30 μl Dynabeads M280 pre-blocked in (100 mM NaCl, 2 mM DTT, 20 mg/ml BSA) overnight at 4°C on a wheel. After three washes in buffer (20 mM Tris–HCl [pH 7.4], 0.5 mM EDTA, 0.05% Triton, 0.1% Tween, 150 mM NaCl, 10% glycerol, and 5 mM $MgCl_2$), bound material was eluted in 30 μl Laemmli buffer. Protein interaction with the transfected biotinylated nucleic acids was assessed by WB.

In vitro biotinylated nucleic acid pull-down

Pull-down was carried out using 30 μl of Dynabeads M280 per condition. Beads were blocked overnight as described above. After three washes in Washing Buffer (5 mM Tris–HCl [pH 7.5], 1 mM EDTA, 2 M NaCl), 3 μg of nucleic acids was coupled to 30 μl of beads according to the manufacturer's instructions before equilibration in TENTG-150. Beads were then washed once with Washing Buffer and equilibrated in TENTG-150. Whole cell extracts were diluted in TENTG-150. One milliliter of diluted lysate was added to the beads and incubated at 4°C on a wheel for 3 h in low-binding tubes (Axygen). Three consecutive washes were performed as above. Bound material was eluted in 30 μl of Laemmli buffer.

© 2022 The Authors

Protein interaction with the biotinylated nucleic acids was assessed by WB.

RNA extraction and RT–qPCR

RNA was extracted using TRIzol (Invitrogen) and treated with TURBO DNase (Ambion) according to manufacturer's protocols. RNA was quantified with a Nanodrop spectrophotometer (ND-1000, Nanodrop Technologies). RNA (1–2 μg) was reverse transcribed using SuperScript IV reverse transcriptase (Invitrogen). Expression of specific mRNAs was determined with a LightCycler 480 (Roche) using the SYBR green PCR master mix (Takara). Reactions were performed in duplicate or triplicate, and relative amounts of cDNA were normalized to Glyceraldehyde3-phosphate dehydrogenase (*GAPDH*) for mouse and human cells, except for GSC9 cDNA where Actin beta (ACTB) was used for normalization or eukaryotic translation elongation factor 1 alpha 1, like 1 (*ef1a*) for zebrafish analyses.

Primers used for RT–qPCR analysis are available in Table EV1.

cGAMP ELISA

For cGAMP quantification, $T98G^{Empty}$, $T98G^{cGAS}$, THP-1^{CTRL}, and THP-$1^{cGAS-/-}$ were seeded 18 h before dsDNA transfection. One hour before dsDNA transfection (1 μg/ml), cells were pretreated with 2 μM NU7441 (#3712, Biotechne/Tocris) in OptiMEM. Cells were harvested 6 h post transfection, counted, washed in phosphate-buffered saline (PBS) (Sigma), pelleted, and frozen at −80°C until extraction. cGAMP extraction was performed using the commercially available Mammalian Protein Extraction Reagent (M-PER) buffer (Thermo Fisher), accordingly to the manufacture protocol. The recovered supernatants were used for cGAMP measurement, following adequate sample dilution. cGAMP enzyme-linked immunosorbent assay (ELISA) was performed according to the manufacturer's protocol using the Cayman Chemical 2′3′-cGAMP ELISA Kit (CAY501700).

Cell treatment and transfection

dsDNA transfections were conducted using previously published protocols (Chamma *et al*, 2022b). In brief, cells were plated in 6-well plate, 100 mm, or 150 mm dishes 18 h before transfection. The day of transfection, media was carefully removed, plates were washed once with 1× PBS (room temperature) and 2, 10 or 20 ml Opti-MEM added, depending on the plate size. 2, 10 or 20 μg of dsDNA was transfected with the JetPrime transfection reagent (Polyplus) at 1:2 ratio. 6 h after transfection, cells were harvested and stored at −80°C prior to protein or RNA extraction.

For dsDNA transfection in GSC9 cells. the GeneJuice transfection reagent (Sigma) was used following manufacturer's protocol. To perform DNA-PKcs inhibition followed by dsDNA transfection, cells were pretreated with 2 μM of NU7441 in Opti-MEM, 1 h prior the transfection.

When transfection was performed on Gli4 or Gli7, glioblastoma spheres were dissociated in Trypsin (0.2%, Sigma) at 37°C for 4 min. Trypsin inhibitor (Sigma, 50 mg/ml), DNase I (0.015%, Roche), and $CaCl_2$ (20 mM) were subsequently added. After mechanical dissociation, cells were resuspended in PBS 1×. Following 2 washes with PBS 1×, cells were counted and 1×10^6 cells were plated per well of a 6-well plate, precoated with poly-D-lysine (25 μg/ml) and laminin (2 μg/cm^2, Sigma).

For cGAMP transfection, 10 μM of 2′3′ cGAMP were transfected for 6 h using Lipofectamine 2000 (Thermo Fischer Scientific) following the manufacturer's instructions.

For chemotherapy treatment, cells were treated with 0.16 μM camptothecin (CPT) for 48 or 72 h, or with 25 μM etoposide (ETO) for 72 h in DMEM. When the treatment was in combination with the NU7441 DNA-PKcs inhibitor, 2 μM of NU7441 was added at 24 and 48 h post CPT or ETO treatment.

Conditioned media

For conditioned media preparation, 3.5×10^6 $T98G^{Empty}$ and $T98G^{cGAS}$ cells were seeded in 150 mm dishes 18 h prior dsDNA transfection. Six hours post transfection Opti-MEM was replaced with 13.5 ml of DMEM media. Conditioned media was collected 24 h post transfection, centrifugated and filtered using 2 μm filters and frozen at −80°C.

THP-1 polarization assay

THP-1 cell lines were treated with PMA (Phorbol 12-myristate 13-acetate) at 150 nM during 24 h. Forty-eight hours later PMA-treated THP-1 were incubated with 2/3 of conditioned media complemented with 1/3 of fresh DMEM for 24 h. Cells were then harvested and samples analyzed by RT–qPCR.

Human blood-derived cells

Buffy coats from healthy donors were obtained from the Etablissement Français du Sang (EFS, Montpellier, France). Isolation and differentiation of human $CD14^+$ monocytes were performed according to previously reported protocols (Blanchet *et al*, 2010; Maarifi *et al*, 2021). Briefly, freshly isolated $CD14^+$ monocytes were incubated with indicated conditioned media for 72 h. Cells were then harvested and samples processed for flow cytometry. As control, $CD14^+$ monocytes were also incubated for 3 days in complete media prior to analysis of polarization status by flow cytometry analysis.

Flow cytometry

Cells (10^5 cells/staining) were harvested and fixed in 1% paraformaldehyde for 15 min. Cells were then washed in PBS/1% BSA buffer and incubated for 30 min on ice with the following antibodies from Biolegend (San Diego, USA): FITC-anti-human CD38 (#303503), PE-anti-human CD206 (#321105), PE-anti-human CD209 (#330106), and APC-anti-human CD86 (#305411). After washes in PBS/1% BSA buffer, cells were acquired on a Novocyte flow cytometer (Agilent Technologies). For blood-derived monocytes phenotyping, FITC-anti-human CD14 (Miltenyi, #130-110-576) antibody from Biolegend was also used.

Mouse xenograft tumor model

Athymic Nude Foxn1-nu males (Envigo) of 7 weeks were subcutaneously injected with 5×10^6 $T98G^{Empty}$ or $T98G^{cGAS}$ in the right or left flank, respectively. Cells were injected in a total volume of 100 μl in a mix of DMEM/Matrigel (v/v). Subcutaneous tumor growth was monitored over time and tumor size measured with a caliper. Tumor volume was estimated using the formula: volume = length × width^2 × 0.526.

THP-1 migration assay

24-well cell culture inserts 3.0 μm PET clear (Cell QART) were placed into the wells of a 24-well plate, containing 500 μl of

 © 2022 The Authors

conditioned media. DMEM containing FBS was used as a negative control, while DMEM with 30 ng/ml of CCL2 as a positive control. THP-1 cells were counted, resuspended at a concentration of 3×10^5 cells/ml in RPMI without FBS and 100 µl of cell suspension added to the insert. Six hours later, the upper surface of the transwell membrane was washed twice with cold PBS and nonmigrated cells were gently scrapped with a cotton swab. Remainder cells were fixed in 100% methanol for 10 min and nuclei stained with 4′,6-diamidino-2-phenylindole (DAPI). Transwell inserts were mounted on coverslips. Apotome Z3 microscope (Zeiss) was used to visualize and count the cells that migrated through the insert.

Proteome profiler

To assess differential chemokine expression, Proteome Profiler Human Chemokine Array Kit (ARY017) was used following manufacturer's instructions. Conditioned media from $T98G^{Empty}$ and $T98G^{cGAS}$ cells were used.

Immunofluorescence and microscopy analysis

Cells were seeded on glass coverslips 18 h prior to dsDNA or CPT treatment and fixed either in methanol or with 4% PFA in 2% sucrose PBS. PFA fixation was followed by permeabilization in 0.1% Triton X-100 in PBS for 5 min at room temperature (RT). After blocking in PBS containing 0.1% Tween (PBS-T) and 5% BSA for 30 min at RT, cells were incubated overnight at 4°C with dilution in PBS-T, 5% BSA. Primary antibodies used in immunofluorescence include anti-dsDNA (ab27156, Abcam, 1:100), anti-53BP1 (MAB 3802, Sigma-Aldrich, 1:300), anti-pDNA-PKcs Ser2056 (ab18192, Abcam,1:200), and anti-DNA-PKcs (ab32566, Abcam, 1:100). Secondary antibody incubation was performed for 1 h at RT. Secondary antibodies used are Alexa Fluor 488 goat anti-Mouse IgG, (#A11001, Thermofischer) and Alexa Fluor 594-coupled goat anti-Rabbit IgG (#R37117, Thermofischer). Nuclei were stained with DAPI and coverslips mounted in Vectashield mounting media. Images were acquired by Apotome Z3 microscope by Zeiss with ZEN (blue edition) software with a 63X oil objective, and images were processed with Omero or Fiji.

Spheroids

T98G cells lines were prepared in DMEM media, and 600 cells were distributed per well of 96 well plates (Corning ultralow attachment) prior to centrifugation for 2 min at 200 g. Cells were kept in the incubator during 72 h to allow spheroid formation prior to daily scanning using a Celigo cell imaging cytometer (Nexcelom) apparatus until day 8. Volume was estimated using Celigo software.

Zebrafish experiments

The homozygous transgenic zebrafish (*Danio rerio*) line *tg(mfap4:RFP)* were maintained at 28°C at a maximum density of 50 larvae per Petri dish in fish water, containing methylene blue. After 24 h, larvae were placed in fish water, containing 200 µM 1-phenyl 2-thiourea (PTU), to prevent melanin synthesis.

Transplantation needles were prepared from borosilicate glass capillaries (outer diameter: 1 mm, inner diameter: 0.75 mm, 10 cm length, Sutter Instrument), lacking the internal filament. Using microloader tips, the transplantation needle was filled with 10 µL of cell suspension. The needle was next inserted in a needle holder, mounted on a manipulator (Narishige) and connected to the oil manual microinjector (Eppendorf CellTram vario). After anesthesia, larvae were placed in PBS containing Tricaïne, aligned and positioned dorsally between smooth forceps and 50–100 cells were transplanted in the brain region of each embryo. Transplanted larvae were next maintained at 33°C in fish water, containing PTU.

Xenotransplantation was conducted in 3 dpf embryos from the same spawn using $T98G\text{-}GFP^{Empty}$ and $T98G\text{-}GFP^{cGAS}$. Control embryos were injected with PBS. Xenotransplanted fish were used for either gene expression analysis or imaging. Briefly, for gene expression analysis, 24 h post transplantation, anesthetized embryos were manually transected with a sterile blade under a stereomicroscope to isolate the head region. Twenty-five embryo heads were then pooled in a tube (#72.693.465, Sarstedt) and frozen at −80°C prior to RNA extraction in trizol using Fastprep 24™ (MP) machine. The relative expression of M1 and M2 zebrafish macrophage markers were normalized to *ef1a*. For image acquisition embryos were placed individually in wells of a 96-well plate-adapted molds (Azelead), anesthetized using 0.16 mg/ml PBS/Tricaine (MS-222) and imaged from the day of transplantation (D0) up to D2. Z-stack Images were acquired through a Cell Discoverer 7 system (Zeiss).

Analysis of zebrafish images

To estimate tumor mass, Z-projections were performed using the Zen software (ZEISS). A threshold was then applied on the Fiji software for each embryo at D0, D1, and D2 to determine tumor area. Tumor area was next normalized to the day of transplantation (D0) to evaluate tumor growth of the injected cell lines. Macrophage recruitment around the tumor site was estimated by manual counting on Fiji, by moving the focus in all Z stacks. To characterize invasiveness, the elongated cells were counted manually on Fiji. Images were prepared for publication using the Fiji program by setting the same parameters in all compared images.

Glioblastoma RNA seq data retrieval and correlation analysis

For mRNA expression analysis, RNA seq data of CGGA-GBM data set, containing 224 glioblastoma samples was used. The data set was accessed from the GlioVis web server (http://gliovis.bioinfo.cnio.es/) and mRNA levels retrieved using the correlation tool. After plotting the mRNA level of *PRKDC* against *MB21D1*, we applied a filter cutoff to determine low and high expression allowing the definition of 4 populations: $PRKDC^{low}/MB21D1^{low}$; $PRKDC^{low}/MB21D1^{high}$; $PRKDC^{high}/MB21D1^{low}$; $PRKDC^{high}/MB21D1^{high}$. mRNA levels of genes of interest were plotted based on these populations. One-way ANOVA with Tukey's multiple comparisons test was used to compare gene expression among populations in glioblastoma dataset.

Human tumor tissue array analysis

The human nervous system glioma tissue array (NGL961) was obtained from Pantomics Inc (Fairfield, CA, USA). The study cohort comprised 41 gliomas of various grades and stages and four healthy tissues. This work was performed in accordance with the Institutional Review Board (IRB) approval at MD Anderson Cancer Center (Houston, TX, USA). The tissue array slides were incubated with primary antibodies against DNA-PKcs (1:100; Abcam, ab32566) and cGAS (1:200; Abcam, ab224144) and biotin-conjugated secondary antibodies, and then incubated with an avidin-biotin-peroxidase

complex. Visualization was performed using 3,3′-diaminobenzidine (DAB) chromogen. The tissue array cores were scored by two researchers blind to cancer outcomes. According to histologic scoring, the intensity of staining was ranked into one of three groups: high (+++, for scores 1.5 and 2), medium (++, for score 1), and low or negative (−/+, for scores 0 and 0.5). While Table 1 includes the entire cohort, Table 2 includes the analyses of the 41 gliomas tissues.

Statistical analysis

Statistical analysis was performed using GraphPad Prism version 7. For statistical analysis of *in vitro* experiments, unpaired Student's *t*-test was performed as indicated in figure legends. One-way Anova with Tukey's multiple comparisons test was used to analyze gene expression among populations in glioblastoma datasets. One-way ANOVA was used for mice studies. Mann-Whitney test was performed to analyze tumor growth and macrophage recruitment in zebrafish. The number of replicates in each experiment (including number of mice or zebrafish) are indicated in the figure legends. All data are expressed as mean ± SEM. The statistical parameters can be found in the figures and the figure legends. Ns: non-significant. $*P < 0.05$, $**P < 0.01$, $***P < 0.001$ and $****P < 0.0001$.

Data availability

This study includes no data deposited in external repositories. Glioblastoma patient data are available at http://gliovis.bioinfo.cnio.es/ (Bowman *et al*, 2017). Source data for Figs 4F and EV4B can be found on the BioImage Archive using accession number S-BIAD571.

Expanded View for this article is available online.

Acknowledgements

We thank P. Pourquier for the HCT116 cell lines, N. Bonnefoy for the parental CFPAC cell line, Soren Paludan for THP-1 cell lines, C. Vanpouille-Box for the GL261^{CTRL} and GL261$^{cGAS-/-}$ cell lines and E. Chevet for the gift of parental GL261 cells. We thank C. Goujon for the parental T98G cell line and CRISPR/Cas9 gRNA sequences. We thank C. Langevin and all members of the molecular basis of inflammation laboratory for discussions. We thank N. Dupuis and A. Blömeke-Eiben for technical assistance. We acknowledge iExplore-RAM animal facility, the MRI imaging facility, member of the national infrastructure France-BioImaging infrastructure supported by the French National Research Agency (ANR-10-INBS-04, "Investments for the future") and the SIRIC Montpellier Cancer (INCa_Inserm_DGOS_12553). Work in NL's laboratory was funded by the European Research Council (ERC-Stg CrIC: 637763, ERC-PoC DIM-CrIC: 893772), LA LIGUE pour la recherche contre le cancer, Agence Nationale de Recherche sur le SIDA et les Hépatites virales (ANRS: ECTZ117448), La Région Languedoc Roussillon, and the CNRS. CT was supported by the Merck Sharp and Dohme Avenir (MSD-Avenir—GnoSTic) program and ANRS (ECTZ119088). HC was supported by LA LIGUE pour la recherche contre le cancer. SG was supported by a LabMuse EpiGenMed. ALCV was supported by ERC-Stg CrIC: 637763, and MSD-Avenir. KP was supported by the Laboratoire d'Excellence EpiGenMed. IKV was supported by ERC-Stg CrIC: 637763, followed by Fondation pour la Recherche Médicale (FRM: ARF20170938586 "Prix Roger PROPICE pour la recherche sur le cancer du pancreas"). JM was supported by an "Agence Nationale de Recherche Technologie" (ANRT). Work in NB laboratory was funded by Fondation de France (JJ), Fondation ARC contre le Cancer (PJA to JG and NB), Institut National du Cancer (INCa PAIR-CEREB INCa_16285), Ligue nationale contre le cancer (Equipe labellisée) et comités de Loire-Atlantique, Maine et Loire, Vendée, Ille-et-Vilaine (JG, NB). CM received a fellowship from Ligue Régionale contre le Cancer and Région Pays-de-la-Loire. Work in MSS laboratory is funded by grants from National Institutes of Health (CA196740, CA258100) and Department of Defense (W81XWH-20-1-0379). Work in CVB laboratory is funded by grants from the Uncle Kory Foundation, the StacheStrong Foundation and the St. Baldrick's Foundation. MDM is supported by a 2022-SITC Nektar Therapeutics Equity and Inclusion in Cancer Immunotherapy fellowship. FPB was supported by ANRS AO1-2019-193123 and AO1-2022-192763.

Author contributions

Clara Taffoni: Conceptualization; investigation; visualization; methodology; writing – original draft; writing – review and editing. **Johanna Marines:** Investigation; visualization; methodology; writing – original draft; writing – review and editing. **Hanane Chamma:** Investigation. **Soumyabrata Guha:** Investigation. **Mathilde Saccas:** Investigation. **Amel Bouzid:** Investigation. **Ana Luiza Chaves Valadão:** Investigation; methodology. **Clément Maghe:** Investigation. **Jane Jardine:** Investigation. **Mi Kyung Park:** Investigation. **Katarzyna Polak:** Investigation; methodology. **Mara De Martino:** Investigation. **Claire Vanpouille-Box:** Supervision. **Maguy Del Rio:** Supervision. **Céline Gongora:** Supervision. **Julie Gavard:** Supervision. **Nicolas Bidere:** Supervision. **Min Sup Song:** Supervision. **Donovan Pineau:** Methodology. **Jean-Philippe Hugnot:** Supervision. **Karima Kissa:** Supervision. **Laura Fontenille:** Supervision. **Fabien P Blanchet:** Supervision; methodology. **Isabelle K Vila:** Investigation; visualization; methodology; writing – review and editing. **Nadine Laguette:** Conceptualization; supervision; visualization; methodology; writing – original draft; writing – review and editing.

Disclosure and competing interests statement

J.M. is a joint PhD student in Azelead, a startup company, and the Laguette laboratory. L.F. and K.K. are co-founders of the Azelead startup company that hosted J.M. All other authors declare that they have no competing interests.

References

Adachi N, So S, Koyama H (2004) Loss of nonhomologous end joining confers camptothecin resistance in DT40 cells. Implications for the repair of topoisomerase I-mediated DNA damage. *J Biol Chem* 279: 37343 – 37348

An X, Zhu Y, Zheng T, Wang G, Zhang M, Li J, Ji H, Li S, Yang S, Xu D *et al* (2019) An analysis of the expression and association with immune cell infiltration of the cGAS/STING pathway in pan-cancer. *Mol Ther Nucleic Acids* 14: 80 – 89

Bakhoum SF, Cantley LC (2018) The multifaceted role of chromosomal instability in cancer and its microenvironment. *Cell* 174: 1347 – 1360

Bakhoum SF, Ngo B, Laughney AM, Cavallo JA, Murphy CJ, Ly P, Shah P, Sriram RK, Watkins TBK, Taunk NK *et al* (2018) Chromosomal instability drives metastasis through a cytosolic DNA response. *Nature* 553: 467 – 472

Berger G, Knelson EH, Jimenez-Macias JL, Nowicki MO, Han S, Panagioti E, Lizotte PH, Adu-Berchie K, Stafford A, Dimitrakakis N *et al* (2022) STING activation promotes robust immune response and NK cell-mediated tumor regression in glioblastoma models. *bioRxiv* https://doi.org/10.1101/2022.02.28.481908

Blanchet FP, Moris A, Nikolic DS, Lehmann M, Cardinaud S, Stalder R, Garcia E, Dinkins C, Leuba F, Wu L *et al* (2010) Human immunodeficiency virus-1

© 2022 The Authors

inhibition of immunoamphisomes in dendritic cells impairs early innate and adaptive immune responses. *Immunity* 32: 654 – 669

Bowman RL, Wang Q, Carro A, Verhaak RG, Squatrito M (2017) GlioVis data portal for visualization and analysis of brain tumor expression datasets. *Neuro Oncol* 19: 139 – 141

Buonfiglioli A, Hambardzumyan D (2021) Macrophages and microglia: the cerberus of glioblastoma. *Acta Neuropathol Commun* 9: 54

Burleigh K, Maltbaek JH, Cambier S, Green R, Gale M Jr, James RC, Stetson DB (2020) Human DNA-PK activates a STING-independent DNA sensing pathway. *Sci Immunol* 5: eaba4219

Chamma H, Vila IK, Taffoni C, Turtoi A, Laguette N (2022a) Activation of STING in the pancreatic tumor microenvironment: a novel therapeutic opportunity. *Cancer Lett* 538: 215694

Chamma H, Guha S, Laguette N, Vila IK (2022b) Protocol to induce and assess cGAS-STING pathway activation *in vitro*. *STAR Protoc* 3: 101384

Chen BP, Chan DW, Kobayashi J, Burma S, Asaithamby A, Morotomi-Yano K, Botvinick E, Qin J, Chen DJ (2005) Cell cycle dependence of DNA-dependent protein kinase phosphorylation in response to DNA double strand breaks. *J Biol Chem* 280: 14709 – 14715

Chen Q, Boire A, Jin X, Valiente M, Er EE, Lopez-Soto A, Jacob L, Patwa R, Shah H, Xu K *et al* (2016) Carcinoma-astrocyte gap junctions promote brain metastasis by cGAMP transfer. *Nature* 533: 493 – 498

Chen Z, Feng X, Herting CJ, Garcia VA, Nie K, Pong WW, Rasmussen R, Dwivedi B, Seby S, Wolf SA *et al* (2017) Cellular and molecular identity of tumor-associated macrophages in glioblastoma. *Cancer Res* 77: 2266 – 2278

Corrales L, McWhirter SM, Dubensky TW Jr, Gajewski TF (2016) The host STING pathway at the interface of cancer and immunity. *J Clin Invest* 126: 2404 – 2411

Dickson I (2016) Viral hepatitis: a lack of hepatocyte STING favours HBV infection. *Nat Rev Gastroenterol Hepatol* 13: 438

Esenboga S, Akal C, Karaatmaca B, Erman B, Dogan S, Orhan D, Boztug K, Ayvaz D, Tezcan I (2018) Two siblings with PRKDC defect who presented with cutaneous granulomas and review of the literature. *Clin Immunol* 197: 1 – 5

Ferguson BJ, Mansur DS, Peters NE, Ren H, Smith GL (2012) DNA-PK is a DNA sensor for IRF-3-dependent innate immunity. *Elife* 1: e00047

Gonzalez-Suarez I, Redwood AB, Grotsky DA, Neumann MA, Cheng EH, Stewart CL, Dusso A, Gonzalo S (2011) A new pathway that regulates 53BP1 stability implicates cathepsin L and vitamin D in DNA repair. *EMBO J* 30: 3383 – 3396

Gu Y, Jin S, Gao Y, Weaver DT, Alt FW (1997) Ku70-deficient embryonic stem cells have increased ionizing radiosensitivity, defective DNA end-binding activity, and inability to support V(D)J recombination. *Proc Natl Acad Sci USA* 94: 8076 – 8081

Guerra J, Valadao AL, Vlachakis D, Polak K, Vila IK, Taffoni C, Prabakaran T, Marriott AS, Kaczmarek R, Houel A *et al* (2020) Lysyl-tRNA synthetase produces diadenosine tetraphosphate to curb STING-dependent inflammation. *Sci Adv* 6: eaax3333

Guichet PO, Bieche I, Teigell M, Serguera C, Rothhut B, Rigau V, Scamps F, Ripoll C, Vacher S, Taviaux S *et al* (2013) Cell death and neuronal differentiation of glioblastoma stem-like cells induced by neurogenic transcription factors. *Glia* 61: 225 – 239

Gulen MF, Koch U, Haag SM, Schuler F, Apetoh L, Villunger A, Radtke F, Ablasser A (2017) Signalling strength determines proapoptotic functions of STING. *Nat Commun* 8: 427

Hammarsten O, Chu G (1998) DNA-dependent protein kinase: DNA binding and activation in the absence of Ku. *Proc Natl Acad Sci USA* 95: 525 – 530

Hammel M, Yu Y, Mahaney BL, Cai B, Ye R, Phipps BM, Rambo RP, Hura GL, Pelikan M, So S *et al* (2010) Ku and DNA-dependent protein kinase dynamic conformations and assembly regulate DNA binding and the initial non-homologous end joining complex. *J Biol Chem* 285: 1414 – 1423

Harford-Wright E, Andre-Gregoire G, Jacobs KA, Treps L, Le Gonidec S, Leclair HM, Gonzalez-Diest S, Roux Q, Guillonneau F, Loussouarn D *et al* (2017) Pharmacological targeting of apelin impairs glioblastoma growth. *Brain* 140: 2939 – 2954

Hartmann G (2017) Nucleic acid immunity. *Adv Immunol* 133: 121 – 169

Hong C, Schubert M, Tijhuis AE, Requesens M, Roorda M, van den Brink A, Ruiz LA, Bakker PL, van der Sluis T, Pieters W *et al* (2022) cGAS-STING drives the IL-6-dependent survival of chromosomally instable cancers. *Nature* 607: 366 – 373

Ishikawa H, Ma Z, Barber GN (2009) STING regulates intracellular DNA-mediated, type I interferon-dependent innate immunity. *Nature* 461: 788 – 792

Lah TT, Novak M, Breznik B (2020) Brain malignancies: glioblastoma and brain metastases. *Semin Cancer Biol* 60: 262 – 273

Lan T, Zhao Z, Qu Y, Zhang M, Wang H, Zhang Z, Zhou W, Fan X, Yu C, Zhan Q *et al* (2016) Targeting hyperactivated DNA-PKcs by KU0060648 inhibits glioma progression and enhances temozolomide therapy via suppression of AKT signaling. *Oncotarget* 7: 55555 – 55571

Larkin B, Ilyukha V, Sorokin M, Buzdin A, Vannier E, Poltorak A (2017) Cutting edge: activation of STING in T cells induces type I IFN responses and cell death. *J Immunol* 199: 397 – 402

Li M, Shu HB (2020) Dephosphorylation of cGAS by PPP6C impairs its substrate binding activity and innate antiviral response. *Protein Cell* 11: 584 – 599

Liu S, Guan W (2018) STING signaling promotes apoptosis, necrosis, and cell death: an overview and update. *Mediators Inflamm* 2018: 1202797

Maarifi G, Lagisquet J, Hertel Q, Bonaventure B, Chamontin C, Fuchs K, Moncorge O, Tauziet M, Mombled M, Papin L *et al* (2021) Alarmin S100A9 restricts retroviral infection by limiting reverse transcription in human dendritic cells. *EMBO J* 40: e106540

Mathieu AL, Verronese E, Rice GI, Fouyssac F, Bertrand Y, Picard C, Chansel M, Walter JE, Notarangelo LD, Butte MJ *et al* (2015) PRKDC mutations associated with immunodeficiency, granuloma, and autoimmune regulator-dependent autoimmunity. *J Allergy Clin Immunol* 135: e5

Mayca Pozo F, Geng X, Tamagno I, Jackson MW, Heimsath EG, Hammer JA, Cheney RE, Zhang Y (2021) MYO10 drives genomic instability and inflammation in cancer. *Sci Adv* 7: eabg6908

Morchikh M, Cribier A, Raffel R, Amraoui S, Cau J, Severac D, Dubois E, Schwartz O, Bennasser Y, Benkirane M (2017) HEXIM1 and NEAT1 long non-coding RNA form a multi-subunit complex that regulates DNA-mediated innate immune response. *Mol Cell* 67: e5

Nussenzweig A, Chen C, da Costa SV, Sanchez M, Sokol K, Nussenzweig MC, Li GC (1996) Requirement for Ku80 in growth and immunoglobulin V(D)J recombination. *Nature* 382: 551 – 555

Sanchez GAM, Reinhardt A, Ramsey S, Wittkowski H, Hashkes PJ, Berkun Y, Schalm S, Murias S, Dare JA, Brown D *et al* (2018) JAK1/2 inhibition with baricitinib in the treatment of autoinflammatory interferonopathies. *J Clin Invest* 128: 3041 – 3052

Sen T, Rodriguez BL, Chen L, Corte CMD, Morikawa N, Fujimoto J, Cristea S, Nguyen T, Diao L, Li L *et al* (2019) Targeting DNA damage response promotes antitumor immunity through STING-mediated T-cell activation in small cell lung cancer. *Cancer Discov* 9: 646 – 661

Sivick KE, Desbien AL, Glickman LH, Reiner GL, Corrales L, Surh NH, Hudson TE, Vu UT, Francica BJ, Banda T *et al* (2018) Magnitude of therapeutic

© 2022 The Authors

STING activation determines CD8(+) T cell-mediated anti-tumor immunity. *Cell Rep* 25: e5
Song S, Peng P, Tang Z, Zhao J, Wu W, Li H, Shao M, Li L, Yang C, Duan F *et al* (2017) Decreased expression of STING predicts poor prognosis in patients with gastric cancer. *Sci Rep* 7: 39858
Stetson DB, Medzhitov R (2006) Recognition of cytosolic DNA activates an IRF3-dependent innate immune response. *Immunity* 24: 93 – 103
Sui H, Zhou M, Imamichi H, Jiao X, Sherman BT, Lane HC, Imamichi T (2017) STING is an essential mediator of the Ku70-mediated production of IFN-lambda1 in response to exogenous DNA. *Sci Signal* 10: eaah5054
Sun L, Wu J, Du F, Chen X, Chen ZJ (2013) Cyclic GMP-AMP synthase is a cytosolic DNA sensor that activates the type I interferon pathway. *Science* 339: 786 – 791
Sun X, Liu T, Zhao J, Xia H, Xie J, Guo Y, Zhong L, Li M, Yang Q, Peng C *et al* (2020) DNA-PK deficiency potentiates cGAS-mediated antiviral innate immunity. *Nat Commun* 11: 6182
Taffoni C, Steer A, Marines J, Chamma H, Vila IK, Laguette N (2021) Nucleic acid immunity and DNA damage response: new friends and old foes. *Front Immunol* 12: 660560
Tanaka Y, Chen ZJ (2012) STING specifies IRF3 phosphorylation by TBK1 in the cytosolic DNA signaling pathway. *Sci Signal* 5: ra20
Tao X, Song J, Song Y, Zhang Y, Yang J, Zhang P, Zhang D, Chen D, Sun Q (2022) Ku proteins promote DNA binding and condensation of cyclic GMP-AMP synthase. *Cell Rep* 40: 111310
Timme CR, Rath BH, O'Neill JW, Camphausen K, Tofilon PJ (2018) The DNA-PK inhibitor VX-984 enhances the Radiosensitivity of glioblastoma cells grown *in vitro* and as Orthotopic xenografts. *Mol Cancer Ther* 17: 1207 – 1216
Tu Y, Ji C, Yang B, Yang Z, Gu H, Lu CC, Wang R, Su ZL, Chen B, Sun WL *et al* (2013) DNA-dependent protein kinase catalytic subunit (DNA-PKcs)-SIN1 association mediates ultraviolet B (UVB)-induced Akt Ser-473 phosphorylation and skin cell survival. *Mol Cancer* 12: 172
Vila IK, Chamma H, Steer A, Saccas M, Taffoni C, Turtoi E, Reinert LS, Hussain S, Marines J, Jin L *et al* (2022) STING orchestrates the crosstalk between polyunsaturated fatty acid metabolism and inflammatory responses. *Cell Metab* 34: e8
Wang Y, Su GH, Zhang F, Chu JX, Wang YS (2015) Cyclic GMP-AMP synthase is required for cell proliferation and inflammatory responses in rheumatoid arthritis Synoviocytes. *Mediators Inflamm* 2015: 192329
Wang F, Zhao M, Chang B, Zhou Y, Wu X, Ma M, Liu S, Cao Y, Zheng M, Dang Y *et al* (2022) Cytoplasmic PARP1 links the genome instability to the inhibition of antiviral immunity through PARylating cGAS. *Mol Cell* 82: e7
Wei J, Chen P, Gupta P, Ott M, Zamler D, Kassab C, Bhat KP, Curran MA, de Groot JF, Heimberger AB (2020) Immune biology of glioma-associated macrophages and microglia: functional and therapeutic implications. *Neuro Oncol* 22: 180 – 194
West RB, Yaneva M, Lieber MR (1998) Productive and nonproductive complexes of Ku and DNA-dependent protein kinase at DNA termini. *Mol Cell Biol* 18: 5908 – 5920
Xia T, Konno H, Ahn J, Barber GN (2016) Deregulation of STING signaling in colorectal carcinoma constrains DNA damage responses and correlates with tumorigenesis. *Cell Rep* 14: 282 – 297
Yang H, Wang H, Ren J, Chen Q, Chen ZJ (2017) cGAS is essential for cellular senescence. *Proc Natl Acad Sci USA* 114: E4612 – E4620
Yue X, Bai C, Xie D, Ma T, Zhou PK (2020) DNA-PKcs: a multi-faceted player in DNA damage response. *Front Genet* 11: 607428
Zhang X, Brann TW, Zhou M, Yang J, Oguariri RM, Lidie KB, Imamichi H, Huang DW, Lempicki RA, Baseler MW *et al* (2011) Cutting edge: Ku70 is a novel cytosolic DNA sensor that induces type III rather than type I IFN. *J Immunol* 186: 4541 – 4545
Zhou W, Whiteley AT, de Oliveira Mann CC, Morehouse BR, Nowak RP, Fischer ES, Gray NS, Mekalanos JJ, Kranzusch PJ (2018) Structure of the human cGAS-DNA complex reveals enhanced control of immune surveillance. *Cell* 174: 300 – 311.e11
Zhu Y, An X, Zhang X, Qiao Y, Zheng T, Li X (2019) STING: a master regulator in the cancer-immunity cycle. *Mol Cancer* 18: 152

License: This is an open access article under the terms of the Creative Commons Attribution License, which permits use, distribution and reproduction in any medium, provided the original work is properly cited.

Expanded View Figures

Figure EV1. DNA-PKcs promotes type I Interferon responses in cancer cells that do not express cGAS.

A Whole cell extracts from THP-1^{CTRL}, THP-$1^{cGAS-/-}$ and T98G cells were analyzed by WB using indicated antibodies.
B Gli7 cells were challenged or not with dsDNA for 6 h prior to WB analysis using indicated antibodies.
C Whole cell extracts from patient derived-glioblastoma stem-like cells (GSC 4, 6, 9, 13, 15) were analyzed by WB using indicated antibodies.
D GSC9 cells were challenged or not with dsDNA for 6 h prior to whole cell extraction and WB analysis using indicated antibodies.
E *IFNB* and *CXCL10* mRNA levels were analyzed by RT–qPCR in samples treated as in B ($n = 3$ independent experiments).
F GSC9 cells were challenged or not for 6 h with dsDNA, in the presence or not of the NU7441 DNA-PKcs inhibitor, prior to analysis of *IFNB* and *CXCL10* levels by RT–qPCR ($n = 3$ independent experiments).
G As in (F), except that whole cell extracts were analyzed by WB using indicated antibodies.
H Gli4 cells were challenged or not for 6 h with dsDNA, in the presence or not of the NU7441 DNA-PKcs inhibitor, prior to analysis of *IFNB* and *CXCL10* levels by RT–qPCR. Graphs present a representative biological triplicate ($n = 3$ independent experiments).
I T98G cells were treated with non-targeting (CTRL) or DNA-PKcs-targeting siRNAs prior to whole cell extraction and WB analysis using indicated antibodies.
J Whole cell extracts from T98G cells, treated or not with camptothecin (CPT) for 48 h, in presence or not of NU7441 inhibitor (24 h), were analyzed by WB using indicated antibodies.
K T98G cells were treated or not with CPT for 48 h, in presence or not of NU7441 inhibitor (24 h), prior to immunofluorescence analysis using dsDNA- and 53BP1-specific antibodies and DAPI nuclear staining ($n = 3$ independent experiments). Scale bar, 20 μm.
L T98G cells were treated or not with 25 μM etoposide (ETO) for 72 h, in presence or not of NU7441, prior to assessment of *IFNB* and *CXCL10* mRNA levels by RT–qPCR. Graphs present a representative biological triplicate ($n = 3$ independent experiments).

Data information: All immunoblots show representative experiments ($n = 3$ independent experiments). All graphs present means ± standard error from the mean (SEM). *P*-values were determined by Student's *t*-test. *$P < 0.05$, ***$P < 0.001$, ****$P < 0.0001$.
Source data are available online for this figure.

© 2022 The Authors

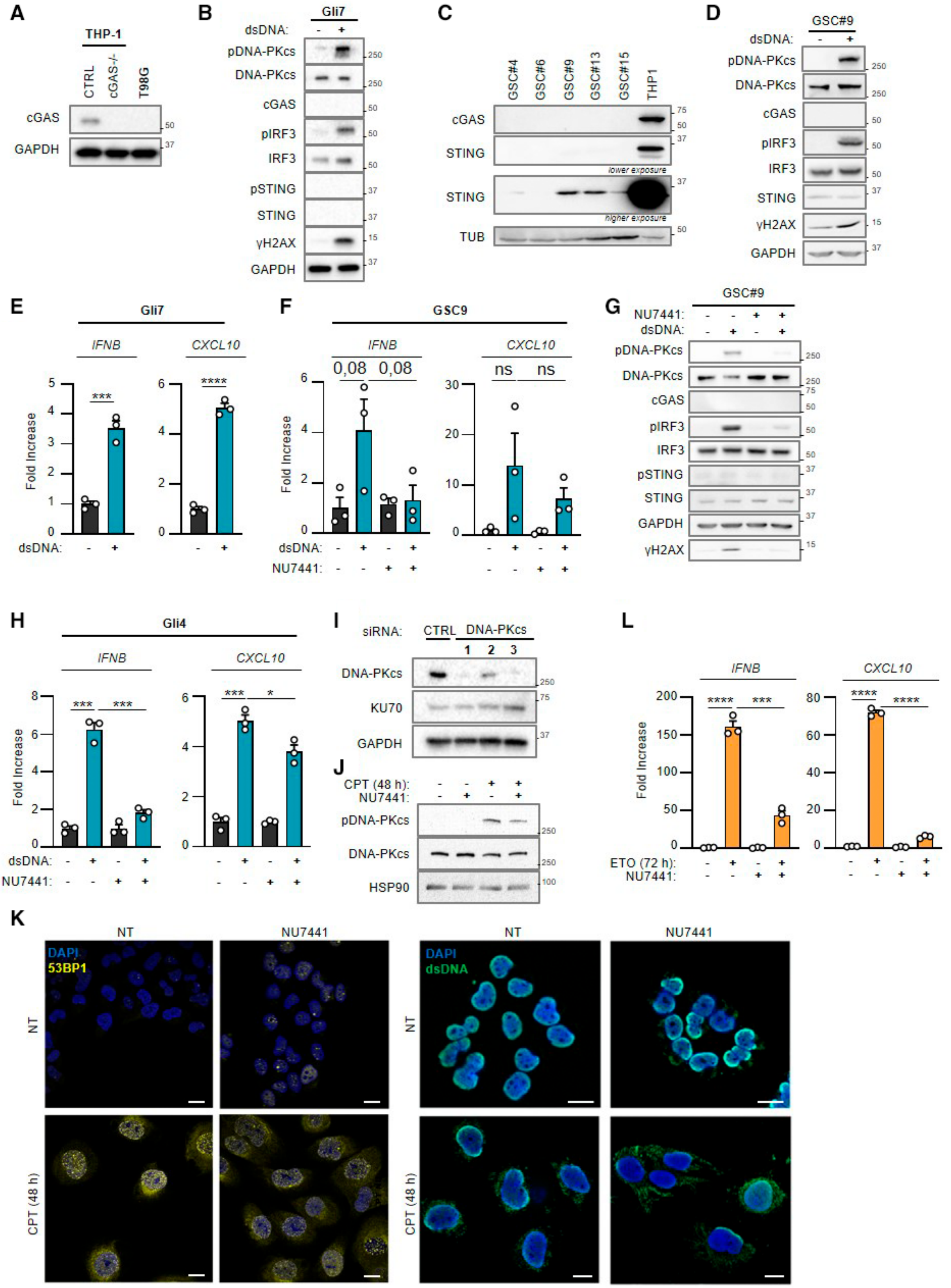

Figure EV1.

© 2022 The Authors

Figure EV2. DNA-PK-dependent detection of cytosolic dsDNA drives STING-independent IRF3-dependent type I IFN responses in cancer cells lacking cGAS.

A Gli7 cells were treated or not with dsDNA for 6 h prior to immunofluorescence analysis using pDNA-PKcs-specific antibody and DAPI nuclear staining ($n = 3$ independent experiments). Scale bar, 20 μm.

B THP-1 cells were transfected or not with 80 nt-long biotinylated dsDNA prior to pull-down using streptavidin-affinity beads. Input and eluates were analyzed by WB using indicated antibodies.

C Whole cell extracts from control HCT116 (CTRL) and HCT116$^{PRKDC-/-}$ were used in pull-down experiments using biotinylated dsDNA and streptavidin affinity beads. Inputs and eluates were analyzed by WB using indicated antibodies.

D T98G cells were treated with non-targeting (CTRL) or KU70-targeting siRNAs prior to whole cell extraction and analysis by WB using indicated antibodies.

E Whole cell extracts from CTRL or *STING*$^{-/-}$ T98G cells transfected or not with 2′3′cGAMP were analyzed by WB using indicated antibodies.

F *IFNB* and *CXCL10* mRNA levels were analyzed by RT–qPCR in samples treated as in (E). Graphs present a representative biological triplicate ($n = 3$ independent experiments).

G Whole cell extracts from CTRL or *STING*$^{-/-}$ T98G cells transfected or not with dsDNA were analyzed by WB using indicated antibodies.

H Whole cell extracts from CTRL or *IRF3*$^{-/-}$ T98G cells transfected or not with dsDNA were analyzed by WB using indicated antibodies.

I Whole cell extracts from CTRL or *STING*$^{-/-}$ T98G cells treated or not with CPT for 72 h were analyzed by WB using indicated antibodies.

J Whole cell extracts from CTRL or *IRF3*$^{-/-}$ T98G cells treated or not with CPT for 72 h were analyzed by WB using indicated antibodies.

K THP-1^{CTRL}, THP-1$^{cGAS-/-}$ and THP-1$^{STING-/-}$ were challenged or not with dsDNA for 6 h, prior to WB analysis using indicated antibodies.

L *IFNB* and *CXCL10* mRNA levels were analyzed by RT–qPCR in samples treated as in (K). Graphs present a representative biological triplicate ($n = 3$ independent experiments).

Data information: All immunoblots are representative experiments ($n = 3$ independent experiments). All graphs present means ± SEM. *P*-values were determined by Student's *t*-test. ns: not significant. *$P < 0.05$, **$P < 0.01$, ***$P < 0.001$, ****$P < 0.0001$.
Source data are available online for this figure.

© 2022 The Authors

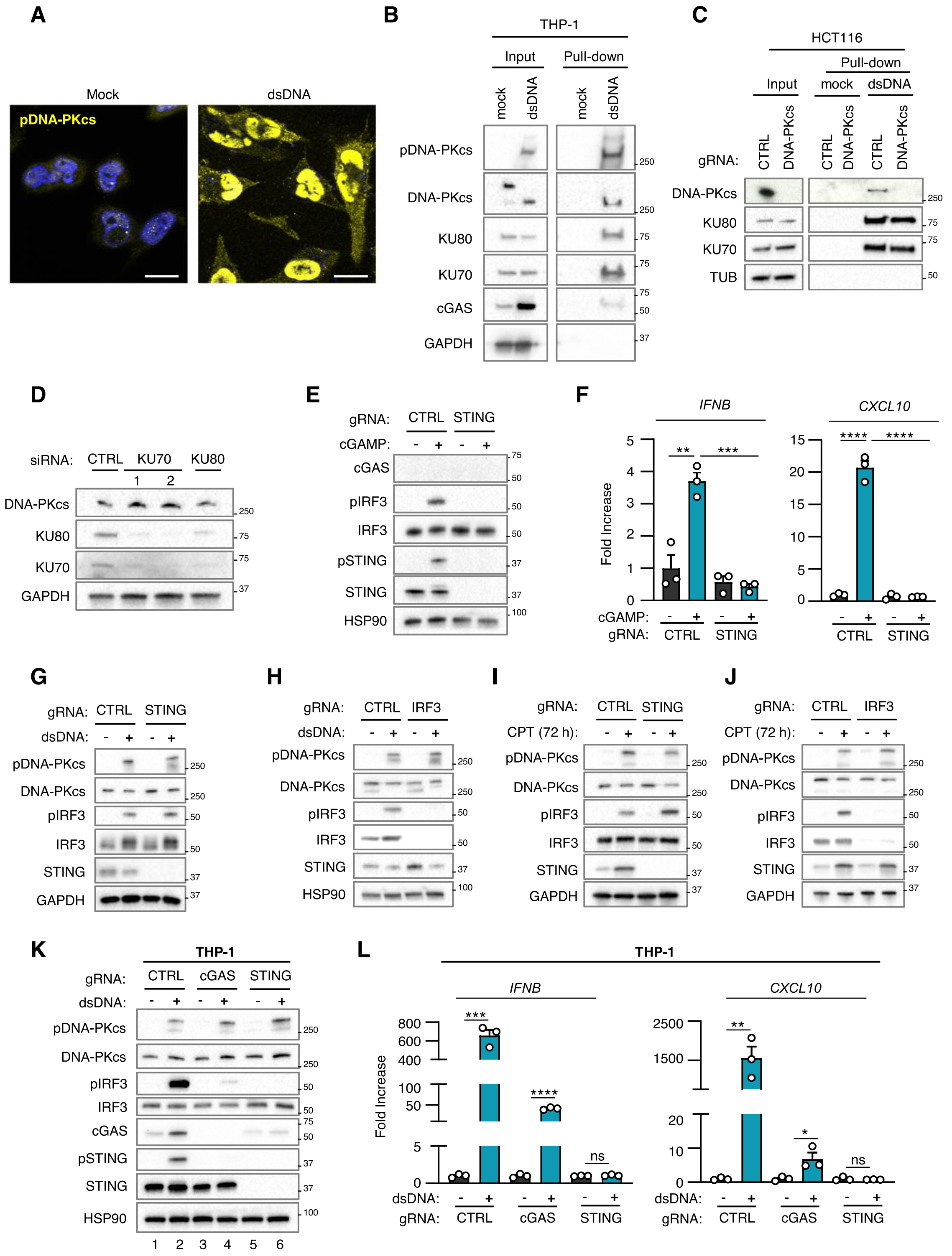

Figure EV2.

 © 2022 The Authors

Figure EV3. DNA-PKcs and cGAS synergize for the activation of type I Interferon responses.

A T98G cells expressing a cGAS-encoding vector (T98G^{cGAS}) were treated or not with dsDNA for 6 h prior to IF analysis using pDNA-PKcs-specific antibody and DAPI nuclear staining (n = 3 independent experiments). Scale bar, 20 μm.

B THP-1 and THP-1$^{cGAS-/-}$ were transfected or not with 80-nt long biotinylated dsDNA prior to whole cell extraction and pull-down using streptavidin-affinity beads. Inputs and eluates were analyzed by WB using indicated antibodies.

C T98G cells expressing an empty (T98G^{Empty}) or T98G^{cGAS} were transfected or not with 5, 10 or 20 μg of biotinylated dsDNA prior to whole cell extraction and pull-down using streptavidin-affinity beads. Inputs and eluates were analyzed by WB using indicated antibodies.

D T98G^{Empty} or T98G^{cGAS} were transfected or not with dsDNA for 6 h in the presence or not of the NU7441 DNA-PKcs inhibitor. Whole cell extracts were analyzed by WB using indicated antibodies.

E THP-1^{CTRL} and THP-1$^{cGAS-/-}$ were transfected or not with dsDNA for 6 h in presence or not of the NU7441 DNA-PKcs inhibitor prior to analysis of protein expression by WB using indicated antibodies.

F CFPAC and CFPAC$^{cGAS-/-}$ were transfected or not with dsDNA for 6 h in presence or not of the NU7441 DNA-PKcs inhibitor prior to WB analysis using indicated antibodies.

G *IFNB* and *CXCL10* mRNA levels were assessed by RT–qPCR in CFPAC^{CTRL} and CFPAC$^{cGAS-/-}$ treated as in D. Graphs present a representative biological triplicate (n = 3 independent experiments).

H As in (F), except that MEF and MEF$^{cGas-/-}$ were transfected.

I As in (G), except that MEF and MEF$^{cGas-/-}$ were transfected. Graphs present a representative biological triplicate (n = 3 independent experiments).

J GL261^{CTRL} and GL261$^{cGAS-/-}$ were transfected or not with dsDNA for 6 h in presence or not of the NU7441 DNA-PKcs inhibitor. Whole cell extracts were analyzed by WB using indicated antibodies. *IFNB* and *CXCL10* mRNA levels were assessed by RT–qPCR. Graphs present a representative biological triplicate (n = 3 independent experiments).

K cGAS activity upon treatment with 2.5 μM of the CU76 cGAS inhibitor and 2, 4, 6, or 8 μM of NU7441 was measured by ELISA (n = 3 independent experiments).

L T98G^{Empty}, T98G^{cGAS} and T98G$^{cGAS\text{-}CD}$ were transfected or not with dsDNA for 6 h prior to analysis of protein expression by WB using indicated antibodies.

Data information: All graphs present means ± SEM. All immunoblots show representative experiments (n = 3 independent experiments). P-values were determined by Student's t-test. ns: not significant. $^*P < 0.05$, $^{**}P < 0.01$, $^{***}P < 0.001$, $^{****}P < 0.0001$.
Source data are available online for this figure.

© 2022 The Authors

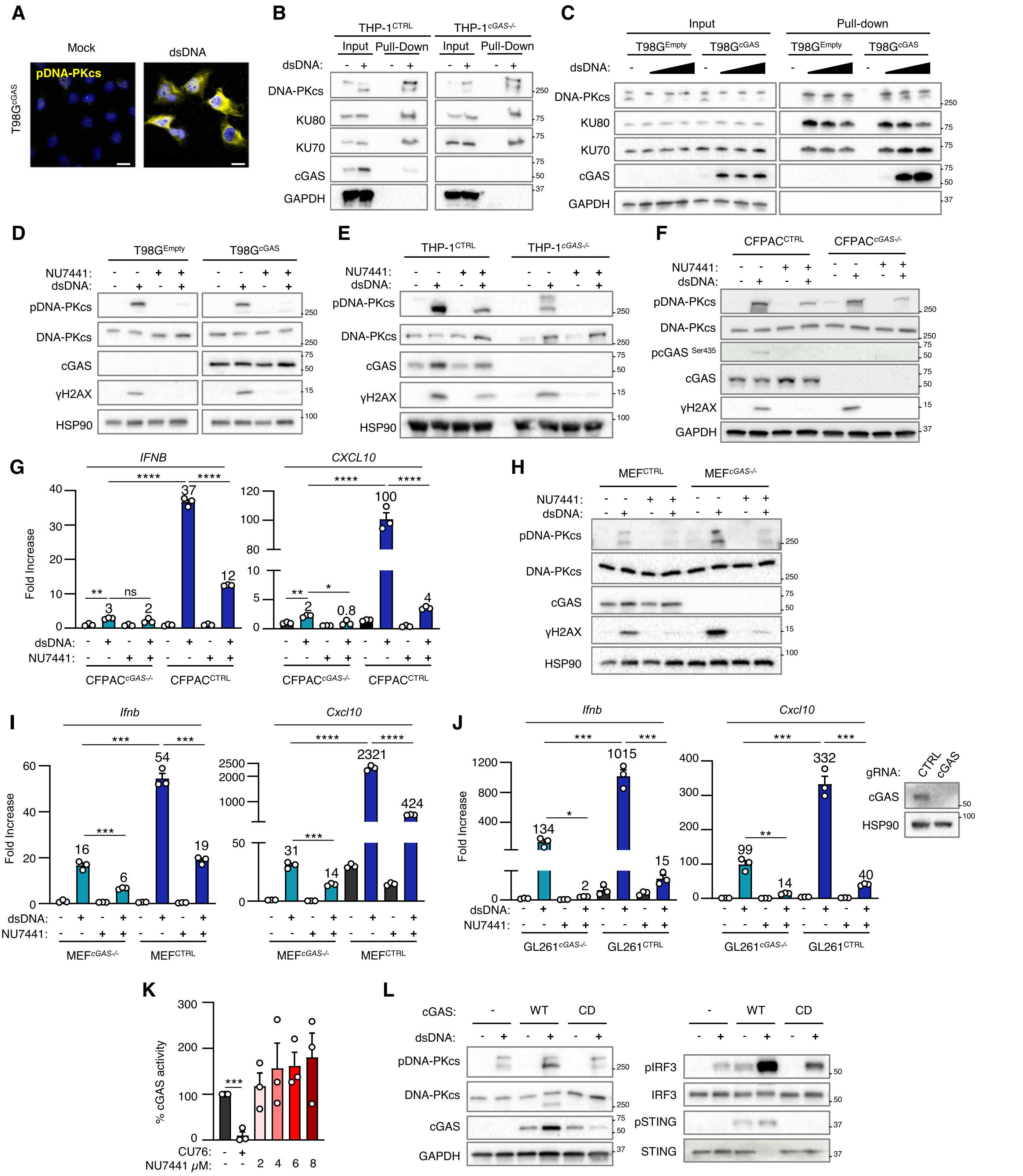

Figure EV3.

 © 2022 The Authors

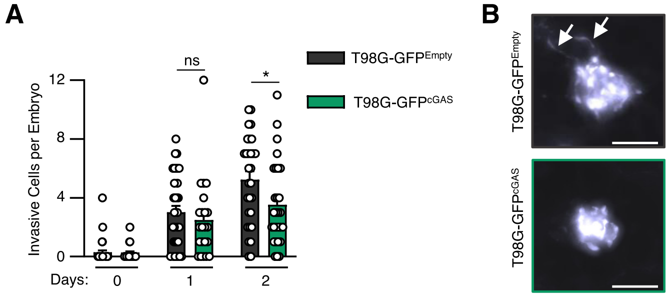

Figure EV4. Re-expression of cGAS in glioblastoma cells decrease invasiveness *in vivo*.

A The number of invasive cells per embryo transplanted in Fig 4G was manually counted at D0, D1 and D2 post transplantation. Data represents mean (± SEM) of $n = 21$ (T98G-GFP^{Empty}) or 29 (T98G-GFP^{cGAS}) embryos. A Mann–Whitney test was performed to assess the significance $^*P < 0.05$.

B Representative images of elongated T98G cells counted in (A). Scale bar, 100 µm. Arrows indicate elongated pseudopodia.

Figure EV5. Re-expression of cGAS in glioblastoma tumors promotes the secretion of chemokines that enhance macrophage recruitment.

A THP-1 monocytes were incubated for 24 h with conditioned media (CM) derived from T98G^{Empty} and T98G^{cGAS} cells, prior to analyses of *CD197*, *CD80*, *CXCL10*, *CD209* and *Fibronectin* gene expression, by RT–qPCR ($n = 4$ independent experiments).

B CD14^{+} monocytes derived from healthy donors were incubated for 72 h with conditioned media derived from T98G^{Empty} and T98G^{cGAS} cells, prior to flow cytometry analysis of M1 (CD86, CD38) and M2 (CD206, CD209) polarization markers ($n = 5$ donors).

C T98G^{Empty}, T98G^{cGAS} and T98G$^{cGAS\text{-}CD}$ cells were transfected or not with dsDNA for 6 h, prior to analyses of *CCL2* and *CCL5* mRNA levels by RT–qPCR. Graphs present a representative biological triplicate ($n = 3$ independent experiments).

D T98G^{Empty} and T98G^{cGAS} were transfected or not with dsDNA for 6 h in the presence or not of NU7441 prior to *CCL2* and *CCL5* expression analysis. Graphs present a representative biological triplicate ($n = 3$ independent experiments).

E As in (D), except that *IFNL2/3*, *CCL3*, *IL6* expressions were analyzed ($n = 3$ independent experiments).

Data information: All graphs present means ± SEM. *P*-values were determined by Student's *t*-test. ns: not significant. $^*P < 0.05$, $^{**}P < 0.01$, $^{***}P < 0.001$, $^{****}P < 0.0001$.

© 2022 The Authors

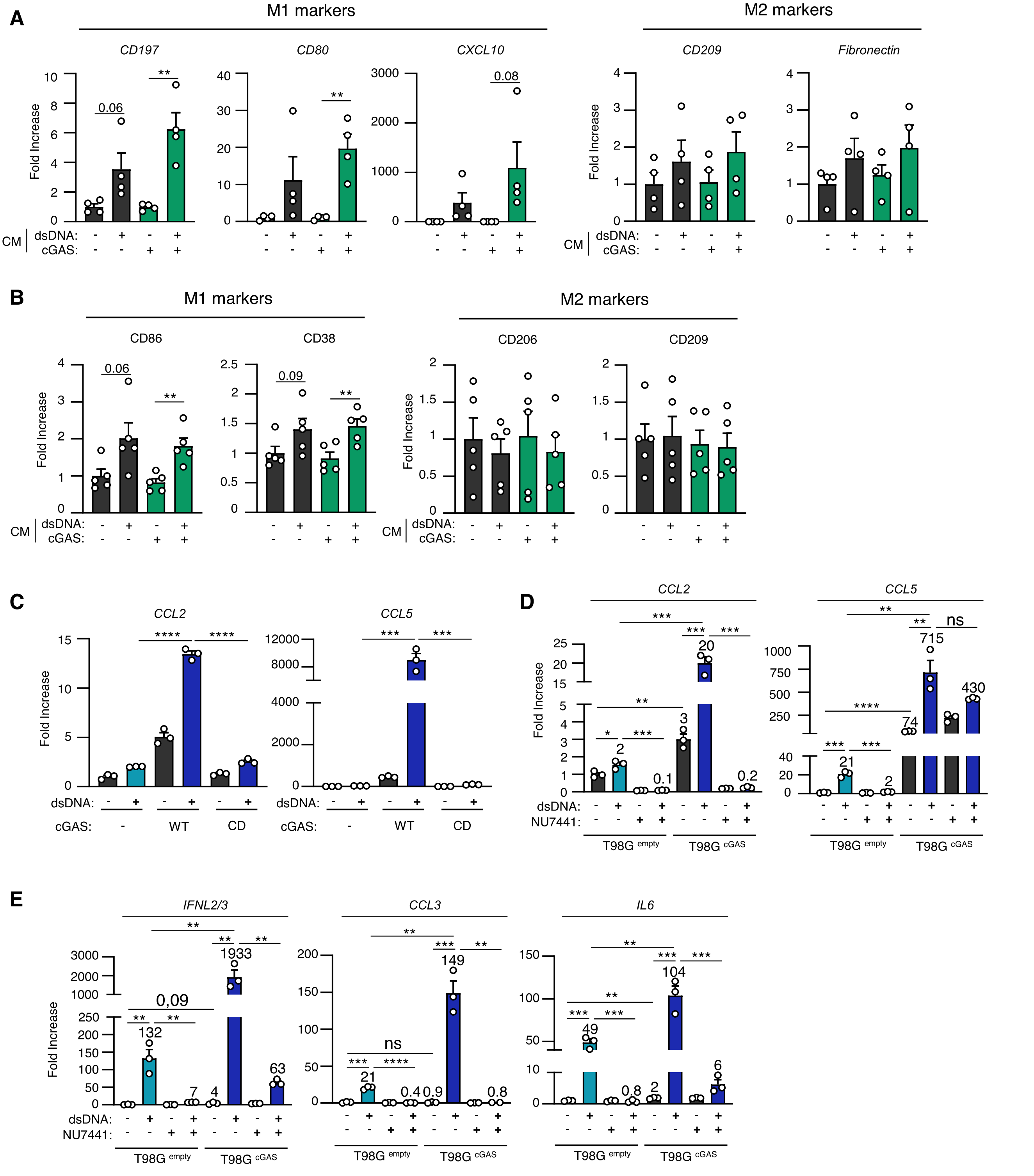

Figure EV5.

 © 2022 The Authors
