# Supplementary figures and images for: DNA damage repair kinase DNA‐PK and cGAS synergize to induce cancer‐related inflammation in glioblastoma (part 2 of 2)
Source: EMBO J. 2022 Dec 27;42(7):e111961. doi: 10.15252/embj.2022111961 (PMC10068334; doi:10.15252/embj.2022111961)

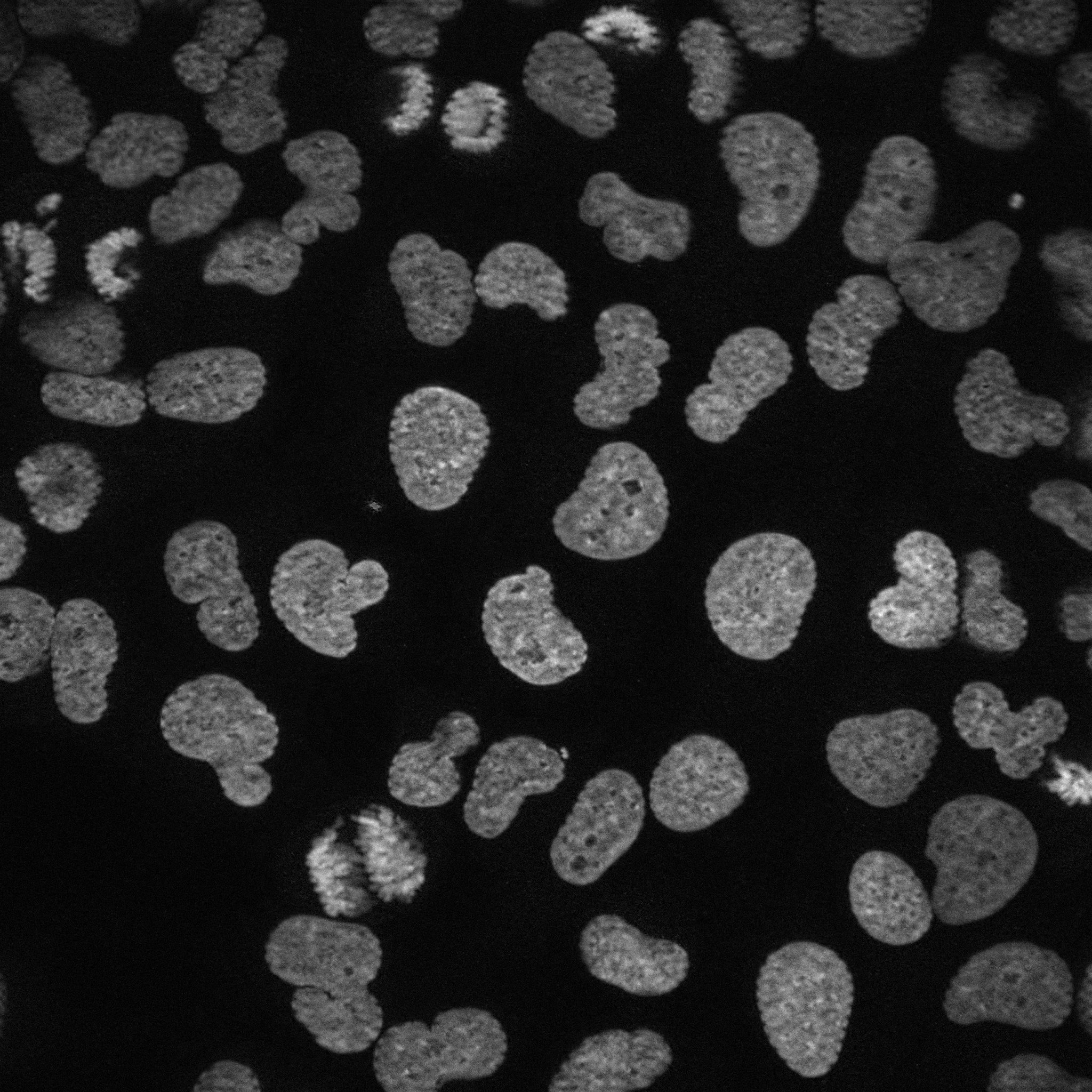

Supplement: Supplementary file 5 — Source Data for Figure 1 [file EMBJ-42-e111961-s009.zip › Figure 1/Figure 1I/Fig 1I_IF_53BP1_Dmso.tif]

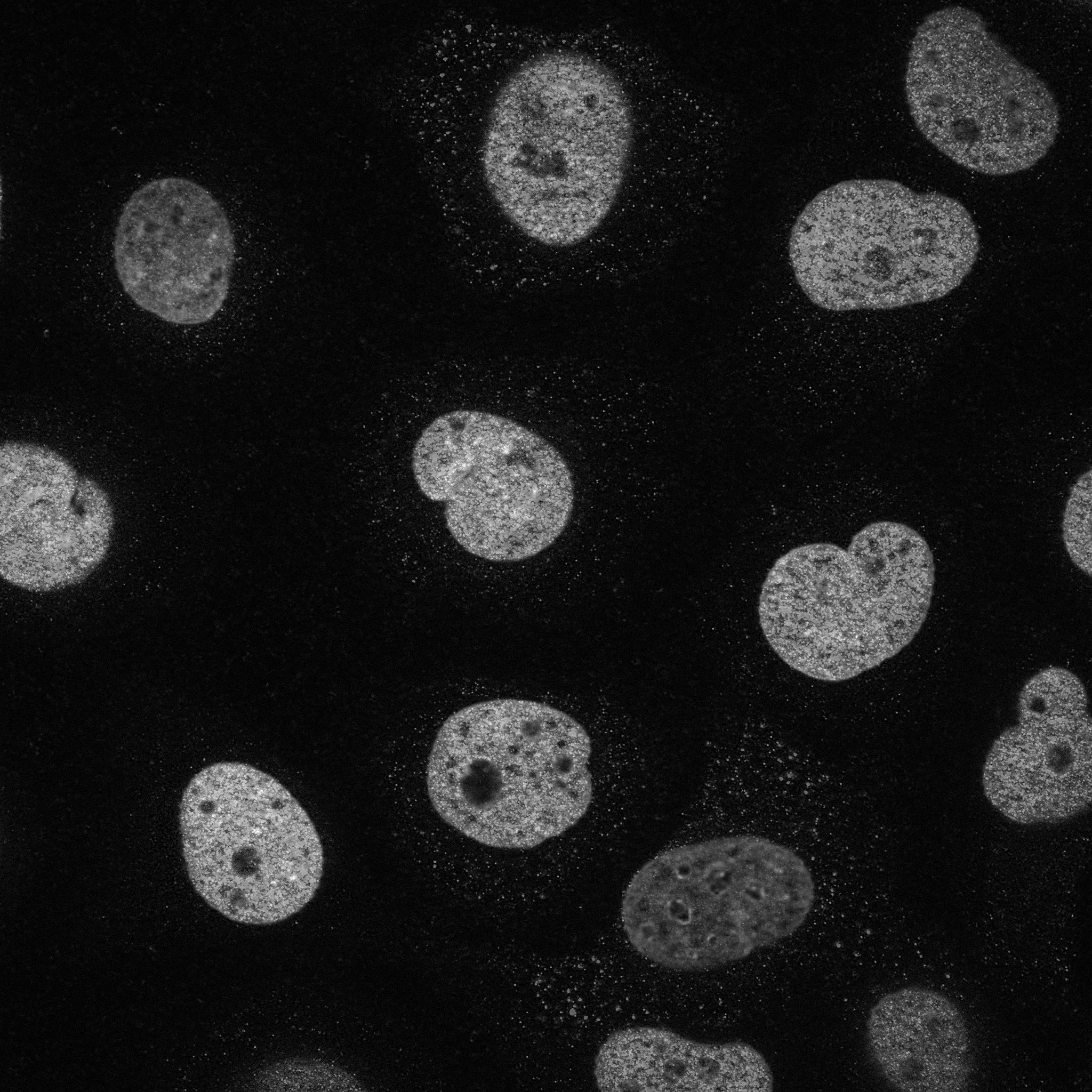

Supplement: Supplementary file 5 — Source Data for Figure 1 [file EMBJ-42-e111961-s009.zip › Figure 1/Figure 1I/Fig 1I_IF_53BP1_CPT.tif]

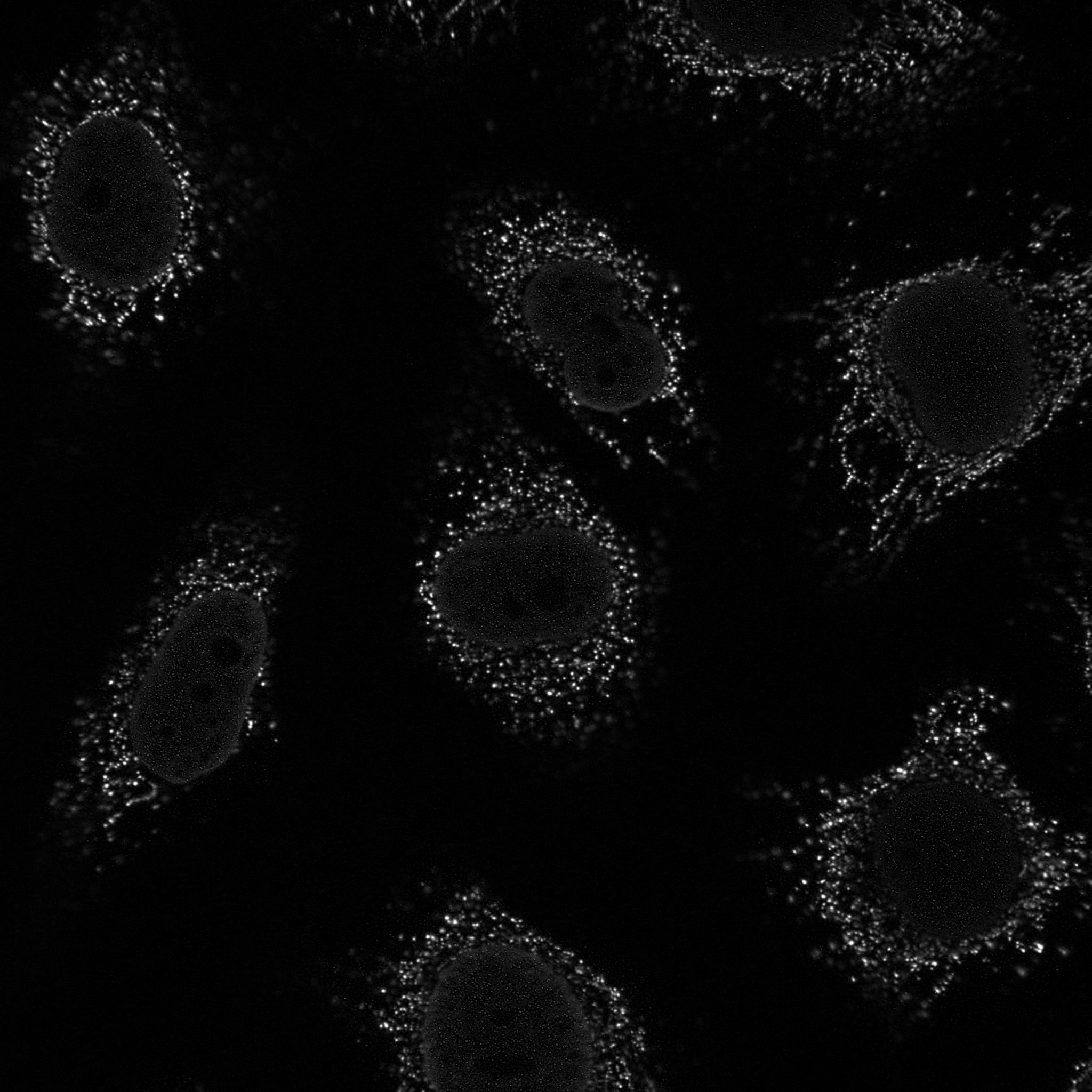

Supplement: Supplementary file 5 — Source Data for Figure 1 [file EMBJ-42-e111961-s009.zip › Figure 1/Figure 1I/Fig 1I_IF_dsDNA_CPT.tif]

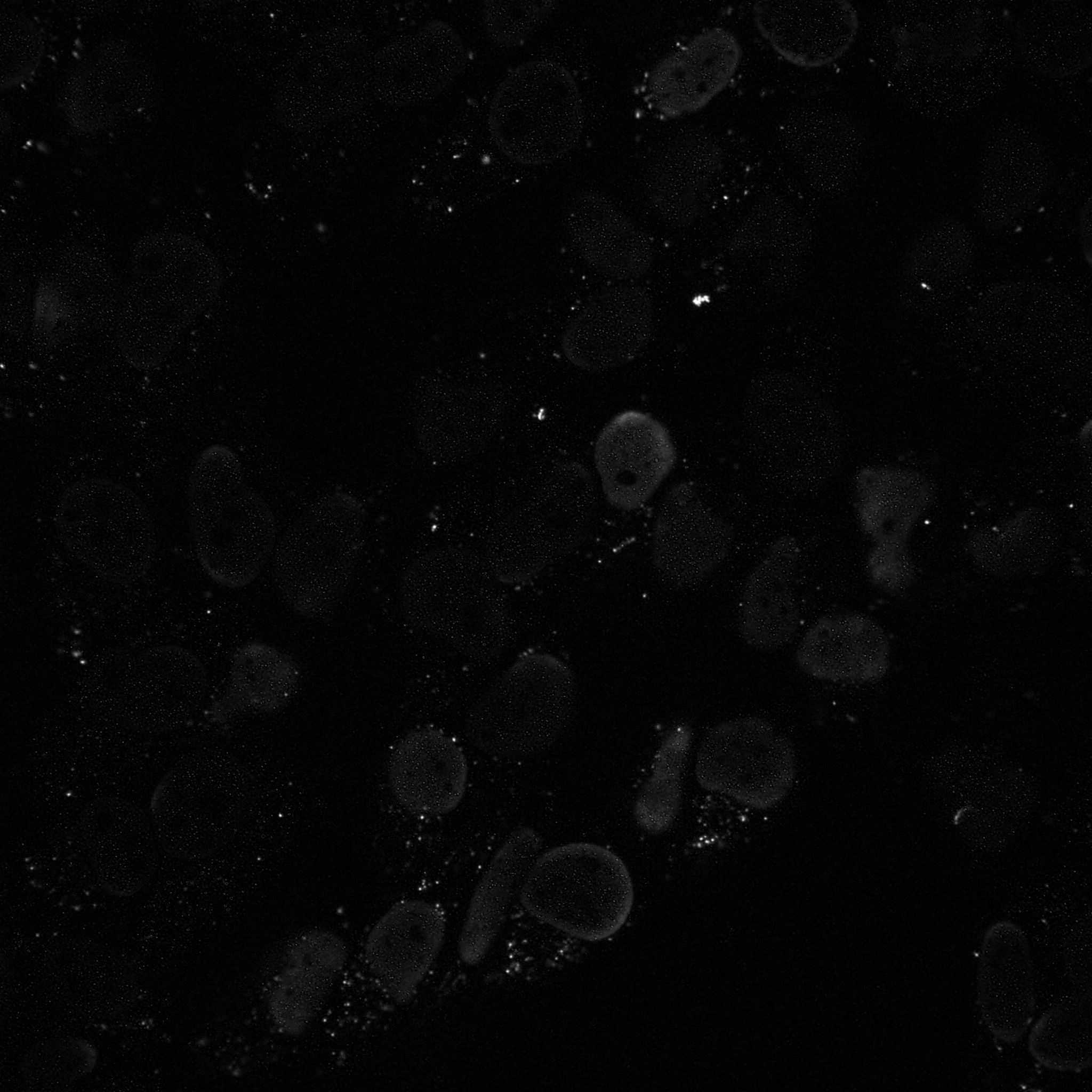

Supplement: Supplementary file 5 — Source Data for Figure 1 [file EMBJ-42-e111961-s009.zip › Figure 1/Figure 1I/Fig 1I_IF_dsDNA_Dmso.tif]

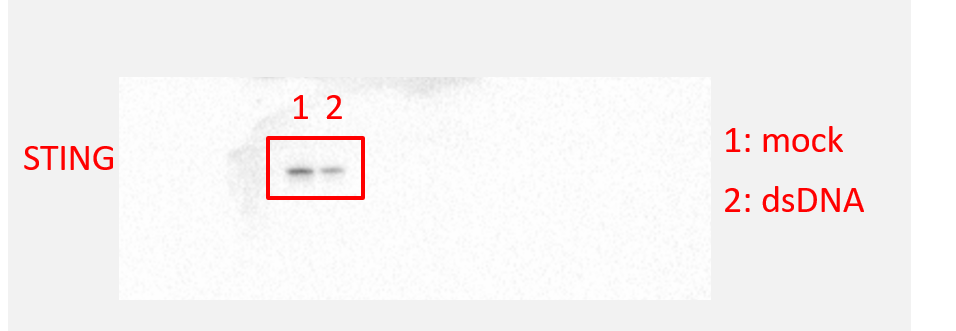

Supplement: Supplementary file 5 — Source Data for Figure 1 [file EMBJ-42-e111961-s009.zip › Figure 1/Figure 1C/Fig 1C_western_STING.tif]

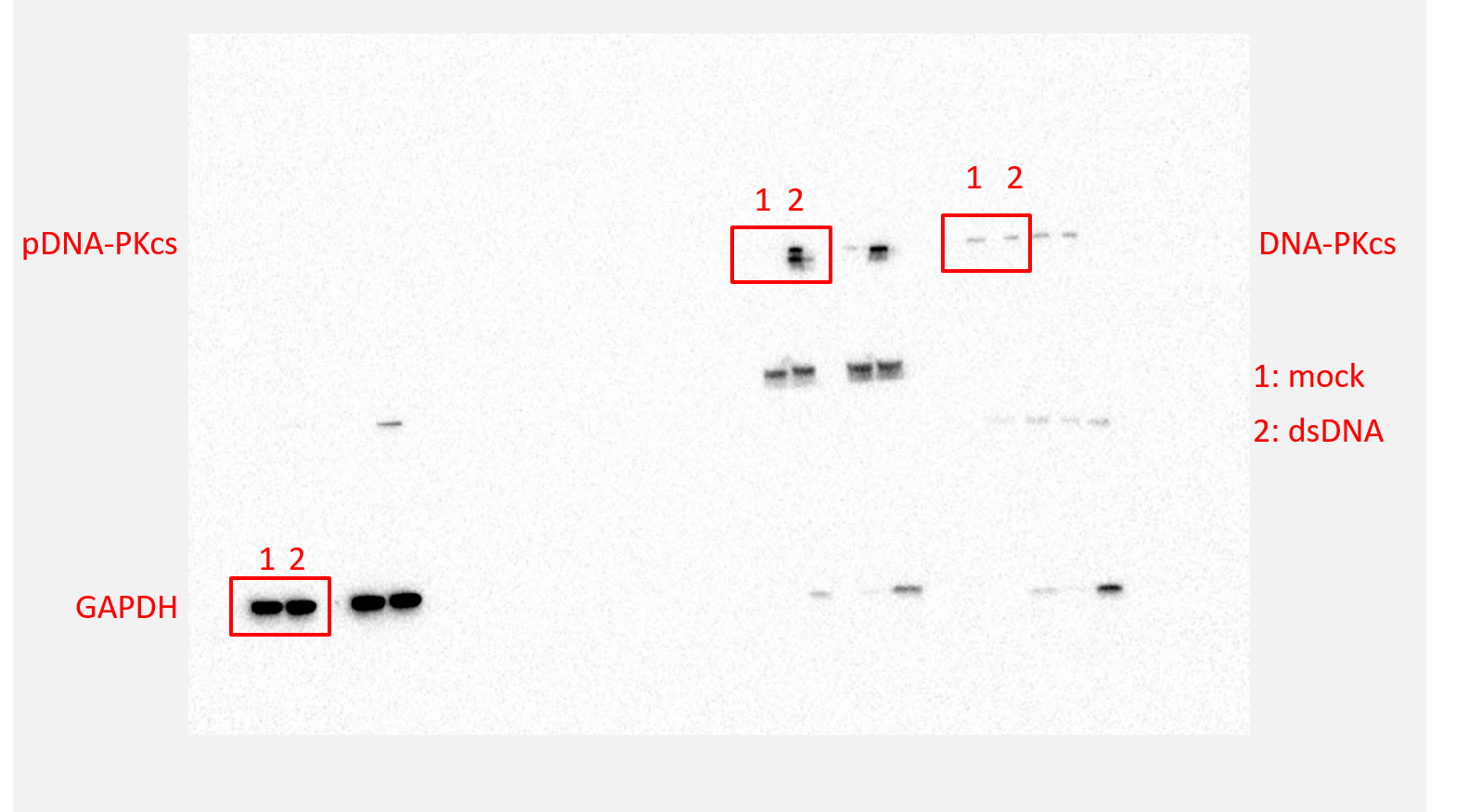

Supplement: Supplementary file 5 — Source Data for Figure 1 [file EMBJ-42-e111961-s009.zip › Figure 1/Figure 1C/Fig 1C_western_DNA-PKcs, pDNA-PKcs & GAPDH.tif]

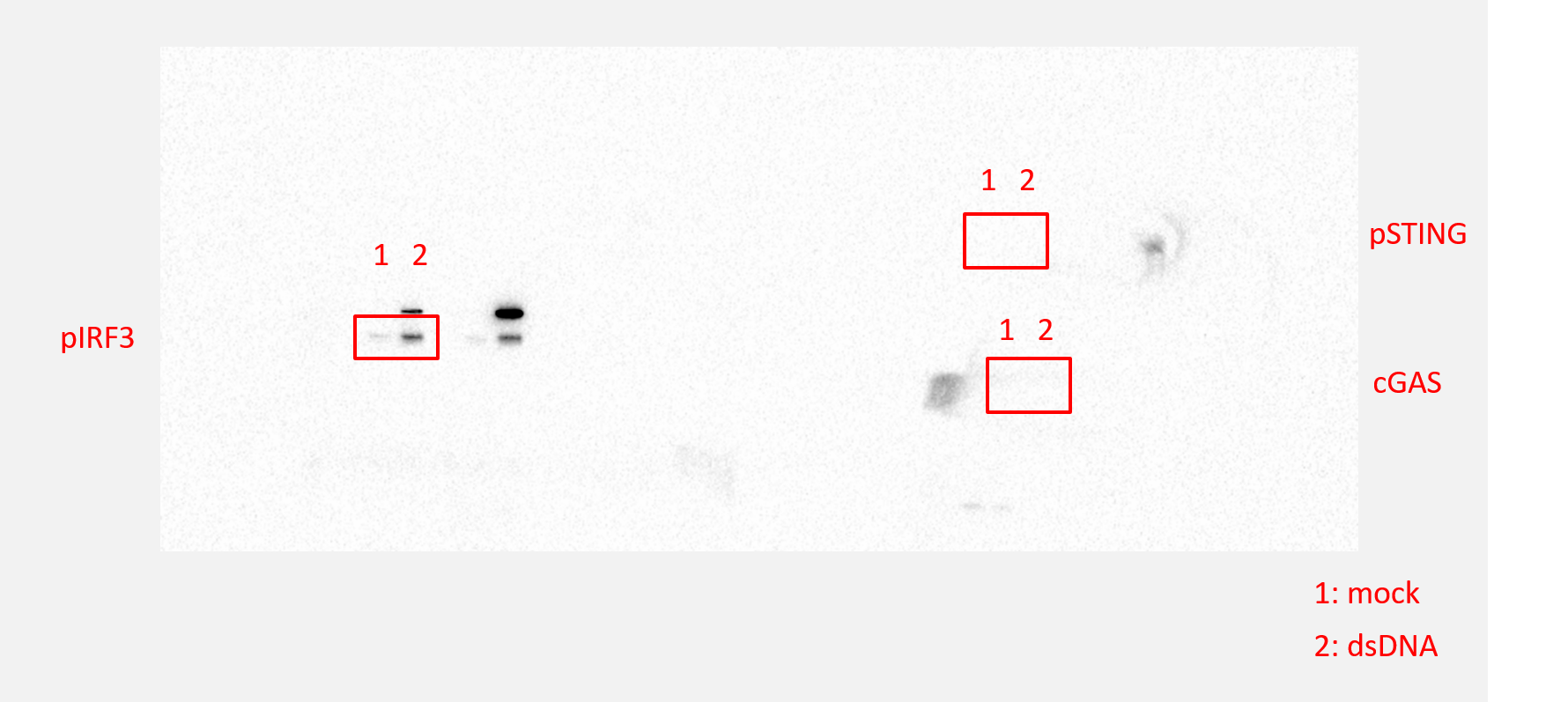

Supplement: Supplementary file 5 — Source Data for Figure 1 [file EMBJ-42-e111961-s009.zip › Figure 1/Figure 1C/Fig 1C_western_pIRF3, pSTING & cGAS.tif]

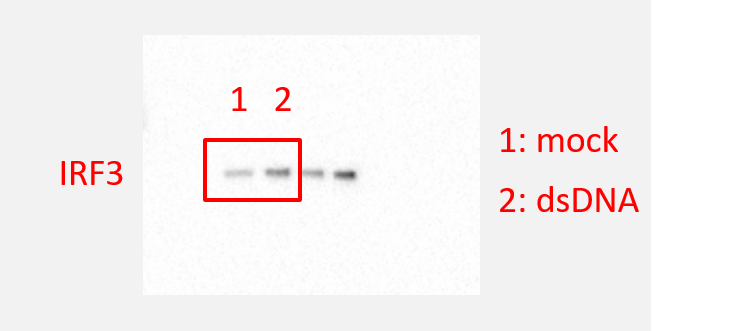

Supplement: Supplementary file 5 — Source Data for Figure 1 [file EMBJ-42-e111961-s009.zip › Figure 1/Figure 1C/Fig 1C_western_IRF3.tif]

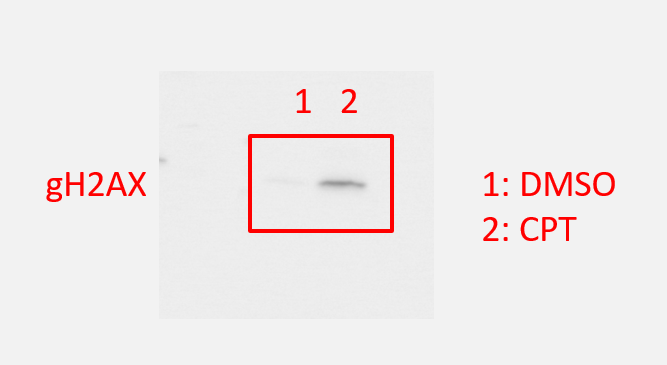

Supplement: Supplementary file 5 — Source Data for Figure 1 [file EMBJ-42-e111961-s009.zip › Figure 1/Figure 1J/Fig 1J_western_gH2AX.tif]

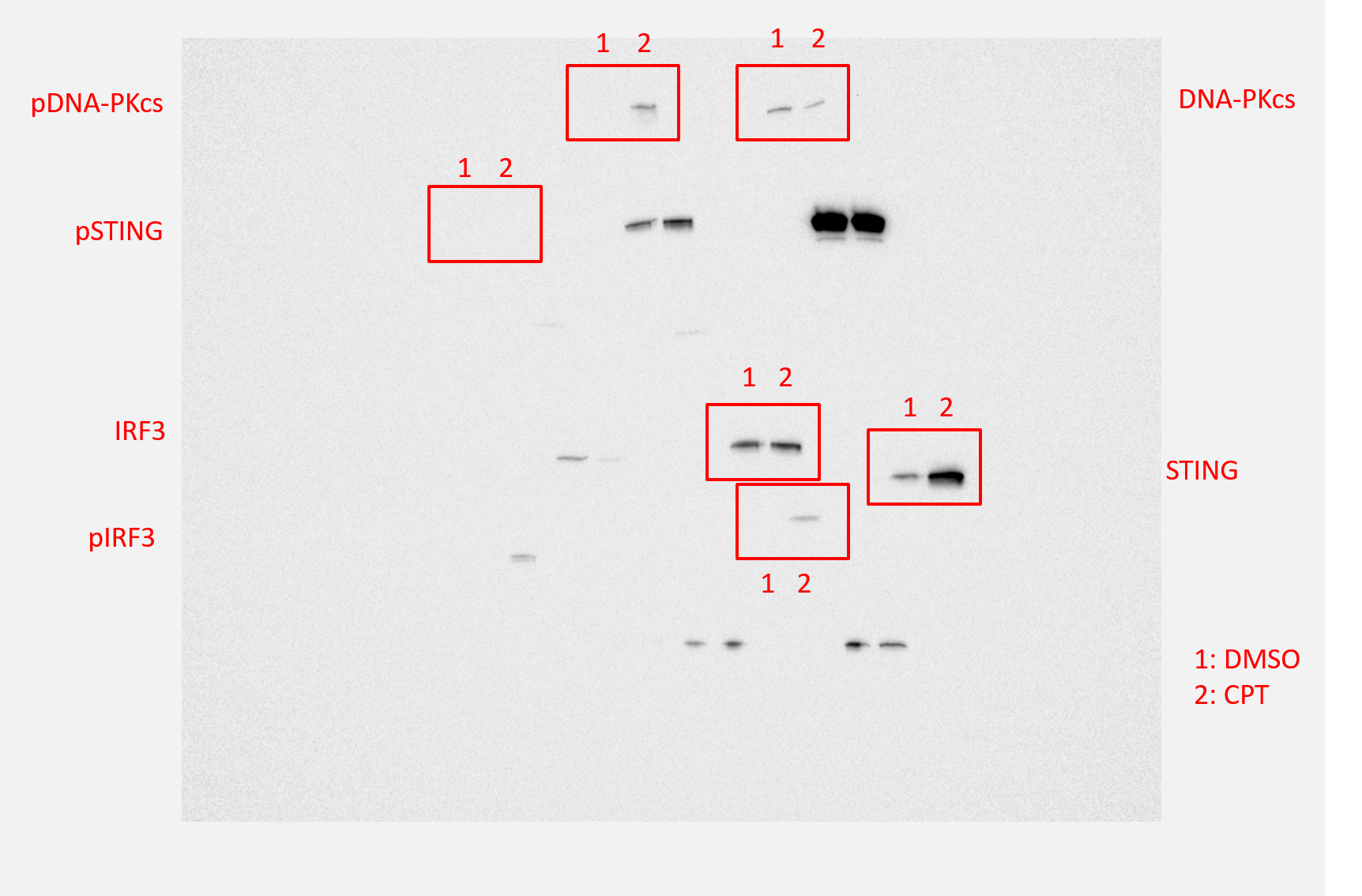

Supplement: Supplementary file 5 — Source Data for Figure 1 [file EMBJ-42-e111961-s009.zip › Figure 1/Figure 1J/Fig 1J_western_pDNA-PKcs, pSTING,IRF3, pIRF3,STING & DNA-PKcs.tif]

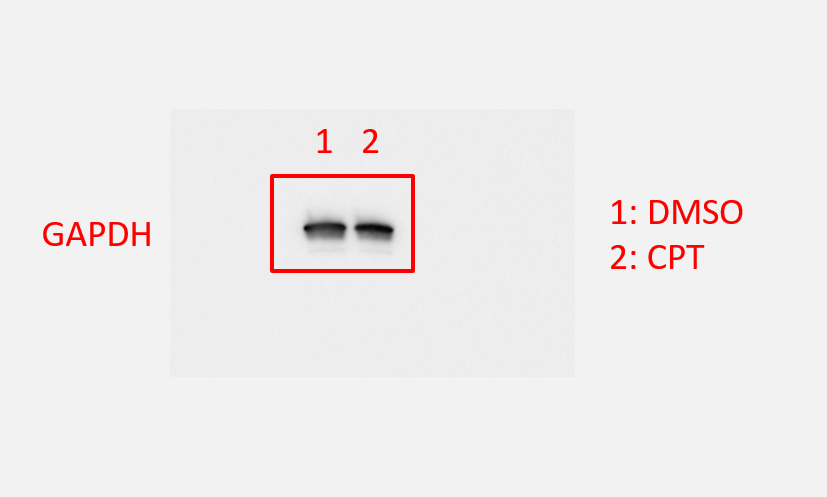

Supplement: Supplementary file 5 — Source Data for Figure 1 [file EMBJ-42-e111961-s009.zip › Figure 1/Figure 1J/Fig 1J_western_GAPDH.tif]

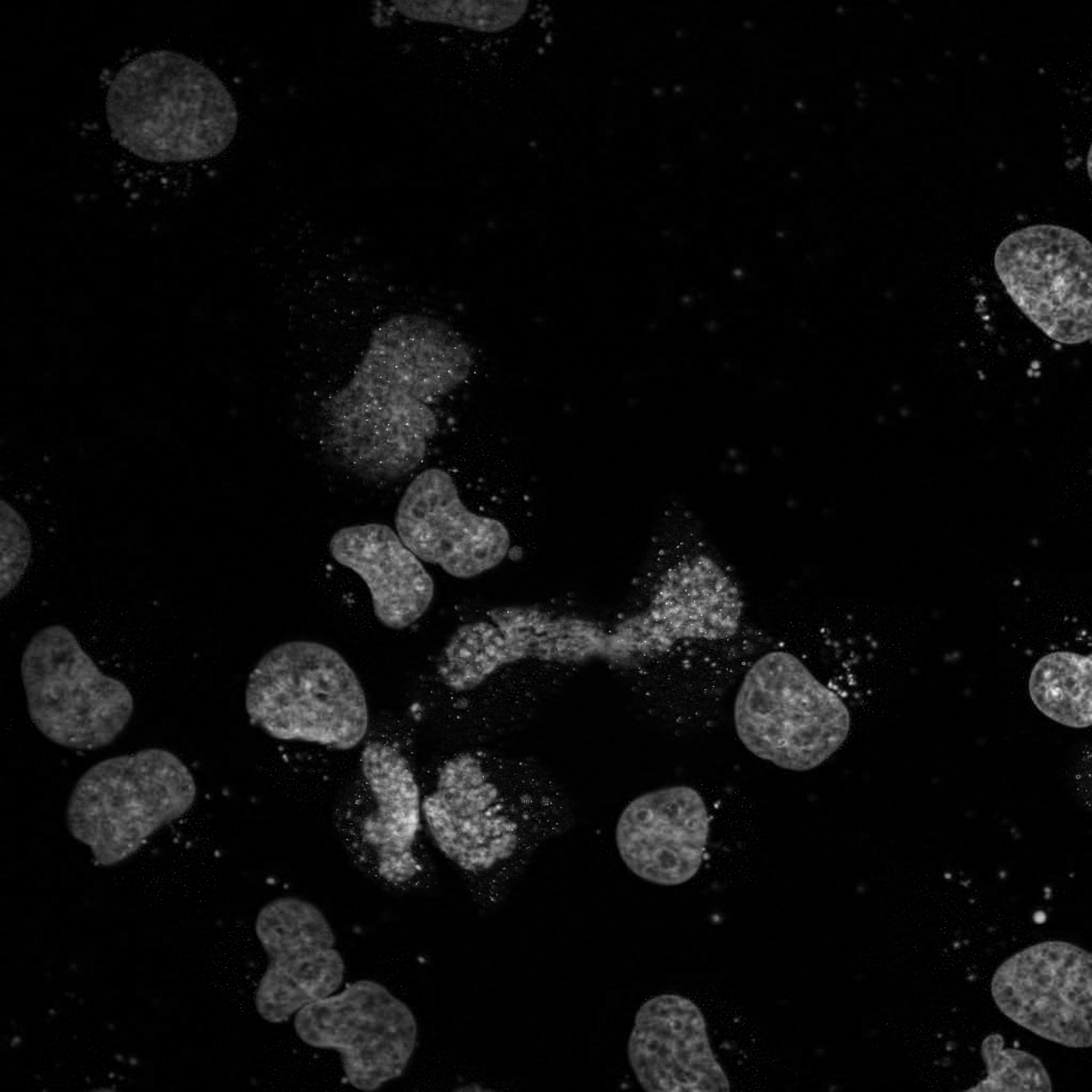

Supplement: Supplementary file 6 — Source Data for Figure 2 [file EMBJ-42-e111961-s001.zip › Figure 2/Figure 2A/Fig 2A_pDNA-PKcs_dsDNA.tif]

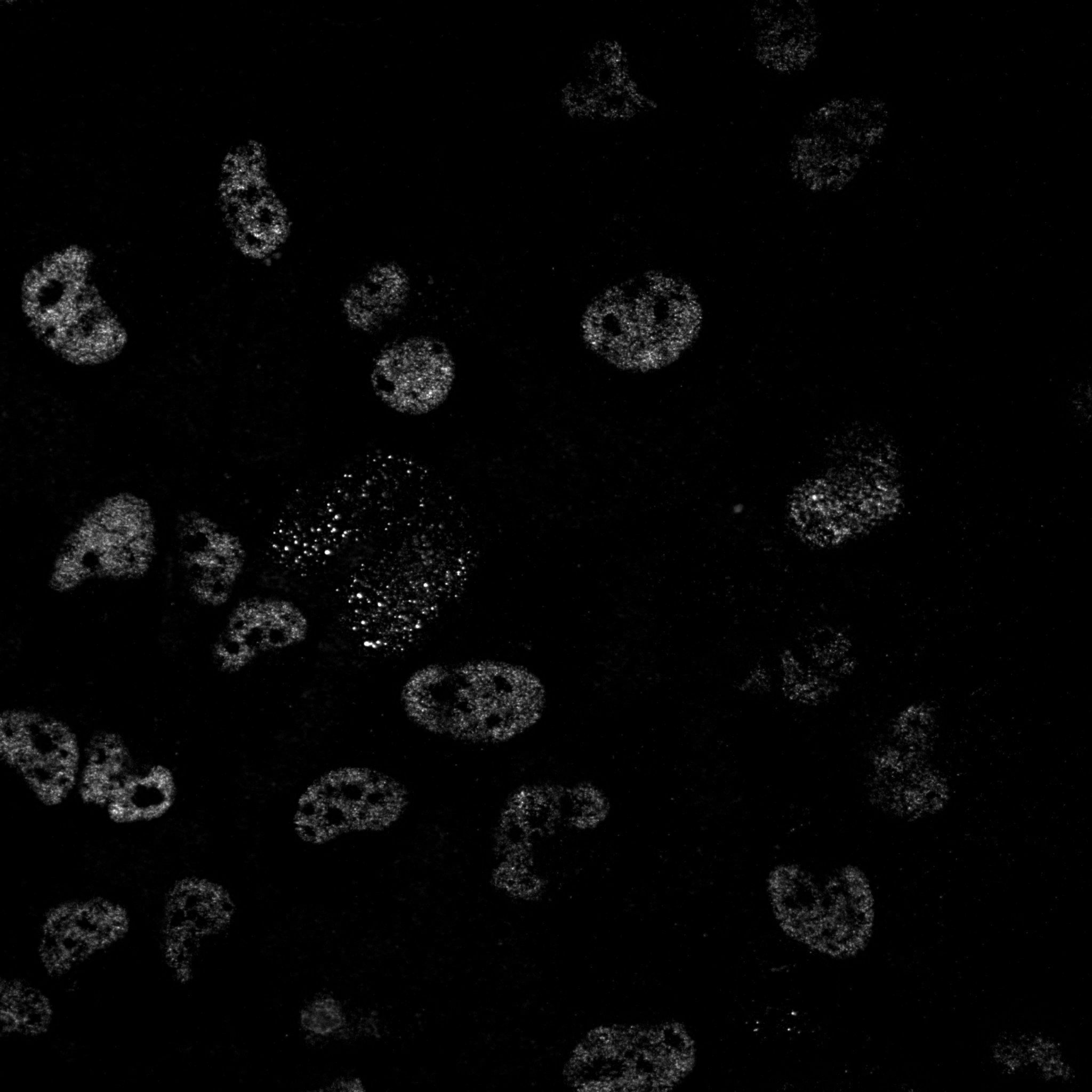

Supplement: Supplementary file 6 — Source Data for Figure 2 [file EMBJ-42-e111961-s001.zip › Figure 2/Figure 2A/Fig 2A_DNA-PKcs_dsDNA.tif]

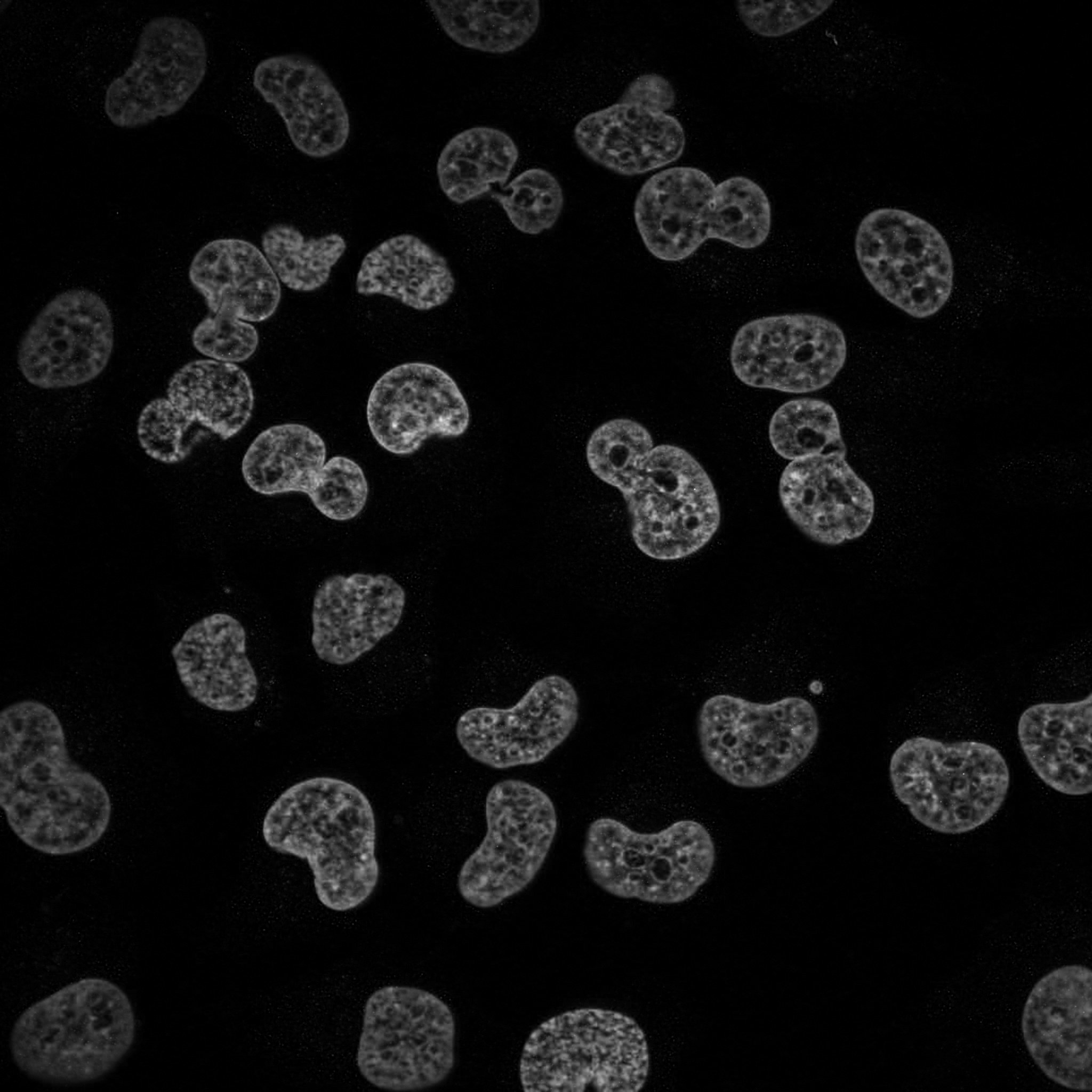

Supplement: Supplementary file 6 — Source Data for Figure 2 [file EMBJ-42-e111961-s001.zip › Figure 2/Figure 2A/Fig 2A_pDNA-PKcs_mock.tif]

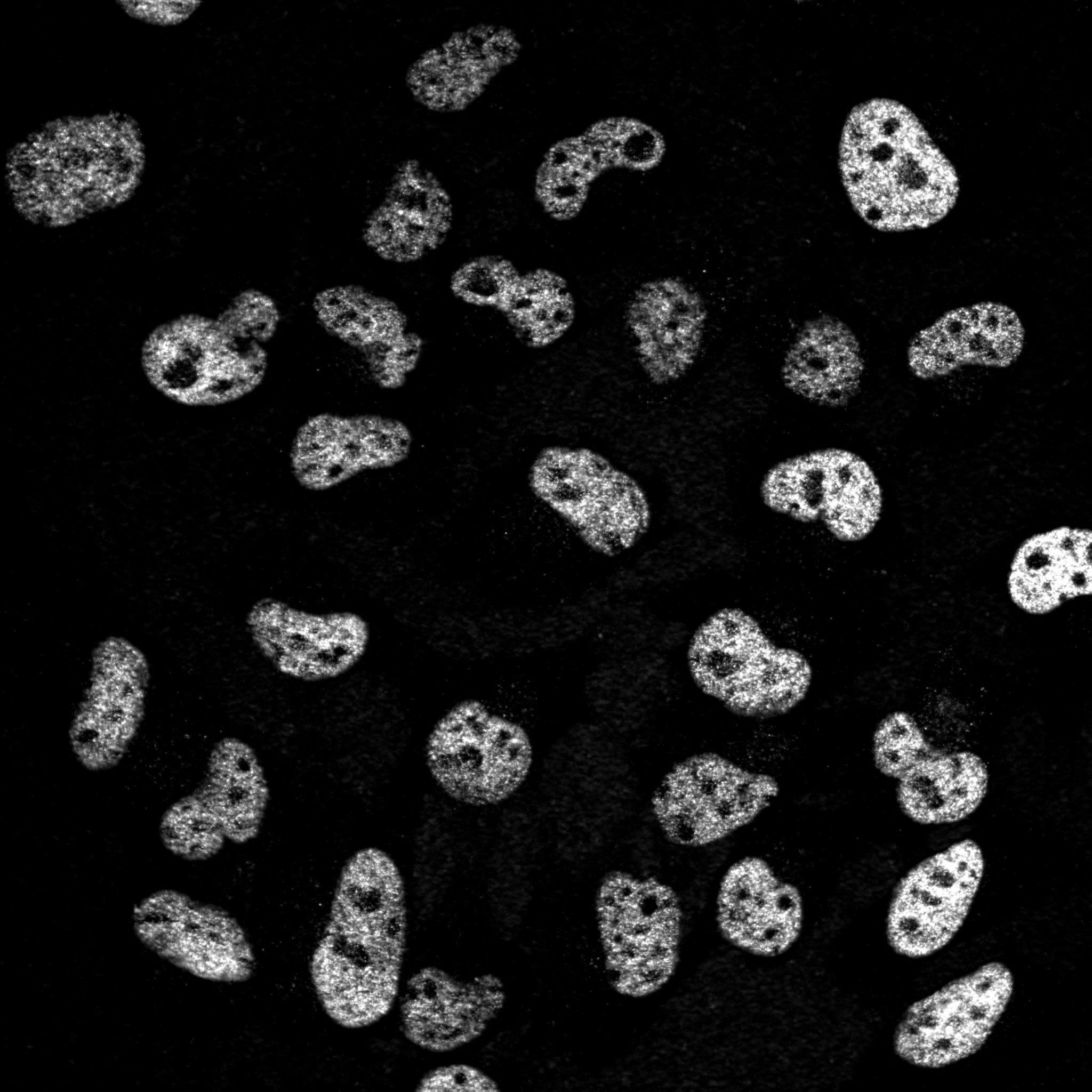

Supplement: Supplementary file 6 — Source Data for Figure 2 [file EMBJ-42-e111961-s001.zip › Figure 2/Figure 2A/Fig 2A_DNA-PKcs_mock.tif]

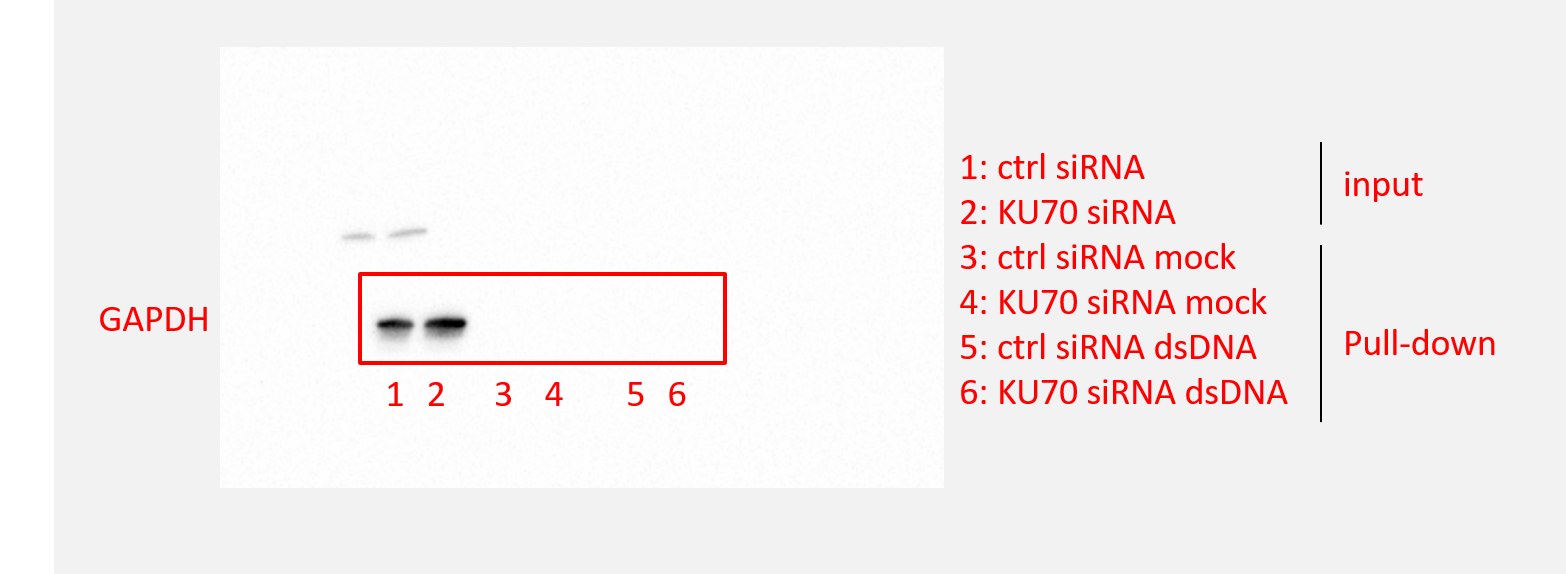

Supplement: Supplementary file 6 — Source Data for Figure 2 [file EMBJ-42-e111961-s001.zip › Figure 2/Figure 2C/Fig 2C_western_GAPDH.tif]

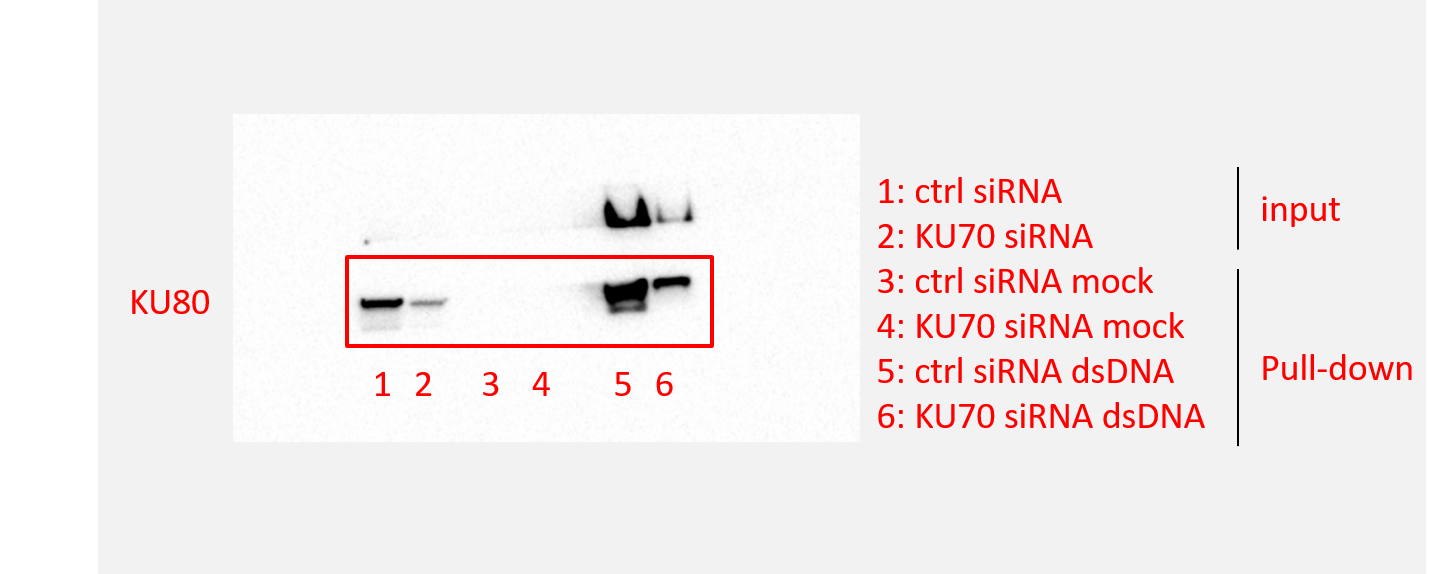

Supplement: Supplementary file 6 — Source Data for Figure 2 [file EMBJ-42-e111961-s001.zip › Figure 2/Figure 2C/Fig 2C_western_KU80.tif]

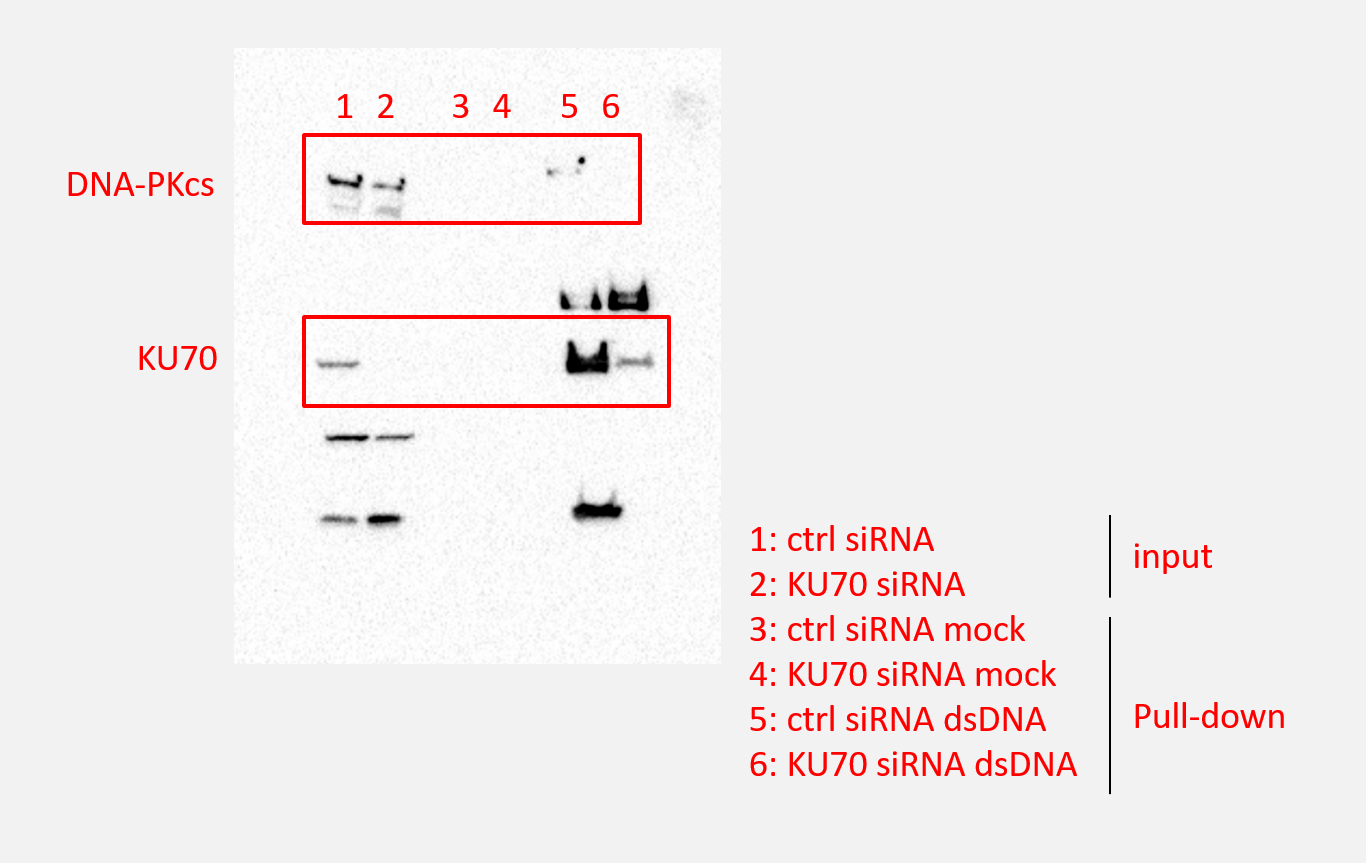

Supplement: Supplementary file 6 — Source Data for Figure 2 [file EMBJ-42-e111961-s001.zip › Figure 2/Figure 2C/Fig 2C_western_DNAPKcs & KU70.tif]

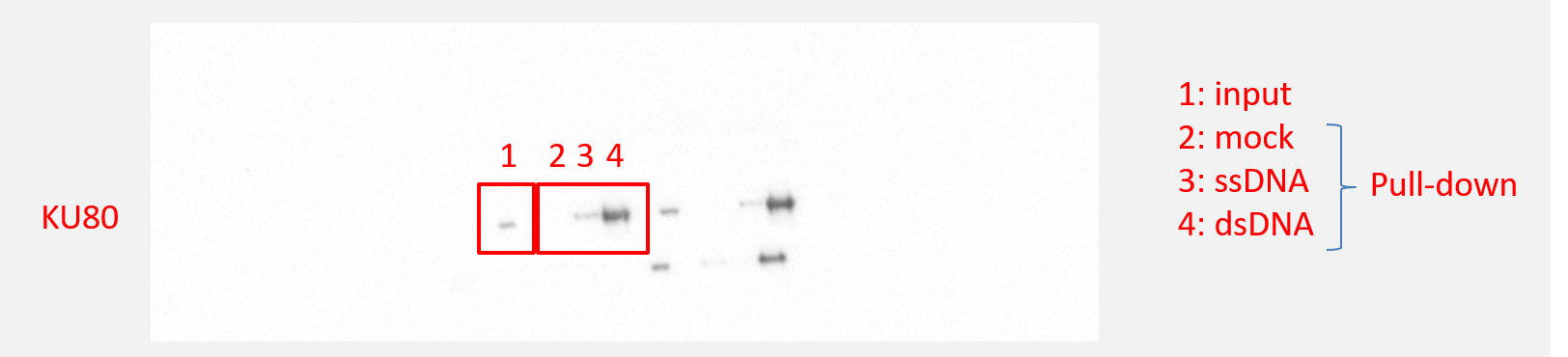

Supplement: Supplementary file 6 — Source Data for Figure 2 [file EMBJ-42-e111961-s001.zip › Figure 2/Figure 2B/Fig 2B_western_KU80.tif]

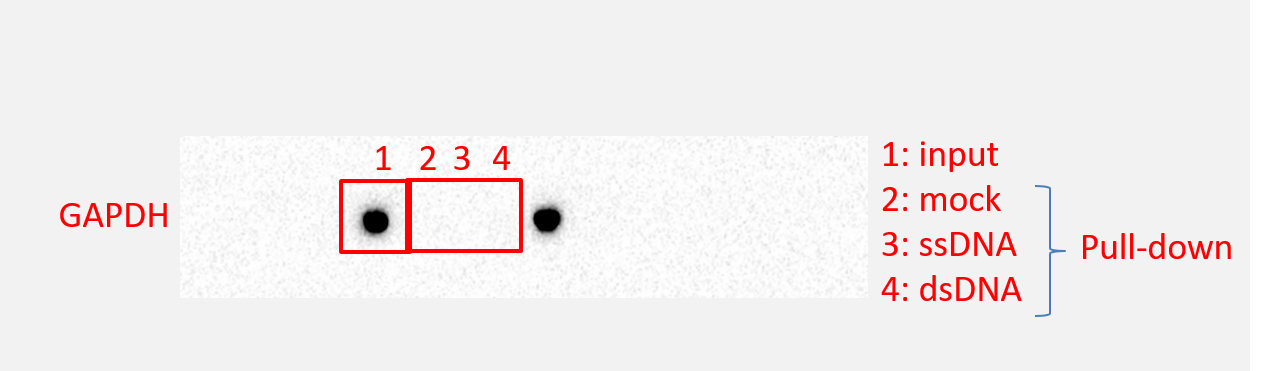

Supplement: Supplementary file 6 — Source Data for Figure 2 [file EMBJ-42-e111961-s001.zip › Figure 2/Figure 2B/Fig 2B_western_GAPDH.tif]

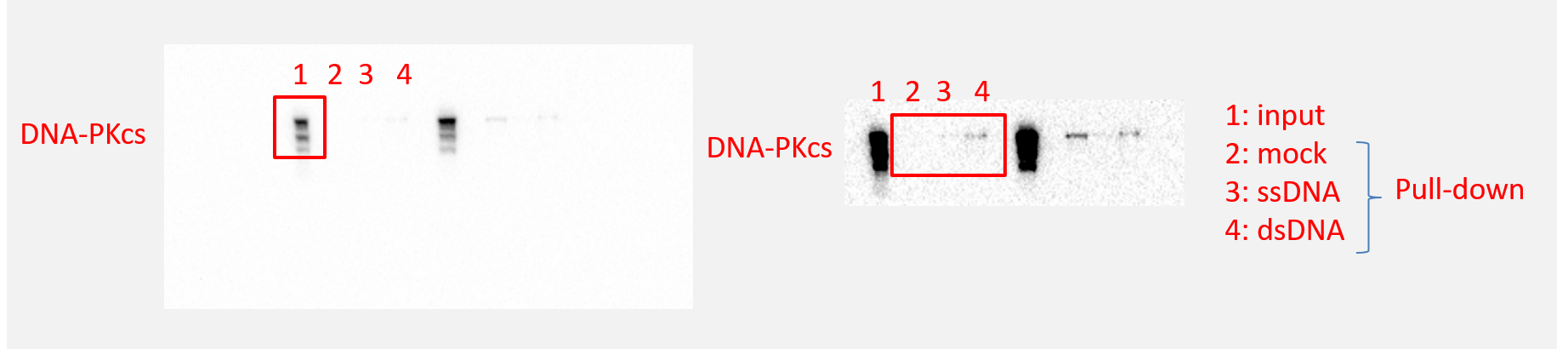

Supplement: Supplementary file 6 — Source Data for Figure 2 [file EMBJ-42-e111961-s001.zip › Figure 2/Figure 2B/Fig 2B_western_DNA-PKcs.tif]

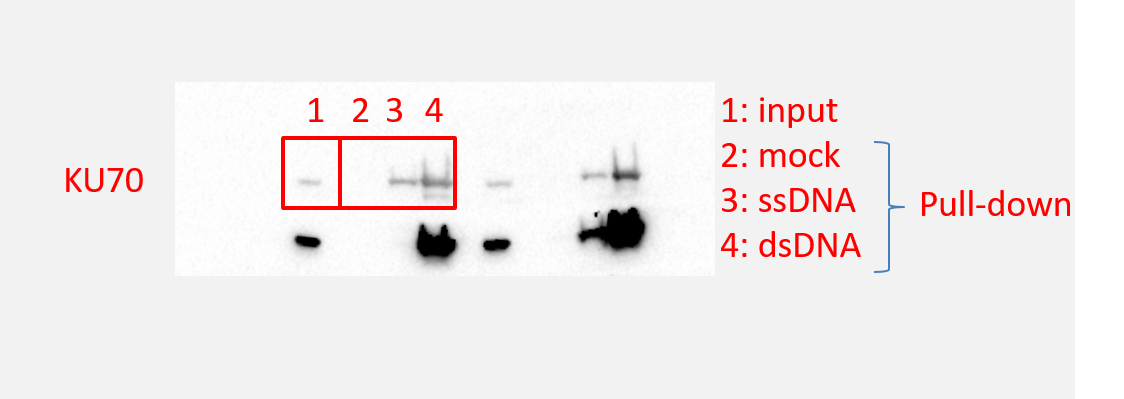

Supplement: Supplementary file 6 — Source Data for Figure 2 [file EMBJ-42-e111961-s001.zip › Figure 2/Figure 2B/Fig 2B_western_KU70.tif]

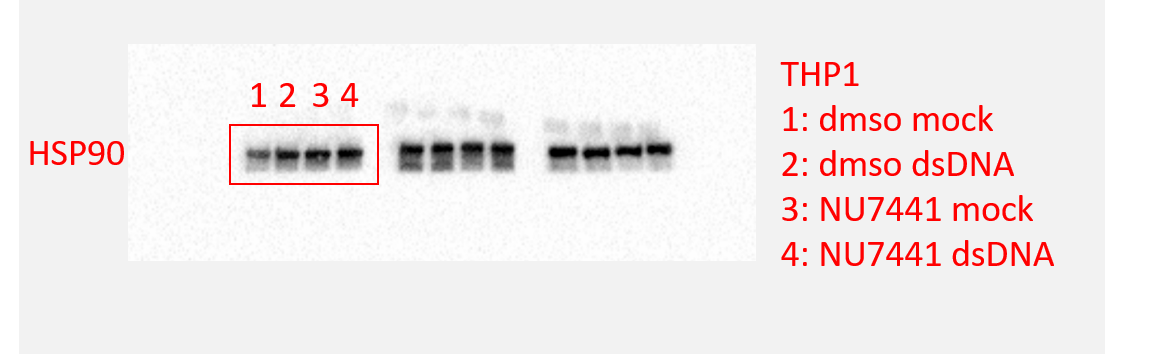

Supplement: Supplementary file 7 — Source Data for Figure 3 [file EMBJ-42-e111961-s011.zip › Figure 3/Figure 3K/Fig 3K_western_HSP90.tif]

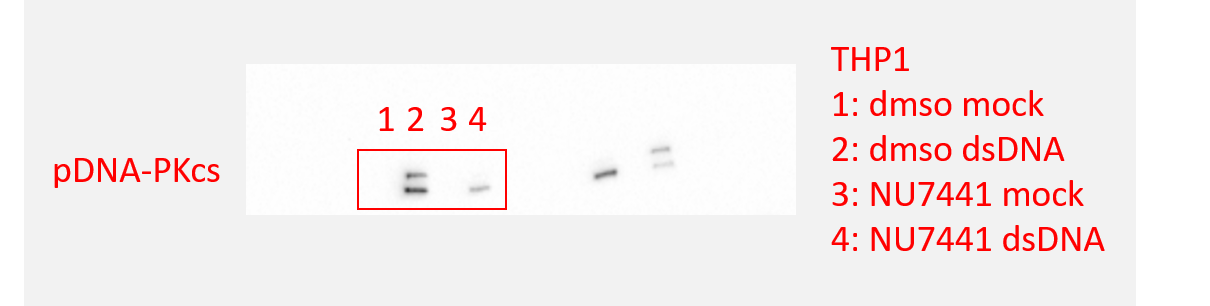

Supplement: Supplementary file 7 — Source Data for Figure 3 [file EMBJ-42-e111961-s011.zip › Figure 3/Figure 3K/Fig 3K_western_pDNA-PKcs.tif]

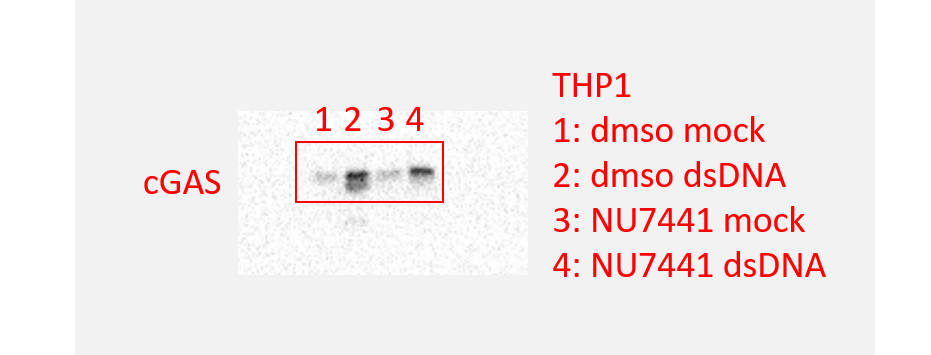

Supplement: Supplementary file 7 — Source Data for Figure 3 [file EMBJ-42-e111961-s011.zip › Figure 3/Figure 3K/Fig 3K_western_cGAS.tif]

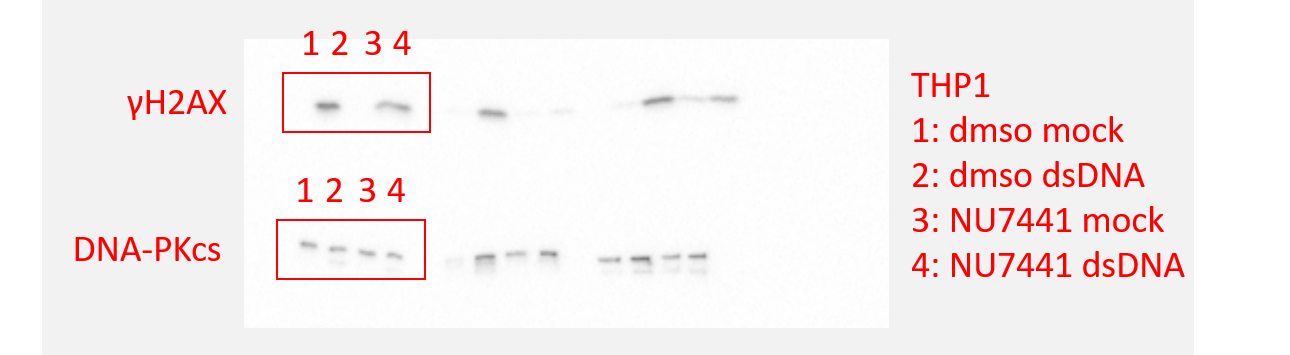

Supplement: Supplementary file 7 — Source Data for Figure 3 [file EMBJ-42-e111961-s011.zip › Figure 3/Figure 3K/Fig 3K_western_DNA-PKcs & gH2AX.tif]

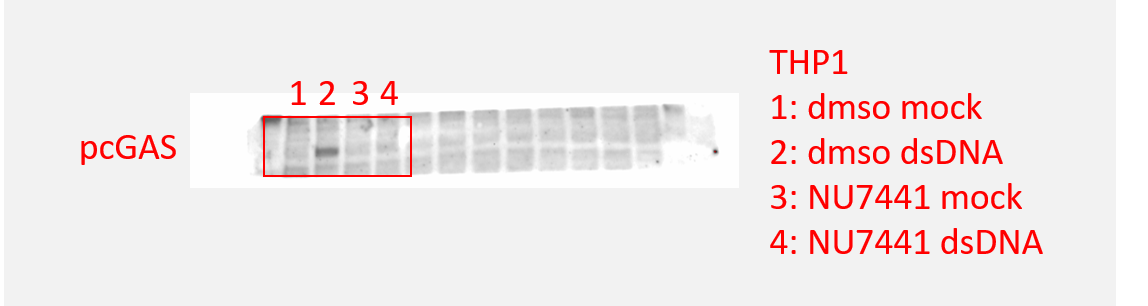

Supplement: Supplementary file 7 — Source Data for Figure 3 [file EMBJ-42-e111961-s011.zip › Figure 3/Figure 3K/Fig 3K_western_pcGAS.tif]

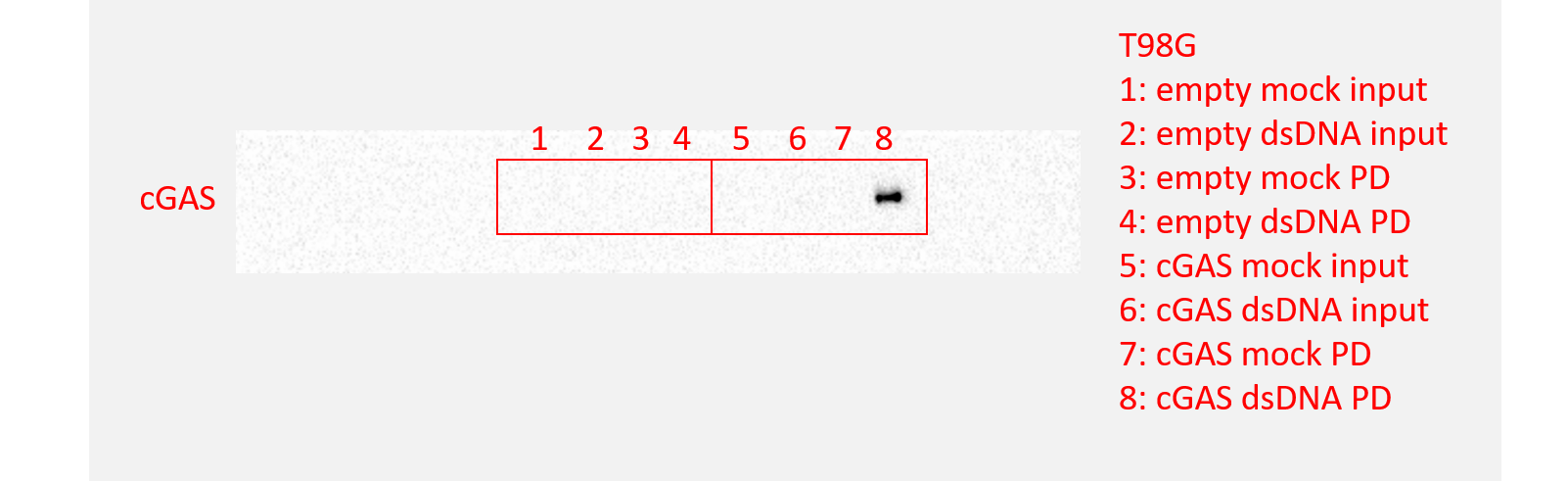

Supplement: Supplementary file 7 — Source Data for Figure 3 [file EMBJ-42-e111961-s011.zip › Figure 3/Figure 3C/Fig 3C_western_cGAS.tif]

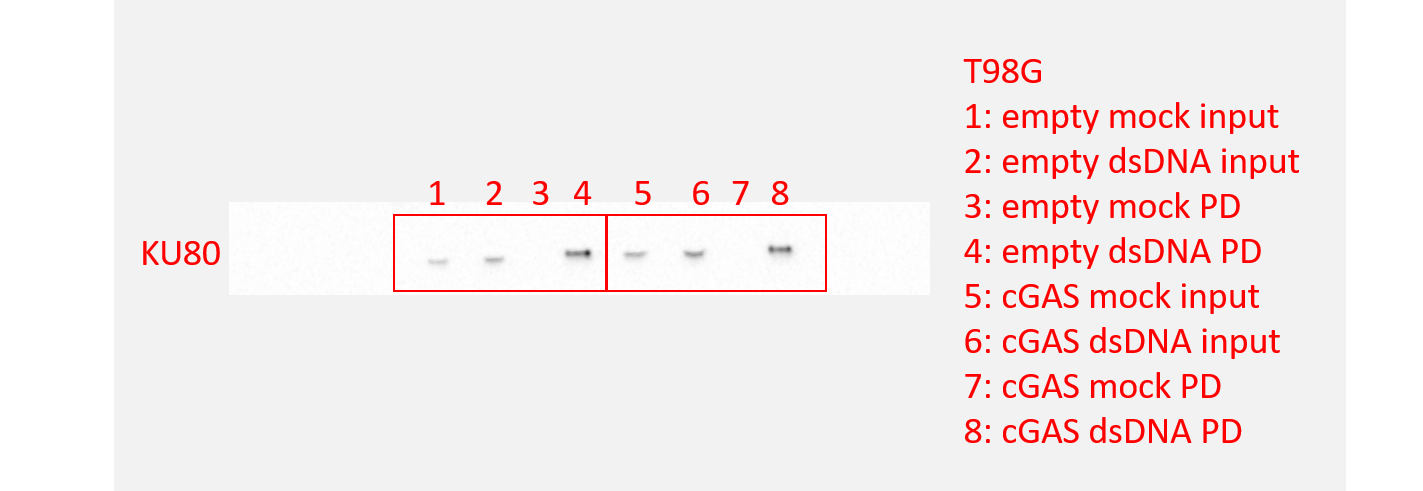

Supplement: Supplementary file 7 — Source Data for Figure 3 [file EMBJ-42-e111961-s011.zip › Figure 3/Figure 3C/Fig 3C_western_KU80.tif]

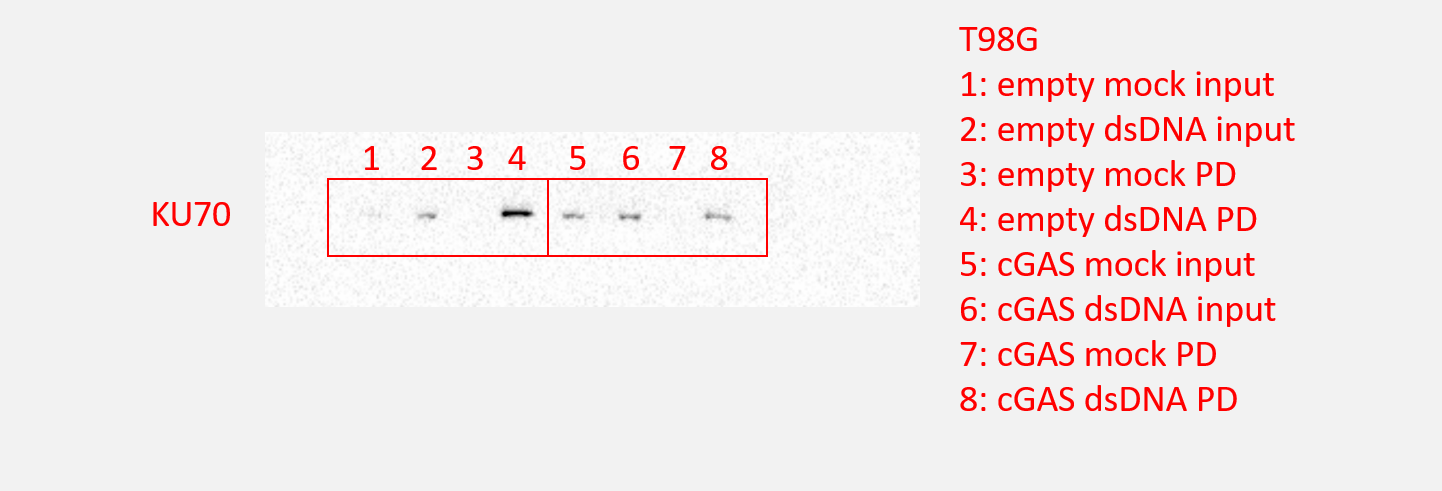

Supplement: Supplementary file 7 — Source Data for Figure 3 [file EMBJ-42-e111961-s011.zip › Figure 3/Figure 3C/Fig 3C_western_KU70.tif]

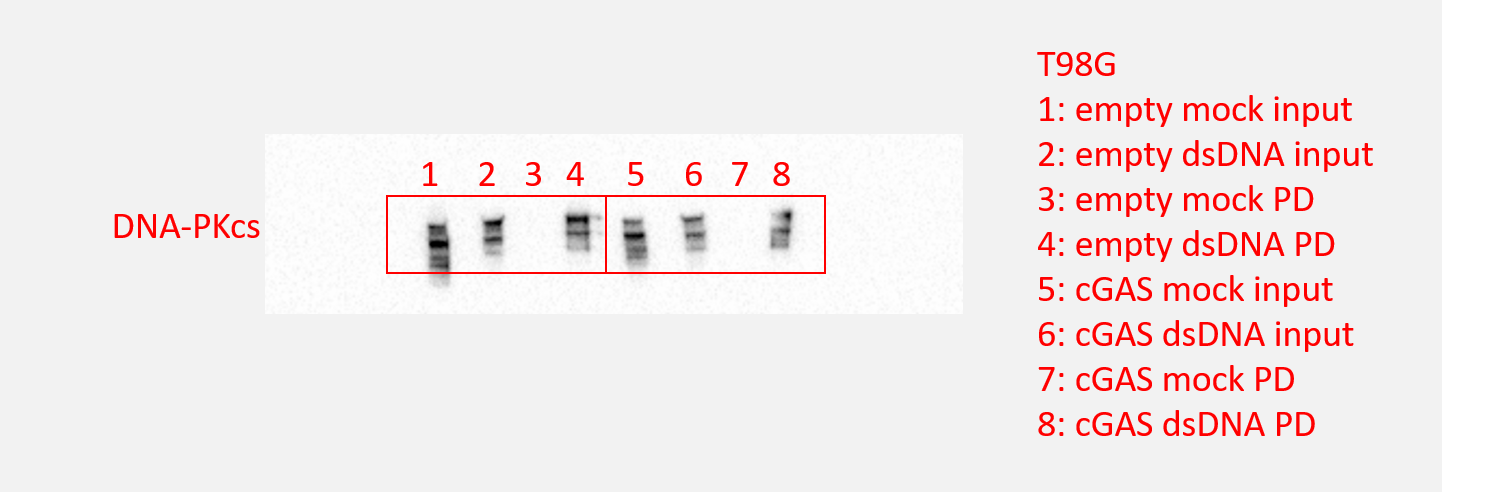

Supplement: Supplementary file 7 — Source Data for Figure 3 [file EMBJ-42-e111961-s011.zip › Figure 3/Figure 3C/Fig 3C_western_DNA-PKcs.tif]

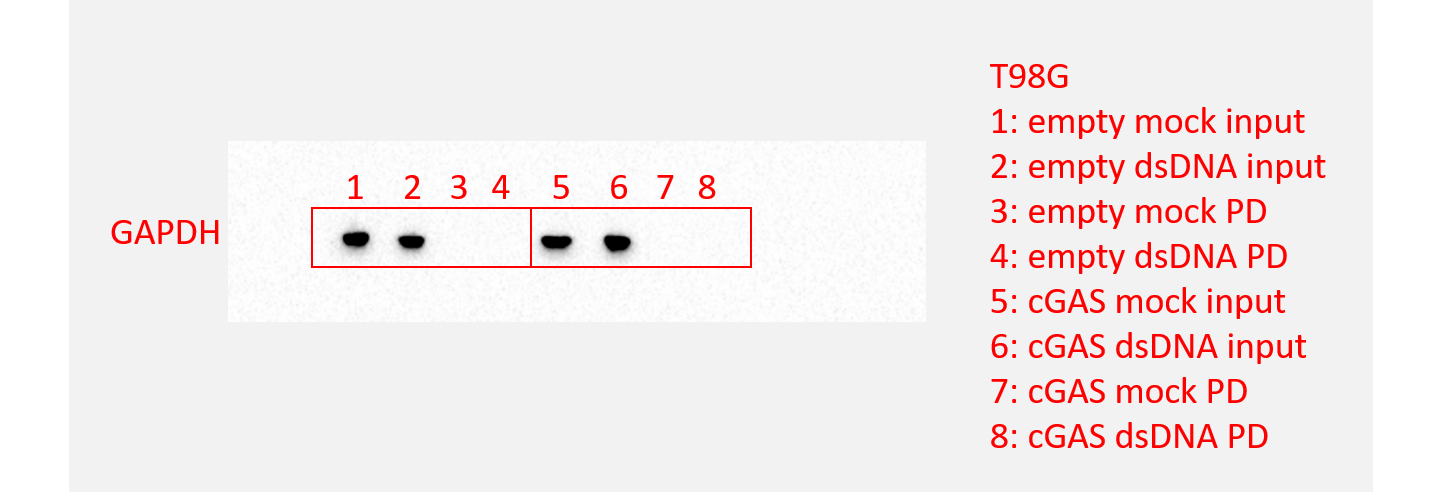

Supplement: Supplementary file 7 — Source Data for Figure 3 [file EMBJ-42-e111961-s011.zip › Figure 3/Figure 3C/Fig 3C_western_GAPDH.tif]

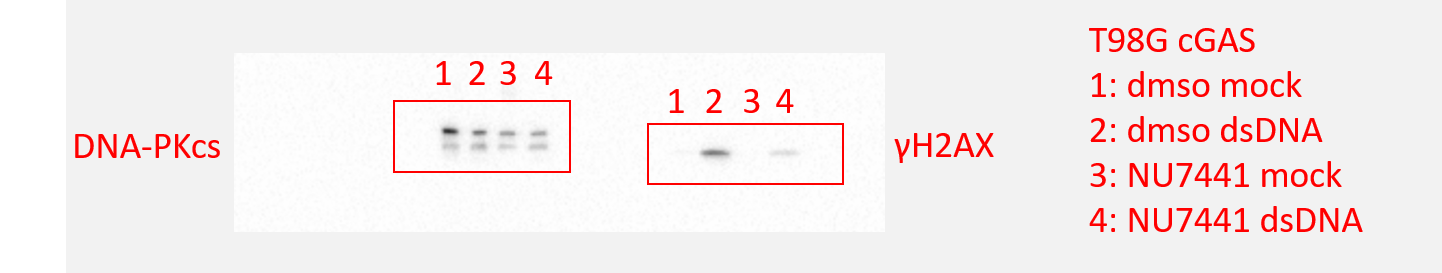

Supplement: Supplementary file 7 — Source Data for Figure 3 [file EMBJ-42-e111961-s011.zip › Figure 3/Figure 3J/Fig 3J_western_DNA-PKcs & gH2AX.tif]

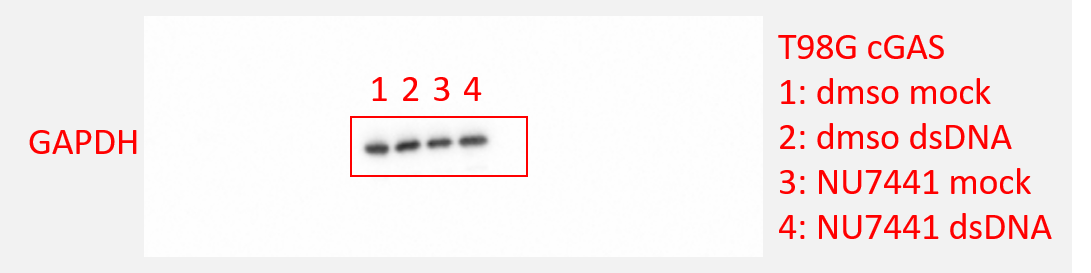

Supplement: Supplementary file 7 — Source Data for Figure 3 [file EMBJ-42-e111961-s011.zip › Figure 3/Figure 3J/Fig 3J_western_GAPDH.tif]

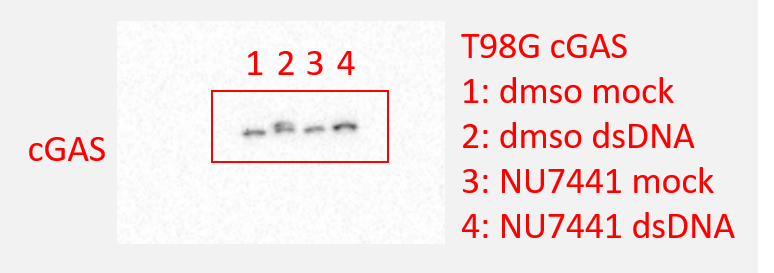

Supplement: Supplementary file 7 — Source Data for Figure 3 [file EMBJ-42-e111961-s011.zip › Figure 3/Figure 3J/Fig 3J_western_cGAS.tif]

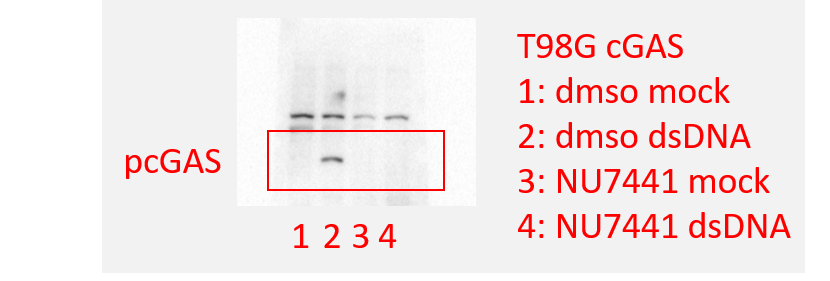

Supplement: Supplementary file 7 — Source Data for Figure 3 [file EMBJ-42-e111961-s011.zip › Figure 3/Figure 3J/Fig 3J_western_pcGAS.tif]

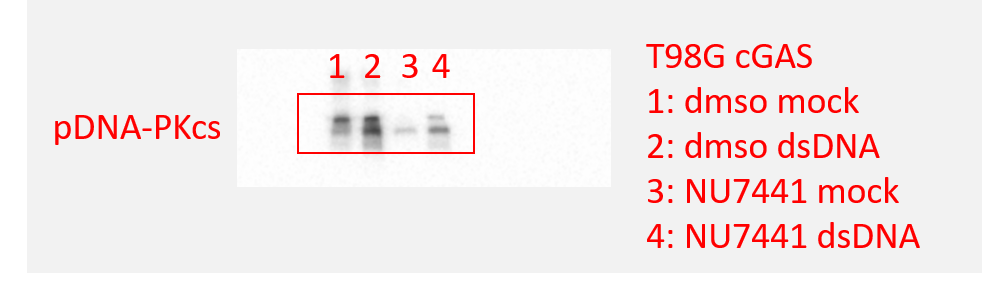

Supplement: Supplementary file 7 — Source Data for Figure 3 [file EMBJ-42-e111961-s011.zip › Figure 3/Figure 3J/Fig 3J_western_pDNA-PKcs.tif]

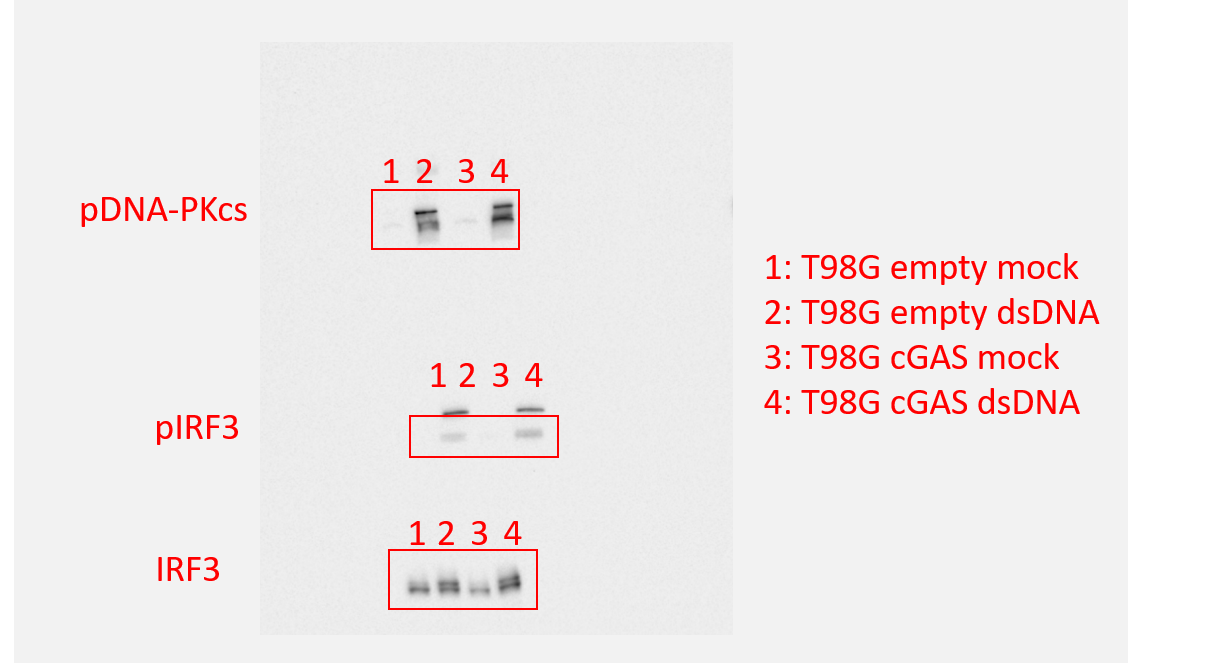

Supplement: Supplementary file 7 — Source Data for Figure 3 [file EMBJ-42-e111961-s011.zip › Figure 3/Figure 3A/Fig3A_western_pDNA-PKcs, pIRF3 & IRF3.tif]

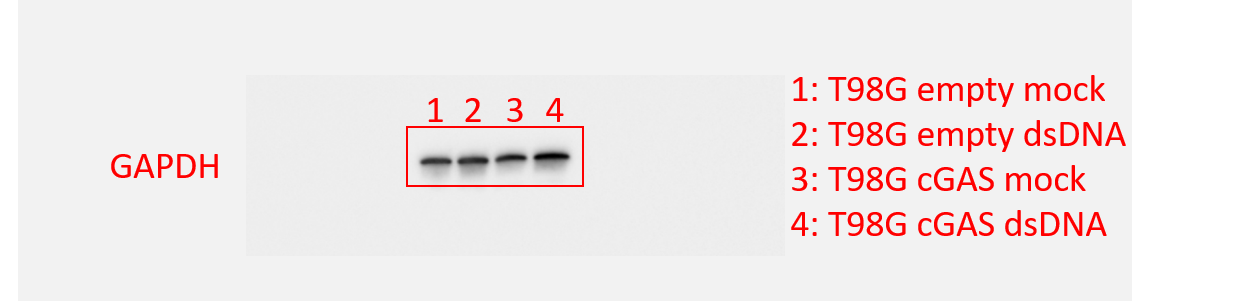

Supplement: Supplementary file 7 — Source Data for Figure 3 [file EMBJ-42-e111961-s011.zip › Figure 3/Figure 3A/Fig3A_western_GAPDH.tif]

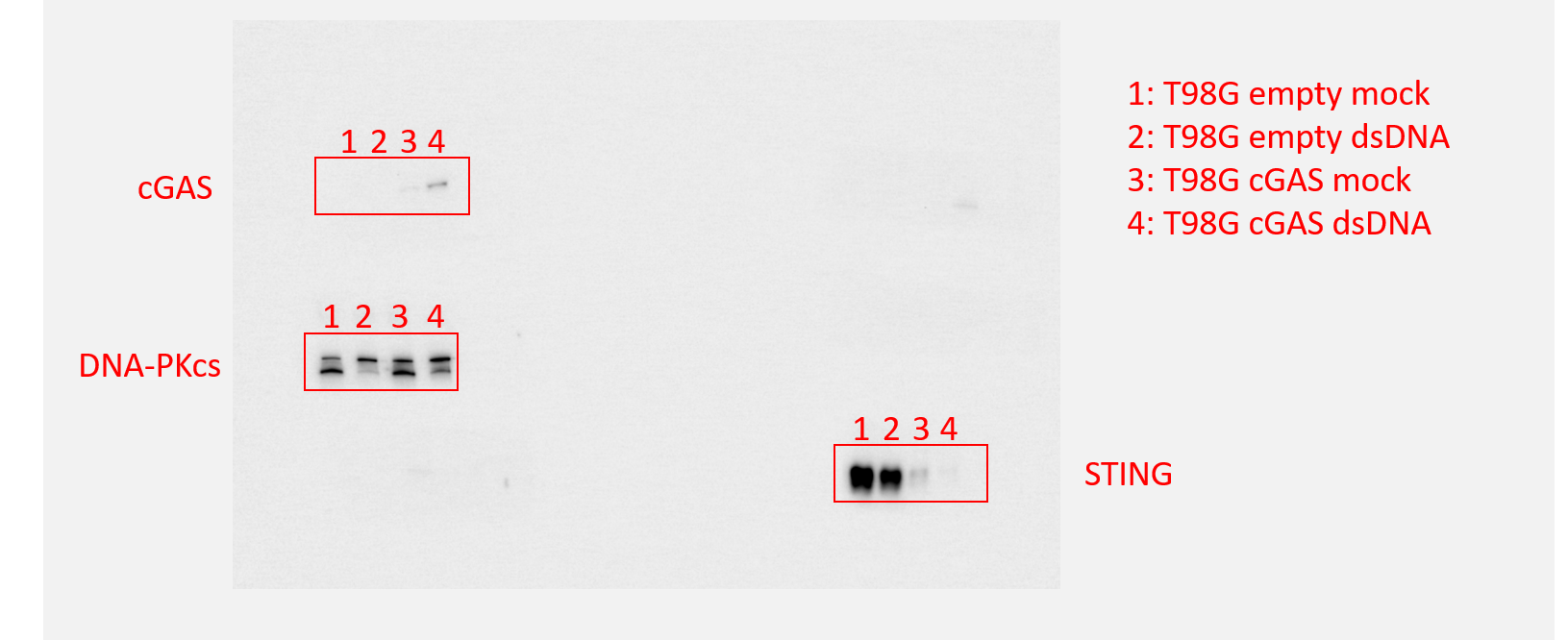

Supplement: Supplementary file 7 — Source Data for Figure 3 [file EMBJ-42-e111961-s011.zip › Figure 3/Figure 3A/Fig3A_western_DNA-PKcs, cGAS & STING.tif]

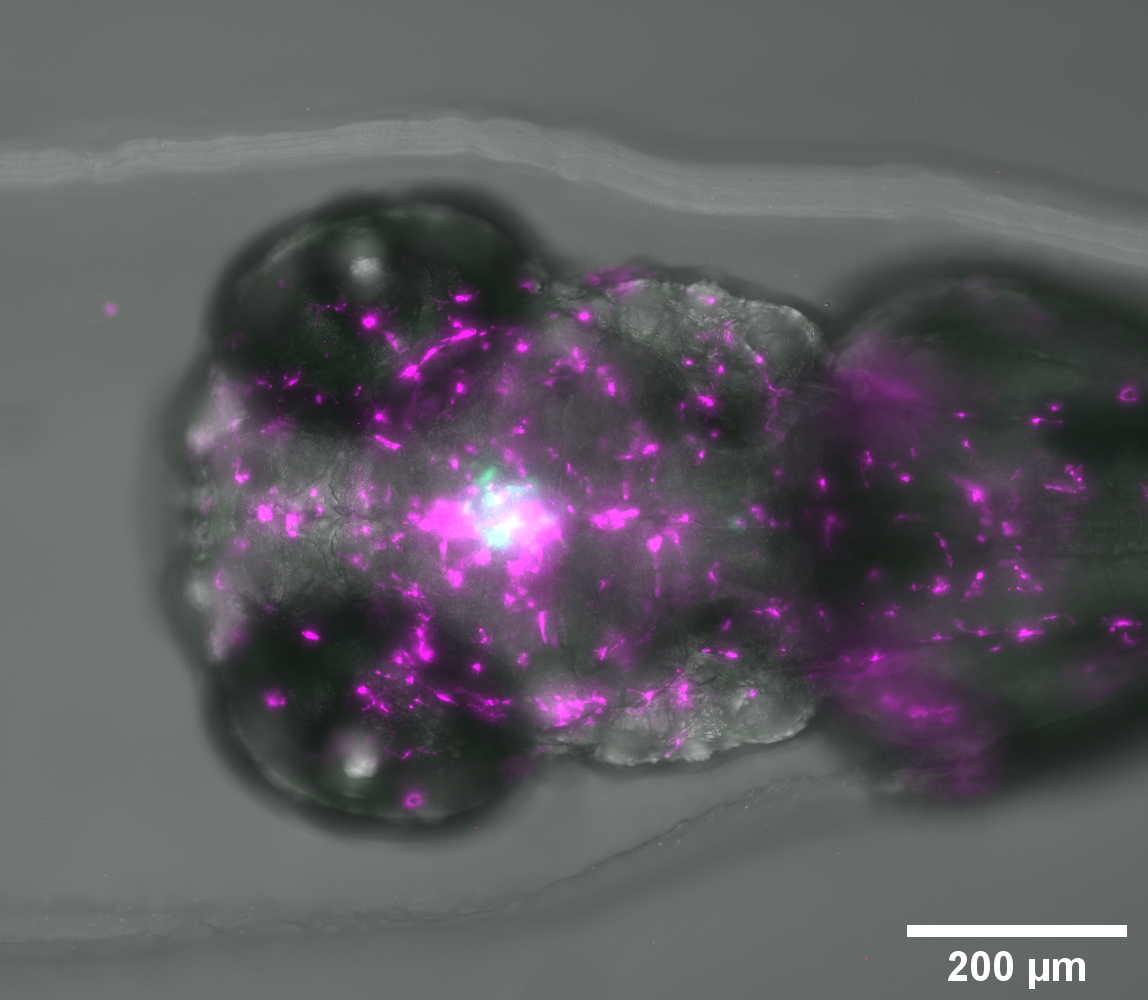

Supplement: Supplementary file 9 — Source Data for Figure 5 [file EMBJ-42-e111961-s006.zip › Figure 5/Figure 5/Fig 5D_T98G-GFP-cGAS_tumor-GFP-macrophage_magenta.tif]

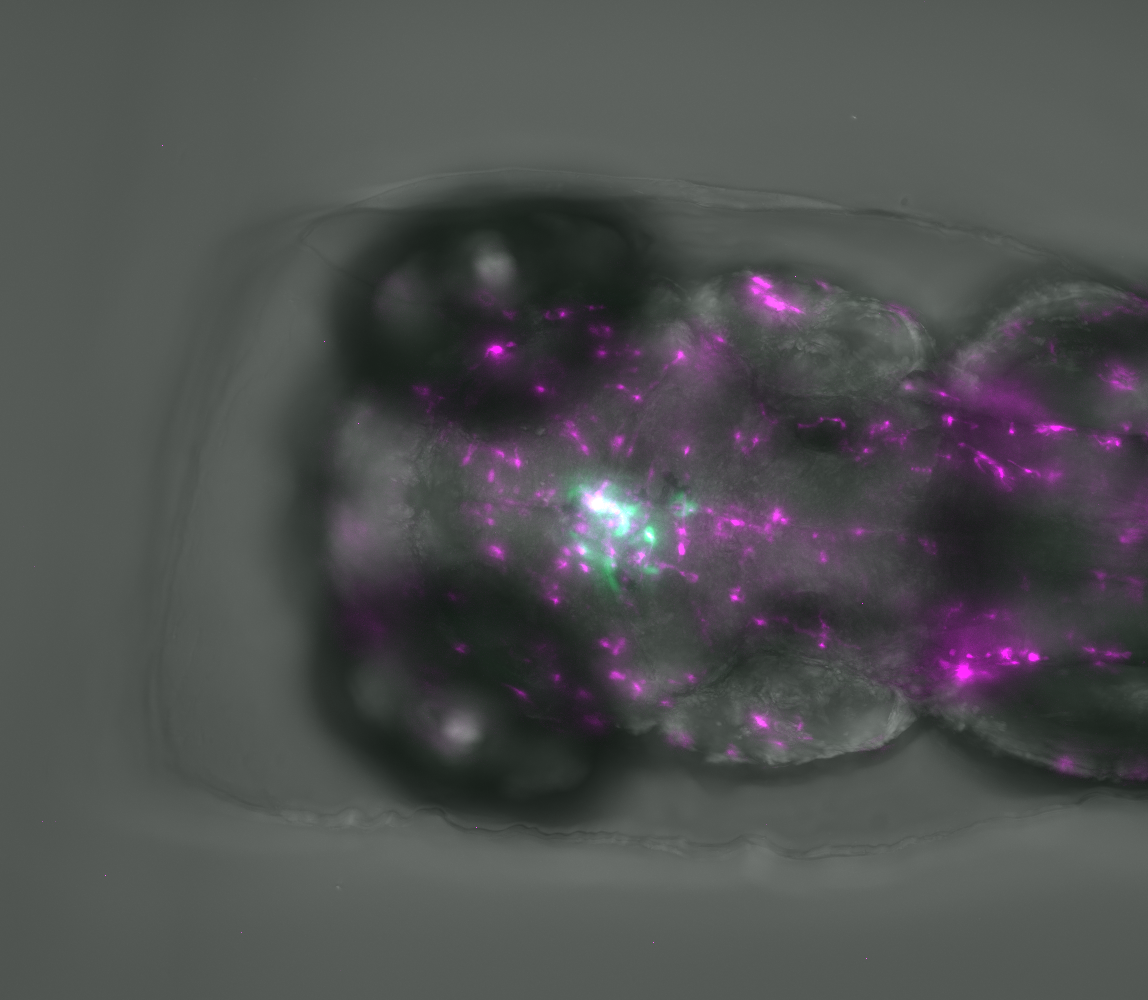

Supplement: Supplementary file 9 — Source Data for Figure 5 [file EMBJ-42-e111961-s006.zip › Figure 5/Figure 5/Fig 5D_T98G-GFP-empty_tumor-GFP_macrophages-magental.tif]
