# Supplementary material for: DNA damage repair kinase DNA‐PK and cGAS synergize to induce cancer‐related inflammation in glioblastoma
Source: EMBO J. 2022 Dec 27;42(7):e111961. doi: 10.15252/embj.2022111961 (PMC10068334; doi:10.15252/embj.2022111961)
Supplement: Supplementary file 11 [file EMBJ-42-e111961-s010.pdf]

THE EMBO JOURNAL

DNA damage repair kinase DNA-PK and cGAS synergize to induce cancer-related inflammation in glioblastoma

Clara Taffoni, Johanna Marines, Hanane Chamma, Soumyabrata Guha, Mathilde Saccas, Amel Bouzid, Ana Chaves Valadão, Clément Maghe, Jane Jardine, Mi Kyung Park, Katarzyna Polak, Mara De Martino, Claire Vanpouille-Box, Maguy Del Rio, Céline Gongora, Julie Gavard, Nicolas Bidere, Min Sup Song, Donovan Pineau, Jean-Philippe Hugnot, Karima Kissa, Laura Fontenille, Fabien Blanchet, Isabelle Vila, and Nadine Laguette
DOI: 10.15252/embj.2022111961

Corresponding author(s): Nadine Laguette (nadine.laguette@igh.cnrs.fr)

Review Timeline:

Submission Date:	23rd Jun 22
Editorial Decision:	22nd Jul 22
Revision Received:	20th Oct 22
Editorial Decision:	29th Nov 22
Revision Received:	30th Nov 22
Accepted:	2nd Dec 22

Editor: Karin Dumstrei

Transaction Report:

(Note: With the exception of the correction of typographical or spelling errors that could be a source of ambiguity, letters and reports are not edited. Depending on transfer agreements, referee reports obtained elsewhere may or may not be included in this compilation. Referee reports are anonymous unless the Referee chooses to sign their reports.)

1st Editorial Decision 22nd Jul 2022

Dear Dr. Laguette,

Thank you for submitting your manuscript for consideration by the EMBO Journal. It has now been seen by three referees whose comments are shown below.

As you can see, the referees find the analysis interesting and insightful. However, they also raise a number of concerns that would have to be resolved for further consideration. Should you be able to address the concerns then I am interested in considering a revised manuscript.

I think it would be helpful to discuss the revisions further and I am happy to do so via video or email. Let me know what works best for you. Please note that I am away on vacation next week, but back in the office on the 1st of August.

When preparing your letter of response to the referees' comments, please bear in mind that this will form part of the Review Process File, and will therefore be available online to the community. For more details on our Transparent Editorial Process, please visit our website: https://www.embopress.org/page/journal/14602075/authorguide#transparentprocess

I thank you for the opportunity to consider your work for publication and look forward to discussing the revisions further with you.

Yours sincerely,

Karin

Karin Dumstrei, PhD
Senior Editor
The EMBO Journal

Instructions for preparing your revised manuscript:

Guide For Authors: https://www.embopress.org/page/journal/14602075/authorguide

I have attached a PDF with helpful tips on how to prepare the revision.

I realize that it is difficult to revise to a specific deadline. In the interest of protecting the conceptual advance provided by the work, we recommend a revision within 3 months (20th Oct 2022).

We generally allow three months as standard revision time. As a matter of policy, competing manuscripts published during this period will not negatively impact on our assessment of the conceptual advance presented by your study.

If you need more time for the revisions please let me know and I can grant an extension.

Use the link below to submit your revision:

https://emboj.msubmit.net/cgi-bin/main.plex

Referee #1:

In this manuscript, the authors examine the role of the DNA damage repair kinase DNA-PK in DNA sensing. This issue has been examined before, and DNA-PK has previously been demonstrated to be involved in STING-dependent DNA sensing (e.g. Ferguson et al., 2012) and has also been shown to promote STING-independent innate immune responses in some human cells (Burleigh et al., 2020). However, its role in cancer cells and immunosurveillance has not yet been studied in depth.
Here, the authors show that glioblastoma cell lines which lack detectable cGAS expression can mount a cGAS- and STING-independent DNA sensing response which results in the activation of IRF3 and the expression of interferons and chemokines such as CXCL10. Furthermore, it is shown that DNA-PK can potentiate cGAS signalling by causing the phosphophorylation of cGAS on Serine 435. Finally, the authors show that the introduction of cGAS in glioblastoma cells suppresses tumour growth in zebrafish and nude mice, but that high cGAS expression in patients is associated with enhanced macrophage recruitment and poor survival.

The findings presented here are interesting - even if partially already present in the literature. The role of additional DNA binding

proteins which synergise with cGAS-STING signalling is still controversial, but needs to be addressed in the field. One of the most striking aspects of this work is some mechanistic insight into the co-operation with cGAS via phosphorylation of cGAS itself - this is a novel finding, and could be explored a little further. The data on tumour growth and patient survival currently show the most striking effects with regard to cGAS expression (with little examination of DNA-PK) - so in my opinion this evidence does not entirely support the proposed title.
Overall, the data is nicely presented, and well controlled.
Main points:
1.) The interferon and CXCL10 mRNA induction in glioblastoma cells (particularly other than T98G) is very low and may not have much physiological relevance - interferon bio-assays and ELISA could be used to show the secretion of (functional) protein.
2.) Some positive controls are missing, e.g. for STING phosphorylation in Fig. 1A, C, E, I, and for the successful deletion of STING in Fig. 2E, G - in both cases treatment with cGAMP or another STING agonist could be used. Specificity of DNA-PK for DNA sensing (rather than general interferon induction) could be shown by using stimulation with dsRNA or poly(I:C) for instance.
3.) The authors mainly measure two read-outs: IFNb and CXCL10 mRNA. However, given the cGAS- and STING-independent nature of DNA-PK signalling, it is not a given that the same cytokine/chemokine profile is induced. A wider expression profile should be monitored for key experiments, e.g. using RNAseq, qPCR arrays or multiplex ELISA.
4.) The response to the chemotherapy agent camptothecin is measured after 48-72h - can an interferon response also be detected at earlier time points?
5.) I am not convinced by the DNA pull-down experiments in Fig. 3C. It looks like there could be less Ku70 pull-down in cGAS-expressing cells, and also the amount of DNA may not be limiting to see more striking effects. Maybe a titration of DNA concentrations, or competition experiments in vitro could answer this important question more convincingly. Also the effect of DNA-PK subunits on cGAS-DNA binding could be examined more directly in vitro, and/or using cGAS phosphorylation mutants and phosphorylation mimics.
6.) Does the presence of DNA-PK subunits affect cGAMP production by cGAS in vitro?
7.) The effects on the interferon response and cGAS phosphorylation in THP1 cells should be confirmed using DNA-PK siRNA - to exclude off-target effects of the inhibitor on another kinase.
8.) To make the results in Fig. 4-6 more relevant to the title of this manuscript, the effect of DNA-PK (rather than just cGAS) should be examined, particularly as the authors propose the use of DNA-PK agonists or antagonists in cancer treatment. The over-expression of cGAS obviously has a very large effect on the tumour models (as has been shown in many other models using e.g. STING agonists), however the manuscript currently does not add another dimension by examining the role of DNA-PK in this context. As DNA-PK inhibition would also be expected to enhance DNA damage in the tumour cells, the data may not be easy to interpret - but as it stands it does not currently contribute much to the remaining manuscript.
9.) Analogously, the effect of DNA-PK high/low expression on patient survival should be shown as in Fig. 6F - is there a difference in a cGAS-low or cGAS-high background?
10.) Both M1 and M2 macrophages increase with higher cGAS and DNA-PK expression (Fig. 6) - would expressing this as M1/M2 ratio reveal any interesting differences? Also, please include PRKDC low, MB21D1 high in the chemokine and macrophage analyses in Fig. 6, to show whether DNA-PK modifies cGAS activity in a high-expressing context.
11.) It is counter-intuitive that in pre-clinical models tumour growth is so strongly inhibited by cGAS, while in patients cGAS expression is detrimental for survival. This is a major question in the field (also concerning the limited/absent success of STING agonist in human trials) which can clearly not be answered in this particular manuscript, but maybe could be mentioned more in the discussion.

Minor points:
1.) For the glioblastoma cells, the term "cGAS-deficient" should be replaced with "without detectable expression of cGAS" or similar.
2.) The presentation of the immunofluorescence data in Fig. 2A is confusing - is this labelled correctly?

Referee #2:

Although downregulation of cGAS, a major cytosolic dsDNA sensor, has been suggested as a mechanism of immune escape by tumor cells, it is unclear if alternate DNA sensor pathways can supplant the cGAS-STING pathway in reinitiating tumor-specific immunity. In this manuscript, Taffoni et al (EMBOJ-2022-111961) presented evidence showing that in GBM cells the DNA-PK DNA repair complex is one such alternate system that not only can supplant cGAS-STING in the absence of cGAS but also may cooperate with cGAS in cGAS-sufficient cells to promote tumor-related immune activities. Thus, this manuscript is both timely and addressing an important question with broad impact in the GBM field and beyond. For the most part, the experiments were well executed and my enthusiasm for the work is relatively high, but significantly reduced by a series of mainly technical issues as articulated below:

Major comments:

1)The choice of models is problematic. T98G cells are a multiploidy cell line that has poor tumorigenicity in vivo. The key in vitro experiments establishing the DNA-PK system as an alternate DNA sensor system should be repeated and presented in low

passage GBM cell lines. The authors introduced the Gli4 and Gli7 GBM stem cells but their use in the data presented was limited.

2)The use of single shRNA/siRNA is a concern. The authors should either use at least 2 independent shRNA/siRNA or perform a rescue experiment to rule out off target effects. Although the use of NU7441, a potent and selective inhibitor of DNA-PK, supported the authors' contention that the observations were specific to DNA-PK, the concentrations of NU7441 used (2-8 M) were well above its IC50 of ~13nM for DNA-PK, reaching levels high enough to inhibit other secondary targets (e.g., mTOR, PI3K) that may confound the reported observations.

3)Although IRF3 is a major downstream target of STING/TBK1 driving type-1 IFN response, NF B (p65) is another major target promoting proinflammatory cytokines that is not addressed by the current data. In STING-deficient cells, can DNA-PK alone also activate NF B in response to dsDNA?

4)The author proposed that the cGAS-DNA-PK cooperation promotes macrophage recruitment based on the use of immunosuppressed preclinical models that don't have an intact adaptive immune system. Although the authors' assertion may be correct, whether it is physiologically relevant in the presence of adaptive immunity is not addressed by the model systems used. For starter, subcutaneous GBM tumors are not physiologic and the author focused on a limited number of cytokines (i.e., CCL2 and CCL5) as indicative of macrophage recruitment. These same chemokines can also recruit adaptive immune cells including cDCs, pDCs and even activated and memory T cells, which are absent in these models. A broader profile of cytokines/chemokines should be included (see above about the potential effects on proinflammatory cytokines that are not included in the analysis). In addition, there are several syngeneic mouse models of GBM that the authors should use instead to dissect the immune effects of the proposed cGAS-DNA-PK cooperation taking into account both the adaptive and innate immune systems.

Minor comments:

1)gRNA labeling is not consistent. Sometimes it's just the gene name (e.g., STING), sometime it's STING-/-.

2)A composite drawing summarizing the data will be helpful.

Referee #3:

Comments
In this study, the authors nicely demonstrated that DNA-PK DNA repair complex drives cGAS-independent inflammatory responses in glioblastoma cells. Moreover, the catalytic activity of DNA-PK is critical for cGAS-dependent cGAMP production and optimal downstream signaling. Furtherly, the re-expression of cGAS in glioblastoma cancer cells resulted in enhanced expression of chemokines that promote macrophage recruitment in the tumor microenvironment. The authors also provided data the expression of both cGAS and DNA-PKcs increased with tumor grade. Overall, the data are convincing and the findings are helpful to understand the role of DNA-PK in genotoxic stress-induced inflammatory response. However, there are major concerns need to be addressed.

1. One important finding of this study is that DNA-PK promotes DNA and chemotherapy-associated inflammatory responses independent of cGAS in the context of glioblastoma cancer cells. However, DNA-PK has been previously demonstrated to be an additional DNA senor driving STING-independent DNA sensing pathway (SIDSP) specifically in human cells (PMID: 31980485). This markedly undermined the novelty of current study. Also, is the finding reported by the authors in this study specific to human-derived glioblastoma cells? How about mouse glioblastoma cells?
2. As the authors mentioned in the introduction part, previous work reported that DNA-PK inhibited cGAS activation by direct modulating its phosphorylation on T68/S213 (PMID: 33273464) or by inducing cytosolic translocation of PARP1 (PMID: 35460603). In this study, the authors proposed that DNA-PK mediated the phosphorylation of cGAS on S435 to increase its catalytic activity. How can the authors reconcile their findings with previous report.
3. Figure 1, the authors employed camptothecin as a genotoxic agent to induce the accumulation of cytosolic DNA and examine its sensing mechanism. How about other genotoxic agents such as cisplatin, etoposide.
4. Figure 2A, dsDNA stimulation caused cytosolic translocation of DNA-PKcs and phosphorylated DNA-PKcs. This is interesting and the authors should at least discuss this. An ensuing question is will this also happen when cGAS is expressed?
Figure 2C, the knockdown of KU70 seemed to cause the reduced expression of KU80 and DNA-PKcs.
Figure 2I, KU70 and KU80 should be placed into the diagram.
5. Figure 3, the authors conclude that DNA-PK and cGAS synergized to induce type I interferon response to DNA stimulation. However, in most cases, it hard to evaluate whether those effects are synergistic or additive.
Figure 3D and 3E, the experiments were performed with DNA-PK inhibitor NU7441, cGAS inhibitors should also be introduced.
Figure 3H, it's striking to find that NU7441 completely abolished the enzymatic activity of cGAS, indicating that at least in THP-1 cells DNA-PK is an upstream regulator of cGAS and cGAS is dominant receptor for dsDNA, which is against with the hypothesis

on the synergy of DNA-PK and cGAS in sensing DNA.
6. Figure 4 and 5, the authors demonstrated that cGAS re-expression in glioblastoma cancer cells impaired tumorigenesis and promoted macrophage recruitment, however, whether these processes are dependent on the cooperation of DNA-PK and cGAS are not known. The authors should provide additional evidence (eg. by introducing DNA-PK inhibitor) to strengthen their conclusions.
7. Figure 6F, the PRKDClowMB21D1low should also be included.

Minors
1. The phrase "immunogenicity" used in title is not accurate.
2. In the 2nd paragraph of introduction part, the protumor effects cGAS-STING were not fully summarized.
3. The reference Wang, Zhao et al., 2022 is referred in the text of introduction part but is not listed in the reference.

Point by Point answer to reviewer's comments:

Referee #1:

In this manuscript, the authors examine the role of the DNA damage repair kinase DNA-PK in DNA sensing. This issue has been examined before, and DNA-PK has previously been demonstrated to be involved in STING-dependent DNA sensing (e.g. Ferguson et al., 2012) and has also been shown to promote STING-independent innate immune responses in some human cells (Burleigh et al., 2020). However, its role in cancer cells and immunosurveillance has not yet been studied in depth.

Here, the authors show that glioblastoma cell lines which lack detectable cGAS expression can mount a cGAS- and STING-independent DNA sensing response which results in the activation of IRF3 and the expression of interferons and chemokines such as CXCL10. Furthermore, it is shown that DNA-PK can potentiate cGAS signalling by causing the phosphophorylation of cGAS on Serine 435. Finally, the authors show that the introduction of cGAS in glioblastoma cells suppresses tumour growth in zebrafish and nude mice, but that high cGAS expression in patients is associated with enhanced macrophage recruitment and poor survival.

The findings presented here are interesting - even if partially already present in the literature. The role of additional DNA binding proteins which synergise with cGAS-STING signalling is still controversial, but needs to be addressed in the field. One of the most striking aspects of this work is some mechanistic insight into the co-operation with cGAS via phosphorylation of cGAS itself - this is a novel finding, and could be explored a little further. The data on tumour growth and patient survival currently show the most striking effects with regard to cGAS expression (with little examination of DNA-PK) - so in my opinion this evidence does not entirely support the proposed title.

Overall, the data is nicely presented, and well controlled.

Main points:

1.) The interferon and CXCL10 mRNA induction in glioblastoma cells (particularly other than T98G) is very low and may not have much physiological relevance - interferon bio-assays and ELISA could be used to show the secretion of (functional) protein.

To demonstrate the production of bioactive Interferons following dsDNA stimulation of glioblastoma cells, we have performed conditioned media assays, where THP-1 cells are treated with supernatants from T98G cells +/- dsDNA. Such assays showed the induction of IFN response genes in THP-1 cells, demonstrating the production of bioactive IFNs (Fig 1E).

Note that Gli4 and Gli7 are very slow growth cells and difficult to transfect, which may account for the low-grade IFNβ induction measured upon stimulation with dsDNA. Yet, *CXCL10* expression attests to the production of bioactive IFNs. In addition, low-grade (subclinical) IFN levels are well-established to be sufficient to induce biological effects and are associated with several human pathologies (PMID: 28420733). Thus, low levels of IFNs are generally accepted to be of physiological relevance.

2.) Some positive controls are missing, e.g. for STING phosphorylation in Fig. 1A, C, E, I, and for the successful deletion of STING in Fig. 2E, G - in both cases treatment with cGAMP or another STING agonist could be used.

We have added the following positive controls requested by the reviewers:

- Positive controls for STING phosphorylation using cGAMP transfection (see new Fig 1A).
- Confirmation of the successful deletion of STING by performing cGAMP transfection in control and STING KO cells, coupled to WB and RT-qPCR analysis (see new Fig EV2E, F).

Specificity of DNA-PK for DNA sensing (rather than general interferon induction) could be shown by using stimulation with dsRNA or poly(I:C) for instance.

The specificity of DNA-PK for DNA sensing has already been tested and published (PMID: 31980485). We thus deemed unnecessary to duplicate this data.

3.) The authors mainly measure two read-outs: IFNb and CXCL10 mRNA. However, given the cGAS- and STING-independent nature of DNA-PK signalling, it is not a given that the same cytokine/chemokine profile is induced. A wider expression profile should be monitored for key experiments, e.g. using RNAseq, qPCR arrays or multiplex ELISA.

In agreement with the reviewer's suggestion, we have now provided a broader view of cytokine/chemokine induction following DNA-PK detection in absence of cGAS, we have included RT-qPCR data of T98G +/- NU7441 and T98GcGAS +/- NU7441; in the presence or absence of dsDNA stimulation. This allowed a visualization of the spectrum of cytokines and chemokines produced in a DNA-PK dependent manner in the presence and absence of cGAS, beyond IFNB and CXCL10. In particular we have shown a synergy similar to that observed for *IFNB* and *CXCL10* in cGAS overexpressing cells for type III IFN (*IFN λ*), *IL6*, *CCL2* and *CCL3* gene expression. Interestingly, *CCL5* expression, although boosted by the re-expression of cGAS was not inhibited by NU7441-mediated inhibition of DNA-PK (see new Fig EV5D-E). These data thus underscore that although cGAS and DNA-PK cooperate for the induction of the expression of subsets of cytokines/chemokines, there is likely additional regulatory parameters controlling gene activation downstream of cGAS and DNA-PK.

4.) The response to the chemotherapy agent camptothecin is measured after 48-72h - can an interferon response also be detected at earlier time points?

The induction measured at 24h is very low. Below is a graph presenting the typical inductions witnessed at 24 h (n=2). A weak, non-significant induction of *IFNB* can be measured at 24h, which is not sufficient to induce *CXCL10* expression. Please refer to **Figure A** provided below**.**

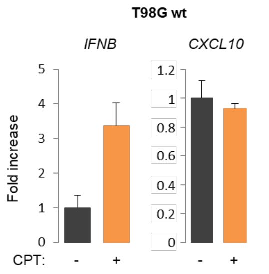

Figure A: T98G cells were treated or not for 24 h with 0.16 µM CPT and *IFNB* and *CXCL10* mRNA levels were analyzed by RT-qPCR (n=2).

5.) I am not convinced by the DNA pull-down experiments in Fig. 3C. It looks like there could be less Ku70 pull-down in cGAS-expressing cells, and also the amount of DNA may not be limiting to see more striking effects. Maybe a titration of DNA concentrations, or competition experiments in vitro could answer this important question more convincingly.

Also the effect of DNA-PK subunits on cGAS-DNA binding could be examined more directly in vitro, and/or using cGAS phosphorylation mutants and phosphorylation mimics.

To address the reviewer's concern, we have now performed titration experiments using limiting amounts of dsDNA. In these assays, we observed that increasing the amount of dsDNA provided to the cells increased dramatically the amount of cGAS retrieved in pull-down experiments and, while the amount of KU80 pulled-down was decreased, the binding of KU70 and DNA-PKcs was not impaired (Fig EV3C. This suggests that cGAS association with dsDNA does not prevent the recruitment of DNA-PK. Furthermore, a recent manuscript (PMID: 36070696) described the cooperation between KU70 and cGAS, showing that in absence of KU70 or KU80, cGAS binds less to dsDNA, leading to an impairment of cGAS activation. Taken together these data rule-out a competition between DNA-PK and cGAS.

6.) Does the presence of DNA-PK subunits affect cGAMP production by cGAS in vitro?

Owing to the large size of DNA-PKcs and the heterotrimeric nature of the DNA-PK complex, such in vitro experiments were not readily feasible. However, a recent manuscript (PMID: 36070696) assessed

the impact of KU70 and KU80 on cGAS showing that both KU70 and KU80 promotes cGAS activation. This is in support of our work showing that DNA-PK boosts cGAS activation.

7.) The effects on the interferon response and cGAS phosphorylation in THP1 cells should be confirmed using DNA-PK siRNA - to exclude off-target effects of the inhibitor on another kinase.
Unfortunately, siRNA transfection of THP-1 did not lead to efficient DNA-PK knockdown, precluding the analysis in this model. However, we have now ruled-out the off-target effects of NU7441 in our experimental set-ups by using 3 independent DNA-PKcs-targeting siRNAs, 2 KU70-targeting siRNAs and 1 KU80 siRNA in T98G cells (see new Fig1H, Fig EV1I, new Fig2D and Fig EV2D). In addition, the concentration ranges of NU7441 used in our study are in agreement with previous reports (PMID: 31980485), where no off-target effect was reported. The dose used in our manuscript (2µM) is below the threshold for inhibition of PI3K. Although inhibition of mTOR could be possible, we have observed that there is not mTOR activation upon dsDNA-mediated stimulation of our cells (see Figure B). Thus, altogether our data support that DNA-PK inhibition is responsible for decreased type I IFN observed upon NU7441 treatment.

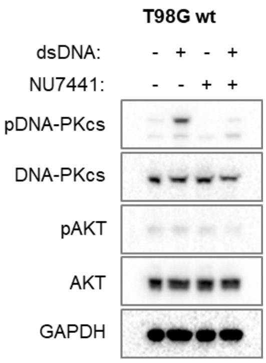

Figure B: T98G cells were challenged or not with dsDNA for 6 h, in the presence or not of the NU7441 DNA-PKcs inhibitor, prior WB analysis using indicated antibodies.

8.) To make the results in Fig. 4-6 more relevant to the title of this manuscript, the effect of DNA-PK (rather than just cGAS) should be examined, particularly as the authors propose the use of DNA-PK agonists or antagonists in cancer treatment. The over-expression of cGAS obviously has a very large effect on the tumour models (as has been shown in many other models using e.g. STING agonists), however the manuscript currently does not add another dimension by examining the role of DNA-PK in this context. As DNA-PK inhibition would also be expected to enhance DNA damage in the tumour cells, the data may not be easy to interpret - but as it stands it does not currently contribute much to the remaining manuscript.
We agree that the role of DNA-PK inhibition, in conjunction with cGAS inhibition is not addressed in the current manuscript. Indeed, DNA-PK inhibition leads to accumulation of DNA-damage in cancer cells, thus rendering read-outs difficult to interpret. As per example, we have observed that inhibition of DNA-PK leads to increased mortality in cGAS expressing T98G cells in prolonged treatments [**Figure C -** Figure shown to referees but removed from RPF]. Such mortality may also result from other functions of DNA-PK and cGAS. For example, cGAS has been reported to inhibit Homologous Recombination (PMID: 30356214; PMID: 31544964). Thus combination of cGAS expression and DNA-PK inhibition would result in enhanced (unrepaired) DNA-damage levels incompatible with cell survival. Assessment of the role of inflammation in this context is therefore not possible. We have therefore changed the title of our manuscript to accurately describe our conclusion and removed the notion of crosstalk.

µM of NU7441, at day 8 post treatment, using the Cell titer Glo kit (Promega). N=3 (+SEM), * $p<0.05$, ** $p<0.01$. 2) Representative image of T98G and cGAS-expressing T98G spheroids taken using the Celigo software at day 8 post NU7441 treatment. 3) T98G and cGAS-expressing T98G spheroids survival (%) is analysed in response to 0.5 µM NU7441 and/or 10 nM camptotecine, at day 8 post treatment, as in panel 1. N=3 (+SEM), * $p<0.05$, ** $p<0.01$, *** $p<0.001$. 4) Representative image of T98G and cGAS-expressing T98G spheroids treated as in panel 3.

9.) Analogously, the effect of DNA-PK high/low expression on patient survival should be shown as in Fig. 6F - is there a difference in a cGAS-low or cGAS-high background?
In agreement with the reviewer's suggestion, we have now included the data from DNA-PK low/cGAS low patients. However, there were too few DNA-PKlow cGAS-high patients in the database (13 cases) to be included in the plots and to perform statistical analysis. These data are now provided in Source data Figure 6.

10.) Both M1 and M2 macrophages increase with higher cGAS and DNA-PK expression (Fig. 6) - would expressing this as M1/M2 ratio reveal any interesting differences?
In agreement with the reviewer's suggestion, we have now included an analysis of M1/M2 ratios, which did not show any significant change (Fig 6F).

Also, please include PRKDC low, MB21D1 high in the chemokine and macrophage analyses in Fig. 6, to show whether DNA-PK modifies cGAS activity in a high-expressing context.
Unfortunately, this population cannot be presented for the above stated reasons and raw data are now provided in Source Data Figure 6.

11.) It is counter-intuitive that in pre-clinical models tumour growth is so strongly inhibited by cGAS, while in patients cGAS expression is detrimental for survival. This is a major question in the field (also concerning the limited/absent success of STING agonist in human trials) which can clearly not be answered in this particular manuscript, but maybe could be mentioned more in the discussion.

To complement the presented meta-analyses, we have now included immunohistochemistry analysis of glioblastoma samples (Fig 6I, Table I and II). This showed a positive correlation between DNA-PKcs and cGAS protein levels, reinforcing that the levels of these proteins are increased during tumorigenesis. We have now added discussion on the impact of cGAS expression on tumorigenesis (see pages 3 and 10). Notably we discuss previous work indicating that primary tumors repress the cGAS-STING axis (PMID: 29342134) and recent data indicating that a functional cGAS-STING axis promotes cancer cell survival (PMID: 34524844). Those reports support our data showing that in early tumorigenesis, the expression of cGAS is repressed presumably because cGAS is deleterious to tumor initiation, but is re-expressed at later stages, to support chronic inflammation.

Minor points:

1.) For the glioblastoma cells, the term "cGAS-deficient" should be replaced with "without detectable expression of cGAS" or similar.

In agreement with the reviewer's comment, this has been rephrased throughout the text.

2.) The presentation of the immunofluorescence data in Fig. 2A is confusing - is this labelled correctly?

Yes, it is correct. For clarity, we used different colors for the two antibodies: DNA-PKcs in green and pDNA-PKcs in yellow.

Referee #2:

Although downregulation of cGAS, a major cytosolic dsDNA sensor, has been suggested as a mechanism of immune escape by tumor cells, it is unclear if alternate DNA sensor pathways can supplant the cGAS-STING pathway in reinitiating tumor-specific immunity. In this manuscript, Taffoni et al (EMBOJ-2022-111961) presented evidence showing that in GBM cells the DNA-PK DNA repair complex is one such alternate system that not only can supplant cGAS-STING in the absence of cGAS but also may cooperate with cGAS in cGAS-sufficient cells to promote tumor-related immune activities. Thus, this manuscript is both timely and addressing an important question with broad impact in the GBM field and beyond. For the most part, the experiments were well executed and my enthusiasm for the work is relatively high, but significantly reduced by a series of mainly technical issues as articulated below:

Major comments:

1)The choice of models is problematic. T98G cells are a multiploidy cell line that has poor tumorigenicity in vivo. The key in vitro experiments establishing the DNA-PK system as an alternate DNA sensor system should be repeated and presented in low passage GBM cell lines. The authors introduced the Gli4 and Gli7 GBM stem cells but their use in the data presented was limited.

To address the reviewer's concern, we have now analyzed the expression of cGAS and STING in additional patient-derived glioblastoma stem-like cells (Fig EV1C) and STING pathway activation upon dsDNA transfection (EV Fig1D,) and the implication of DNA-PKcs in eliciting type I IFN responses (EV Fig1F-G). We also provide immunofluorescence analysis performed on the Gli7 GBM cell line that shows pDNAPK is in the cytosol upon dsDNA transfection (Fig EV2A). Altogether, these experiments confirmed that DNA-PK can supplant cGAS in GBM cells and serve as an alternative cytosolic dsDNA detection pathway.

2)The use of single shRNA/siRNA is a concern. The authors should either use at least 2 independent shRNA/siRNA or perform a rescue experiment to rule out off target effects.

To address the reviewer's concerns, we have now included data obtained using:

- 3 different siRNAs targeting DNA-PKcs (Fig 1H and EV1I).
- 2 different siRNAs targeting KU70 and 1 siRNA targeting KU80 (Fig 2D and EV2D)

Adding these additional siRNA allowed us to confirm that the DNA-PK complex is responsible for cGAS-independent type I IFN responses to dsDNA in glioblastoma cells.

Although the use of NU7441, a potent and selective inhibitor of DNA-PK, supported the authors' contention that the observations were specific to DNA-PK, the concentrations of NU7441 used (2-8 μM) were well above its IC50 of ~13nM for DNA-PK, reaching levels high enough to inhibit other secondary targets (e.g., mTOR, PI3K) that may confound the reported observations.
We used 2 μM of Nu7441 in experiments performed in cells, a concentration that was previously established in similar experimental conditions to inhibit DNA-PK activity selectively (PMID: 31980485). While 2 μM remains below the threshold for inhibition of PI3K, we have evidence that mTOR is not activated in T98G upon dsDNA transfection. Indeed, dsDNA transfection did not lead to phosphorylation of the AKT downstream target of mTOR (see Figure B). Therefore, we believe that in our experimental set-up, there would not be a confounding effect associated with potential mTOR inhibition. Higher amounts of NU7441 were used in vitro to show that even in large excess, this compound does not inhibit cGAS (Fig EV3K).

3)Although IRF3 is a major downstream target of STING/TBK1 driving type-1 IFN response, NFκB (p65) is another major target promoting proinflammatory cytokines that is not addressed by the current data. In STING-deficient cells, can DNA-PK alone also activate NFκB in response to dsDNA?
We have now monitored NF-KB activation and observed that dsDNA stimulation of T98G cells does not lead to NF-kB activation (Fig 1A). This suggests that NF-kB is not a downstream target of DNA-PK in T98G cells.

4)The author proposed that the cGAS-DNA-PK cooperation promotes macrophage recruitment based on the use of immunosuppressed preclinical models that don't have an intact adaptive immune system. Although the authors' assertion may be correct, whether it is physiologically relevant in the presence of adaptive immunity is not addressed by the model systems used. For starter, subcutaneous GBM tumors are not physiologic and the author focused on a limited number of cytokines (i.e., CCL2 and CCL5) as indicative of macrophage recruitment.
These same chemokines can also recruit adaptive immune cells including cDCs, pDCs and even activated and memory T cells, which are absent in these models. A broader profile of cytokines/chemokines should be included (see above about the potential effects on proinflammatory cytokines that are not included in the analysis). In addition, there are several syngeneic mouse models of GBM that the authors should use instead to dissect the immune effects of the proposed cGAS-DNA-PK cooperation taking into account both the adaptive and innate immune systems.
Here, we wish to stress that orthotopic transplants have been performed in zebrafish (ie: a physiologic localization), which is the *in vivo* set up where we monitor macrophage recruitment. Indeed, this model does not capture all innate immune cells, but is sufficient to show recruitment of macrophages. In addition, the nude mouse model used in the study does not possess dendritic cells. Therefore, it is reasonable to hypothesize that cells of the macrophage lineage alone are sufficient to prevent the tumorigenesis of cGAS-expressing T98G cells. Finally, the analysis of the contribution of adaptive immune cells is beyond the scope of the present manuscript, hence the use of syngeneic mouse models did not appear essential to the conclusions that are made – and could potentially complexify our read-outs.
In order to broaden our observations, we have now included additional cytokines and chemokines including IFN Lambda, CCL3 and IL6 (Fig EV5E).

Minor comments:
1)gRNA labeling is not consistent. Sometimes it's just the gene name (e.g., STING), sometime it's STING-/-.
We thank the reviewer for spotting this mistake which we have now corrected.

2)A composite drawing summarizing the data will be helpful.
A composite drawing has been added to Fig 6J to summarize our data.

Referee #3:
Comments
In this study, the authors nicely demonstrated that DNA-PK DNA repair complex drives cGAS-independent inflammatory responses in glioblastoma cells. Moreover, the catalytic activity of DNA-PK is critical for cGAS-dependent cGAMP production and optimal downstream signaling. Furtherly, the re-expression of cGAS in glioblastoma cancer cells resulted in enhanced expression of chemokines that promote macrophage recruitment in the tumor microenvironment. The authors also provided data the expression of both cGAS and DNA-PKcs increased with tumor grade. Overall, the data are convincing and the findings are helpful to understand the role of DNA-PK in genotoxic stress-induced inflammatory response. However, there are major concerns need to be addressed.

1. One important finding of this study is that DNA-PK promotes DNA and chemotherapy-associated inflammatory responses independent of cGAS in the context of glioblastoma cancer cells. However, DNA-PK has been previously demonstrated to be an additional DNA senor driving STING-independent DNA sensing pathway (SIDSP) specifically in human cells (PMID: 31980485). This markedly undermined the novelty of current study. Also, is the finding reported by the authors in this study specific to human-derived glioblastoma cells? How about mouse glioblastoma cells?
SIDSP was previously described to operate alongside the cGAS-STING axis in immune cells, to be non-functional in murine cells and to not be involved in chemotherapy-associated IFN responses. Here, we demonstrate important points with regard to the relationship between DNA-PK and cGAS-dependent type I IFN responses: (1) we show that differently from SIDSP, DNA-PK controls Interferon responses in cGAS-deficient cells, including in response to chemotherapy, and that cGAS and DNA-PK cooperate to promote inflammatory responses, notably through a process that involves DNA-PK-dependent cGAS activation.
Importantly, we now provide data showing that the synergy between cGAS and DNA-PK can be measured in murine cells, using both the GL261 murine glioblastoma cell line (Fig EV3J), but also mouse embryonic fibroblasts (Fig EV3H-I)

2. As the authors mentioned in the introduction part, previous work reported that DNA-PK inhibited cGAS activation by direct modulating its phosphorylation on T68/S213 (PMID: 33273464) or by inducing cytosolic translocation of PARP1 (PMID: 35460603). In this study, the authors proposed that DNA-PK mediated the phosphorylation of cGAS on S435 to increase its catalytic activity. How can the authors reconcile their findings with previous report.
We now discuss our data in light of these manuscripts (pages 9-10).

3. Figure 1, the authors employed camptothecin as a genotoxic agent to induce the accumulation of cytosolic DNA and examine its sensing mechanism. How about other genotoxic agents such as cisplatin, etoposide.

We now provide data showing that etoposide treatment leads to DNA-PK-dependent activation of type I IFN responses (Fig EV1L).

4. Figure 2A, dsDNA stimulation caused cytosolic translocation of DNA-PKcs and phosphorylated DNA-PKcs. This is interesting and the authors should at least discuss this. An ensuing question is will this also happen when cGAS is expressed?
We now discuss the fact that dsDNA stimulation induces cytosolic translocation of DNA-PKcs and provide additional data showing that such DNA-PKcs translocation occurs regardless of cGAS expression (Fig EV3A).

Figure 2C, the knockdown of KU70 seemed to cause the reduced expression of KU80 and DNA-PKcs.
Knockdown of KU70 is known to lead to destabilization of the DNA-PK complex (PMID: 8700231; PMID: 9223317). This is now clarified in the text.
Figure 2I, KU70 and KU80 should be placed into the diagram.
KU70 and KU80 have now been added to the diagrams for increased clarity (See Fig 2I, 3L and 6J).

5. Figure 3, the authors conclude that DNA-PK and cGAS synergized to induce type I interferon response to DNA stimulation. However, in most cases, it hard to evaluate whether those effects are synergistic or additive.
We now indicate the values on the graphs of Fig 3, so that it can be readily appreciated that there is a synergistic and not additive effect.

Figure 3D and 3E, the experiments were performed with DNA-PK inhibitor NU7441, cGAS inhibitors should also be introduced.
Although this experiment being of interest, we did not succeed at identifying a dose of cGAS inhibitor that was efficient in our cells.

Figure 3H, it's striking to find that NU7441 completely abolished the enzymatic activity of cGAS, indicating that at least in THP-1 cells DNA-PK is an upstream regulator of cGAS and cGAS is dominant receptor for dsDNA, which is against with the hypothesis on the synergy of DNA-PK and cGAS in sensing DNA.
Indeed, the decrease of cGAS activity is dramatic but not completely abolished. Rather it is below the detection threshold of our assay. Testament to residual cGAS activity, we provide below a WB showing low pSTING levels (Fig D), that are indicative of STING activation, suggesting residual cGAMP production.
Based on the additional pulldowns presented in the revised manuscript and on recently published work (PMID: 36070696) indicating that KU70 potentiates cGAS activity, it is reasonable to conclude that (1) DNA-PK is able to directly trigger type I IFN responses (independently of the cGAS-STING axis), (2) DNA-PK favours cGAS activity when the cGAS-STING axis is functional. The synergy between cGAS and DNA-PK thus operates at 2 levels: both cGAS and DNA-PK favour IRF3 activation and DNA-PK also boosts cGAS activation. No herein provided data allows establishing a hierarchy between cGAS and DNA-PK for dsDNA sensing.

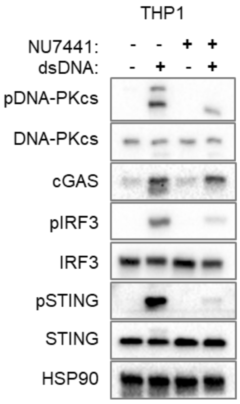

Figure D. THP1 cells were challenged or not with dsDNA for 6 h, in the presence or not of the NU7441 DNA-PKcs inhibitor, prior WB analysis using indicated antibodies.

6. Figure 4 and 5, the authors demonstrated that cGAS re-expression in glioblastoma cancer cells impaired tumorigenesis and promoted macrophage recruitment, however, whether these processes are dependent on the cooperation of DNA-PK and cGAS are not known. The authors should provide additional evidence (eg. by introducing DNA-PK inhibitor) to strengthen their conclusions.

This question is similar to what is asked by referee 1, point 8. We agree that the role of cooperation between DNA-PK and cGAS in glioblastoma models is not assessed in the present manuscript, owing to the absence of NU7441 treatment in in vivo tumorigenesis models. This experiment was not performed because experiments performed on spheroids of glioblastoma revealed that DNA-PK inhibition in cGAS expressing cells leads to high mortality rates upon prolonged treatment [**Figure C -** Figure shown to referees but removed from RPF]. Indeed, DNA-PK inhibition, in addition to inhibition of the associated inflammatory cytokine expression, leads to accumulation of DNA-damage in cancer cells. This in conjunction with cGAS expression, that is also reported to repress HR (PMID: 30356214; PMID: 31544964), can be expected to lead to levels of DNA damage incompatible with cell survival. In these conditions, analysis of inflammatory profiles was not possible.

7: Figure 6F, the PRKDClowMB21D1low should also be included.

We have now included this population on the graph as requested by the reviewer.

Minors

1. The phrase "immunogenicity" used in title is not accurate.

We have now changed the title to remove the word immunogenicity.

2. In the 2nd paragraph of introduction part, the protumor effects cGAS-STING were not fully summarized.

- We now provide more information of the pro-tumorigenic effect of the cGAS-STING axis.

3. The reference Wang, Zhao et al., 2022 is referred in the text of introduction part but is not listed in the reference.

- We have now verified that Wang, Zhao et al. 2022 is included in the reference list.

1st Revision - Editorial Decision **29th Nov 2022**

Dear Nadine,

Thank you for submitting your revised manuscript to The EMBO Journal. Your study has now been seen by referees #1 and 2. As you can see from the comments below, the referees appreciate the introduced changes.

They have a few remaining comments that I would like to ask you to take into consideration in a final revision.

When you submit the revised version will you also please take care of the following points?

- The data availability section should "only" list datasets that were generated in the reported study and not datasets previously reported. https://www.embopress.org/page/journal/14602075/authorguide#dataavailability
I see that you list the glioblastoma patient database in results and M&M and that should be fine.
Go ahead and remove the sentence: requests for data and reagents should be directed to...
If your study does not include datasets, please insert the following statement: This study includes no data deposited in external repositories.

- please check the funding information and make sure that it matches between the MS and online submission system.

- COI needs to be re-labeled as Disclosure and competing interests statement

- Please remove the Authors Contributions from the manuscript. The 'Author Contributions' section is replaced by the CRediT contributor roles taxonomy to specify the contributions of each author in the journal submission system. Please use the free text box in the 'author information' section of the manuscript submisssion system to provide more detailed descriptions (e.g., 'X provided intracellular Ca++ measurements in fig Y')

- I see that you have included a synopsis image. Please also upload a synopsis text => summary statement plus 3-5 bullet points describing the key findings of the

- Our publisher has also done their pre-publication check on your manuscript. When you log into the manuscript submission system you will see the file "Data Edited Manuscript file". Please take a look at the word file and the comments regarding the figure legends and respond to the issues.

- Table EV1 should be uploaded as a separate file.

- When you submit the revised MS version please submit and point-by-point response and a "clean" MS version without text markups.

That should be all - congratulations on a nice study!

Best Karin

Karin Dumstrei, PhD
Senior Editor
The EMBO Journal

Guide For Authors: https://www.embopress.org/page/journal/14602075/authorguide

Use the link below to submit your revision:

https://emboj.msubmit.net/cgi-bin/main.plex

Referee #1:

In this manuscript, the authors examine the function of DNA-PK and cGAS in the response to cytosolic DNA and DNA damage in glioblastoma cells. They describe cGAS- and STING-independent activation of the interferon response by DNA-PK, and also a synergistic relationship with cGAS where DNA-PK enhances cGAS function.
The data presented is clear, and additional controls have made this version of the manuscript more solid and convincing. I only

have minor comments on the manuscript in its current form:
1. The title was changed from the previous version, but still does not fully reflect the focus of this study. In my opinion, the title should mention both cGAS- and STING-independent signalling, as well as the synergy with cGAS - given the controversies in the field that this manuscript is attempting to reconcile.
2. The significance of the timing of inflammatory responses following detection of cytosolic DNA is mentioned in the discussion. In that light, it would be useful if the timing of the treatments (e.g. 6h for DNA transfection and 48 or 72h for CPT treatment) was included in the figures themselves (particularly fig. 1). Also, the authors propose that DNA-PK may have stimulatory and inhibitory roles on cGAS activity at different time points - would the authors be able to conduct a time course analysis (e.g. by qRT-PCR) to confirm or refute this hypothesis?
3.) The cGAS- and STING-independence of DNA-PK-induced IRF3 activation is already mentioned on p5, based at that point only on the inability to detect phospho-STING by immunoblotting. This is somewhat premature, and STING-independence should only be mentioned later when STING-deletion cells are described.

Overall, I feel that the authors have addressed many of my previous concerns, and I believe this study adds significant evidence to the DNA sensing field in the context of cancer.

Referee #2:

The authors have adequately addressed most of this reviewer's previous major comments. One remaining point is the macrophage recruitment experiment. The contention that this reviewer wished to convey is that showing macrophage recruitment in a system in which macrophages are the predominant immune cells is not a surprise and may not address the physiologic relevance of the findings. Although the Zebra fish experiments are informative, they should not be equated to those performed in a mammal for the questions the authors wish to address. It is this reviewer's opinion that if the authors do not wish to perform the same experiment in an immune competent mammal model to study the true effects of the cGAS-DNA-PK on the full immune system in a mammal, the conclusions in this section need to be toned down and alternate interpretation included in the discussion to reflect the limitation of the data and experimental set-up.

Referee #1:

In this manuscript, the authors examine the function of DNA-PK and cGAS in the response to cytosolic DNA and DNA damage in glioblastoma cells. They describe cGAS- and STING-independent activation of the interferon response by DNA-PK, and also a synergistic relationship with cGAS where DNA-PK enhances cGAS function.

The data presented is clear, and additional controls have made this version of the manuscript more solid and convincing. I only have minor comments on the manuscript in its current form:

1. The title was changed from the previous version, but still does not fully reflect the focus of this study. In my opinion, the title should mention both cGAS- and STING-independent signalling, as well as the synergy with cGAS - given the controversies in the field that this manuscript is attempting to reconcile.

According the reviewer's suggestion we modified the title to include both the notion of synergy between DNA-PK and cGAS associated inflammatory responses. However, due to the limitation in character count, we could not include the notion of STING dependency. To clarify this point, we have now included a synopsis text and bullet point recapitulating the findings.

2. The significance of the timing of inflammatory responses following detection of cytosolic DNA is mentioned in the discussion. In that light, it would be useful if the timing of the treatments (e.g. 6h for DNA transfection and 48 or 72h for CPT treatment) was included in the figures themselves (particularly fig. 1). Also, the authors propose that DNA-PK may have stimulatory and inhibitory roles on cGAS activity at different time points - would the authors be able to conduct a time course analysis (e.g. by qRT-PCR) to confirm or refute this hypothesis?

The time points used for stimulation of type I IFN responses are indicated in the legends. In case of dsDNA transfection the time point is always of 6 hours. Because it is only in case of chemotherapy treatment that the time points differ, we have now indicated those time points directly on all relevant figures. That DNA-PK activity would initially synergize with the cGAS-STING pathway, and at later time point inhibit cGAS activity is a speculation based on the literature, to reconcile the observed discrepancies. Note however that a manuscript supportive of our mechanism has recently been published (PMID: 36070696). Thorough assessment of whether (and how) such a switch would occur is beyond the scope of the present manuscript. We have however made this point more precise in the discussion.

3.) The cGAS- and STING-independence of DNA-PK-induced IRF3 activation is already mentioned on p5, based at that point only on the inability to detect phospho-STING by immunoblotting. This is somewhat premature, and STING-independence should only be mentioned later when STING-deletion cells are described.

In agreement with the reviewer's suggestion, we have now removed this occurrence of "Sting-independence".

Overall, I feel that the authors have addressed many of my previous concerns, and I believe this study adds significant evidence to the DNA sensing field in the context of cancer.

Referee #2:

The authors have adequately addressed most of this reviewer's previous major comments. One remaining point is the macrophage recruitment experiment. The contention that this reviewer wished to convey is that showing macrophage recruitment in a system in which macrophages are the predominant immune cells is not a surprise and may not address the physiologic relevance of the findings. Although the Zebra fish experiments are informative, they should not be equated to those performed in a mammal for the questions the authors wish to address. It is this reviewer's opinion that if the authors do not wish to perform the same experiment in an immune competent mammal model to study the true effects of the cGAS-DNA-PK on the full immune system in a mammal, the

conclusions in this section need to be toned down and alternate interpretation included in the discussion to reflect the limitation of the data and experimental set-up.

In agreement with the reviewer's suggestion, we have now toned-down the conclusions from the section where Zebrafish are used. In addition, we clearly state in the discussion that the contribution of adaptive immune cells is not tested using this model.

2nd Revision - Editorial Decision 2nd Dec 2022

Dear Nadine,

Thanks for submitting your revised manuscript I have now had a chance to take a careful look at everything and all look good.

I am therefore very pleased to accept the MS for publication here.

Congratulations on a nice study

With best wishes

Karin

Karin Dumstrei, PhD
Senior Editor
The EMBO Journal

Please note that it is EMBO Journal policy for the transcript of the editorial process (containing referee reports and your response letter) to be published as an online supplement to each paper. If you do NOT want this, you will need to inform the Editorial Office via email immediately. More information is available here:
https://www.embopress.org/page/journal/14602075/authorguide#transparentprocess

Your manuscript will be processed for publication in the journal by EMBO Press. Manuscripts in the PDF and electronic editions of The EMBO Journal will be copy edited, and you will be provided with page proofs prior to publication. Please note that supplementary information is not included in the proofs.

You will be contacted by Wiley Author Services to complete licensing and payment information. The required 'Page Charges Authorization Form' is available here: https://www.embopress.org/pb-assets/embo-site/tej_apc.pdf - please download and complete the form and return to embopressproduction@wiley.com

EMBO Press participates in many Publish and Read agreements that allow authors to publish Open Access with reduced/no publication charges. Check your eligibility: https://authorservices.wiley.com/author-resources/Journal-Authors/open-access/affiliation-policies-payments/index.html

Should you be planning a Press Release on your article, please get in contact with embojournal@wiley.com as early as possible, in order to coordinate publication and release dates.

If you have any questions, please do not hesitate to call or email the Editorial Office. Thank you for your contribution to The EMBO Journal.

** Click here to be directed to your login page: https://emboj.msubmit.net

EMBO Press Author Checklist

Corresponding Author Name: LAGUETTE	
Journal Submitted to: EMBO Journal	
Manuscript Number: EMBOJ-2022-111961R	

USEFUL LINKS FOR COMPLETING THIS FORM
The EMBO Journal - Author Guidelines
EMBO Reports - Author Guidelines
Molecular Systems Biology - Author Guidelines
EMBO Molecular Medicine - Author Guidelines

Reporting Checklist for Life Science Articles (updated January
This checklist is adapted from Materials Design Analysis Reporting (MDAR) Checklist for Authors. MDAR establishes a minimum set of requirements in transparent reporting in the life sciences (see Statement of Task: 10.31222/osf.io/9sm4x). Please follow the journal's guidelines in preparing your
Please note that a copy of this checklist will be published alongside your article.

Abridged guidelines for figures
1. Data
The data shown in figures should satisfy the following conditions:
- the data were obtained and processed according to the field's best practice and are presented to reflect the results of the experiments in an accurate and unbiased manner.
- ideally, figure panels should include only measurements that are directly comparable to each other and obtained with the same assay.
- plots include clearly labeled error bars for independent experiments and sample sizes. Unless justified, error bars should not be shown for technical
- if n<5, the individual data points from each experiment should be plotted. Any statistical test employed should be justified.
- Source Data should be included to report the data underlying figures according to the guidelines set out in the authorship guidelines on Data

2. Captions
Each figure caption should contain the following information, for each panel where they are relevant:
- a specification of the experimental system investigated (eg cell line, species name).
- the assay(s) and method(s) used to carry out the reported observations and measurements.
- an explicit mention of the biological and chemical entity(ies) that are being measured.
- an explicit mention of the biological and chemical entity(ies) that are altered/varied/perturbed in a controlled manner.
- the exact sample size (n) for each experimental group/condition, given as a number, not a range;
- a description of the sample collection allowing the reader to understand whether the samples represent technical or biological replicates (including how many animals, litters, cultures, etc.).
- a statement of how many times the experiment shown was independently replicated in the laboratory.
- definitions of statistical methods and measures:
  - common tests, such as t-test (please specify whether paired vs. unpaired), simple χ2 tests, Wilcoxon and Mann-Whitney tests, can be unambiguously identified by name only, but more complex techniques should be described in the methods section;
  - are tests one-sided or two-sided?
  - are there adjustments for multiple comparisons?
  - exact statistical test results, e.g., P values = x but not P values < x;
  - definition of 'center values' as median or average;
  - definition of error bars as s.d. or s.e.m.

Please complete ALL of the questions below.
Select "Not Applicable" only when the requested information is not relevant for your study.

Materials

Newly Created Materials	Information included in the manuscript?	In which section is the information available? (Reagents and Tools Table, Materials and Methods, Figures, Data Availability Section)
New materials and reagents need to be available; do any restrictions apply?	Yes	Materials and Methods

Antibodies	Information included in the manuscript?	In which section is the information available? (Reagents and Tools Table, Materials and Methods, Figures, Data Availability Section)
For **antibodies** provide the following information:<br>- Commercial antibodies: RRID (if possible) or supplier name, catalogue number and or/clone number<br>- Non-commercial: RRID or citation	Yes	Reagents and Tools Table

DNA and RNA sequences	Information included in the manuscript?	In which section is the information available? (Reagents and Tools Table, Materials and Methods, Figures, Data Availability Section)
**Short novel DNA or RNA including primers, probes**: provide the sequences.	Yes	Table EV1

Cell materials	Information included in the manuscript?	In which section is the information available? (Reagents and Tools Table, Materials and Methods, Figures, Data Availability Section)
**Cell lines:** Provide species information, strain. Provide accession number in repository **OR** supplier name, catalog number, clone number, and/**OR** RRID.	Yes	Reagents and Tools Table and Materials and Methods
**Primary cultures:** Provide species, strain, sex of origin, genetic modification status.	Yes	Reagents and Tools Table and Materials and Methods
Report if the cell lines were recently **authenticated** (e.g., by STR profiling) and tested for mycoplasma contamination.	Not Applicable	

Experimental animals	Information included in the manuscript?	In which section is the information available? (Reagents and Tools Table, Materials and Methods, Figures, Data Availability Section)
**Laboratory animals or Model organisms:** Provide species, strain, sex, age, genetic modification status. Provide accession number in repository **OR** supplier name, catalog number, clone number, **OR** RRID.	Yes	Reagents and Tools Table and Materials and Methods
**Animal observed in or captured from the field:** Provide species, sex, and age where possible.	Not Applicable	
Please detail **housing and husbandry conditions**.	Yes	Materials and Methods

Plants and microbes	Information included in the manuscript?	In which section is the information available? (Reagents and Tools Table, Materials and Methods, Figures, Data Availability Section)
**Plants:** provide species and strain, ecotype and cultivar where relevant, unique accession number if available, and source (including location for collected wild specimens).	Not Applicable	
**Microbes:** provide species and strain, unique accession number if available, and source.	Not Applicable	

Human research participants	Information included in the manuscript?	In which section is the information available? (Reagents and Tools Table, Materials and Methods, Figures, Data Availability Section)
If collected and within the bounds of privacy constraints report on age, sex and gender or ethnicity for all study participants.	Not Applicable	

Core facilities	Information included in the manuscript?	In which section is the information available? (Reagents and Tools Table, Materials and Methods, Figures, Data Availability Section)
If your work benefited from core facilities, was their service mentioned in the acknowledgements section?	Yes	Acknowledgements

Design

Study protocol	Information included in the manuscript?	In which section is the information available? (Reagents and Tools Table, Materials and Methods, Figures, Data Availability Section)
If study protocol has been **pre-registered, provide DOI in the manuscript**. For clinical trials, provide the trial registration number **OR** cite DOI.	Not Applicable	
Report the **clinical trial registration number** (at ClinicalTrials.gov or equivalent), where applicable.	Not Applicable	

Laboratory protocol	Information included in the manuscript?	In which section is the information available? (Reagents and Tools Table, Materials and Methods, Figures, Data Availability Section)
Provide DOI OR other citation details if **external detailed step-by-step protocols** are available.	Yes	Materials and Methods

Experimental study design and statistics	Information included in the manuscript?	In which section is the information available? (Reagents and Tools Table, Materials and Methods, Figures, Data Availability Section)
Include a statement about **sample size** estimate even if no statistical methods were used.	Yes	Materials and Methods
Were any steps taken to minimize the effects of subjective bias when allocating animals/samples to treatment (e.g. **randomization procedure**)? If yes, have they been described?	Yes	Materials and Methods
Include a statement about **blinding** even if no blinding was done.	Yes	Materials and Methods
Describe **inclusion/exclusion criteria** if samples or animals were excluded from the analysis. Were the criteria pre-established?<br><br>If sample or data points were omitted from analysis, report if this was due to attrition or intentional exclusion and provide justification.	Not Applicable	
For every figure, are **statistical tests** justified as appropriate? Do the data meet the assumptions of the tests (e.g., normal distribution)? Describe any methods used to assess it. Is there an estimate of variation within each group of data? Is the variance similar between the groups that are being statistically compared?	Yes	Materials and Methods and Figures

Sample definition and in-laboratory replication	Information included in the manuscript?	In which section is the information available? (Reagents and Tools Table, Materials and Methods, Figures, Data Availability Section)
In the figure legends: state number of times the experiment was **replicated** in laboratory.	Yes	Figures
In the figure legends: define whether data describe **technical or biological replicates**.	Yes	Figures

Ethics

Ethics	Information included in the manuscript?	In which section is the information available? (Reagents and Tools Table, Materials and Methods, Figures, Data Availability Section)
Studies involving **human participants**: State details of **authority granting ethics approval** (IRB or equivalent committee(s), provide reference number for approval.	Yes	Materials and Methods
Studies involving **human participants**: Include a statement confirming that **informed consent** was obtained from all subjects and that the experiments conformed to the principles set out in the WMA Declaration of Helsinki and the Department of Health and Human Services Belmont Report.	Not Applicable	
Studies involving **human participants:** For publication of **patient photos**, include a statement confirming that consent to publish was obtained.	Not Applicable	
Studies involving experimental **animals**: State details of **authority granting ethics approva**l (IRB or equivalent committee(s), provide reference number for approval. Include a statement of compliance with ethical regulations.	Yes	Materials and Methods
Studies involving **specimen and field samples:** State if relevant **permits** obtained, provide details of authority approving study; if none were required, explain why.	Not Applicable	

Dual Use Research of Concern (DURC)	Information included in the manuscript?	In which section is the information available? (Reagents and Tools Table, Materials and Methods, Figures, Data Availability Section)
Could your study fall under dual use research restrictions? Please check biosecurity documents and list of **select agents and toxins** (CDC): https://www.selectagents.gov/sat/list.htm	Not Applicable	
If you used a select agent, is the security level of the lab appropriate and reported in the manuscript?	Not Applicable	
If a study is subject to dual use research of concern regulations, is the name of the **authority granting approval and reference number** for the regulatory approval provided in the manuscript?	Not Applicable	

Reporting

The MDAR framework recommends adoption of discipline-specific guidelines, established and endorsed through community initiatives. Journals have their own policy about requiring specific guidelines and recommendations to complement MDAR.

Adherence to community standards	Information included in the manuscript?	In which section is the information available? (Reagents and Tools Table, Materials and Methods, Figures, Data Availability Section)
State if relevant guidelines or checklists (e.g., **ICMJE, MIBBI, ARRIVE, PRISMA**) have been followed or provided.	Not Applicable	
For **tumor marker prognostic studies,** we recommend that you follow the **REMARK** reporting guidelines (see link list at top right). See author guidelines, under 'Reporting Guidelines'. Please confirm you have followed these guidelines.	Not Applicable	
For **phase II and III randomized controlled trials**, please refer to the **CONSORT** flow diagram (see link list at top right) and submit the CONSORT checklist (see link list at top right) with your submission. See author guidelines, under 'Reporting Guidelines'. Please confirm you have submitted this list.	Not Applicable	

Data Availability

Data availability	Information included in the manuscript?	In which section is the information available? (Reagents and Tools Table, Materials and Methods, Figures, Data Availability Section)
Have **primary datasets** been deposited according to the journal's guidelines (see 'Data Deposition' section) and the respective accession numbers provided in the Data Availability Section?	Not Applicable	
Were **human clinical and genomic datasets** deposited in a public access-controlled repository in accordance to ethical obligations to the patients and to the applicable consent agreement?	Not Applicable	
Are **computational models** that are central and integral to a study available without restrictions in a machine-readable form? Were the relevant accession numbers or links provided?	Not Applicable	
If publicly available data were reused, provide the respective **data citations in the reference list**.	Yes	Materials and Methods
